# Impact of allogeneic dental pulp stem cell injection on tissue regeneration in periodontitis: a multicenter randomized clinical trial

**DOI:** 10.1038/s41392-025-02320-w

**Published:** 2025-07-31

**Authors:** Yi Liu, Yitong Liu, Jingchao Hu, Jianhui Han, Lin Song, Xu Liu, Nannan Han, Xia Xia, Jia He, Hongfang Meng, Meng Wan, Haojie Wang, Xiaodan Liu, Zhanyi Gao, Xiao Wang, Chutse Wu, Songlin Wang

**Affiliations:** 1 Laboratory of Tissue Regeneration and Immunology and Department of Periodontics, Beijing Key Laboratory of Tooth Regeneration and Function Reconstruction, School of Stomatology, Capital Medical University, Beijing, China; 2 Beijing Laboratory of Oral Health, Capital Medical University, Beijing, China; 3 Immunology Research Center for Oral and Systemic Health, Beijing Friendship Hospital, Capital Medical University, Beijing, China; 4 Department of Radiology, School of Stomatology, Capital Medical University, Beijing, China; 5 Beijing SH Biotechnology Co. Ltd., Beijing, China; 6 Department of Stomatology, Peking University Third Hospital, Beijing, China; 7 Department of Biochemistry and Molecular Biology, School of Basic Medical Sciences, Capital Medical University, Beijing, China; 8 Laboratory of Homeostatic Medicine, School of Medicine and SUSTech Homeostatic Medicine Institute, Southern University of Science and Technology, Shenzhen, China; 9 Research Units of Tooth Development and Regeneration, Chinese Academy of Medical Sciences, Beijing, China

**Keywords:** Medical research, Stem cells

## Abstract

Periodontitis causes the destruction of tooth-supporting tissues, and current therapies for periodontal regeneration are invasive. In this study, a human dental pulp stem cell (DPSC; hDP-MSC) injection was developed to promote periodontal regeneration through a non-invasive procedure. A total of 132 patients with chronic periodontitis (158 teeth) from two centers in China were included. Thirty-six were randomly assigned to different DPSC dose groups (ranging from 1 × 10^6^ to 1 × 10^7^ DPSCs per tooth, with nine injected with saline only), and 96 were randomly assigned to a single-injection group (1 × 10^7^/0.6 mL DPSCs), a double-injection group (1 × 10^7^/0.6 mL DPSCs × 2), or a saline group, in a 1:1:1 ratio. At 6 months post-therapy, attachment loss (AL), periodontal probing depth (PD), gingival recession (GR), tooth mobility (TM), and bone defect depth (BDD) were examined. The primary outcome was AL. DPSC injection resulted in greater improvement in BDD (0.30 ± 0.484 mm) compared to saline injection (0.04 ± 0.315 mm). Post hoc analysis showed that DPSC injection had significantly better outcomes in patients with stage III periodontitis (AL ≥ 5 mm): 54 patients received DPSCs, and 40 received saline. AL improved by 1.67 ± 1.508 mm in the DPSC group (26.81% improvement) and by 1.03 ± 1.310 mm in the saline group (17.43% improvement). The therapeutic effects encompassed improvements in both soft and hard tissues. In summary, DPSC injection was safe and improved clinical outcomes compared to saline injection in patients with stage III periodontitis. Larger trials are warranted to validate these findings (ClinicalTrials.gov registration: NCT05924373).

## Introduction

Many species of bacteria are commensals in the human oral cavity.^1,2^ Periodontitis, one of the most common oral diseases, is a chronic inflammatory condition triggered by a limited diversity of pathogenic bacteria in the mouth.^3^ It affects more than 700 million people worldwide^4^ and leads to the destruction of bone and soft tissue, as well as systemic inflammatory manifestations.^5^ Epidemiological studies have also associated severe periodontitis with increased risks of cancer initiation and progression.^6^

In critical cases, treatment typically involves guided tissue regeneration—a technique aimed at restoring the functional attachment apparatus by combining bone graft surgery with the use of bioactive agents, such as growth factors.^7–9^ However, surgical procedures often cause significant discomfort and pain for patients. Moreover, tissue regeneration is often incomplete in such cases, with an elevated risk of infection and uncontrolled tissue growth.^10^

A promising alternative to these surgical procedures is the transplantation of dental pulp stem cells into areas with bone defects. In animal models, this technique has been successfully applied in pigs, facilitating faster and simpler recovery.^11^ Although many studies have attempted to regenerate periodontal bone defects using this strategy in humans,^12,13^ the primary challenge remains that all current treatments still rely on invasive surgical methods. In addition, applications of dental pulp stem cells have not yet enabled large-scale stem cell drug production. Thus, developing new drugs based on this technique—particularly for minimally invasive therapy—is a key focus of current research.

For decades, our team has conducted extensive in vitro and in vivo research on novel stem cell drugs, ultimately obtaining approval for the first odontogenic stem cell drug for the clinical treatment of periodontitis in humans (approved as an Investigational New Drug for periodontitis in China, CXSL1700137). This approval was based on detailed comparisons of immunogenicity, safety, and efficacy between autologous and allogeneic stem cells,^14,15^ and also considered stem cells derived from different sources.^16–18^ Moreover, we investigated the regulatory effects of allogeneic stem cells on the immune microenvironment and host stem cells.^19–23^ Our systematic and comprehensive research framework for stem cell therapy is currently among the most advanced in the world. Nevertheless, the efficacy and safety of this approach must be rigorously evaluated before large-scale clinical application of stem cell drugs can be pursued.

Therefore, in this study, we conducted two randomized, placebo-controlled clinical trials to evaluate the efficacy and safety of dental pulp stem cell injection in humans for the first time. Notably, the development of a minimally invasive clinical treatment via stem cell injection—building on our stem cell implantation methods—represents a significant breakthrough in periodontitis treatment technology. This marks a critical step toward the routine clinical use of dental pulp stem cell injections for periodontitis.^11^

## Results

### Quality assessment of the drug preparation

During primary cell isolation, spindle-like cells were observed around the digested pulp tissues on day 10. On day 14, the primary cells were digested with trypsin and transferred to a serum-free medium. Quality control results showed that the banked cells were spindle-shaped and arranged in a spiral pattern in culture flasks (Supplementary Fig. 2a), retracting into a closely packed, rounded shape when trypsinized. Under induction conditions, osteogenesis (Supplementary Fig. 2b) and adipogenesis (Supplementary Fig. 2c) were successfully observed.

To determine whether the isolated cells were indeed mesenchymal stem cells, we analyzed their expression of CD73, CD90, and CD105, which are markers typically associated with this cell type.^24^ As expected, the cells showed positive expression of these markers. Furthermore, we confirmed that they did not express common surface markers of hematopoietic or immune lineage cells—namely, CD19, CD34, CD45, CD11b, and HLA-DR (Supplementary Fig. 2d)—thereby reinforcing their classification as mesenchymal stem cells and proving their characteristics of low immunogenicity. Additionally, a series of genes related to osteogenic differentiation ability were highly expressed in these cells during the process of osteogenic induction, including alkaline phosphatase (*Alp*), osteopontin (*Opn*), osterix (*Osx*), bone morphogenetic protein‌ 2 (*Bmp2*), runt-related transcription factor 2 (*Runx2*) (Supplementary Fig. 2e).

The dental pulp stem cell injection preparation was derived from these banked cells. Quality control results indicated low levels of bovine serum albumin, trypsin, collagenase I, and dispase II, and confirmed that the preparation was free of bacterial contamination. Prior to administration, the cell count was recorded as 1 × 10^7^ ± 2 × 10^6^ cells per 0.6 mL preparation, with cell viability reaching as high as 90% (Supplementary Table 4).

### Therapeutic and safety assessments

**Fig. 1:**
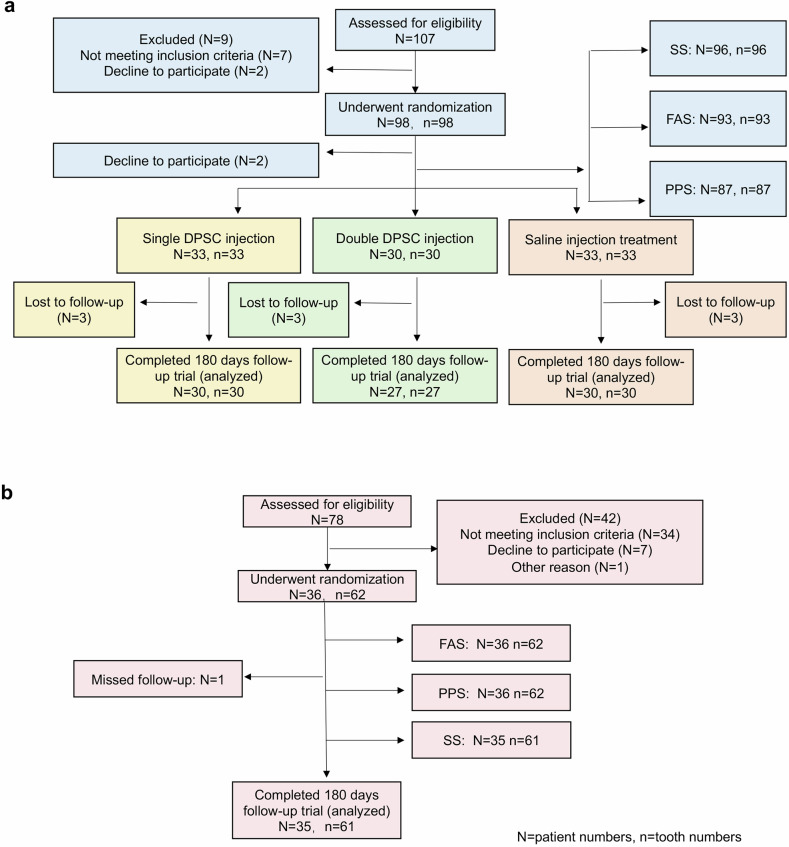
Randomization scheme and participant flow for (**a**) the investigator-initiated trial and (**b**) the Phase I trial. **DPSC** injection refers to the experimental group receiving dental pulp stem cells, while saline injection refers to the control group. **SS** denotes the Safety Set, which includes participants who received at least one dose of treatment and were monitored for safety; this set is used to assess adverse events and safety outcomes. **FAS** represents the Full Analysis Set, which includes all participants randomly assigned to a treatment group regardless of protocol adherence or study completion, reflecting treatment effects under real-world conditions. **PPS** refers to the Per Protocol Set, consisting of participants who completed the study and strictly followed the protocol, used for assessing efficacy under ideal conditions

The final all-randomized analysis sample included 96 participants from Beijing Stomatological Hospital (investigator-initiated trial) and 36 participants from Peking University Third Hospital (Phase I trial) (Fig. 1). Aside from sex, baseline demographic and clinical characteristics were generally similar between the two trials, with no statistically significant differences observed (Table 1; Supplementary Tables 5 and 6).

**Table 1:** Demographic characteristics of patients in the intention-to-treat population

Characteristic		
Age (year)	Number of cases (missing count)	132 (0)
	Mean (SD)	37.5 (8.47)
	Median	38
	Quartile	31.0, 43.5
	Minimum, maximum	21, 58
Sex	Male	56 (42.4)
	Female	76 (57.6)
Nationality	Han	119 (90.2)
	Others	13 (9.8)
Weight (kg)	Number of cases (missing count)	132 (0)
	Mean (SD)	65.73 (12.847)
	Median	63.25
	Quartile	56.50, 73.50
	Minimum, maximum	45.0, 120.0
Height (cm)	Number of cases (missing count)	132 (0)
	Mean (SD)	167.63 (7.603)
	Median	166
	Quartile	162.00, 172.00
	Minimum, maximum	152.0, 192.0
BMI (kg/m^2^)	Number of cases (missing count)	132 (0)
	Mean (SD)	23.24 (3.242)
	Median	22.6
	Quartile	20.95, 25.40
	Minimum, maximum	16.3, 34.7

There were no statistically significant differences in baseline periodontal characteristics among all participants in the two trials (P values for comparisons between any two randomized groups were >0.05; Supplementary Tables 5 and 6).

Dental pulp stem cell therapy demonstrated an excellent safety profile in this study, with no serious adverse events reported among the 132 participants (Table 2; Supplementary Tables 9 and 10). In the investigator-initiated trial, two drug-related adverse events occurred in the experimental group—specifically, toothache and injection site swelling—while one participant in the control group experienced an injection-related adverse event (injection site swelling). All of these adverse events were classified as grade 1 and resolved spontaneously without intervention. Similarly, in the Phase I trial, two participants in the 5 × 10^6^ cells/injection group experienced grade 1 adverse events (gum swelling and diarrhea) that may have been related to the injection; both recovered without treatment. These findings indicate that dental pulp stem cell injection is safe and well tolerated for the clinical treatment of periodontitis.

**Table 2:** Treatment-emergent adverse events associated with DPSC injection in the safety set of the investigator-initiated trial

	Single DPSC injection N = 33 n (%)		Double DPSC injection N = 30 n (%)		Saline injection N = 33 n (%)	
Injection site swelling	0 (0.0)		1 (3.3)		1 (3.0)	
Toothache	1 (3.0)		0 (0.0)		0 (0.0)	
Relationship with DPSCs	possible		Certain		certain	
Clinical outcome	disappear		Disappear		disappear	
Phase I trial	1 × 10^6^ DPSCs /1 tooth N = 3 n (%)	5 × 10^6^ DPSCs/1 tooth N = 6 n (%)	1 × 10^7^ DPSCs/1 tooth N = 6 n (%)	2 × 10^7^ DPSCs/2 teeth N = 6 n (%)	3–4 × 10^7^ DPSCs/3–4 teeth N = 6 n (%)	Saline injection N = 9 n (%)
At least 1 case of TEAE associated with the trial drug	0 (0.0)	2 (33.3)	0 (0.0)	0 (0.0)	0 (0.0)	0 (0.0)
Gastrointestinal system diseases	0 (0.0)	2 (33.3)	0 (0.0)	0 (0.0)	0 (0.0)	0 (0.0)
Diarrhea	0 (0.0)	1 (16.7)	0 (0.0)	0 (0.0)	0 (0.0)	0 (0.0)
Gingival swelling	0 (0.0)	1 (16.7)	0 (0.0)	0 (0.0)	0 (0.0)	0 (0.0)

After 6 months of follow-up, we analyzed the outcomes of the investigator-initiated trial. Supplementary Table 11 presents the treatment outcomes for the single and double dental pulp stem cell injection groups and the saline control group. By day 180, tooth attachment loss increased by 1.2 ± 1.90 mm in the single injection group (P = 0.36), 0.8 ± 1.03 mm in the double injection group (P = 0.95), and 0.8 ± 0.95 mm in the control group (P = 0.92). Although the results for probing depth and gingival recession were not statistically significant, the single injection group demonstrated a significant therapeutic effect in improving bone defect depth (P = 0.0083)—an outcome that is challenging to achieve with current non-surgical clinical treatments (Supplementary Table 11). While evaluating therapeutic efficacy was not the primary endpoint of the Phase I trial due to its small sample size, clinical outcomes for different doses of dental pulp stem cells are shown in Supplementary Table 12. These results indicate no statistically significant difference between the experimental and saline groups, likely due to the limited sample size.

### Clinical outcomes

During clinical treatment, we noticed that patients with varying levels of inflammation responded differently to dental pulp stem cell therapy. Consequently, post hoc analyses were conducted to evaluate the efficacy of injections at specific doses. Data from participants who received the same injection dose (the saline injection group and the 1 × 10^7^ DPSCs per tooth injection group) across both trials were pooled for analysis, and baseline characteristics for these combined participants were verified (Supplementary Table 13).

The results indicated that dental pulp stem cell injections at a dose of 1 × 10^7^ were more effective in patients with stage III periodontitis (attachment loss ≥5 mm) compared to those with stage II periodontitis (attachment loss <5 mm). In stage III periodontitis patients, the experimental group exhibited an improvement in attachment loss of 1.67 ± 1.508 mm (26.81% improvement), while the saline control group showed an improvement of 1.03 ± 1.310 mm (17.43% improvement) (P = 0.0338; 95% CI, −1.23 to −0.05). Additionally, the improvement in periodontal probing depth in the experimental group was 1.81 ± 1.490 mm, compared to 1.08 ± 1.289 mm in the control group (P = 0.0147; 95% CI, −1.31 to −0.15). Similarly, bone defect depth improved by 0.24 ± 0.471 mm in the experimental group, whereas the improvement was only 0.02 ± 0.348 mm in the control group (P = 0.0147; 95% CI, −0.39 to −0.04) (Table 3).

**Table 3:** Clinical outcomes of DPSC injection for periodontitis with varying degrees of inflammation (combined dataset)

			AL <5 mm				AL ≥5 mm			
			DPSC injection (n = 9)	Saline injection (n = 3)	P value (DPSC vs Saline)	Difference (95%CI)	DPSC injection (n = 54)	Saline injection (n = 40)	P value (DPSC vs Saline)	Difference (95% CI)
AL (mm) mean ± SD (95% CI)	Baseline		3.61 ± 0.601 (3.15, 4.07)	3.83 ± 0.289 (3.12, 4.55)	0.5599	−0.22 (−1.04, 0.60)	6.23 ± 1.393 (5.85, 6.61)	5.91 ± 1.377 (5.47, 6.35)	0.2729	0.32 (−0.26, 0.89)
	Day 90 change from baseline		−0.72 ± 0.795 (−1.33, −0.11)	−0.67 ± 0.289 (−1.38, 0.05)	0.9105	−0.06 (−1.13, 1.02)	−1.49 ± 1.276 (−1.84, −1.14)	−1.00 ± 1.068 (−1.34, −0.66)	0.0515	−0.49 (−0.98, 0.00)
	Day 180 change from baseline		−0.56 ± 0.950 (−1.29, 0.17)	-0.67 ± 0.289 (-1.38, 0.05)	0.8501	0.11 (−1.17, 1.39)	−1.67 ± 1.508 (−2.08, −1.26)	−1.03 ± 1.310 (−1.44, −0.61)	0.0338*	−0.64 (−1.23, −0.05)
PD (mm) mean ± SD (95% CI)	Baseline		3.61 ± 0.601 (3.15, 4.07)	3.83 ± 0.289 (3.12, 4.55)	0.5599	−0.22 (−1.04, 0.60)	5.79 ± 1.294 (5.43, 6.14)	5.61 ± 1.146 (5.25, 5.98)	0.4994	0.17 (−0.34, 0.69)
	Day 90 change from baseline		−0.56 ± 1.130 (−1.42, 0.31)	−0.83 ± 0.289 (−1.55, −0.12)	0.6913	0.28 (−1.24, 1.79)	−1.57 ± 1.326 (−1.94, −1.21)	−1.06 ± 1.069 (−1.40, -0.72)	0.0480*	−0.51 (−1.02, 0.00)
	Day 180 change from baseline		−0.67 ± 1.118 (−1.53, 0.19)	−0.83 ± 0.289 (−1.55, −0.12)	0.8092	0.17 (−1.33, 1.66)	−1.81 ± 1.490 (−2.21, −1.40)	−1.08 ± 1.289 (−1.49, −0.66)	0.0147*	−0.73 (−1.31, −0.15)
TM, n (%)	Basline	(—)	7 (77.78%)	3 (100.00%)	1.0000		36 (66.67%)	34 (85.00%)	0.0849	
		I°	2 (22.22%)				15 (27.78%)	4 (10.00%)		
		II°					3 (5.56%)	2 (5.00%)		
	Day 90	(—)	8 (88.89%)	3 (100.00%)	0.5637		42 (77.78%)	34 (85.00%)	0.2001	
		I°	1 (11.11%)				11 (20.37%)	4 (10.00%)		
		II°					1 (1.85%)	2 (5.00%)		
	D180	(—)	9 (100.00%)	3 (100.00%)	0.3918		49 (90.74%)	34 (85.00%)	0.0182*	
		I°					4 (7.41%)	4 (10.00%)		
		II°					1 (1.85%)	2 (5.00%)		
GR (mm) mean ± SD (95% CI)	Baseline		0.00 ± 0.000 (., .)	0.00 ± 0.000 (., .)		0.00 (., .)	0.44 ± 0.805 (0.22, 0.66)	0.31 ± 0.676 (0.10, 0.53)	0.4032	0.13 (−0.18, 0.44)
	Day 90 change from baseline		0.06 ± 0.167 (−0.07, 0.18)	0.17 ± 0.289 (−0.55, 0.88)	0.4178	−0.11 (−0.40, 0.18)	0.08 ± 0.657 (−0.10, 0.26)	0.06 ± 0.483 (−0.09, 0.22)	0.8658	0.02 (−0.22, 0.27)
	Day 180 change from baseline		0.22 ± 0.441 (−0.12, 0.56)	0.17 ± 0.289 (−0.55, 0.88)	0.8449	0.06 (−0.56, 0.67)	0.12 ± 0.726 (−0.08, 0.32)	0.05 ± 0.336 (-0.06, 0.16)	0.5709	0.07 (−0.18, 0.32)
BDD (mm) mean ± SD (95% CI)	Baseline		1.42 ± 0.974 (0.67, 2.17)	1.20 ± 0.693 (−0.52, 2.92)	0.7261	0.22 (−1.15, 1.60)	2.51 ± 1.283 (2.16, 2.86)	2.03 ± 1.460 (1.56, 2.50)	0.0960	0.48 (−0.09, 1.04)
	Day 90 change from baseline		−0.13 ± 0.374 (−0.42, 0.15)	−0.30 ± 0.173 (−0.73, 0.13)	0.4834	0.17 (−0.34, 0.68)	−0.16 ± 0.383 (−0.27, −0.06)	−0.08 ± 0.314 (−0.18, 0.02)	0.2519	−0.09 (−0.23, 0.06)
	Day 180 change from baseline		−0.18 ± 0.335 (−0.43, 0.08)	−0.30 ± 0.265 (−0.96, 0.36)	0.5814	0.12 (−0.36, 0.60)	−0.24 ± 0.471 (−0.37, −0.11)	−0.02 ± 0.348 (−0.13, 0.09)	0.0147*	−0.22 (−0.39, −0.04)

**Fig. 2:**
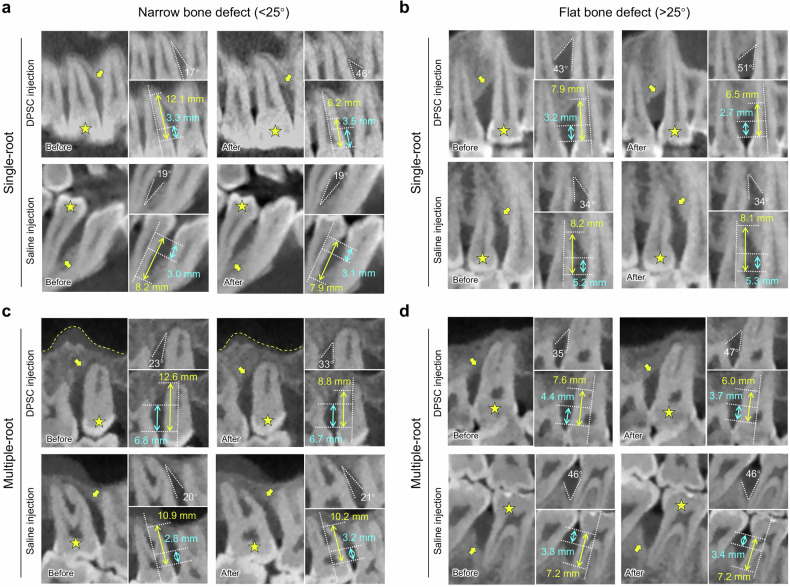
DPSC injection demonstrated greater efficacy in bone defect regeneration compared to saline injection. **a**, **c** In narrow initial bone defects, CBCT scans show that DPSC injection significantly reduced the alveolar bone resorption height at day 180 post-treatment. Notably, DPSC injection also reduced signs of periodontitis-induced maxillary sinus inflammation in some cases, as indicated by the yellow dotted line in (**c**), providing further evidence of its therapeutic effects on local inflammation. **b**, **d** In flat initial bone defects, CBCT scans also revealed therapeutic benefits of DPSC injection, although to a lesser extent compared with the narrow defect group. The yellow stars indicate the chief complaint tooth, and the yellow arrows indicate the bone defect

For the exploration of dental pulp stem cell (DPSC) injection treatment indications, the morphology of periodontal bone defects was also considered. As illustrated by a series of representative cases in Fig. 2, samples were divided into two groups based on the initial bone defect angle: a narrow intrabony defect group (<25°) and a flat intrabony defect group (>25°). The results suggest that DPSC injections were effective for both types of bone defects, but the therapeutic effect was more pronounced in the narrow intrabony defect group (Fig. 2). Furthermore, given that the outcomes of scaling and root planning (SRP) differ significantly between single-root and multiple-root teeth, we further categorized the treated teeth into a single-root group and a multiple-root group based on tooth position (Supplementary Table 16). The results showed that, overall, DPSC injection treatment was more effective in single-root teeth than in multiple-root teeth. However, this difference was not statistically significant, likely due to the small sample size. The observed outcome difference may be attributed to the complex anatomical structure of multiple-root teeth, including furcation involvement and other anatomical factors. To confirm this hypothesis, the multiple-root teeth were further divided into two subgroups: those with furcation involvement and those without (Supplementary Table 17). The analysis confirmed that DPSC injections were more effective in multiple-root teeth without furcation involvement than in those with furcation involvement. Additionally, the primary outcome—attachment loss (AL)—showed statistically significant improvement on day 180 in multiple-root teeth without furcation involvement, compared to their baseline levels (P = 0.0103). Therefore, these findings suggest that DPSC injections may be more effective for single-root teeth and multiple-root teeth without furcation involvement. This conclusion requires validation in future studies with a larger sample size.

Overall, dental pulp stem cell therapy demonstrated therapeutic efficacy in improving clinical outcomes in the treatment of periodontitis, enhancing both soft and hard tissue parameters—including attachment loss, periodontal probing depth, and bone defect depth—outcomes that have not been previously reported in the literature.

## Discussion

In this study, we developed an allogeneic human dental pulp stem cell injection for use in the treatment of periodontitis for the first time. Our clinical trials confirmed its significant efficacy in promoting periodontal regeneration, particularly in patients with stage III periodontitis. The pathogenesis of periodontitis is complex, making it essential to maintain a balance among various cellular functions within the local microenvironment—referred to as periodontal microenvironment homeostasis—for effective tissue repair. Stem cell therapy is considered a promising strategy for promoting periodontal regeneration, as transplanted stem cells not only serve as seed cells for tissue regeneration and differentiation but also enhance host cell function by secreting multiple cytokines, thereby restoring balance to the local molecular network.^11^

Our previous studies demonstrated that dental pulp stem cells possess immunomodulatory properties and interact with T cells, B cells, natural killer cells, monocytes, macrophages, dendritic cells, and neutrophils.^25–27^ We also previously showed that these cells exhibit low immunogenicity and exert immunosuppressive effects through PGE2-induced T-cell anergy,^28^ and regulate humoral immune responses by secreting interleukin-6, which enhances B-cell viability while inhibiting B-cell proliferation, differentiation, and migration.^20^ These findings alleviate concerns regarding post-implantation immune rejection. In our clinical trials, DPSC transplantation exhibited a safety profile consistent with our previous animal studies.^11,29^ Results from the double-injection group further confirmed the safety of repeated DPSC administration in vivo. These are essential prerequisites for the clinical application of stem cell therapy. Additional clinical studies involving allogeneic DPSC therapy have also demonstrated the safety of DPSC transplantation in humans. For example, Ye Qingsong et al. treated patients with severe COVID-19 using allogeneic human dental pulp stem cells and found that transplantation was generally safe and had a positive impact on clinical outcomes.^30^ Hernández-Monjaraz Beatriz et al. reported a case of periodontitis treated with allogeneic DPSCs and confirmed the safety and efficacy of the therapy.^31^ Moreover, a randomized, placebo-controlled multicenter trial (J-REPAIR) investigating the use of allogeneic human dental pulp stem cells for the treatment of acute ischemic stroke indicated that DPSC therapy was not associated with significant toxicity or major adverse events during the treatment period.^32^

**Fig. 3:**
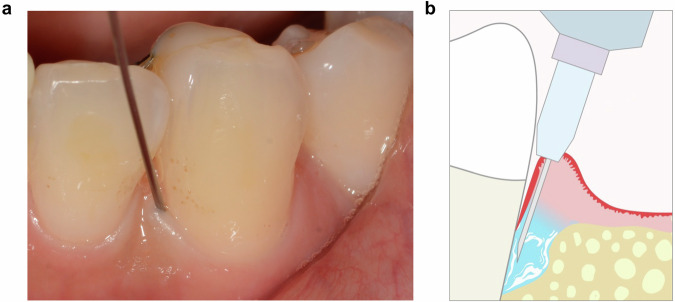
Standard operating procedure for clinical DPSC injection (**a**) Representative image of the injection site. **b** Schematic diagram illustrating the DPSC injection procedure

Several oral and maxillofacial mesenchymal stem cell types with high proliferative and differentiation capacities and low lipogenic potential have been identified. Multiple in vivo and in vitro studies have examined candidate stem cell sources for oral disease treatment, among which DPSCs are considered particularly promising due to their superior proliferative ability and enhanced resistance to subculture-induced and inflammation-induced senescence. The DPSCs used in this study were identified by the National Institutes for Food and Drug Control. The identification reports indicated that DPSCs exhibit low immunogenicity, strong osteogenic differentiation potential, and immunoregulatory capacity. Meanwhile, their chondrogenic differentiation potential was relatively weak (data not shown). Compared to other types of mesenchymal stem cells, such as bone marrow–derived stem cells or adipose tissue–derived stem cells, DPSCs offer several advantages. For example, DPSCs are easily obtained from extracted orthodontic or wisdom teeth, and the collection process does not involve invasive procedures.^33,34^ In addition, compared to other stem cell types, DPSCs proliferate more rapidly^33^ and can resist lipopolysaccharide-induced oxidative stress and cellular senescence in the periodontitis microenvironment, making them particularly effective for inducing periodontal regeneration.^17^ DPSC implantation has been shown to promote pulp regeneration and root development, proving effective in the treatment of tooth injuries caused by trauma and demonstrating the clinical safety and efficacy of this approach.^35^ Notably, the efficacy of allogeneic stem cell therapy for periodontitis observed in our clinical trials was comparable to the results of our previous in vivo studies in miniature swine, in which AL improved by 2.9 ± 0.90 mm with autologous stem cell therapy, compared to 1.67 ± 1.51 mm in the present DPSC clinical trial.^14^ However, in our prior animal experiments, stem cells were transplanted following surgical flap procedures. In contrast, the goal of clinical periodontitis therapy is to achieve effective regeneration through a minimally invasive, non-surgical approach. These results underscore the advantages of DPSC injection therapy over surgical transplantation techniques. In the context of injection-based drug delivery, cell retention efficiency remains an important issue warranting further investigation. In this study, all dentists performing DPSC injections were professionally trained. The injection sites were located in alveolar bone defects beneath the conjunctival epithelium. The needle was inserted until it reached the bone surface, and the injection was administered while withdrawing the needle, thereby ensuring that the entire bone defect was filled with cells (Fig. 3). Moreover, in our previous in vivo study,^11^ we demonstrated that injected human DPSCs (hDPSCs) remained within mandibular bone defects when delivered beneath the periosteum using iodinated contrast medium. computed tomography (CT) imaging confirmed the localization of the injected cells, and hDPSC administration significantly enhanced periodontal tissue regeneration in miniature swine. We also compared the effects of DPSC injection with those of an established non-invasive periodontal therapy using enamel matrix derivatives (EMDs), which are known to promote the regeneration of cementum, periodontal ligament, and alveolar bone in human periodontal defects. The outcomes were comparable, with attachment level gain of 1.67 ± 1.51 mm for DPSC injection and 3.9 ± 1.7 mm for EMD therapy.^36^ It is noteworthy that most follow-up assessments in studies involving enamel matrix derivatives were conducted over a period of 1 year, whereas the follow-up duration in our trial was 6 months. Therefore, additional cases and longer observation periods are needed to allow for a more accurate comparison between these clinical treatments. Nevertheless, compared with regenerative surgery using recombinant growth factors, stem cell therapy may bring economic concerns, as well as effort-related burdens. In addition, the immunogenicity risk of allogeneic stem cells is also worthy of in-depth exploration in the future.

Although the therapeutic effect did not become more pronounced with a double dose of DPSC administration, further investigation is needed to determine the optimal dosage and timing. Nonetheless, this study suggests that the level of inflammation may influence the required treatment dose. Specifically, a 1 × 10^7^ dose may be more appropriate for severe periodontitis, whereas lower doses could be more effective in mild to moderate cases. Patients with stage III periodontitis—typically candidates for periodontal surgery—may now benefit from a more minimally invasive option through DPSC injection, representing a significant advancement in clinical treatment technology for periodontitis. In addition, we observed that the therapeutic effect of DPSC injection on single-root teeth was greater than that observed in multiple-root teeth, likely due to the more complex anatomical constraints of multiple-root teeth, such as furcation involvement. Further analysis also confirmed that multiple-root teeth without furcation involvement exhibited more favorable outcomes following DPSC injection. Larger sample sizes are needed to validate these findings in future studies.

Overall, DPSC injection demonstrated significant advantages over traditional periodontal treatments, offering convenience, minimal invasiveness, improved patient comfort, superior clinical outcomes, and a reduced risk of adverse events such as external inflammatory root resorption. Given its lower technical complexity compared to maxillofacial tissue reconstruction surgeries, this approach is likely to be more time- and cost-effective, promoting alveolar bone regeneration through a minimally invasive injection strategy—a breakthrough in oral disease treatment technology.

This study has several limitations, in addition to the need for a longer observation period, a larger sample size is required in future studies to explore the optimal dosage of DPSC injection for each stage of periodontitis. Our results suggest that the optimal dose of DPSC injection may differ depending on the severity of periodontal inflammation, which limited the effectiveness of our statistical analysis and necessitated post hoc analyses combining data from both trials. Due to the final dataset constraints, the number of samples with AL < 5 mm included in the post hoc analysis was small. Although the final analysis and current expert consensus guidelines suggest that such patients may not require stem cell therapy, this conclusion remains open to challenge given the limited sample size. Further research is therefore needed to determine whether lower doses of DPSC injection are appropriate for mild to moderate periodontitis and to establish the optimal timing for a second injection.

In summary, this study demonstrated that dental pulp stem cell injection combined with scaling and root planning is an effective, non-surgical treatment for intraosseous periodontal defects in chronic periodontitis. The procedure is safe, straightforward, and minimally invasive, and it results in significant alveolar bone regeneration.

## Materials and methods

### Donors, trials, and patients

DPSCs are a type of mesenchymal stem cell.^37^ To obtain these cells, healthy teeth were collected from adult donors aged 18–35 years at the Dental Clinic of Beijing Stomatological Hospital, following written informed consent for participation in this study. DPSCs were extracted from fresh, caries-free, pulpitis-free, and non-necrotic third molars or from intact permanent teeth extracted for orthodontic purposes.^38^ All extracted teeth had intact roots. Donors with infections caused by hepatitis B virus, hepatitis C virus, human immunodeficiency virus, syphilis, cytomegalovirus, Epstein–Barr virus, or human T-lymphotropic virus were excluded from the final donor pool. (For detailed protocols on DPSC preparation, see the Supplementary Fig. 1 in Supplementary Materials. The donor informed consent forms can be found in the Supplementary Materials).

To evaluate the safety and efficacy of DPSC injection, we investigated whether this technique, when combined with standard periodontal therapy, could safely and effectively promote periodontal tissue regeneration in humans. Two randomized, placebo-controlled clinical trials were conducted at separate clinical centers using consistent protocols for medication, injection method (periodontal tissue injection), inclusion and exclusion criteria, and outcome assessments.

The first trial was an investigator-initiated, randomized controlled study designed to evaluate the safety and efficacy of single-dose DPSC therapy, conducted at Beijing Stomatological Hospital from May 22, 2020, to February 16, 2023 (Ethical code: CMUSH-IRB-KJ-PJ-2019-14). The second trial, conducted at Peking University Third Hospital (Ethical code: D2020188), was a Phase I clinical trial—a randomized, double-blind, controlled study aimed at assessing the safety and preliminary efficacy of DPSC therapy at varying dose concentrations, carried out from July 31, 2021, to December 24, 2022.

Patients aged 18–65 years with chronic periodontitis (probing pocket depth of 4–8 mm) were eligible to participate. However, individuals with unstable blood pressure; systemic diseases (e.g., cancer, diabetes, heart disease, recent myocardial infarction within 6 months, recent symptoms of angina pectoris, or congenital heart disease); systemic infections; prior surgical treatment near the affected tooth; or current smoking habits exceeding 10 cigarettes per day were excluded.^12^ (For further details, see Table 1).

### Randomization and procedures

In the initial treatment stage, periodontal infections in all patients were managed using oral hygiene instruction and mechanical debridement. When necessary, antiseptics were applied to disinfect the area.^39^ Mechanical debridement involved the removal of plaque, calculus, and damaged tissue. During debridement, the root surfaces and bone defects were carefully scaled to remove residual mineralized deposits while preserving the root cementum. Bone defects were assessed using a needle injection immediately after scaling and root planning. Following these initial treatments, 0.6 mL of DPSCs were injected at the root surface to overfill the defect prior to the start of the experimental procedure (Fig. 3).

A total of 132 participants were enrolled across both trials: 96 patients in the investigator-initiated trial and 36 patients in the Phase I trial. In both studies, either DPSCs (experimental group) or saline (control group) was injected directly into the local periodontal bone defects immediately after deep cleaning, including scaling and root planning. The control procedure was identical to the experimental procedure, except for the omission of DPSCs (Fig. 3). A flow chart outlining the step-by-step sampling design for both trials is provided in Fig. 1.

In the investigator-initiated trial, patients underwent two stages of randomization using the block randomization method, with random group codes generated by SAS software version 9.4. In the first stage, participants were randomly assigned in a 1:1 ratio to receive either DPSC injection or saline injection based on their assigned codes. In the second stage, sealed envelopes were used to further allocate participants into one of three groups: a single DPSC injection (1 × 10⁷ cells/injection), a double DPSC injection (1 × 10⁷ cells/injection, administered twice at a 30-day interval), or a saline injection. Allocation was conducted in a 1:1:1 ratio according to the randomization codes, resulting in 33 patients in the saline group, 33 in the single-injection group, and 30 in the double-injection group.

The investigator-initiated trial adopted a complete randomization method. Randomization tables and the corresponding groups of the randomization tables were generated using SAS software version 9.4. And randomization numbers were assigned using the Interactive Web Response System (IWRS). Each group was randomly assigned separately. The subjects in each group were randomly assigned to the experimental group or the control group at a ratio of 3:1. Each subject was assigned a random number from small to large according to the screening number on Day 1 and entered the corresponding group.

### Outcomes and statistical analyses

For the sample size calculation of Phase I trial, the objective was to assess safety; therefore, the sample size was not determined based on statistical assumptions and was not formally calculated. A total of 36 participants were included, and each participant was randomly assigned to a distinct dose group, receiving a single dose of DPSC injection. The sample sizes for each group were as follows: nine individuals received no cells for one tooth (saline injection group); three individuals received 1 × 10^6^ cells for one tooth; six individuals received 5 × 10^6^ cells for one tooth; six individuals received 1 × 10^7^ cells for one tooth; six individuals received 2 × 10^7^ cells for two teeth; and six individuals received 3–4 × 10^7^ cells for three or four teeth. In each dose group, controls were randomly selected. Four participants were enrolled in the 1 × 10^6^ cells/one tooth group and randomly assigned to the experimental group (three cases) or the saline injection group (one case), while for the remaining four dose groups, eight participants were enrolled in each group and randomly assigned to the experimental group (six cases) or the saline injection group (two cases).

For the investigator-initiated trial, the unit of statistical analysis was the tooth. Based on historical data, attachment loss improved by 2.0 mm 6 months after surgery alone, while improvement reached 3.4 mm following DPSC injection, with a standard deviation of 1.7 mm. The single- and double-injection DPSC groups were compared with the saline group using a significance level of α = 0.025. Assuming a 1:1:1 group allocation ratio, a sample size of 29 teeth per group was required to achieve 80% power to detect between-group differences. Accounting for a 10% loss to follow-up, 32 teeth per group were needed.^12,40^

Clinical safety and efficacy evaluations were conducted over a 6-month follow-up period (180 ± 14 days).^41^ All adverse events were documented. Visit details are provided in Supplementary Table 2. Following treatment, all participants were monitored in the hospital for 24 h to assess clinical safety prior to discharge. The primary safety outcome was the occurrence of any serious adverse events.

**Fig. 4:**
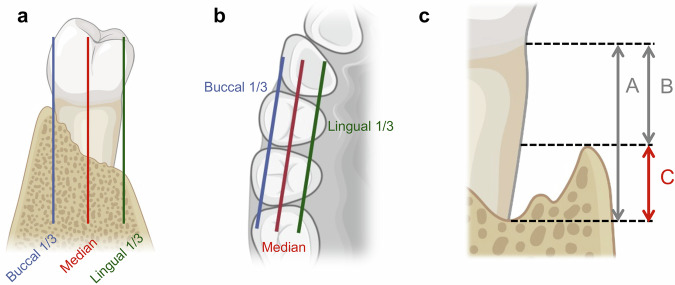
Schematic illustration of the BDD measurement based on CBCT results. **a**, **b** The analysis of each target tooth was accomplished based on a comprehensive assessment in three layers, including the buccal 1/3, median, and lingual 1/3 layer. **c** In each layer, the BDD (**C**) was calculated according to the formula: **C** = **A** – **B**. **A** represents the distance from the cementoenamel junction (**CEJ**) to the lowest point of alveolar bone defect; **B** represents the distance from the **CEJ** to the vertex of the alveolar crest. The final analyzed BDD of this target tooth was the average of the measured values in three layers (buccal 1/3, median, and lingual 1/3 layer). Figure created with BioRender.com. CEJ cementoenamel junction

To evaluate the efficacy of DPSC injection, both soft and hard tissue prognostic indicators of periodontitis were assessed at each follow-up visit through clinical examination.^42^ The primary efficacy outcome was the level of tooth AL at 6 months post-treatment. Secondary efficacy outcomes included periodontal probing depth (PD), gingival recession (GR), tooth mobility (TM), bleeding on probing (BOP), and bone defect depth (BDD) at 6 months post-treatment. BDD was evaluated based on cone-beam computed tomography (CBCT) imaging, and the specific measurement method is illustrated in Fig. 4. Briefly, the bone defect depth of the target tooth was measured in the CBCT data. The observation line was adjusted in the axial position to be consistent with the mesial-distal direction of the tooth. The observation line was adjusted in the oblique sagittal position to be consistent with the long axis direction of the tooth. Then the key data were measured respectively at the buccal 1/3, median and lingual 1/3 layer of the target tooth, including A: The distance from the cementoenamel junction (CEJ) to the lowest point of alveolar bone defect, B: The distance from the CEJ to the vertex of the alveolar crest. The BDD (C) in this layer was calculated according to such a formula: C = A − B. And the average values measured in all layers indicated the BDD of this tooth.

For the separate analysis of results from the investigator-initiated study and the Phase I trial, the database was locked and loaded into SAS version 9.4 (Statistical Analysis Software; SAS Institute, Cary, NC, USA). All statistical tests were conducted on a two-sided basis (unless otherwise specified), and a P value of less than 0.05 was considered statistically significant.^39^ Statistical significance (P < 0.05) was determined by Student’s t-test.

In addition to the separate analyses, a combined analysis was conducted to assess the response to DPSC injection in patients with varying degrees of periodontitis, as well as to evaluate the efficacy of the 1 × 10^7^ dose of DPSC injection. The combined treatment group consisted of teeth that received the same injection dose (1 × 10^7^ cells/0.6 mL) in both trials: 33 teeth from the investigator-initiated study and 18 teeth from the Phase I trial. Similarly, data from the saline injection groups in both trials were pooled, comprising 29 teeth from the investigator-initiated study and 34 teeth from the Phase I trial. Based on baseline AL levels, patients were categorized into two groups: stage II periodontitis (AL < 5 mm) and stage III periodontitis (AL ≥ 5 mm). In addition to AL, all other efficacy outcomes were compared between these two periodontitis stages. The combined dataset was analyzed using SAS version 9.4, employing the same statistical methods as those used in the separate analyses. Statistical significance (P < 0.05) was determined by Student’s t-test.

Similarly, a combined analysis was conducted to assess the response to DPSC injection in teeth with single root or multiple roots. According to the position of the treated teeth, the anterior teeth and anterior molars are classified as single-root teeth, while the molars are classified as multiple-root teeth. Multiple-root teeth were further divided into two subgroups: those with furcation involvement and those without, based on their clinical examination results before the treatments. All efficacy outcomes were assessed on the day 180 post-treatment, and the combined dataset was analyzed using SAS version 9.4, employing the same statistical methods as those used in the separate analyses. Statistical significance (P < 0.05) was determined by Student’s t-test.

## Supplementary information

Supplementary Materials

Supplementary File 1

Supplementary File 2

Supplementary File 3

Supplementary File 4

Supplementary File 5

CONSORT Checklist

## Data Availability

All data are available in the main text or the Supplementary Materials.
