# Supplementary material for: Impact of allogeneic dental pulp stem cell injection on tissue regeneration in periodontitis: a multicenter randomized clinical trial
Source: Signal Transduct Target Ther. 2025 Jul 31;10:239. doi: 10.1038/s41392-025-02320-w (PMC12311062; doi:10.1038/s41392-025-02320-w)
Supplement: Supplementary file 1 — Supplementary Materials [file 41392_2025_2320_MOESM1_ESM.docx]

Supplementary Materials for

Impact of allogeneic dental pulp stem cell injection on tissue regeneration in periodontitis: a multicenter randomized clinical trial

Yi Liu^1,2,3†^*, Yitong Liu^1†^, Jingchao Hu^1†^, Jianhui Han^4^, Lin Song^1^, Xu Liu^1^, Nannan Han^1^, Xia Xia^5†^, Jia He^5^, Hongfang Meng^5^, Meng Wan^6†^, Haojie Wang^6^, Xiaodan Liu^6^, Zhanyi Gao^6^, Xiao Wang^6^*, Chutse Wu^5^*, Songlin Wang^2,3,7,8,9^*

Correspondence to: lililiuyi@163.com (Y, Liu), bysywangxiao@163.com (X, Wang), 13910026365@163.com (C, Wu), slwang@ccmu.edu.cn (S. Wang).

† These authors contributed equally to this work.

**This PDF file includes:**

Materials and Methods

Supplementary Figure 1-2

Supplementary Table 1-17

**Other Supplementary Materials for this manuscript include the following:**

| **Supplementary File** | **File title** |
| --- | --- |
| Supplementary File 1 | Study protocol for IIT |
| Supplementary File 2 | Study protocol for phase I trial |
| Supplementary File 3 | Statistical analysis plan for IIT |
| Supplementary File 4 | Statistical analysis plan for phase I trial |
| Supplementary File 5 | Statistical analysis plan for combined dataset |

Materials and Methods

**Cell banking and quality control**

Primary cells were digested with 0.05 % trypsin (Gibco, NYC, USA) and passaged in serum-free medium every 4 days. P3, P5, and P6 hDPSCs were stored in a Primary Cell Bank (P3), Working Cell Bank (P5), and Stock Solution (P6), respectively. Quality control evaluations were performed before cell banking, and included cell number, viability, morphology, sterility, endotoxin, mycoplasma, short tandem repeat profiling, karyotyping, immunophenotyping, multilineage differentiation, intrinsic and extrinsic factor analysis, and biological effectiveness analysis. The details of cell banking and quality control are summarized in Fig. S1.

**Quality control and release of preparations**

Cells were derived from the Stock Solution (P6). Briefly, human DPSCs (hDPSCs) at P6 were recovered, filtered, and resuspended in normal saline at a density of 1.66 × 10^7^ cells/mL. Residues including bovine serum albumin, trypsin, collagenase I, and dispase II were strictly controlled. Cell viability, sterility, and endotoxin concentrations were tested and met quality standards before the preparation was released to the clinic.

**Clinical study design**

The study conducted at the Beijing Stomatological Hospital was a randomized, controlled, open-label, parallel-group study (IIT), initiated in June 2018. The first participant was enrolled on May 22, 2020, and the final participant completed the day 180 visit on February 16, 2023.

The trial conducted at the Peking University Third Hospital was a dose-increasing, randomized, double-blind phase I clinical trial. The first participant was enrolled on July 31, 2021, and the final participant completed the day 180 visit on December 24, 2022. Both clinical studies were conducted in accordance with the principles of the Declaration of Helsinki, and the protocol was approved by the institutional ethics committees.

Inclusion criteria were as follows: patients aged 18–65 years and of any gender; willing and able to provide written consent to participating in a research study; diagnosed with periodontal defects and a cuneiform bone defect, confirmed by radiological studies; PD of 4–8mm.

Exclusion criteria were as follows: systolic blood pressure ≥ 180 mm Hg or diastolic blood pressure ≥ 110 mm Hg; systemic diseases (cancer, diabetes, heart disease, myocardial infarction within 6 months, symptoms of angina pectoris developed within 6 months, and congenital heart disease, among others); use of nonsteroidal anti-inflammatory drugs, steroid hormones within 3 months before treatment, and/or long-term use of hormones or bisphosphonates; systemic infections, including HIV or hepatitis B; previous surgical treatments near the studied tooth; known allergies to any of the materials used in the study; alveolar bone resorption exceeding 2/3 of tooth root length; severe hepatic and renal insufficiency; any bleeding tendencies or coagulation dysfunction (white blood cell, WBC < 3.0×10^9^/L or platelet count< 60×10^9^/L); recent engagement in unprotected sexual behavior; pregnancy, lactation, or use of estrogen-based contraception; intention to become pregnant during the study period or within 6 months thereafter; current smoking (> 10 cigarettes/day); projected survival of less than 12 months; other factors, at the discretion of the principal investigator.

Supplementary Figures


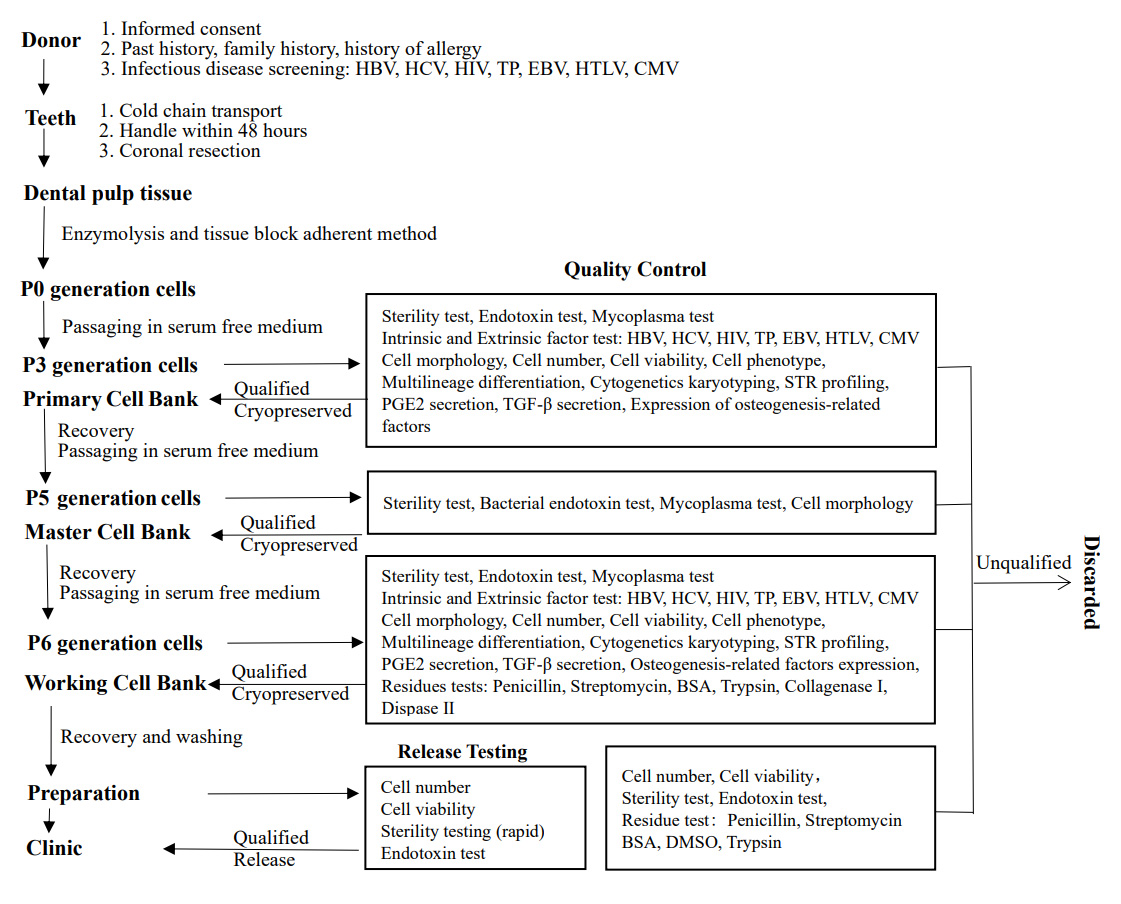


Supplementary Fig 1. Preparation production and quality control


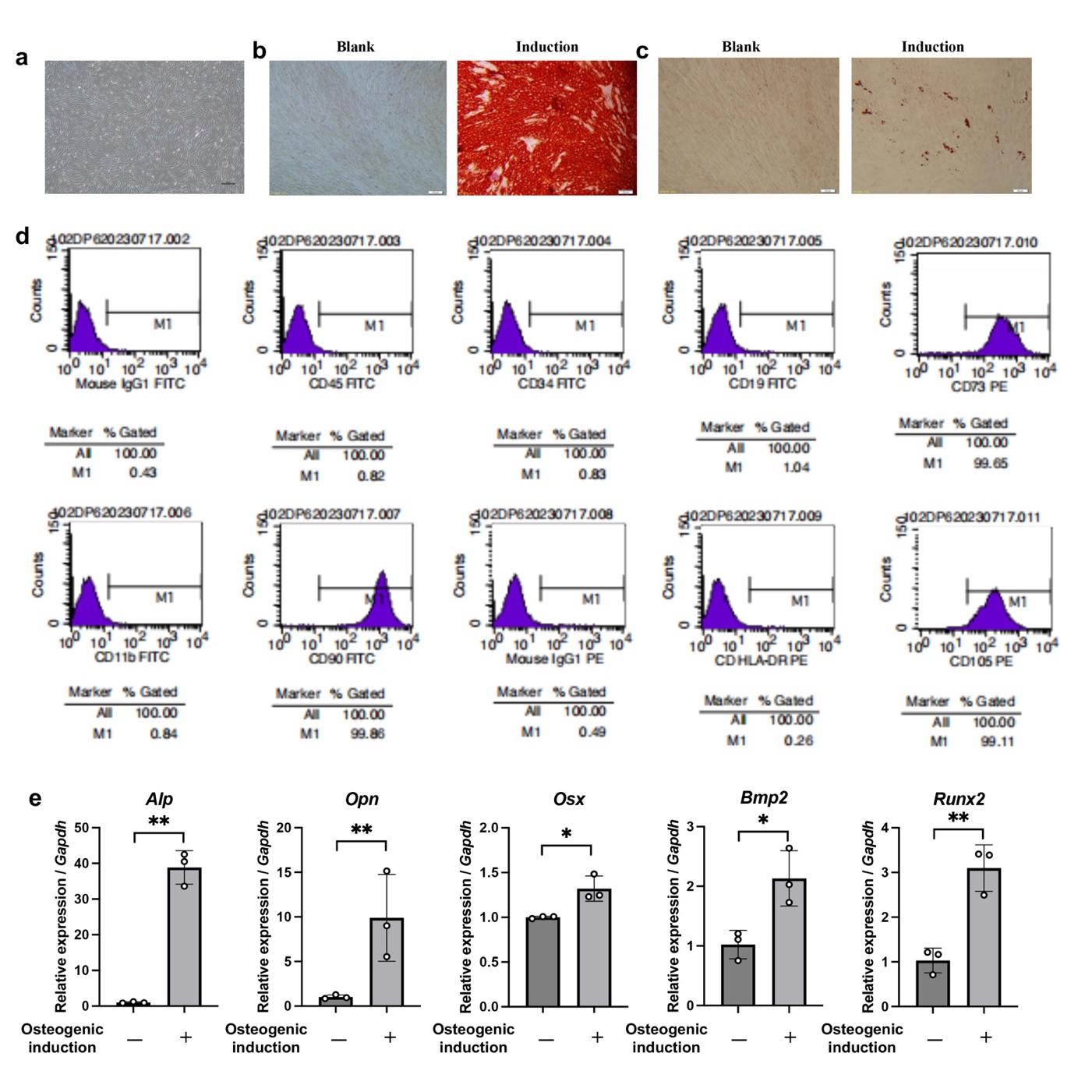


Supplementary Fig 2. The *in vitro* results of DPSCs

(a) Banked cells exhibited a spindle shape and spiral arrangement in culture flasks. (b) Alizarin red staining result. (c) Oil red O staining result. (d) Flow cytometry analysis of DPSC surface markers. (e) Real-time PCR analysis of osteogenic differentiation-related genes during osteogenic induction of DPSCs.

All results are representative of at least three independent experiments. Scale bar in (a-c) = 100 μm. Data are presented as mean±SD. *P<0.05. **P<0.01.

Supplementary Tables

Supplementary Table 1. The visit details for IIT study

|  | Applications | | Screening and treatment period | | Study and follow-up period | | | |
| --- | --- | --- | --- | --- | --- | --- | --- | --- |
|  |  |  | Screening | Treatment | 1^st^ follow-up | 2^nd^ follow-up | 3^rd^ follow-up | 4^th^ follow-up |
|  |  |  | (D-14~0) | (D1) | (D7±1, on phone) | (D30±3) | (D90±3) | (D180±5) |
| 1 | Sign ICF | | x |  |  |  |  |  |
| 2 | Inclusion /exclusion criteria | | x | x |  |  |  |  |
| 3 | Demographic information | | x |  |  |  |  |  |
| 4 | Medical history information | | x |  |  |  |  |  |
| 5 | Vital signs: respiration, heart rate, blood pressure, body temperature, clinical assessment of general condition | | x | x (Before and 2h±30min after the injection) |  | x (Before and 2h±30min after the 2^nd^ injection) |  |  |
| 6 | Periodontal clinical indicators: AL, PD, BOP, GR, TM | | x | x |  |  | x | x |
| 7 | Laboratory examination | Blood routine examination | x | x |  | x | x | x |
|  |  | Blood coagulation function | x | x |  |  |  |  |
|  |  | Liver and kidney function | x | x |  | x | x | x |
|  |  | Hypersensitive C-reactive protein | x | x (Before and 2h±30min after the injection) |  | x (Before and 2h±30min after the 2^nd^ injection) |  |  |
|  |  | Infectious disease detection: HBsAg, HBsAb, HBeAg, HBeAb, HBcAb, Anti-HCV, HIVcombin, Anti-TP, CMV-IgM, CMV-IgG | x |  |  | x | x | x |
|  |  | Immunological detection: IgA, IgG, IgM, total IgE | x | x (Before and 2h±30min after the injection) |  | x (Before and 2h±30min after the 2^nd^ injection) | x | x |
|  |  | Pregnancy examination (blood β-HCG) | x | x |  | x | x | x |
|  |  | Urine routine examination | x | x |  |  |  |  |
| 8 | Primary periodontal therapy （SRP） | | x (Complete oral cleansing on D-14, half oral SRP on D-7±1) | x (Half oral SRP) |  |  |  |  |
| 9 | DPSCs injection application | |  | x |  | x (Only for the double DPSCs injection group) |  |  |
| 10 | TEAE records | | x | x | x | x (Before and 2h±30min after the 2^nd^ injection) | x | x |
| 11 | Records of combined medication or treatment | | x | x | x | x | x | x |
| 12 | CBCT examination | | x |  |  |  | x | x |
| 13 | Intraoral photography | | x | x |  | x | x | x |

Supplementary Table 2. The visit details for phase I trial

|  | Applications | | Screening and treatment period | | | |  | Study and follow-up period | | | |  |
| --- | --- | --- | --- | --- | --- | --- | --- | --- | --- | --- | --- | --- |
|  |  |  | Screening | Primary periodontal treatment of the opposite half side of the studied tooth | Assign random numbers | Treatment | Observation | 1^st^ follow-up | 2^nd^ follow-up | 3^rd^ follow-up | 4^th^ follow-up | 5^th^ follow-up |
|  |  |  | (D-28~D-9) | (D-8±2) | (D-1) | (D1) | (D2) | (D7±1) | (D14±1) | (D30±3) | (D90±7) | (D180±14) |
| 1 | Sign ICF | | x |  |  |  |  |  |  |  |  |  |
| 2 | Inclusion /exclusion criteria | | x | x | x |  |  |  |  |  |  |  |
| 3 | Demographic information | | x |  |  |  |  |  |  |  |  |  |
| 4 | Medical history information | | x |  |  |  |  |  |  |  |  |  |
| 5 | Vital signs: respiration, heart rate, blood pressure, body temperature, clinical assessment of general condition | | x |  |  | x | x | x | x | x | x | x |
| 6 | Periodontal clinical indicators: AL, PD, BOP, GR, TM | |  | x |  |  |  |  |  |  | x | x |
| 7 | Laboratory examination | Blood routine examination | x |  |  | x | x | x | x | x | x | x |
|  |  | Blood coagulation function | x |  |  | x |  |  |  |  |  |  |
|  |  | Liver and kidney function | x |  |  | x | x | x | x | x | x | x |
|  |  | Hypersensitive C-reactive protein | x |  |  | x |  |  |  |  |  |  |
|  |  | Infectious disease detection: HBsAg, HBsAb, HBeAg, HBeAb, HBcAb, Anti-HCV, HIVcombin, Anti-TP, CMV-IgM, CMV-IgG | x |  |  |  |  |  |  | x | x | x |
|  |  | Immunological detection: IgA, IgG, IgM, total IgE | x |  |  | x | x | x | x | x | x |  |
|  |  | Pregnancy examination (blood β-HCG) | x |  | x |  |  |  |  | x | x | x |
|  |  | Urine routine examination | x |  |  | x | x |  |  |  |  |  |
|  |  | Stool routine examination | x |  |  |  | x |  |  |  |  |  |
|  | Random inclusion | | x |  | x |  |  |  |  |  |  |  |
| 8 | Primary periodontal therapy （Complete oral cleansing） | | x |  |  |  |  |  |  |  |  |  |
| 9 | Primary periodontal therapy （SRP） | |  | x |  | x |  |  |  |  |  |  |
| 10 | DPSCs injection application | |  |  |  | x |  |  |  |  |  |  |
| 11 | TEAE records | |  | x |  | x | x | x | x | x | x | x |
| 12 | Records of combined medication or treatment | | x |  |  | x | x | x | x | x | x | x |
| 13 | CBCT examination | | x |  |  |  |  |  |  |  | x | x |
| 14 | Electrocardiogram examination | | x |  | x | x | x | x | x | x | x | x |

Supplementary Table 3. Summary of banked P6 DPSC results

| Test item | Results |
| --- | --- |
| Cell characteristics | Well-adhesion |
| Cell morphology | Long fusiform spiral arrangement |
| Bacterial detection | Negative |
| Mycoplasma | Negative |
| Bacterial endotoxin content | <5EU/ml |
| Exogenous virus detection | Negative |

Supplementary Table 4. Preparation residue and cell count, viability detection

|  | Test item | Results | | |
| --- | --- | --- | --- | --- |
| 1 | Recombinant pancreatic enzyme  ng/dose | 0.1 | 0.1 | 0.1 |
| 2 | Collagenase  ng/dose | ----- | ----- | ----- |
| 3 | Dispersing enzyme  nmol/mL | 0.472 | | |
| 4 | BSA  ng/dose | 0.4 | 0.5 | 0.5 |
| 5 | Cell number | 0.96x10^7 | 1.03x10^7 | 0.95x10^7 |
| 6 | Cell viability | 91.62% | 93.87% | 92.92% |

Supplementary Table 5. Characteristics of participants in the phase I trial (FAS)

|  |  | 1×10^6^ DPSCs /1 tooth | 5×10^6^ DPSCs /1 tooth | 1×10^7^DPSCs  /1 tooth | 2×10^7^ DPSCs  /2 teeth | 3-4×10^7^ DPSCs  /3-4 teeth | Saline injection | Total |
| --- | --- | --- | --- | --- | --- | --- | --- | --- |
|  |  | N=3 | N=6 | N=6 | N=6 | N=6 | N=9 | N=36 |
| Age (year) | Number of cases (missing count) | 3(0) | 6(0) | 6(0) | 6(0) | 6(0) | 9(0) | 36(0) |
|  | Mean (SD) | 43.7(3.79) | 41.8(5.64) | 37.3(11.08) | 44.0(6.42) | 38.0(10.97) | 34.8(7.90) | 39.2(8.54) |
|  | Median | 42.0 | 42.5 | 38.5 | 44.5 | 35.5 | 38.0 | 41.5 |
|  | Quartile | 41.0, 48.0 | 38.0, 47.0 | 25.0, 47.0 | 42.0, 48.0 | 34.0, 49.0 | 29.0, 42.0 | 33.5, 46.0 |
|  | Minimum, maximum | 41, 48 | 33, 48 | 24, 51 | 33, 52 | 22, 52 | 21, 43 | 21, 52 |
|  |  |  |  |  |  |  |  |  |
| Sex | Male | 1 (33.3) | 3 (50.0) | 2 (33.3) | 4 (66.7) | 5 (83.3) | 3 (33.3) | 18 (50.0) |
|  | Female | 2 (66.7) | 3 (50.0) | 4 (66.7) | 2 (33.3) | 1 (16.7) | 6 (66.7) | 18 (50.0) |
|  |  |  |  |  |  |  |  |  |
| Nationality | Han | 3 (100.0) | 6 (100.0) | 5 (83.3) | 6 (100.0) | 6 (100.0) | 9 (100.0) | 35 (97.2) |
|  | Others | 0 (0.0) | 0 (0.0) | 1 (16.7) | 0 (0.0) | 0 (0.0) | 0 (0.0) | 1 (2.8) |
|  |  |  |  |  |  |  |  |  |
| Weight (kg) | Number of cases (missing count) | 3(0) | 6(0) | 6(0) | 6(0) | 6(0) | 9(0) | 36(0) |
|  | Mean (SD) | 58.83(14.003) | 68.83(13.408) | 69.17(11.286) | 73.83(14.716) | 68.75(13.971) | 66.06(7.393) | 68.18(11.867) |
|  | Median | 51.00 | 66.50 | 67.00 | 71.50 | 69.00 | 65.00 | 69.00 |
|  | Quartile | 50.50, 75.00 | 57.00, 79.00 | 59.00, 80.00 | 69.00, 90.00 | 59.50, 80.00 | 62.00, 73.00 | 59.00, 76.00 |
|  | Minimum, maximum | 50.5, 75.0 | 56.0, 88.0 | 57.0, 85.0 | 51.0, 90.0 | 48.0, 87.0 | 54.0, 77.0 | 48.0, 90.0 |
|  |  |  |  |  |  |  |  |  |
| Height (cm) | Number of cases (missing count) | 3(0) | 6(0) | 6(0) | 6(0) | 6(0) | 9(0) | 36(0) |
|  | Mean (SD) | 161.50(7.467) | 169.37(10.957) | 167.50(5.320) | 169.08(6.711) | 173.00(7.403) | 167.61(7.873) | 168.52(7.813) |
|  | Median | 158.50 | 167.00 | 169.50 | 166.50 | 173.50 | 165.00 | 169.50 |
|  | Quartile | 156.00, 170.00 | 161.00, 176.50 | 165.00, 170.00 | 165.00, 172.50 | 170.00, 177.00 | 160.00, 174.00 | 161.50, 173.50 |
|  | Minimum, maximum | 156.0, 170.0 | 158.2, 186.5 | 158.0, 173.0 | 163.0, 181.0 | 161.0, 183.0 | 159.0, 181.5 | 156.0, 186.5 |
|  |  |  |  |  |  |  |  |  |

Supplementary Table 6. Characteristics of participants in the IIT study (FAS)

|  |  | Single DPSCs injection | Double DPSCs injection | Saline injection | Total |
| --- | --- | --- | --- | --- | --- |
|  |  | N=32 | N=30 | N=31 | N=93 |
| Age (year) | Number of cases (missing count) | 32 (0) | 30 (0) | 31 (0) | 93 (0) |
|  | Mean (SD) | 37.2 (8.15) | 37.5 (9.02) | 36.8 (8.19) | 37.2 (8.36) |
|  | Median | 36.5 | 37.5 | 35.0 | 36.0 |
|  | Quartile | 31.5, 43.5 | 29.0, 42.0 | 31.0, 43.0 | 31.0, 42.0 |
|  | Minimum, maximum | 22, 59 | 22, 56 | 23, 54 | 22, 59 |
|  |  |  |  |  |  |
| Sex | Male | 12 (37.5) | 14 (46.7) | 10 (32.3) | 36 (38.7) |
|  | Female | 20 (62.5) | 16 (53.3) | 21 (67.7) | 57 (61.3) |
|  | Total | 32 (100.0) | 30 (100.0) | 31 (100.0) | 93 (100.0) |
|  |  |  |  |  |  |
| Nationality | Han | 29 (90.6) | 29 (96.7) | 23 (74.2) | 81 (87.1) |
|  | Others | 3 (9.4) | 1 (3.3) | 8 (25.8) | 12 (12.9) |
|  | Total | 32 (100.0) | 30 (100.0) | 31 (100.0) | 93 (100.0) |
|  |  |  |  |  |  |
| Height (cm) | Number of cases (missing count) | 32 (0) | 30 (0) | 31 (0) | 93 (0) |
|  | Mean (SD) | 167.13 (7.495) | 169.00 (8.275) | 166.25 (7.009) | 167.44 (7.605) |
|  | Median | 164.00 | 168.00 | 166.00 | 166.00 |
|  | Quartile | 162.00, 172.00 | 163.00, 173.00 | 162.00, 171.00 | 162.00, 172.00 |
|  | Minimum, maximum | 158.0, 189.0 | 158.0, 192.0 | 152.0, 183.0 | 152.0, 192.0 |
|  |  |  |  |  |  |
| Weight (kg) | Number of cases (missing count) | 32 (0) | 30 (0) | 31 (0) | 93 (0) |
|  | Mean (SD) | 66.90 (16.260) | 67.10 (12.237) | 61.08 (9.861) | 65.02 (13.261) |
|  | Median | 65.00 | 65.00 | 58.00 | 62.00 |
|  | Quartile | 55.00, 75.00 | 58.00, 75.00 | 54.00, 65.00 | 55.00, 70.00 |
|  | Minimum, maximum | 45.0, 120.0 | 46.0, 92.0 | 50.0, 86.0 | 45.0, 120.0 |
|  |  |  |  |  |  |

Supplementary Table 7. Periodontal characteristics at baseline for IIT study (FAS)

|  |  | Single DPSCs injection | Double DPSCs injection | Saline injection |
| --- | --- | --- | --- | --- |
|  |  | n=32 | n=30 | n=31 |
| AL | Mean (SD) | 6.3 (1.69) | 6.3 (1.90) | 6.2 (1.61) |
|  | Median | 6.3 | 6.3 | 6.5 |
|  | Quartile | 5.3, 7.8 | 5.0, 8.0 | 5.0, 7.5 |
|  | Minimum, maximum | 3, 9 | 3, 10 | 4, 11 |
|  | P value (single DPSCs injection vs saline injection) | 0.4594 |  |  |
|  | P value (double DPSCs injection vs saline injection) |  | 0.7884 |  |
|  | P value (single DPSCs injection vs double DPSCs injection) |  | 0.7396 |  |
| PD | Mean (SD) | 5.7 (1.46) | 5.6 (1.77) | 5.7 (1.33) |
|  | Median | 6.0 | 5.0 | 5.5 |
|  | Quartile | 4.5, 7.0 | 4.0, 7.0 | 5.0, 7.0 |
|  | Minimum, maximum | 3, 8 | 2, 9 | 4, 8 |
|  | P value (single DPSCs injection vs saline injection) | 0.9724 |  |  |
|  | P value (double DPSCs injection vs saline injection) |  | 0.5959 |  |
|  | P value (single DPSCs injection vs double DPSCs injection) |  | 0.5907 |  |
| TM | (—) | 21 (65.6) | 23 (76.7) | 26 (83.9) |
|  | I° | 7 (21.9) | 2 (6.7) | 3 (9.7) |
|  | II° | 4 (12.5) | 5 (16.7) | 2 (6.5) |
|  | P value (single DPSCs injection vs saline injection) | 0.1005 |  |  |
|  | P value (double DPSCs injection vs saline injection) |  | 0.5668 |  |
|  | P value (single DPSCs injection vs double DPSCs injection) |  | 0.2440 |  |
| BDD | Mean (SD) | 2.61 (1.472) | 2.45 (1.314) | 2.28 (1.690) |
|  | Median | 2.40 | 2.25 | 1.80 |
|  | Quartile | 1.40, 3.40 | 1.50, 3.30 | 0.90, 3.00 |
|  | Minimum, maximum | 0.7, 7.7 | 0.4, 5.8 | 0.4, 7.7 |
|  | P value (single DPSCs injection vs saline injection) | 0.2248 |  |  |
|  | P value (double DPSCs injection vs saline injection) |  | 0.3870 |  |
|  | P value (single DPSCs injection vs double DPSCs injection) |  | 0.7507 |  |

Supplementary Table 8. Periodontal characteristics at baseline for the phase I trail (FAS)

|  |  | 1×10^6^ DPSCs /1 tooth | 5×10^6^ DPSCs /1 tooth | 1×10^7^DPSCs  /1 tooth | 2×10^7^ DPSCs  /2 teeth | 3-4×10^7^ DPSCs  /3-4 teeth | Saline injection |
| --- | --- | --- | --- | --- | --- | --- | --- |
|  |  | n=3 | n=6 | n=6 | n=12 | n=19 | n=16 |
| AL | Mean (SD) | 5.17 (0.289) | 5.25 (1.541) | 5.92 (2.131) | 5.13 (0.932) | 5.53 (1.476) | 5.25 (1.080) |
|  | Median | 5.00 | 4.75 | 5.75 | 5.50 | 5.50 | 5.00 |
|  | Quartile | 5.00, 5.50 | 4.00, 6.00 | 4.50, 7.50 | 4.25, 5.75 | 4.00, 6.50 | 4.50, 5.75 |
|  | Minimum, maximum | 5.0, 5.5 | 4.0, 8.0 | 3.0, 9.0 | 3.5, 6.5 | 3.5, 8.5 | 4.0, 7.5 |
|  | P value among groups | 0.861 |  |  |  |  |  |
| PD | Mean (SD) | 4.83 (0.764) | 4.42 (0.801) | 4.92 (1.201) | 4.79 (1.010) | 5.47 (1.504) | 5.16 (1.076) |
|  | Median | 5.00 | 4.00 | 5.00 | 4.75 | 5.00 | 5.00 |
|  | Quartile | 4.00, 5.50 | 4.00, 4.50 | 4.50, 5.50 | 4.00, 5.50 | 4.00, 6.50 | 4.50, 5.25 |
|  | Minimum, maximum | 4.0, 5.5 | 4.0, 6.0 | 3.0, 6.5 | 3.5, 6.5 | 3.5, 8.5 | 4.0, 7.5 |
|  | P value among groups | 0.443 |  |  |  |  |  |
| TM | (—) | 3 | 5 | 4 | 4 | 5 | 9 |
|  | I° | 0 | 1 | 2 | 3 | 1 | 2 |
|  | II° | 0 | 0 | 0 | 0 | 0 | 0 |
|  | P value among groups | 0.366 |  |  |  |  |  |
| BDD | Mean (SD) | 1.43 (1.501) | 1.62 (1.511) | 2.33 (1.528) | 2.02 (0.826) | 2.10 (1.186) | 1.64 (0.745) |
|  | Median | 1.50 | 1.20 | 2.20 | 1.70 | 2.00 | 1.60 |
|  | Quartile | -0.10, 2.90 | 0.60, 1.50 | 0.80, 4.00 | 1.50, 2.90 | 1.10, 2.90 | 1.25, 1.80 |
|  | Minimum, maximum | -0.1, 2.9 | 0.6, 4.6 | 0.7, 4.1 | 0.8, 3.3 | 0.6, 4.4 | 0.5, 3.9 |
|  | P value among groups | 0.644 |  |  |  |  |  |

Supplementary Table 9. IIT study-summary of TEAE (SS)

|  | Single DPSCs injection (N=30) | Double DPSCs injection  (N=30) | Saline injection  (N=33) | Total(N=96) |
| --- | --- | --- | --- | --- |
| Classification by system organ, Preferred Term | Examples (%) | Examples (%) | Examples (%) | Examples(%) |
| At least 1 adverse event occurred | 17 (51.5) | 7 (23.3) | 12 (36.4) | 36 (37.5) |
| Infections and infectious diseases | 2 (6.1) | 1 (3.3) | 2 (6.1) | 5 (5.2) |
| Nasopharyngitis | 1 (3.0) | 0 (0.0) | 1 (3.0) | 2 (2.1) |
| Rhinitis | 0 (0.0) | 1 (3.3) | 0 (0.0) | 1 (1.0) |
| Urinary tract infection | 0 (0.0) | 0 (0.0) | 1 (3.0) | 1 (1.0) |
| Gingival abscess | 1 (3.0) | 0 (0.0) | 0 (0.0) | 1 (1.0) |
| Various clinical examinations | 7 (21.2) | 5 (16.7) | 3 (9.1) | 15 (15.6) |
| Urinary white blood cell (+) | 4 (12.1) | 5 (16.7) | 2 (6.1) | 11 (11.5) |
| Urinary red blood cell (+) | 4 (12.1) | 2 (6.7) | 1 (3.0) | 7 (7.3) |
| Various surgeries and medical procedures | 0 (0.0) | 0 (0.0) | 1 (3.0) | 1 (1.0) |
| Periodontal scaling | 0 (0.0) | 0 (0.0) | 1 (3.0) | 1 (1.0) |
| Systemic disease and various reactions at the site of administration | 8 (24.2) | 1 (3.3) | 1 (3.0) | 10 (10.4) |
| Influenza-like illness | 3 (9.1) | 0 (0.0) | 0 (0.0) | 3 (3.1) |
| Injection site hemorrhage | 1 (3.0) | 0 (0.0) | 0 (0.0) | 1 (1.0) |
| Injection site pain | 1 (3.0) | 0 (0.0) | 0 (0.0) | 1 (1.0) |
| Injection site swelling | 3 (9.1) | 1 (3.3) | 1 (3.0) | 5 (5.2) |
| Reproductive system and breast diseases | 1 (3.0) | 0 (0.0) | 0 (0.0) | 1 (1.0) |
| Cervical ectropion | 1 (3.0) | 0 (0.0) | 0 (0.0) | 1 (1.0) |
| Gastrointestinal system diseases | 7 (21.2) | 0 (0.0) | 6 (18.2) | 13 (13.5) |
| Gingival bleeding | 0 (0.0) | 0 (0.0) | 1 (3.0) | 1 (1.0) |
| oral ulcer | 0 (0.0) | 0 (0.0) | 2 (6.1) | 2 (2.1) |
| Tooth sensitivity | 2 (6.1) | 0 (0.0) | 0 (0.0) | 2 (2.1) |
| Tooth wear | 0 (0.0) | 0 (0.0) | 1 (3.0) | 1 (1.0) |
| Tooth Mobility | 2 (6.1) | 0 (0.0) | 0 (0.0) | 2 (2.1) |
| Dental plaque | 1 (3.0) | 0 (0.0) | 0 (0.0) | 1 (1.0) |
| Toothache | 1 (3.0) | 0 (0.0) | 0 (0.0) | 1 (1.0) |
| Malocclusion | 1 (3.0) | 0 (0.0) | 0 (0.0) | 1 (1.0) |
| Gingival Diseases | 1 (3.0) | 0 (0.0) | 2 (6.1) | 3 (3.1) |
| Gingival pain | 1 (3.0) | 0 (0.0) | 1 (3.0) | 2 (2.1) |
| Gingival swelling | 1 (3.0) | 0 (0.0) | 2 (6.1) | 3 (3.1) |

Supplementary Table 10. Phase I trial-summary of TEAE (SS)

|  | 1×10^6^ DPSCs  /1 tooth  N=3 | | 5×10^6^ DPSCs  /1 tooth  N=6 | | 1×10^7^ DPSCs  /1 tooth  N=6 | | 2×10^7^ DPSCs  /2 teeth  N=6 | | 3-4×10^7^ DPSCs  /3-4 teeth  N=6 | | Saline injection  N=9 | | Total  N=9 | |
| --- | --- | --- | --- | --- | --- | --- | --- | --- | --- | --- | --- | --- | --- | --- |
| Classification by system organ  Preferred Term | n | Incidence | n | Incidence | n | Incidence | n | Incidence | n | Incidence | n | Incidence | n | Incidence |
| At least 1 case of TEAE | 0 | 0.0 | 3 | 50.0 | 1 | 16.7 | 1 | 16.7 | 1 | 16.7 | 3 | 33.3 | 0 | 0.0 |
| Gastrointestinal system diseases | 0 | 0.0 | 2 | 33.3 | 0 | 0.0 | 0 | 0.0 | 0 | 0.0 | 0 | 0.0 | 0 | 0.0 |
| Diarrhea | 0 | 0.0 | 1 | 16.7 | 0 | 0.0 | 0 | 0.0 | 0 | 0.0 | 0 | 0.0 | 0 | 0.0 |
| Gingival swelling | 0 | 0.0 | 1 | 16.7 | 0 | 0.0 | 0 | 0.0 | 0 | 0.0 | 0 | 0.0 | 0 | 0.0 |
| All kinds of inspection | 0 | 0.0 | 1 | 16.7 | 1 | 16.7 | 1 | 16.7 | 1 | 16.7 | 1 | 11.1 | 0 | 0.0 |
| Increased blood pressure | 0 | 0.0 | 1 | 16.7 | 0 | 0.0 | 1 | 16.7 | 0 | 0.0 | 0 | 0.0 | 0 | 0.0 |
| Urinary protein detection | 0 | 0.0 | 0 | 0.0 | 1 | 16.7 | 0 | 0.0 | 1 | 16.7 | 0 | 0.0 | 0 | 0.0 |
| Positive occult blood | 0 | 0.0 | 0 | 0.0 | 0 | 0.0 | 0 | 0.0 | 0 | 0.0 | 1 | 11.1 | 0 | 0.0 |
| Heart Organ Diseases | 0 | 0.0 | 1 | 16.7 | 0 | 0.0 | 0 | 0.0 | 0 | 0.0 | 0 | 0.0 | 0 | 0.0 |
| Bradycardia | 0 | 0.0 | 1 | 16.7 | 0 | 0.0 | 0 | 0.0 | 0 | 0.0 | 0 | 0.0 | 0 | 0.0 |
| Infectious and infectious diseases | 0 | 0.0 | 0 | 0.0 | 0 | 0.0 | 0 | 0.0 | 0 | 0.0 | 2 | 22.2 | 0 | 0.0 |
| Periodontitis | 0 | 0.0 | 0 | 0.0 | 0 | 0.0 | 0 | 0.0 | 0 | 0.0 | 1 | 11.1 | 0 | 0.0 |
| Herpes virus infection | 0 | 0.0 | 0 | 0.0 | 0 | 0.0 | 0 | 0.0 | 0 | 0.0 | 1 | 11.1 | 0 | 0.0 |

Supplementary Table 11. Clinical outcomes of DPSCs injection in IIT study

|  |  | Single DPSCs injection | Double DPSCs injection | Saline injection |
| --- | --- | --- | --- | --- |
|  |  | N=32 | N=30 | N=31 |
| P value of AL (△mean±SE, mm) | Day 90 change from baseline | 0.7533（-0.7±1.40) | 0.9416(-0.8±1.01) | 0.8414(-0.8±1.05) |
|  | Day 180 change from baseline | 0.3624(-1.2±1.90) | 0.9506(-0.8±1.05) | 0.3115(-0.8±1.37) |
| P value of PD (△mean±SE, mm) | Day 90 change from baseline | 0.9889(-0.8±1.22) | 0.9462 (-0.8±1.03) | 0.9244(-0.8±0.95) |
|  | Day 180 change from baseline | 0.4652(-1.2±1.65) | 0.6724(-0.7±1.09) | 0.2334(-0.9±1.39) |
| P value of GR (△mean±SE, mm) | Day 90 change from baseline | 0.2879(0.1±0.49) | 0.5685(0.0±0.23) | 0.0979(0.0±0.24) |
|  | Day 180 change from baseline | 0.5179(0.0±0.56) | 0.1058(-0.1±0.31) | 0.5344(0.1±0.30) |
| P value of BOP | Day 90 change from baseline | 0.3326 | 0.5255 | 0.2622 |
|  | Day 180 change from baseline | 0.0837 | 0.3674 | 0.0095 |
| P value of TM | Day 90 change from baseline | 0.7102 | 0.8463 | 0.5653 |
|  | Day 180 change from baseline | 0.3456 | 0.6056 | 0.0761 |
| P value of BDD (△mean±SE, mm) | Day 90 change from baseline | 0.0497* (-0.26±0.336） | 0.5877（-0.04±0.234） | 0.0061(-0.07±0.325） |
|  | Day 180 change from baseline | 0.0083**(-0.30±0.484) | 0.0990（-0.11±0.250） | 0.1121(0.04±0.315） |

Supplementary Table 12. Clinical outcomes of DPSCs injection in phase I trial

|  |  | 1×10^6^ DPSCs/1 tooth  n=3 | 5×10^6^ DPSCs/1 tooth  n=6 | 1×10^7^ DPSCs/1 tooth  n=6 | 2×10^7^ DPSCs/2 teeth  n=12 | 3-4×10^7^ DPSCs/3-4 teeth  n=19 | Saline injection n=16 |
| --- | --- | --- | --- | --- | --- | --- | --- |
| P value of AL (△mean±SE, mm) | Day 90 change from baseline | 0.860（-2.0±0.50) | 1.000（-1.3±1.57) | 0.678（-2.0±0.77) | 1.000（-1.5±0.64) | 0.552（-1.9±1.09) | (-1.4±0.85) |
|  | Day 180 change from baseline | 0.560（-2.2±0.29) | 0.969（-1.7±0.82) | 0.600（-2.0±0.94) | 0.999（-1.5 ±0.83) | 0.440（-1.9±0.96) | (-1.4±0.86) |
| P value of PD (△mean±SE, mm) | Day 90 change from baseline | 1.000（-1.5±0.50) | 0.854（-1.2±1.17) | 0.994（-1.4±0.86) | 0.998（-1.5 ±0.71) | 0.279（-2.3±1.28) | (-1.6±0.92) |
|  | Day 180 change from baseline | 0.993(-1.8±0.29) | 0.988（-1.3 ±1.13) | 0.990（-1.8±0.76) | 1.000（-1.6±1.00) | 0.091（-2.3±1.16) | (-1.6±0.70) |
| P value of GR (△mean±SE, mm) | Day 90 change from baseline | 0.463（-0.5±0.50) | 0.799(-0.2±0.82) | 0.123 (-0.6±1.20) | 0.954(0.0 ±0.21) | 0.937(0.4±0.70) | （0.2 ±0.75) |
|  | Day 180 change from baseline | 0.718 (-0.3±0.58) | 0.463(-0.3±0.41) | 0.797(-0.2±1.25) | 0.999(0.1±0.47) | 0.553( 0.5±0.61) | （0.2 ±0.72) |
| P value of BOP | Day 90 change from baseline | 0.989 | 0.881 | 1.000 | 1.000 | 1.000 |  |
|  | Day 180 change from baseline | 0.989 | 0.881 | 0.966 | 0.999 | 0.975 |  |
| P value of TM | Day 90 change from baseline | 0.190 | 1.000 | 0.577 | 1.000 | 0.942 |  |
|  | Day 180 change from baseline | 0.010 | 1.000 | 0.473 | 1.000 | 0.942 |  |
| P value of BDD (△mean±SE, mm) | Day 90 change from baseline | 0.988(-0.27±0.231) | 0.528 (0.08±0.147) | 0.737 (0.03±0.356) | 1.000(-0.18±0.368 ) | 0.991 (-0.10±0.437) | (-0.16± 0.273) |
|  | Day 180 change from baseline | 1.000 (-0.13±0.231) | 0.531 (0.12±0.354) | 0.892 (0.02±0.526) | 0.999 (-0.20±0.333) | 0.997 (-0.21±0.444) | (-0.16±0.371) |

Supplementary Table 13. Basic information of subjects with periodontitis AL ≥ 5 mm (combined dataset)

|  |  | DPSCs injection N=42 n=54  n(%) | Saline injection N=35 n=40  n(%) | Total N=77 n=94  n(%) |
| --- | --- | --- | --- | --- |
| Sex | Number of cases (missing count) | 42 ( 0) | 35 ( 0) | 77 ( 0) |
|  | Male | 19 ( 45.2) | 11 ( 31.4) | 30 ( 39.0) |
|  | Female | 23 ( 54.8) | 24 ( 68.6) | 47 ( 61.0) |
|  |  |  |  |  |
| Nationality | Number of cases (missing count) | 42 ( 0) | 35 ( 0) | 77 ( 0) |
|  | Han | 40 ( 95.2) | 27 ( 77.1) | 67 ( 87.0) |
|  | Hui | 1 ( 2.4) | 3 ( 8.6) | 4 ( 5.2) |
|  | Man | 1 ( 2.4) | 3 ( 8.6) | 4 ( 5.2) |
|  | Meng | 0 | 2 ( 5.7) | 2 ( 2.6) |
|  |  |  |  |  |
| Age (year) | Number of cases (missing count) | 42 ( 0) | 35 ( 0) | 77 ( 0) |
|  | Mean (SD) | 38.1 (8.78) | 36.4 (8.39) | 37.3 (8.59) |
|  | Median | 37.5 | 35.0 | 36.0 |
|  | Quartile | 33.0, 46.0 | 29.0, 43.0 | 30.0, 44.0 |
|  | Minimum, maximum | 21, 58 | 21, 54 | 21, 58 |
|  |  |  |  |  |
| Height (cm) | Number of cases (missing count) | 42 ( 0) | 35 ( 0) | 77 ( 0) |
|  | Mean (SD) | 168.18 (7.358) | 166.41 (7.446) | 167.37 (7.403) |
|  | Median | 167.00 | 165.00 | 166.00 |
|  | Quartile | 163.00, 172.00 | 161.00, 172.00 | 162.00, 172.00 |
|  | Minimum, maximum | 158.0, 189.0 | 152.0, 183.0 | 152.0, 189.0 |
|  |  |  |  |  |
| Weight (kg) | Number of cases (missing count) | 42 ( 0) | 35 ( 0) | 77 ( 0) |
|  | Mean (SD) | 68.67 (15.153) | 62.44 (10.011) | 65.84 (13.359) |
|  | Median | 69.00 | 58.00 | 65.00 |
|  | Quartile | 58.00, 77.00 | 55.00, 70.00 | 55.00, 73.00 |
|  | Minimum, maximum | 45.0, 120.0 | 50.0, 86.0 | 45.0, 120.0 |
|  | | | | |

Supplementary Table 14. Screening criteria for banking DPSCs

|  | Verification items | Verification standards |
| --- | --- | --- |
| Physical testing | Appearance | Light yellow uniform suspension, no sediment |
|  | Visible foreign matter | No visible foreign objects other than cells to the naked eye |
|  | Differences in packaging | Not lower than indicated |
| Bioassay | Sterility test | Complies |
|  | Baterial endotoxin | Should be less than 3EU/mL |
|  | Mycoplasma test | Negative |
|  | Cytomegalovirus (CMV) test | Negative |
|  | EB virus (EBV) test | Negative |
|  | Human Immunodeficiency Virus (HIV) test | Negative |
|  | Hepatitis B virus (HBV) test | Negative |
|  | Hepatitis C virus (HCV) test | Negative |
|  | Treponema pallidum antibody (TP) test | Negative |
|  | Cell characteristics | Capable of sticking to plastic wells |
|  | Morphology | Adherent spindle cells |
|  | Cell count | 100% ± 20% indicated in the label |
|  | Cell viability | ≥80% |
|  | Cell phenotype | Positive (≥95%): CD73, CD90, CD105;  Negative (≤2%): CD34, CD45, HLA-DR，CD19，CD11b |
|  | Chromosome test | No chromosomal abnormalities |
|  | PEG2 secretion | Not less than 200 pg/3 × 10^5 cells |
|  | Cell differentiation ability *in vitro* | Osteogenic differentiation ability and lipogenic differentiation ability  (Identified by *in vitro* cell culture staining) |

Supplementary Table 15. Immunomodulatory function of banking DPSCs

| Lymphocyte proliferation rate | 95.55%±5.02% |
| --- | --- |
| Th1 cell inhibition rate | 52.57%±8.00% |
| Th2 cell inhibition rate | 44.53%±13.52% |
| Th17 cell inhibition rate | 61.37%±3.53% |
| Treg cell promotion rate | 71.60%±23.40% |
| TNF-α inhibition rate | 75.27%±19.78% |

Supplementary Table 16. Clinical outcomes of the DPSC injection for teeth with single-root or multiple-root

(combined dataset)

|  |  |  | AL<5mm | | | | | | | | AL>=5mm | | | | | | | |
| --- | --- | --- | --- | --- | --- | --- | --- | --- | --- | --- | --- | --- | --- | --- | --- | --- | --- | --- |
|  |  |  | Single-root | | | | Multiple-root | | | | Single-root | | | | Multiple-root | | | |
|  |  |  | TRT | PBO | P value | 95% CI | TRT | PBO | P value | 95% CI | TRT | PBO | P value | 95% CI | TRT | PBO | P value | 95% CI |
|  |  |  | n=5 | n=1 |  |  | n=4 | n=2 |  |  | n=19 | n=8 |  |  | n=35 | n=32 |  |  |
| AL (mm) | baseline |  | 3.5±0.707 (2.62, 4.38) | 3.5±. (., .) | 1 | -2.15, 2.15 | 3.75±0.5 (2.95, 4.55) | 4±0 (., .) | 0.5415 | -1.29, 0.79 | 6.29±1.437 (5.6, 6.98) | 5.75±2.138 (3.96, 7.54) | 0.4488 | -0.9, 1.98 | 6.2±1.389 (5.72, 6.68) | 5.95±1.16 (5.54, 6.37) | 0.4349 | -0.38, 0.87 |
|  | D90 change |  | -0.8±0.57 (-1.51, -0.09) | -0.5±. (., .) | 0.656 | -2.03, 1.43 | -0.63±1.109 (-2.39, 1.14) | -0.75±0.354 (-3.93, 2.43) | 0.8896 | -2.22, 2.47 | -2.13±1.234 (-2.73, -1.54) | -1.75±1.414 (-2.93, -0.57) | 0.4883 | -1.5, 0.74 | -1.14±1.173 (-1.55, -0.74) | -0.86±0.918 (-1.19, -0.53) | 0.2778 | -0.8, 0.23 |
|  | D180 change |  | -0.8±1.037 (-2.09, 0.49) | -0.5±. (., .) | 0.8047 | -3.45, 2.85 | -0.25±0.866 (-1.63, 1.13) | -0.75±0.354 (-3.93, 2.43) | 0.4954 | -1.35, 2.35 | -2.16±1.375 (-2.82, -1.5) | -1.88±1.408 (-3.05, -0.7) | 0.632 | -1.48, 0.92 | -1.4±1.528 (-1.92, -0.88) | -0.86±1.252 (-1.31, -0.41) | 0.1201 | -1.23, 0.14 |
| PD (mm) | baseline |  | 3.5±0.707 (2.62, 4.38) | 3.5±. (., .) | 1 | -2.15, 2.15 | 3.75±0.5 (2.95, 4.55) | 4±0 (., .) | 0.5415 | -1.29, 0.79 | 5.97±1.457 (5.27, 6.68) | 5.19±1.28 (4.12, 6.26) | 0.1978 | -0.44, 2.01 | 5.69±1.207 (5.27, 6.1) | 5.72±1.107 (5.32, 6.12) | 0.9077 | -0.6, 0.53 |
|  | D90 change |  | -0.7±0.758 (-1.64, 0.24) | -0.5±. (., .) | 0.8216 | -2.51, 2.11 | -0.38±1.601 (-2.92, 2.17) | -1±0 (., .) | 0.6301 | -2.71, 3.96 | -2.16±1.434 (-2.85, -1.47) | -2±1.439 (-3.2, -0.8) | 0.7963 | -1.4, 1.09 | -1.26±1.166 (-1.66, -0.86) | -0.88±0.852 (-1.18, -0.57) | 0.1334 | -0.88, 0.12 |
|  | D180 change |  | -1±0.612 (-1.76, -0.24) | -0.5±. (., .) | 0.4975 | -2.36, 1.36 | -0.25±1.555 (-2.72, 2.22) | -1±0 (., .) | 0.5551 | -2.49, 3.99 | -2.42±1.305 (-3.05, -1.79) | -1.81±1.462 (-3.04, -0.59) | 0.2952 | -1.78, 0.56 | -1.47±1.495 (-1.98, -0.96) | -0.94±1.23 (-1.38, -0.49) | 0.1172 | -1.21, 0.14 |
| GR (mm) | baseline |  | 0±0 (., .) | 0±. (., .) | . | ., . | 0±0 (., .) | 0±0 (., .) | . | ., . | 0.32±0.901 (-0.12, 0.75) | 0.63±1.094 (-0.29, 1.54) | 0.4513 | -1.14, 0.52 | 0.51±0.752 (0.26, 0.77) | 0.23±0.523 (0.05, 0.42) | 0.0845 | -0.04, 0.6 |
|  | D90 change |  | 0.1±0.224 (-0.18, 0.38) | 0±. (., .) | 0.704 | -0.58, 0.78 | 0±0 (., .) | 0.25±0.354 (-2.93, 3.43) | 0.1778 | -0.68, 0.18 | -0.03±0.935 (-0.48, 0.42) | 0.25±0.886 (-0.49, 0.99) | 0.4835 | -1.08, 0.52 | 0.14±0.447 (-0.01, 0.3) | 0.02±0.323 (-0.1, 0.13) | 0.19 | -0.06, 0.32 |
|  | D180 change |  | 0.2±0.447 (-0.36, 0.76) | 0±. (., .) | 0.704 | -1.16, 1.56 | 0.25±0.5 (-0.55, 1.05) | 0.25±0.354 (-2.93, 3.43) | 1 | -1.12, 1.12 | 0.26±0.933 (-0.19, 0.71) | -0.06±0.32 (-0.33, 0.21) | 0.3493 | -0.38, 1.03 | 0.04±0.586 (-0.16, 0.24) | 0.08±0.339 (-0.04, 0.2) | 0.7668 | -0.27, 0.2 |
| TM, n (%) | baseline | (—) | 3 (60.00) | 1 (100.00) | 1 |  | 4 (100.00) | 2 (100.00) | - |  | 12 (63.16) | 5 (62.50) | 1 |  | 24 (68.57) | 29 (90.63) | 0.0256 |  |
|  |  | I° | 2 (40.00) | 0 |  |  | 0 | 0 |  |  | 6 (31.58) | 3 (37.50) |  |  | 9 (25.71) | 1 (3.13) |  |  |
|  |  | II° | 0 | 0 |  |  | 0 | 0 |  |  | 1 (5.26) | 0 |  |  | 2 (5.71) | 2 (6.25) |  |  |
|  | D90 change | (—) | 4 (80.00) | 1 (100.00) | 1 |  | 4 (100.00) | 2 (100.00) | - |  | 15 (78.95) | 6 (75.00) | 0.7381 |  | 27 (77.14) | 28 (87.50) | 0.0625 |  |
|  |  | I° | 1 (20.00) | 0 |  |  | 0 | 0 |  |  | 3 (15.79) | 2 (25.00) |  |  | 8 (22.86) | 2 (6.25) |  |  |
|  |  | II° | 0 | 0 |  |  | 0 | 0 |  |  | 1 (5.26) | 0 |  |  | 0 | 2 (6.25) |  |  |
|  | D180 change | (—) | 5 (100.00) | 1 (100.00) | - |  | 4 (100.00) | 2 (100.00) | - |  | 16 (84.21) | 5 (62.50) | 0.3715 |  | 33 (94.29) | 29 (90.63) | 0.7983 |  |
|  |  | I° | 0 | 0 |  |  | 0 | 0 |  |  | 0 | 1 (12.50) |  |  | 1 (2.86) | 1 (3.13) |  |  |
|  |  | II° | 0 | 0 |  |  | 0 | 0 |  |  | 3 (15.79) | 2 (25.00) |  |  | 1 (2.86) | 2 (6.25) |  |  |
| BDD (mm) | baseline |  | 1.24±1.155 (-0.19, 2.67) | 0.4±. (., .) | 0.5429 | -2.67, 4.35 | 1.65±0.794 (0.39, 2.91) | 1.6±0 (., .) | 0.9371 | -1.6, 1.7 | 2.21±1.081 (1.69, 2.73) | 2.41±2.18 (0.59, 4.24) | 0.7478 | -1.48, 1.08 | 2.67±1.368 (2.2, 3.14) | 1.93±1.249 (1.48, 2.38) | 0.0255 | 0.09, 1.38 |
|  | D90 change |  | -0.18±0.415 (-0.69, 0.33) | -0.2±. (., .) | 0.967 | -1.24, 1.28 | -0.08±0.369 (-0.66, 0.51) | -0.35±0.212 (-2.26, 1.56) | 0.3986 | -0.53, 1.08 | -0.24±0.478 (-0.47, -0.01) | -0.09±0.36 (-0.39, 0.21) | 0.4362 | -0.54, 0.24 | -0.12±0.321 (-0.23, -0.01) | -0.08±0.311 (-0.19, 0.03) | 0.5922 | -0.2, 0.11 |
|  | D180 change |  | -0.12±0.179 (-0.34, 0.1) | -0.1±. (., .) | 0.9236 | -0.56, 0.52 | -0.25±0.493 (-1.03, 0.53) | -0.4±0.283 (-2.94, 2.14) | 0.7199 | -0.93, 1.23 | -0.28±0.462 (-0.51, -0.06) | -0.14±0.472 (-0.53, 0.26) | 0.461 | -0.55, 0.26 | -0.21±0.48 (-0.38, -0.05) | 0.02±0.314 (-0.09, 0.13) | 0.0247 | -0.43, -0.03 |

Supplementary Table 17. Clinical outcomes of the DPSC injection for multiple-root teeth with furcation involvement or without furcation involvement (combined dataset)

|  |  |  | AL<5mm | | | | | | | | AL>=5mm | | | | | | | |
| --- | --- | --- | --- | --- | --- | --- | --- | --- | --- | --- | --- | --- | --- | --- | --- | --- | --- | --- |
|  |  |  | With furcation involvement | | | | Without  furcation involvement | | | | With furcation involvement | | | | Without  furcation involvement | | | |
|  |  |  | TRT | PBO | P value | 95% CI | TRT | PBO | P value | 95% CI | TRT | PBO | P value | 95% CI | TRT | PBO | P value | 95% CI |
|  |  |  | n=3 | n=1 |  |  | n=1 | n=1 |  |  | n=12 | n=16 |  |  | n=23 | n=16 |  |  |
| AL (mm) | baseline |  | 3.67±0.577 (2.23, 5.1) | 4±. (., .) | 0.6667 | -3.2, 2.54 | 4±. (., .) | 4±. (., .) | . | ., . | 6.63±1.583 (5.62, 7.63) | 6.19±1.031 (5.64, 6.74) | 0.3839 | -0.58, 1.45 | 5.98±1.257 (5.43, 6.52) | 5.72±1.264 (5.04, 6.39) | 0.5308 | -0.57, 1.09 |
|  | D90 change |  | -0.5±1.323 (-3.79, 2.79) | -1±. (., .) | 0.7745 | -6.07, 7.07 | -1±. (., .) | -0.5±. (., .) | . | ., . | -0.92±1.329 (-1.76, -0.07) | -1.22±0.856 (-1.67, -0.76) | 0.471 | -0.55, 1.15 | -1.26±1.096 (-1.73, -0.79) | -0.5±0.856 (-0.96, -0.04) | 0.0257 | -1.42, -0.1 |
|  | D180 change |  | -0.17±1.041 (-2.75, 2.42) | -1±. (., .) | 0.5598 | -4.34, 6 | -0.5±. (., .) | -0.5±. (., .) | . | ., . | -1.13±1.367 (-1.99, -0.26) | -1.41±1.268 (-2.08, -0.73) | 0.579 | -0.75, 1.31 | -1.54±1.616 (-2.24, -0.84) | -0.31±0.998 (-0.84, 0.22) | 0.0103 | -2.15, -0.31 |
| PD (mm) | baseline |  | 3.67±0.577 (2.23, 5.1) | 4±. (., .) | 0.6667 | -3.2, 2.54 | 4±. (., .) | 4±. (., .) | . | ., . | 6.25±1.438 (5.34, 7.16) | 5.69±1.138 (5.08, 6.29) | 0.2581 | -0.44, 1.56 | 5.39±0.977 (4.97, 5.81) | 5.75±1.111 (5.16, 6.34) | 0.293 | -1.04, 0.32 |
|  | D90 change |  | -0.17±1.893 (-4.87, 4.54) | -1±. (., .) | 0.7397 | -8.57, 10.24 | -1±. (., .) | -1±. (., .) | . | ., . | -1.04±1.196 (-1.8, -0.28) | -1.13±0.785 (-1.54, -0.71) | 0.8256 | -0.69, 0.85 | -1.37±1.16 (-1.87, -0.87) | -0.63±0.866 (-1.09, -0.16) | 0.036 | -1.44, -0.05 |
|  | D180 change |  | 0.17±1.607 (-3.83, 4.16) | -1±. (., .) | 0.5938 | -6.82, 9.15 | -1.5±. (., .) | -1±. (., .) | . | ., . | -1.21±1.484 (-2.15, -0.27) | -1.41±1.281 (-2.09, -0.72) | 0.7084 | -0.88, 1.27 | -1.61±1.515 (-2.26, -0.95) | -0.47±1.008 (-1.01, 0.07) | 0.0124 | -2.02, -0.26 |
| GR (mm) | baseline |  | 0±0 (., .) | 0±. (., .) | . | ., . | 0±. (., .) | 0±. (., .) | . | ., . | 0.38±0.678 (-0.06, 0.81) | 0.5±0.483 (0.24, 0.76) | 0.5733 | -0.58, 0.33 | 0.59±0.793 (0.24, 0.93) | -0.03±0.427 (-0.26, 0.2) | 0.0073 | 0.18, 1.06 |
|  | D90 change |  | 0±0 (., .) | 0±. (., .) | . | ., . | 0±. (., .) | 0.5±. (., .) | . | ., . | 0.21±0.396 (-0.04, 0.46) | -0.09±0.328 (-0.27, 0.08) | 0.0363 | 0.02, 0.58 | 0.11±0.476 (-0.1, 0.31) | 0.13±0.289 (-0.03, 0.28) | 0.9035 | -0.29, 0.25 |
|  | D180 change |  | 0±0 (., .) | 0±. (., .) | . | ., . | 1±. (., .) | 0.5±. (., .) | . | ., . | 0.08±0.469 (-0.21, 0.38) | 0±0.365 (-0.19, 0.19) | 0.601 | -0.24, 0.41 | 0.02±0.648 (-0.26, 0.3) | 0.16±0.301 (0, 0.32) | 0.445 | -0.49, 0.22 |
| TM, n (%) | baseline | (—) | 3 (100.00) | 1 (100.00) | - |  | 1 (100.00) | 1 (100.00) | - |  | 7 (58.33) | 15 (93.75) | 0.0688 |  | 17 (73.91) | 14 (87.50) | 0.1497 |  |
|  |  | I° | 0 | 0 |  |  | 0 | 0 |  |  | 3 (25.00) | 0 |  |  | 6 (26.09) | 1 (6.25) |  |  |
|  |  | II° | 0 | 0 |  |  | 0 | 0 |  |  | 2 (16.67) | 1 (6.25) |  |  | 0 | 1 (6.25) |  |  |
|  | D90 change | (—) | 3 (100.00) | 1 (100.00) | - |  | 1 (100.00) | 1 (100.00) | - |  | 8 (66.67) | 14 (87.50) | 0.3541 |  | 19 (82.61) | 14 (87.50) | 0.0644 |  |
|  |  | I° | 0 | 0 |  |  | 0 | 0 |  |  | 4 (33.33) | 2 (12.50) |  |  | 4 (17.39) | 0 |  |  |
|  |  | II° | 0 | 0 |  |  | 0 | 0 |  |  | 0 | 0 |  |  | 0 | 2 (12.50) |  |  |
|  | D180 change | (—) | 3 (100.00) | 1 (100.00) | - |  | 1 (100.00) | 1 (100.00) | - |  | 12 (100.00) | 15 (93.75) | 1 |  | 21 (91.30) | 14 (87.50) | 0.7416 |  |
|  |  | I° | 0 | 0 |  |  | 0 | 0 |  |  | 0 | 1 (6.25) |  |  | 1 (4.35) | 0 |  |  |
|  |  | II° | 0 | 0 |  |  | 0 | 0 |  |  | 0 | 0 |  |  | 1 (4.35) | 2 (12.50) |  |  |
| BDD (mm) | baseline |  | 1.47±0.862 (-0.68, 3.61) | 1.6±. (., .) | 0.9057 | -4.42, 4.15 | 2.2±. (., .) | 1.6±. (., .) | . | ., . | 2.63±0.753 (2.15, 3.1) | 1.78±1.134 (1.18, 2.39) | 0.0346 | 0.07, 1.62 | 2.69±1.614 (1.99, 3.39) | 2.09±1.375 (1.35, 2.82) | 0.2306 | -0.4, 1.61 |
|  | D90 change |  | -0.07±0.451 (-1.19, 1.05) | -0.5±. (., .) | 0.4928 | -1.81, 2.67 | -0.1±. (., .) | -0.2±. (., .) | . | ., . | -0.22±0.386 (-0.46, 0.03) | -0.17±0.357 (-0.36, 0.02) | 0.7369 | -0.34, 0.24 | -0.07±0.278 (-0.19, 0.05) | 0.01±0.235 (-0.12, 0.13) | 0.3528 | -0.25, 0.09 |
|  | D180 change |  | -0.27±0.603 (-1.76, 1.23) | -0.6±. (., .) | 0.6792 | -2.66, 3.33 | -0.2±. (., .) | -0.2±. (., .) | . | ., . | -0.43±0.531 (-0.76, -0.09) | -0.04±0.358 (-0.23, 0.15) | 0.0316 | -0.73, -0.04 | -0.1±0.421 (-0.28, 0.08) | 0.08±0.259 (-0.06, 0.22) | 0.1347 | -0.42, 0.06 |
