# Supplementary material for: Impact of allogeneic dental pulp stem cell injection on tissue regeneration in periodontitis: a multicenter randomized clinical trial
Source: Signal Transduct Target Ther. 2025 Jul 31;10:239. doi: 10.1038/s41392-025-02320-w (PMC12311062; doi:10.1038/s41392-025-02320-w)
Supplement: Supplementary file 2 — Supplementary File 1 [file 41392_2025_2320_MOESM2_ESM.pdf]

Clinical study protocol

Clinical study on allogeneic human dental pulp stem cells in the treatment of chronic periodontitis

Randomized, open, controlled clinical study of basic periodontal therapy combined with DPSCs injection for chronic moderate periodontitis

Protocol number: hDPMSC-CP-03-2019

Version: Version V 7.0, July 16,2021

Project contractor: Beijing Stomatological Hospital Affiliated to Capital Medical University

Address: No.4, Tiantan Xili, Dongcheng District, Beijing

Project source: Beijing Municipal Commission of Science and Technology, Beijing SH Biotechnology Co.,Ltd.,Beijing 100070, China

Principal investigator: Wang Songling, Liu Yi

Project undertaking departments: Periodontology Department and emergency comprehensive treatment Center

Project partner: Beijing SH Biotechnology Co.,Ltd., Beijing 100070, China

Management of the hospital, department: Science and Technology Department

Project start and end time: June 2018 to August 2021, postponed to August 2022

declaration of secrecy

This clinical research protocol contains commercial secrets and the commercial information is privileged. All information about the reagents contained in the protocol belongs to Beijing SH Biotechnology Co.,Ltd., Beijing 100070, China, and shall not be disclosed without law. Therefore, it is only provided to researchers, co-investigators, ethics committees and regulatory authorities. Without written approval of the sponsor, except to the subject who may participate in the study.

Principal Investigator:

Principal Investigator	Wang Songling and Liu Yi,<br>Beijing Stomatological Hospital Affiliated to Capital Medical University		
Address	No.4 Tiantan Xili, Dongcheng District, Beijing		
zip code	100050	telephone	13601324511、 18600262811

Project name:
Clinical study on allogeneic human dental pulp stem cells in the treatment of chronic periodontitis
Randomized, open, controlled clinical study of basic periodontal therapy combined with DPSCs injection for chronic moderate periodontitis

(hDPMSC-CP-03-2019)

List of researchers

01	Beijing Stomatological Hospital affiliated to Capital Medical University	The Institute of Oral Medicine	Wang Songling
02	Beijing Stomatological Hospital affiliated to Capital Medical University	Periodontology	Liu Yi
03	Beijing Stomatological Hospital affiliated to Capital Medical University	Periodontology	Hu Jingchao
04	Beijing Stomatological Hospital affiliated to Capital Medical University	Emergency department comprehensive diagnosis and treatment center	Cao Yu
05	Beijing Stomatological Hospital affiliated to Capital Medical University	Emergency department comprehensive diagnosis and treatment center	Jin Lu yuan
06	Beijing Stomatological Hospital affiliated to Capital Medical University	Periodontology	Ji Jin
07	Beijing Stomatological Hospital affiliated to Capital Medical University	Periodontology	Wang Minfeng
08	Beijing Stomatological Hospital affiliated to Capital Medical University	Periodontology	Song Lin

The researcher's statement

I promise:

1. This clinical study protocol ("Clinical study on allogeneic human dental pulp stem cells in the treatment of chronic periodontitis（Randomized, open, controlled clinical study of basic periodontal therapy combined with DPSCs injection for chronic moderate periodontitis"), protocol No.: hDPMSC-CP-03-2019), the research content is from the Beijing Science and Technology Plan Project (Project No.: Z 181100001718208). For the inconsistent protocol, see Annex 1: Clinical Study Protocol Modification Comparison Table.
2. This clinical study was conducted in strict accordance with the requirements of the Declaration of Helsinki, current Chinese regulations and clinical study protocol.
3. Standardize and record and preserve the raw data of clinical studies.
4. To allow the regulatory authorities to examine the clinical study.

I have read all the clinical study protocol,including the above statement. I agree to all the above contents and will keep all the above contents confidential.

Project undertaking unit:

Beijing Stomatological Hospital affiliated to Capital Medical University

Principal Investigator（signature）: ____________ Principal Investigator（signature）: ________

date: _________ date: ___________

The researcher's statement

I promise:

1. This clinical study protocol ("Clinical study on allogeneic human dental pulp stem cells in the treatment of chronic periodontitis（Randomized, open, controlled clinical study of basic periodontal therapy combined with DPSCs injection for chronic moderate periodontitis"), protocol No.: hDPMSC-CP-03-2019), the research content is from the Beijing Science and Technology Plan Project (Project No.: Z 181100001718208). For the inconsistent protocol, see Annex 1: Clinical Study Protocol Modification Comparison Table.
2. This clinical study was conducted in strict accordance with the requirements of the Declaration of Helsinki,current Chinese regulations and clinical study protocol.
3. Standardize and record and preserve the raw data of clinical studies.
4. To allow the regulatory authorities to examine the clinical study.

I have read all the clinical study protocol, including the above statement. I agree to all the above contents and will keep all the above contents confidential.

Preparation preparation facility：

Beijing SH Biotechnology Co.,Ltd., Beijing 100070, China

Project Leader (Signature): ：________

date: ：________

catalogue

Thumbnail control table 6
scenario summary 7
Flow chart of the study 14
(1) Research topic 15
(2) Research purpose 15
(3) the basis for the establishment of the topic 15
(4) The expected results 16
(5) Study design 17
(6) Conditions for research and implementation 25
(7) Subject inclusion, exclusion criteria and entry method of assignment 26
(8) the number of cases required 28
(9) Subjectmanagement 28
(10) Subjectmotivationandcompensation 29
(11) Subject privacy and confidentiality 30
(12) The use method, dose, time and course of treatment of stem cell preparations 31
(13) Management of stem cell preparations 32
(14) criteria for suspension and termination of clinical studies 32
(15) Efficacy effect evaluation criteria 33
(16) Requirements for recording of adverse events and reporting methods and handling measures for serious adverse events 35
(17) The risks and benefits of the study 38
(18) Sample draft of the case report form (see Annex 2: Case Report Form (CRF) 》) 40
(19) Statistical analysis of the research results 40
(20) Plan and implementation measures of follow-up 41
(21) Quality control and assurance of the research 45
reference 47

Thrill control table

MSC	mesenchymal stem cells
hDP-MSC	Humanpulp mesenchymal stem cells
AL	attachment loss
PD	Periodontal depth
BOP	bleeding on probing
GR	gingival recession
TM	tooth mobility
CBCT	Cone-beam CT
HBsAg	hepatitis b surface antigen
HBsAb	hepatitis b surface antibody
HBeAg	hepatitis b e antigen
HBeAb	hepatitis b e antibody
HBcAb	hepatitis b core antibody
Anti-HCV	Antibody to Hepatitis C
HIVcombin	HIV combined antibodies
Anti-TP	Treponema pallidum
CMV-IgM	cytomegalovirus antibody IgM
CMV-IgG	cytomegalovirus antibody IgG
IgA	immunoglobulin A
IgG	immunoglobulin G
IgM	immunoglobulin M
T-IgE	total IgE
β-HCG	β-human chorionic gonadotropin
GCP	Good Clinical Practice for Drugs
FAS	full analysis set
PPS	per-protocol set
SS	safety set
LOCT	last observation carried forward
SOP	standard operating procedure
AE	adverse event
SAE	serious adverse event
ICF	informed consent form
CRF	Case report form
eCRF	Electronic case report form
EDC	Electronic data collection

scenario summary

**Study protocol number**	hDPMSC-CP-03-2019
**Scenario name**	Clinical study on allogeneic human dental pulp stem cells in the treatment of chronic Periodontitis （Randomized, open, controlled clinical study of basic periodontal therapy combined with DPSCs injection for chronic moderate periodontitis）
**Version number / date**	Version V 7.0, July 16,2021
**Research category**	Clinical exploratory studies
**Research preparation**	Human dental pulp mesenchymal stem cells（hDP-MSC）
**indication**	Chronic and moderate periodontitis
**purpose of research**	1. To evaluate the safety and efficacy of DPSCs injection in chronic moderate periodontitis;<br>2. Provide a basis for the formulation of clinical standard protocol for the application of human dental pulp MSC cells for the treatment of chronicmoderate periodontitis.
**Total number of cases**	96 cases
**Number of research centers**	one
**The study period**	From June 2018 to August 2021, and extended to August 2022
research design	This study is a single-center, randomized, controlled study. After subjects with a clinical diagnosis of chronic moderate periodontitis signed the informed consent and met the inclusion criteria and not the exclusion criteria, the investigator selected the proposed site (individual tooth position / subject). All subjects received basic periodontal treatment and received bilateral multipoint local injections of hDP-MSC cells / saline on individual tooth positions immediately after completion of the basic periodontal treatment. All subjects were observed for 2 hours after study treatment, and their clinical safety and efficacy observation measures were collected until the end of the study at 12 months.<br><br>（1）Randomized, open, and controlled study design<br>The study was conducted in 2 phases to recruit 96 subjects.<br>In the first stage, two groups will be enrolled in 46 subjects in a 1:1 ratio. The group design is as follows:<br>1. DPSCs injection group: bilateral multisite injection immediately after the completion of basic periodontal therapy , a single therapeutic dose of 1107Of hDP-MSC cells (saline 0.6mLsuspension) / tooth position / person, about 23 subjects;<br>2. Saline iniection group: immediately after the completion of basic periodontal treatment Injection, with one treatment dose of 0.6mL saline / tooth position / person, enrolled approximately 23 subjects.<br>In the second stage,3 groups were set up, and 50 subjects were enrolled at random according to the ratio of 1:1:3. As follows:

	**1. Saline iniection group**: immediately after the completion of basic periodontal treatment Injection, therapeutic dose of 0.6mL saline / dental position / person, 10 subjects;<br><br>**2. Single DPSCs injection group**: Bilateral multi-point injection was performed on a single tooth position of the subject immediately after the completion of basic periodontal treatment ejaculation, with a therapeutic dose of 1×107 hDP-MSC cells (saline 0.6mL suspension)/dental position/person, 10 subjects were enrolled;<br><br>**3. Double DPSCs iniection group**: Immediately after the completion of periodontal basic treatment, bilateral multi-point injection was performed on the single tooth of the subject, and the therapeutic dose was 1 × 107hDP-MSC cells (0.6mL saline suspension)/tooth position/person. A month later, bilateral multi-point injection was performed on the single tooth of the subject again. The therapeutic dose was also 1 × 107 HDP-MSC cells (0.6mL suspension of normal saline)/dental position/person, and 30 subjects were enrolled.<br><br>In summary, in the two stages of this study, about 33 subjects are expected to be included in the normal saline control group, about 33 subjects in the stem cell treatment group, and 30 subjects in the stem cell secondary administration group, meeting the requirements of statistical analysis.<br><br>**(2) Design of cell therapy dose**<br><br>According to the results of previous studies, the local cell injection cell dose is: 1107Cell / 0.6 mL / tooth position / human.<br><br>**(3) Blind setting**: This study was an open study, and only periodontal data collection personnel and image data measurement personnel were kept blind. In this study, the appearance of human dental pulp mesenchymal stem cell preparations after suspension before use was pale milky uniform suspension, and the appearance of normal saline control was colorless clear liquid, so it was impossible to implement blind method for subjects and researchers conducting local injection operations.
Target subject population	**Inclusion criteria**: Subjects must meet all of the following criteria to be included in this study:<br>(1) Age: 18-65 years old, with no gender limit;<br>(2) To voluntarily participate in this clinical study and sign the Informed Consent Form;<br>(3) The radiological detection of the periodontal defect site is a wedge-shaped bone defect;<br>(4) The Periodontal depth (PD) of the periodontal defect site is 4 to 8 mm.<br><br>**Inclusion criteria**: Subjects must meet all of the following criteria to be included in this study:<br>(1) Blood pressure of hypertensive subjects within 1 month before surgery: systolic blood pressure ≥180mmHg or diastolic blood pressure ≥110mmHg;<br>(2) Patients with systemic diseases (malignant tumor subjects or tumor test positive during screening, diabetic subjects, heart failure caused by heart disease, myocardial infarction within six months; Symptoms of angina pectoris developed within six months; Congenital heart disease.) ;<br>(3) Use of non-steroidal anti-inflammatory drugs, steroid (steroid) hormone therapy within 3 months before surgery, and/or long-term use of hormones, use of bisphosphonates;

	(4) systemic infection;<br>(5) Patients with periodontal defect sites and adjacent periodontal tissues underwent surgical treatment;<br>(6) people who are known to be allergic to any material used in the procedure; (Allergic constitution, history of allergy to blood products;)<br>(7) Patients with severe periodontal disease (alveolar bone resorption exceeding two-thirds of tooth root length);<br>(8) severe hepatic and renal insufficiency;<br>(9) Those with bleeding tendency or coagulation dysfunction (white blood cell count WBC< 3.0 × 109/L or a platelet meter Number (PLT) < 60× 109/L);<br>(10) People infected with HIV or hepatitis B virus;<br>(11) Study of unprotected sex within one month before screening;<br>(12) Pregnant and lactating women or women using estrogen contraception;<br>(13) Patients whose spouse or I plan to become pregnant during the study period and within 6 months after the end of the study;<br>(14) Smokers (current > 10 PCS/day);<br>(15) Those who are expected to survive less than 12 months;<br>(16) Other circumstances deemed unsuitable by the researcher.<br><br>**Exit/drop off criteria:**<br>（1） The subject has an adverse event during the study, and the investigator believes that the study can no longer be continued;<br>（2） The subjects had poor compliance and could not complete the follow-up on time;<br>（3） Use of other drugs that affect tolerance or safety judgments, such as glucocorticoids, bisphosphonates, and females Hormones;<br>（4） the use of other foods that affect tolerance or safety judgments, such as betel nut; It takes a long time to chew to cause biting The combined burden of resilient food such as beef tendon;<br>（5） The subject is unwilling to continue the clinical study and proposes to withdraw from the investigator;<br>（6） Female subjects became pregnant during the study;<br>（7） Loss of follow-up.
Study termination	**Termination criteria**: If any of the following issues are found in a clinical study, the study is terminated:<br>（1） Serious safety issues occurred in the study (such as serious adverse reactions or serious complications or Rapid deterioration of the condition);<br>（2） The effect found in the study is poor, or even ineffective, and does not have clinical value, and its continuation will be delayed Subject treatment;<br>（3） Major errors in the formulation of clinical research protocols or major deviations in the implementation of clinical research protocols are found in the study, and then continue The continuation is difficult to evaluate.

Research procedure	**(1) Screening for inclusion (D-14) :** After the subjects signed the informed consent, all pre-enrollment screening examinations were conducted in the periodontal/emergency comprehensive clinic of the research institution. Subjects who met the inclusion criteria and did not meet the exclusion criteria were selected Join the group. The investigator selected the subject's proposed treatment site (single tooth position/subject).<br>**(2)Periodontal primary treatment (D-14~D1) :** Periodontal primary treatment includes supragingival scaling, subgingival scaling and root treatment The surface is flat. Divided into 3 times, the specific treatment steps are as follows:<br>■ Supragingival cleaning (D- 14) : normal saline rinse and gargle, whole mouth ultrasonic supragingival cleaning and polishing, normal saline rinse;<br>■ Subgingival scaling (D-7±1, non-therapeutic dental area) : 2% bilan anesthesia, half-mouth ultrasonic subgingival scaling, flat root surface, polishing, normal saline rinse;<br>■ Subgingival scaling (D1, including treatment area) : 2% bilan anesthesia, the other half of the mouth ultrasound subgingival scaling, root surface flat, polishing, normal saline rinse.<br><br>**(3) Local injection therapy (D1) :** According to the randomly assigned number, all subjects were given bilateral multi-point local injection therapy of hDP-MSC cells/normal saline immediately after the peridental foundation treatment was completed. The local injection method for the peridental defect site is: Under local anesthesia, the periodontal probe was used to determine the top position of the alveolar ridge, and the needle was injected into the bottom of the periodontal bag (in the surrounding tissue at the bottom of the periodontal bag). After the needle reached the medial periodontal membrane of the alveolar ridge and touched the junction between the bone surface and the tooth root, the multi-point interstitial injection was performed on both sides of the buccal (lip) and lingual and palatine sides with a 1ml spiral syringe. The injection time is 20-30 seconds at each point. The stop point of the injection needle is located on the tooth root surface to ensure that the injected stem cell preparation can be in the tooth root The area is clustered. Specific operations are as follows:<br>■ DPSCs injection group: 48 subjects. Immediately after the completion of periodontal basic treatment, the patient was given bilateral multi-point injection at a single dental position, and the therapeutic dose was 1× 107 hDP-MSC cells (0.6mL suspension of normal saline)/dental position/person.<br>■ Saline iniection group: 48 subjects. Immediately after the completion of periodontal basic treatment, the patient was given bilateral multi-point injection at a single dental position, and the therapeutic dose was 0.6mL normal saline/dental position/person.<br><br>**Local stem cell secondary injection therapy (D30±3) :** Only for secondary stem cells in the second phase of this study Subjects in the administration group. At the end of a one-month follow-up, the subjects were given more hDP-MSC cells on both sides Point local injection therapy. The local injection method of periodontal defect site is the same as the first local injection treatment:<br>■Double DPSCs iniection group: After 1 month follow-up, the subjects received bilateral multi-point injection at a single dental position and the therapeutic dose It was 1× 107 HDP-MSC cells (saline 0.6mL suspension)/dental position/person.<br><br>**(4) Observation (D1)** : After treatment, all subjects were admitted to the periodontal/emergency comprehensive clinic 2 Hours of adverse reactions observation and safety assessment.<br><br>**(5) Follow-up (D7±1 telephone follow-up, D30±3, D90±3, D180±5, D360±5) :**<br>■ **Post-treatment follow-up**: Subjects were followed up at D7±1 (telephone follow-up), D30±3, D90±3, and D180±5 once each after discharge, and clinical safety and efficacy indexes were collected.<br>■ **Long-term follow-up**: After the end of treatment, D360±5 follow-up was conducted once, clinical efficacy and immunological evaluation indicators were collected, and the study was ended.
**Safety Assessment**	。Vital signs: Respiration, heart rate, blood pressure (systolic, diastolic), temperature (armpits) , Clinical evaluation of the general condition

	During the screening period, before treatment, 2h±30min after the end of treatment, D30±3 during the follow-up period, and 2h±30min after the end of treatment in the second phase of stem cell secondary administration group in this study.<br>②Laboratory tests<br>● Routine blood count: white blood cells, red blood cells, hemoglobin, hematocrit, mean corpuscular volume, mean hemoglobin amount, mean hemoglobin concentration, platelet count, mean platelet volume, thrombocrit, neutrophils, lymphocytes, monocytes, eosinophils, basophils, neutrophils absolute, lymphocyte absolute, monocyte absolute, eosinophil absolute, basophil absolute, erythrocyte distribution width SD, erythrocyte distribution width CV, large platelet ratio, platelet fraction cloth width; During the screening period, before treatment, and during the follow-up period, D30±3, D90±3, D180±5 were examined.<br>● Coagulation: plasma prothrombin time, international normalized ratio of prothrombin time, activated partial thromboplastin time, plasma prothrombin time, plasma fibrinogen; at screening and before treatment.<br>● Liver and renal function: alanine aminotransferase, aspartate aminotransferase, total protein, albumin, globulin, white globule ratio, total bilirubin, direct bilirubin, indirect bilirubin, alkaline phosphatase, creatinine, gamma-glutamyltransferase, lactate dehydrogenase, creatine kinase, urea, uric acid, total carbon dioxide, glucose; D30±3, D90±3, and D180±5 at Screening, Pre-treatment, and Follow-up Periods.<br>● Inflammatory indicators: ultrasensitive C-reactive protein, examined during the screening period, before treatment, 2h±30min after the end of treatment, the and subjects in the secondary stem cell dosing group in the second phase of this study before, at the end of the secondary dosing treatment 2h±30min after<br>● Infectious disease testing： HBsAg 、 HBsAb 、 HBeAg 、 HBeAb 、 HBcAb 、 Anti-HCV 、 HIVcombin 、Anti-TP 、CMV-IgM 、CMV-IgG；During the screening period and follow-up period, D30±3, D90±3, D180±5 were examined.<br>● Immunological tests: IgA, IgG, IgM, total IgE; examined during the screening period, pre-treatment, 2h±30min after the end of treatment, D30±5, D90±5, D180±5, D360±5 during the follow-up period and 2h±30min after the end of the secondary administration of treatment for subjects in the secondary administration group of the second phase of the present study of stem cells.<br>● Pregnancy test: blood β-HCG, D30±5, D90±5, D180±5 in premenopausal female subjects during screening, pre-treatment, and follow-up periods.<br>● Urine routine: pH, specific gravity, protein, ketone bodies, bilirubin, urobilinogen, nitrite, leukocytes, erythrocytes, urine glucose, urine urine urine test. cells, erythrocytes, urine glucose, urine sediment microscopy, in the screening period, before treatment.<br>For subjects with adverse reactions, timely treatment, follow-up inspection and recording should be carried out in accordance with the diagnosis and treatment routine.<br><br>③Adverse events and serious adverse events: The occurrence of adverse events and serious adverse events was recorded in a timely manner during the study, and the degree of adverse events was determined according to the NCI CTCAE v4.03 grading standard. Swollen and painful gums within one week after surgery will be recorded as an adverse event and judged by the investigator to be a treatment-related adverse reaction.

**Effectiveness evaluation**	**①Primary efficacy measures**<br>■ Oral clinical testing indicators: at screening periods, pre-treatment periods, and follow-up periods D90±3, D180±5, D360±5, examination was performed using a Florida probe, and the measurement of D90±3 was used as the evaluation endpoint.<br>■ attachment level AL (Periodontal depth PD + gingival recession GR).<br>**② Secondary efficacy indicators**<br>■ Imaging test indicators: at screening periods and follow-up periods D90±3, D180±5, and D360±5, examinations was performed using a Florida probe, and the screening periods was used as baseline, D180±5 measured value was used as the evaluation endpoint.<br>•Changes in periodontal defect height at 3, 6, and 12 months after the end of treatment compared with baseline (CBCT measurements, change of periodontal defect height = difference in distance from the cementoenamel to the top of the alveolar ridge).<br>•Changes in mean alveolar ridge density at 3, 6, and 12 months after the end of treatment compared to baseline (CBCT measurements).<br><br>■ Oral clinical testing index: at screening periods and follow-up periodsD90±3, D180±5, D360±5, using Florida probe for examination, use the measurement of D90±3 as the evaluation endpoint.<br>•PD compared to baseline;<br>•Periodontal healing compared to baseline (probing bleeding index BOP, gingival recession GR, tooth mobility TM).
**Statistical methods**	The phases of this study were randomized using block group randomization, with statisticians using SAS 9.4 software to generate random grouping codes. In Phase I, subjects were assigned in a 1:1 ratio according to the code to DPSCs injection group and saline injection group, and the randomization envelopes were recovered by the statistical management unit after the completion of this phase. In phase II, subjects were assigned to the saline injection group, single DPSCs injection group and double DPSCs injection group in a ratio of 1:1:3 according to the phase code. Statisticians were not involved in the screening, inclusion of subjects and subsequent clinical studies. The evaluators of the measurements were kept blinded during the study.<br>Statistical analysis datasets included the full analysis set (FAS), per protocol set (PPS), safety set (SS).<br>Analyses included subject distribution, balance analysis of baseline indicators, efficacy analysis, and safety analysis.<br>Means, standard deviations, medians, quartiles, minimum and maximum values were used for statistical description of measures, and ANOVA was used for comparisons between groups.<br>Statistical descriptions of count and rank information were made using rates or component ratios, and comparisons of count information were made using the x2 test or the Fisher exact probability method.
**Technical Support**	The human dental pulp mesenchymal stem cell preparations used in this project were manufactured by Beijing SH Biotechnology Co., Ltd., Beijing 100070, China, with a cell preparation workshop that meets the GMP production conditions. The preparation process of human dental pulp mesenchymal stem cell used in the study, such as isolation, culture, characterization, quality control, etc., was carried out under the guidance of the corresponding standard operation procedures (SOPs), and the three batches of MSCs used in the study with batch numbers 01012012110205D, 01012012110701D, 01012012111304D have passed strict self-inspection by the company and third-party inspection by the China Academy of Food and Drug Administration

	(CAFDA), which are in line with the quality standard of clinical-grade stem cells, and provide safety assurance for the subjects as well as the researchers.

No.	Project		Screening /treatment period		Research follow-up period				Long-term follow-up
			Screening period	Treatment period	Follow-up visit 1	Follow-up visit 2	Follow-up visit 3	Follow-up visit 4	Long-term follow-up
			（D-14-0）	（D1）	（D7±1），Telephone	（D30±3）	（D90±3）	（D180±5）	（D360±5）
1	Sign ICF		×						
2	Inclusion /Exclusion Criteria		×	×					
3	Demographic information		×						
4	Medical history information		×						
5	Vital signs: respiration, heart rate, blood pressure (systolic, diastolic), temperature (axillary), clinical condition assessment		×	× (Before / 2h±30 min after injection)		× (Double DPSCs injection group 2h±30min after injection)			
6	Periodontal clinical indicators: AL, PD, BOP, GR, TM		×	×			×	×	×
7	Lab tests	Routine blood tests	×	×		×	×	×	
		Coagulation	×	×					
		Liver and kidney function	×	×		×	×	×	
		Ultrasensitive C-reactive protein	×	× (Before / 2h±30 min after injection)		× (Double DPSCs injection group 2h±30min after injection)			
		Infectious Disease Screening: HBsAg, HBsAb, HBeAg, HBeAb, HBcAb, Anti-HCV, HIVcombin, Anti-TP, CMV-IgM, CMV-IgG.	×			×	×	×	

8. Final trial protocol for IIT study

		**Immunology: IgA, IgG, IgM, total IgE.**	×	× **(Before / 2h±30 min after injection)**		× **(Double DPSCs injection group 2h±30min after injection)**	×	×	×
		**Pregnancy Tests**	×	×		×	×	×	
		**Urinalysis**	×	×					
**8**	**Basic periodontal treatment: Supragingival cleaning, Subgingival scaling, root planning**		×	× **（1/2 Subgingival scraping + root planing）**					
**9**	**Drug delivery therapy**			×		× **（Only phase II Double DPSCs injection group）**			
**10**	**Adverse event records**		×	×	×	× **(Double DPSCs injection group 2h±30min after injection)**	×	×	×
**11**	**Combined medication/treatment discipline**		×	×	×	×	×	×	×
**12**	**Imaging (CBCT)**		×				×	×	×
**13**	**Intraoral photography**			×		×	×	×	×

8. Final trial protocol for IIT study

(I) Research Title
Clinical Study of Allogeneic Human Pulp Stem Cells in the Treatment of Chronic Periodontitis (A randomized, open, controlled clinical study of periodontal basic therapy combined with human pulpal mesenchymal stem cell injections for the treatment of chronic moderate periodontitis).

(II) Objectives of the study
(1) To evaluate the safety and efficacy of human DPSCs injection in the treatment of chronic moderate periodontitis;
(2) To provide a basis for the development of a standardized clinical protocol for the treatment of chronic moderate periodontitis with DPSCs injection.

(III) Basis of the project
Periodontitis is a progressive and destructive disease caused by bacterial infection that occurs around the natural teeth as a supportive tissue and is one of the most common chronic diseases of the oral cavity, with a prevalence of up to 90% in China. It is characterized by soft tissue inflammation, periodontal pocket formation and alveolar bone resorption, resulting in insufficient supportive hard tissues around the teeth, progressively loosening and displacing of the teeth, and eventually loss. Periodontitis has been called the "incurable disease" of dentistry, is not only the first cause of tooth loss among adults in China, but also increases the risk of cardiovascular disease, diabetes and rheumatoid arthritis[1]. The ultimate goal of periodontitis treatment is the regeneration of periodontal tissues (alveolar bone, periodontium, osteoid, and gingiva), and the regeneration of functional periodontal tissues is the focus and difficulty of clinical periodontitis treatment. At present, conventional clinical treatments such as basic periodontal therapy, periodontal surgery (periodontal flap surgery, guided bone regeneration, guided tissue regeneration) are difficult to achieve satisfactory tissue regeneration. In recent years, the rapid development of stem cells and tissue engineering has provided an opportunity for periodontal regeneration, the rapid development of stem cells and tissue engineering has provided new ideas for periodontal regeneration [2], among which mesenchymal stem cells (MSCs) are widely available and easy to culture and expand in vitro. MSC can not only secrete a variety of bioactive molecules, including immunomodulation, anti-apoptosis, anti-inflammation, anti-fibrosis, and pro-angiogenesis, but also chemotaxis to tissue trauma and inflammation sites, these characteristics make MSC widely used in regenerative medicine research, as of July 2019, as many as 972 clinical therapeutic studies of MSC have been conducted worldwide [3-5], and there are clinical trials registered for bone marrow mesenchymal stem cells and oral mucosal stem cells for periodontal tissue regeneration research and periodontal disease treatment research (https://www.clinicaltrials.gov/). In China, many hospitals have started MSC treatment for immune disorders and degenerative and injurious diseases. These studies have demonstrated a very good safety profile in the application of MSC for the treatment of localized tissue lesions or systemic diseases.
This project has developed DPSCs injection which can regenerate periodontal tissue and form new periodontal attachments for periodontitis treatment, clinical studies have shown that DPSCs injection are effective in repairing periodontal defects based on their multidirectional differentiation and immunomodulatory abilities. We retrospectively analyzed the data of 18 subjects from two non-registered clinical trials. 18 subjects with chronic moderate periodontitis who were enrolled in two non-registered clinical trials and completed basic periodontal scaling combined with a single topical cellular injection, of which 18 subjects were analyzed for safety and 14 subjects with follow-up data were included in the effectiveness analysis. The results of the study showed:

(1) No serious adverse events or adverse reactions occurred in the 18 subjects during the trial period, and no subjects withdrew from the trial because of adverse events or adverse reactions. The subjects received 1×10^7-8×10^7 cell doses of DPSCs injection locally after basic periodontal treatment, no adverse reactions were observed.
(2) In a randomized controlled study of 10 cases, the periodontal soft tissue recovery in the basic periodontal therapy combined with DPSCs injection group was better than saline group. In the saline group, 2/5 subjects had mild redness, swelling of the gums and multiple bleeding on probing on the 90th day after treatment, while in the DPSCs injection group, 5/5 subjects had no further gingival redness, swelling, or bleeding on probing until the end of the follow-up period (18/24 months).
(3) No tumor development was observed in any of the 18 subjects, including: no tumor development was observed in the 8 open study subjects who were followed up by telephone 48 months after administration of the drug; and no tumorigenic risk was observed in the 10 randomized controlled study subjects who were tested for blood tumor markers at 18/24 months, no tumorigenic risk of DPSCs injection.
(4) An analysis of the effectiveness of the 14 subjects showed that the clinical efficacy of periodontal basic therapy + DPSCs injection group was superior to that of periodontal basic therapy + untreated / saline injection group: ① the patients' periodontal attachment level AL improved, and compared with saline injection group, there was a significant improvement in the AL values after 3 months of DPSCs injection. ②the periodontal probing depth PD of the patients improved in both group, and DPSCs injection group showed significant improvement in PD values at 3 and 6 months after treatment compared with saline injection group.

Therefore, this project proposes to further evaluate the safety and effectiveness of DPSCs injection in the treatment of chronic moderate periodontitis, and to provide a basis for the development of clinical standardized protocols for the treatment of chronic moderate periodontitis with human dental pulp mesenchymal stem cells.

(IV) Expected results

After basic periodontal treatment combined with hDP-MSC localized injection treatment, local periodontal inflammation was relieved or disappeared, and periodontal tissues were regenerated to a certain extent.

1. Periodontal observation

1-12 months after hDP-MSC localized injection treatment, compared with pre-treatment and basic periodontal treatment only, the redness and swelling of gingiva in the periodontitis area disappeared or receded, bleeding of gingiva disappeared or decreased, and the looseness of teeth was improved.

2. Clinical indicators

3-12 months after hDP-MSC localized injection treatment, compared with pre-treatment and basic periodontal treatment only, clinical examination showed that the AL, PD and BOP index at the periodontitis site were reduced.

3. Imaging examination

3-12 months after hDP-MSC localized injection treatment, compared with pre-treatment and basic periodontal treatment only, oral CBCT examination can see the hard tissue proliferation in the periodontal area of the injected teeth and the alveolar bone edge, or the volume of the periodontal hard tissue defect reduced.

(V) Study design

1. This study is a single-center, randomized, controlled study of basic periodontal basic treatment combined with hDP-MSC localized injection, the sample size was 96 cases.

2. The study conducted two phases: the first phase consisted two groups randomized according to a 1:1 ratio; the second phase consisted of three groups randomized according to a 1:1:3 ratio.

2.1 Randomization method: The randomization method for each stage of the study was district group randomization, and the statistician used SAS 9.4 software to generate random group codes. In phase 1, subjects were assigned to the DPSCs injection group and saline injection group, randomization envelopes were retrieved by the SAS unit upon completion of this phase; Phase II subjects were assigned to the single DPSCs injection group, double DPSCs injection group and the saline injection control group in the ratio of 1:1:3 according to the code of this phase.

2.2 Inclusion Criteria: Subjects must meet all of the following criteria to be included:

(1) Age 18-65 years old, gender is not limited;
(2) Voluntarily participate in this clinical study and sign the informed consent form;
(3) Radiologic detection of wedge-shaped bone defects at the periodontal defect sites;
(4) The probing depth (PD) of the periodontal defect site was 4-8 mm.

2.3 Exclusion Criteria: subjects meeting any of the following will be excluded:

(1) Hypertensive subjects with blood pressure: systolic blood pressure ≥180 mmHg or diastolic blood pressure ≥110 mmHg within 1 month prior to surgery;
(2) Systemic diseases (subjects with malignant tumors or those who tested positive for tumors during the screening process, diabetic subjects, cardiac diseases resulting in heart failure, and myocardial infarction within six months; angina symptoms within six months; congenital heart disease);
(3) Subjects have used non-steroidal anti-inflammatory drugs, steroidal hormone therapy within three months prior to surgery, and/or those who have used hormones for a long period of time, those who have used bisphosphonates;
(4) Systemic infections;
(5) Subjects have undergone surgical treatment of periodontal defect sites and adjacent periodontal tissues;
(6) Subjects who are known to be allergic to any materials used in the surgical procedure; (allergy, history of allergy to blood products;)
(7) Severe periodontitis(alveolar bone resorption exceeding 2/3 of the length of root);
(8) Subjects with severe hepatic and renal insufficiency;
(9) Subjects with bleeding tendency or coagulation disorders (WBC<3.0×10^9 /L or PLT <60×10^9 /L);
(10) HIV or hepatitis B virus infection;
(11) Subjects had unprotected sex within one month prior to study screening;
(12) Women who are pregnant, lactating, or applying estrogen for contraception;
(13) Subjects whose own or spouses plan to become pregnant during the study and within 6 months of study completion;
(14) Smokers (currently >10 cigarettes/day);
(15) Subjects with an expected survival of less than 12 months;
(16) Other circumstances deemed inappropriate for participation by the investigator.

2.4 Withdrawal/Discharge Criteria:

(1) Subject has experienced adverse event during the course of the study that, in the opinion of the investigator, precludes continuation of the study;
(2) Subjects have poor compliance and are unable to complete follow-up visits on time;
(3) Use of other medications that affect tolerability or safety judgment, such as glucocorticoids, bisphosphonates, estrogens;
(4) Use of other foods that affect tolerability or safety judgment, such as betel nut, or

tough foods that require prolonged chewing, resulting in bite force burden, such as beefsteak;

(5) Subjects who wish to withdraw from the clinical study and request to investigator;
(6) Female subjects become pregnant during the study;
(7) Drop-out.

2.5 Subgroup design: The study conducted 2 phases, 96 subjects were recruited.

In phase I, there were 2 groups and 46 subjects were enrolled. After the subjects with a clinical diagnosis of chronic moderate periodontitis signed an I informed consent form and met the inclusion criteria while did not meet the exclusion criteria, the investigator randomly assigned them to DPSCs injection group, approximately 23 subjects; saline injetion group, approximately 23 subjects, based on the randomization grouping codes provided by the statistician. The randomization envelopes were collected by the Numerical Statistics Unit upon completion of this phase. The investigator selected the proposed treatment sites for the subjects (individual tooth site/subject). All subjects were treated with periodontal basic therapy and perform bilateral multi-point inject hDP-MSC/saline injection immediately after the completion of periodontal basic therapy.

In phase II, there were 3 groups and 50 subjects. After the subjects with a clinical diagnosis of chronic moderate periodontitis signed an informed consent form and met the inclusion criteria while did not meet the exclusion criteria, the investigator randomly assigned them to saline injection group, approximately 30 subjects; Single DPSCs injection group, approximately 10 subjects; Double DPSCs injection group, approximately 10 subjects, based on the randomization grouping codes provided by the statistician. The randomization envelopes were collected by the Numerical Statistics Unit upon completion of this phase. The investigator selected the proposed treatment sites for the subjects (individual tooth site/subject). All subjects were treated with periodontal basic therapy and perform bilateral multi-point inject hDP-MSC/saline immediately after the completion of periodontal basic therapy. At the end of the one-month follow-up, double DPSCs injection group received a bilateral multi-point inject hDP-MSC/saline.

In summary, the two phases of this study, saline group is expected to enroll approximately 33 subjects, single DPSCs injection group is expected to enroll approximately 33 subjects, and double DPSCs injection group is expected to enroll 30 subjects, which meets the requirements of statistical analysis.

3. blinding setup:

This was an open study and blinded to periodontal data collectors and imaging data measurers. Since the study used DPSCs preparation had the appearance of pale creamy white homogeneous suspension after re-mixing before use, and the saline had appearance of a colorless appearance, it was impossible to blind the subjects and the investigators of local injection manipulation.

4. Study steps:

(1) Screening Period (D-14~0):

【Signed Informed Consent Form】： The investigator fully informs the subjects about the details of the study, the treatment options for chronic moderate periodontitis, possible risks and benefits. After obtaining a written, voluntarily signed informed consent form from the subject, the subject was included in the subjects screening.

【Inclusion/exclusion criteria】： Screening of subjects who meet the inclusion criteria and do not meet the exclusion criteria.

【Demographic information】： Demographic information of the subjects will be collected and recorded, including gender, date of birth, ethnicity, height, and weight.

【**Medical history**】： Collect and record the subject's current medical history, allergy history, family history, antecedent history (smoking, diabetes, other surgical history, etc.).
【**Vital signs**】：respiration, heart rate, blood pressure (systolic and diastolic), temperature (axillary), and clinical assessment of general condition.
【**Periodontal Clinical Indicators**】： Periodontal examination was standardized using the Florida Probe of the Beijing Stomatological Hospital, Capital Medical University. Records: periodontal attachment level AL, periodontal probing depth PD, probing bleeding index BOP, gingival recession GR, tooth mobility TM, and the proposed treatment site (single tooth/subject) were selected by the investigator.
【**Lab Tests**】：

☞Blood routine: leukocytes, erythrocytes, hemoglobin, erythrocyte pressure product, mean erythrocyte volume, mean hemoglobin volume, mean hemoglobin concentration, platelet count, mean platelet volume, platelet pressure product, neutrophils, lymphocytes, monocytes, eosinophils, basophils, neutrophil absolute value, lymphocyte absolute value, monocyte absolute value, eosinophil absolute value, basophil absolute value, erythrocyte distribution width SD, erythrocyte distribution width CV, large platelet ratio, platelet distribution width.

☞Hemorrhagic and coagulation function: plasma prothrombin time, international normalized ratio of prothrombin time, activated partial thromboplastin time, prothrombin time, plasma fibrinogen.

☞Liver and kidney function: alanine aminotransferase, aspartate aminotransferase, total protein, albumin, globulin, albumin-globulin ratio, total bilirubin, direct bilirubin, indirect bilirubin, alkaline phosphatase, creatinine, γ-glutamine acyltransferase, lactate dehydrogenase, creatine kinase, urea, uric acid, total carbon dioxide, glucose.

☞Inflammatory markers: ultrasensitive C-reactive protein.

☞Infectious disease tests: HBsAg, HBsAb, HBeAg, HBeAb, HBcAb, Anti-HCV, HIVcombin, Anti-TP, CMV-IgM, CMV-IgG.

☞Immunologic tests: IgA, IgG, IgM, total IgE.

☞Pregnancy test: blood beta-HCG, premenopausal female subjects only.

☞Urine routine: pH, specific gravity, protein, ketone bodies, bilirubin, urobilinogen, nitrites, leukocytes, erythrocytes, urine sugar, urine sediment microscopy.

【**Basic periodontal treatment**】：All groups of subjects underwent ultrasonic supragingival cleaning, subgingival scraping of half of the mouth and root planing. Periodontal irritants such as plaque were removed. Periodontal basic treatment required the preservation of residual dentin on the root surface according to basic principles. The investigators recorded the start and end times of the subjects' periodontal basic treatments.

■ Supragingival cleaning (D-14): saline rinsing, full-mouth ultrasonic supragingival cleaning and polishing, and saline rinsing;

■ Subgingival scaling (D-7±1, non-treated tooth site area): 2% Articaine Hydrochloride anesthesia, half-mouth ultrasonic subgingival scaling, root planning, polishing and physiological saline rinse.

【**Imaging**】： CBCT examination. The investigator re-established the treatment site of the subject (single tooth/subject).
【**Adverse Event Record**】
【**Record of combined medications/treatments**】
【**Intraoral photography**】：Photographs were taken to record the condition of the oral cavity and treatment sites.
【**Randomized grouping**】：Before D-2, for subjects who met the enrollment criteria, the investigators gave subjects a randomized grouping number based on the randomized grouping

code provided by the statistician to determine the group of subjects and to select the proposed treatment site.

(2) Baseline/treatment period (D1):

【Inclusion/Exclusion Criteria】： For subjects who met the enrollment criteria, the investigators informed the subjects of the treatment tooth position information.

【Vital signs】： respiration, heart rate, blood pressure (systolic and diastolic), temperature (axillary), and clinical assessment of general condition.

【Periodontal Clinical Indicators】： Periodontal examination was standardized using the Florida Probe of the Beijing Stomatological Hospital, Capital Medical University. Records: periodontal attachment level AL, periodontal probing depth PD, probing bleeding index BOP, gingival recession GR, tooth mobility TM, and the proposed treatment site (single tooth/subject) were selected by the investigator.

【Lab Tests】：

☞Blood routine: leukocytes, erythrocytes, hemoglobin, erythrocyte pressure product, mean erythrocyte volume, mean hemoglobin volume, mean hemoglobin concentration, platelet count, mean platelet volume, platelet pressure product, neutrophils, lymphocytes, monocytes, eosinophils, basophils, neutrophil absolute value, lymphocyte absolute value, monocyte absolute value, eosinophil absolute value, basophil absolute value, erythrocyte distribution width SD, erythrocyte distribution width CV, large platelet ratio, platelet distribution width.

☞Hemorrhagic and coagulation function: plasma prothrombin time, international normalized ratio of prothrombin time, activated partial thromboplastin time, prothrombin time, plasma fibrinogen.

☞Liver and kidney function: alanine aminotransferase, aspartate aminotransferase, total protein, albumin, globulin, albumin-globulin ratio, total bilirubin, direct bilirubin, indirect bilirubin, alkaline phosphatase, creatinine, γ-glutamine acyltransferase, lactate dehydrogenase, creatine kinase, urea, uric acid, total carbon dioxide, glucose.

☞Inflammatory markers: ultrasensitive C-reactive protein.

☞Infectious disease tests: HBsAg, HBsAb, HBeAg, HBeAb, HBcAb, Anti-HCV, HIVcombin, Anti-TP, CMV-IgM, CMV-IgG.

☞Immunologic tests: IgA, IgG, IgM, total IgE.

☞Pregnancy test: blood beta-HCG, premenopausal female subjects only.

☞Urine routine: pH, specific gravity, protein, ketone bodies, bilirubin, urobilinogen, nitrites, leukocytes, erythrocytes, urine sugar, urine sediment microscopy.

【Basic periodontal treatment】： All groups of subjects underwent ultrasonic supragingival cleaning, subgingival scraping of half of the mouth and root planing. Periodontal irritants such as plaque were removed. Periodontal basic treatment required the preservation of residual dentin on the root surface according to basic principles. The investigators recorded the start and end times of the subjects' periodontal basic treatments.

■ Supragingival cleaning (D-1, included therapeutic tooth area): 2% Articaine Hydrochloride anesthesia, half-mouth ultrasonic subgingival scaling, root planning, polishing and physiological saline rinse.

【Local injection treatment】： The investigator, according to the randomized assignment number, were treated with periodontal basic therapy and perform bilateral multi-point inject DPSCs/saline injection immediately after the completion of periodontal basic therapy.

The injection method was as follows: under local anesthesia, a periodontal probe was used to determine the position of the top of the alveolar ridge, and the needle was inserted into the bottom of the periodontal pocket (in the tissue around the bottom of the periodontal pocket). After the needle reaches the periodontium on the inner side of the alveolar ridge and touches

the junction of bone surface and root, a 1 ml screw-port syringe was used for buccal (labial), lingual (palate) injection with the inject time of 20-30 seconds per point. Stopping point of the injection needle is located on the root surface to ensure that the drug can be concentrated in the root area.

■ DPSCs injection group: a single treatment dose of 0.6 mL / tooth, cell volume of about 1 $\times 10^7$ hDP-MSC cells / tooth / person, suspension of saline 0.6 mL / tooth.

■ Saline injection group: a single treatment dose of 0.6 mL /tooth.

The investigator recorded the start and end time of the subjects' local injection treatments.

【Vital signs】： Checked 2h±30min after the end of local injection treatment. Record: respiration, heart rate, blood pressure (systolic, diastolic), temperature (axillary), and clinical assessment of general condition.

【Laboratory tests】： 2h±30min after the end of local injection treatment.

☞ Inflammation index: ultrasensitive C-reactive protein.

☞Immunologic tests: IgA, IgG, IgM, total IgE.

【Record of adverse events】： All subjects in all groups were observed for 2 hours after treatment and left the hospital, during which adverse reactions were recorded.

【Record of combined medication/treatment】

【Intraoral photography】： Photographs were taken to record the condition of the oral cavity and treatment sites.

(3) Post-treatment follow-up 1: Telephone follow-up 1 week (D7±1 day) after the end of treatment.

【Adverse event record】

【Record of combined medication/treatment】

(4) Post-treatment follow-up visit 2: One month (D30±3 days) after the end of treatment.

【Vital signs】： respiration, heart rate, blood pressure (systolic and diastolic), temperature (axillary), and clinical assessment of general condition.

【Lab Tests】：

☞Blood routine: leukocytes, erythrocytes, hemoglobin, erythrocyte pressure product, mean erythrocyte volume, mean hemoglobin volume, mean hemoglobin concentration, platelet count, mean platelet volume, platelet pressure product, neutrophils, lymphocytes, monocytes, eosinophils, basophils, neutrophil absolute value, lymphocyte absolute value, monocyte absolute value, eosinophil absolute value, basophil absolute value, erythrocyte distribution width SD, erythrocyte distribution width CV, large platelet ratio, platelet distribution width.

☞Hemorrhagic and coagulation function: plasma prothrombin time, international normalized ratio of prothrombin time, activated partial thromboplastin time, prothrombin time, plasma fibrinogen.

☞Liver and kidney function: alanine aminotransferase, aspartate aminotransferase, total protein, albumin, globulin, albumin-globulin ratio, total bilirubin, direct bilirubin, indirect bilirubin, alkaline phosphatase, creatinine, γ-glutamine acyltransferase, lactate dehydrogenase, creatine kinase, urea, uric acid, total carbon dioxide, glucose.

☞Inflammatory markers: ultrasensitive C-reactive protein.

☞Infectious disease tests: HBsAg, HBsAb, HBeAg, HBeAb, HBcAb, Anti-HCV, HIVcombin, Anti-TP, CMV-IgM, CMV-IgG.

☞Immunologic tests: IgA, IgG, IgM, total IgE.

☞Pregnancy test: blood beta-HCG, premenopausal female subjects only.

☞Inflammation indicator: ultrasensitive C-reactive protein, only for subjects in the double DPSCs injection group of Phase II of this study

【Adverse Event Record】

【Record of combined medications/treatments】
【Double DPSCs Injection Treatment】: only for subjects in the double DPSCs injection group in Phase II of this study. At the end of the one-month follow-up visit, subjects received a bilateral multi-point inject hDP-MSC. Periodontal defect sites were injected in the same manner as the first local injection treatment.

■ Double DPSCs injection group: at the end of the one-month follow-up visit, the subjects were given bilateral multipoint injections into individual tooth sites at a single treatment dose of about 1×10^7 hDP-MSC (saline 0.6mL suspension) / tooth / person.
The investigators recorded the start and end time of the subjects' local injection treatments.
【Vital Signs】:only for subjects in the Phase II double DPSCs injection group of this study. 2h±30min after the end of local injection treatment, record: respiration, heart rate, blood pressure (systolic and diastolic), temperature (axillary), and clinical assessment of general condition.
【Laboratory tests】: Only for subjects in the Phase II S double DPSCs injection group of this study. After the local injection treatment 2h±30min after the end of local injection treatment.

☞Inflammation indicator: Ultrasensitive C-reactive protein.

☞Immunologic tests: IgA, IgG, IgM, total IgE.

【Adverse event record】: Only for subjects in the Phase II Stem Cell Secondary Dosing Group of this study, who were observed 2 hours after treatmen before left the hospital and adverse events were record.

(5) **Post-treatment follow-up 3:** 3 months (D90±3 days) after the end of treatment.
【Periodontal Clinical Indicators】: Periodontal examination was standardized using the Florida Probe of the Beijing Stomatological Hospital, Capital Medical University. Records: periodontal attachment level AL, periodontal probing depth PD, probing bleeding index BOP, gingival recession GR, tooth mobility TM, and the proposed treatment site (single tooth/subject) were selected by the investigator.
【Lab Tests】:

☞Blood routine: leukocytes, erythrocytes, hemoglobin, erythrocyte pressure product, mean erythrocyte volume, mean hemoglobin volume, mean hemoglobin concentration, platelet count, mean platelet volume, platelet pressure product, neutrophils, lymphocytes, monocytes, eosinophils, basophils, neutrophil absolute value, lymphocyte absolute value, monocyte absolute value, eosinophil absolute value, basophil absolute value, erythrocyte distribution width SD, erythrocyte distribution width CV, large platelet ratio, platelet distribution width.

☞Hemorrhagic and coagulation function: plasma prothrombin time, international normalized ratio of prothrombin time, activated partial thromboplastin time, prothrombin time, plasma fibrinogen.

☞Liver and kidney function: alanine aminotransferase, aspartate aminotransferase, total protein, albumin, globulin, albumin-globulin ratio, total bilirubin, direct bilirubin, indirect bilirubin, alkaline phosphatase, creatinine, γ-glutamine acyltransferase, lactate dehydrogenase, creatine kinase, urea, uric acid, total carbon dioxide, glucose.

☞Inflammatory markers: ultrasensitive C-reactive protein.

☞Infectious disease tests: HBsAg, HBsAb, HBeAg, HBeAb, HBcAb, Anti-HCV, HIVcombin, Anti-TP, CMV-IgM, CMV-IgG.

☞Immunologic tests: IgA, IgG, IgM, total IgE.

☞Pregnancy test: blood beta-HCG, premenopausal female subjects only.

【Adverse Event Record】
【Record of combined medications/treatments】

【Imaging】： CBCT examination.
【Intraoral photography】： Photographs were taken to record the condition of the oral cavity and treatment sites.
(6) **Post-treatment follow-up 4:** 6 months (D180±5 days) after the end of treatment.
【Periodontal Clinical Indicators】： Periodontal examination was standardized using the Florida Probe of the Beijing Stomatological Hospital, Capital Medical University. Records: periodontal attachment level AL, periodontal probing depth PD, probing bleeding index BOP, gingival recession GR, tooth mobility TM, and the proposed treatment site (single tooth/subject) were selected by the investigator.
【Lab Tests】：

☞Blood routine: leukocytes, erythrocytes, hemoglobin, erythrocyte pressure product, mean erythrocyte volume, mean hemoglobin volume, mean hemoglobin concentration, platelet count, mean platelet volume, platelet pressure product, neutrophils, lymphocytes, monocytes, eosinophils, basophils, neutrophil absolute value, lymphocyte absolute value, monocyte absolute value, eosinophil absolute value, basophil absolute value, erythrocyte distribution width SD, erythrocyte distribution width CV, large platelet ratio, platelet distribution width.

☞Hemorrhagic and coagulation function: plasma prothrombin time, international normalized ratio of prothrombin time, activated partial thromboplastin time, prothrombin time, plasma fibrinogen.

☞Liver and kidney function: alanine aminotransferase, aspartate aminotransferase, total protein, albumin, globulin, albumin-globulin ratio, total bilirubin, direct bilirubin, indirect bilirubin, alkaline phosphatase, creatinine, γ-glutamine acyltransferase, lactate dehydrogenase, creatine kinase, urea, uric acid, total carbon dioxide, glucose.

☞Inflammatory markers: ultrasensitive C-reactive protein.

☞Infectious disease tests: HBsAg, HBsAb, HBeAg, HBeAb, HBcAb, Anti-HCV, HIVcombin, Anti-TP, CMV-IgM, CMV-IgG.

☞Immunologic tests: IgA, IgG, IgM, total IgE.

☞Pregnancy test: blood beta-HCG, premenopausal female subjects only.

【Adverse Event Record】
【Record of combined medications/treatments】
【Imaging】： CBCT examination.
【Intraoral photography】： Photographs were taken to record the condition of the oral cavity and treatment sites.
(7) **Long-term follow-up:** 12 months (D360±5 days) after the end of treatment.
【Periodontal Clinical Indicators】： Periodontal examination was standardized using the Florida Probe of the Beijing Stomatological Hospital, Capital Medical University. Records: periodontal attachment level AL, periodontal probing depth PD, probing bleeding index BOP, gingival recession GR, tooth mobility TM, and the proposed treatment site (single tooth/subject) were selected by the investigator.
【Lab Tests】：

☞Immunologic tests: IgA, IgG, IgM, total IgE.

【Adverse Event Record】
【Record of combined medications/treatments】
【Imaging】： CBCT examination.
【Intraoral photography】： Photographs were taken to record the condition of the oral cavity and treatment sites.

(6) Research implementation conditions

1. Research personnel qualifications:

The main researchers of this study are Professor Songling Wang and Professor Yi Liu. Prof. Songling Wang serves as the principal investigator. He is currently the vice director of the Beijing Stomatological Hospital Tissue Regeneration and Functional Reconstruction Research Laboratory, and is a professor and chief physician. Prof. Wang has long been dedicated to translational research in dental and periodontal tissue regeneration and functional reconstruction. He has developed novel drugs using dental pulp stem cells and successfully used allogeneic stem cells to regenerate biological tooth roots for repairing tooth loss and treating chronic periodontitis. He has also studied the molecular mechanism of epithelial-mesenchymal interaction during tooth development. Prof. Wang has received research funding from the Beijing Municipal Government Beijing Scholars Program and the National 973 Program. He has published 85 SCI papers as corresponding author or first author in journals such as Stem Cells Dev, BMC Genomics, Oncotarget, and J Dent Res, including 46 articles with an impact factor greater than 3. Prof. Yi Liu serves as the quality assurance investigator. She currently serves as the director of the Periodontology Department. Prof. Liu is an expert in the diagnosis and treatment of periodontal diseases, periodontal soft tissue surgery, guided tissue regeneration surgery, and dental implantation. She has long been dedicated to research on promoting oral and maxillofacial tissue regeneration by regulating the host microenvironment, and has achieved original results in the regulation mechanism of the host microenvironment on oral and maxillofacial bone regeneration and its application. She has published over 40 SCI papers in renowned journals such as Nat Med, Cell Stem Cell, Cell Res, and Stem Cells and applied for 2 patents.

Other researchers includes, Jingchao Hu, an associate chief physician in the Department of Periodontics, with expertise in the diagnosis and treatment of periodontitis, periodontal flap surgery, guided tissue regeneration, mucogingival surgery, management of peri-implant soft and hard tissues, and periodontal treatment of implant-associated peri-implantitis; Yu Cao, the chief physician in the Emergency Comprehensive Diagnosis and Treatment Center, specializing in the diagnosis and treatment of dental pulp and periodontal diseases; Luyuan Jin, the associate chief physician in the Emergency Comprehensive Diagnosis and Treatment Center, specializing in the diagnosis and treatment of periodontal diseases and oral and maxillofacial surgical diseases; Jin Ji, an associate chief physician in the Department of Periodontics, with extensive experience in the diagnosis and treatment of periodontal diseases and interdisciplinary treatment, expert in mucogingival surgery, implant surgery, and periodontal attachment widening surgery in adjunct to orthodontic treatment; Minfeng Wang, an associate chief physician in the Department of Periodontics, with expertise in systemic treatment of periodontal diseases and reconstruction surgery of periodontal soft and hard tissues; Lin Song, an associate chief physician in the Department of Periodontics, specializing in the diagnosis and treatment of periodontitis, guided tissue regeneration, and other periodontal surgical treatments.

The researchers involved in this study all have many years of clinical and research experience. They have scheduled clinical practice days and research days.Additionally, they have all completed training on GCP for drug clinical trials and obtained the corresponding certificates. Furthermore, Songling Wang has participated in training courses on quality management of stem cell preparation and cell bank quality management self-inspection, and has

obtained the training certificate. Yi Liu and Jingchao Hu have participated in training courses on the registration of stem cell clinical research projects and also obtained the training certificate.

2. Research institutions and facilities:

This study recruited subjects from the Department of Periodontics and the Emergency Comprehensive Diagnosis and Treatment Center. There are an ample number of patients with chronic moderate periodontitis available, ensuring an adequate number of eligible subjects who meet the inclusion criteria and do not meet the exclusion criteria can be enrolled. The Department of Periodontics and the Emergency Comprehensive Diagnosis and Treatment Center together have 48 dental chairs, which can provide independent treatment rooms and relatively independent chairs for pre-enrollment discussions with subjects. Additionally, there are enough independent chairs available for post-enrollment oral clinical parameter assessments, periodontal basic treatments, and local injection of hDP-MSC cells.

The release test of the dental pulp mesenchymal stem cell product used in this study conducted by Stomatological Hospital. The items in the release test include: cell count, cell viability. The equipments needed are inverted microscope, sodium chloride injection and trypan blue staining solution. The Stomatological Hospital meets the conditions for release test of dental pulp mesenchymal stem cell product.

After the human dental pulp mesenchymal stem cell preparation is received by the researcher responsible for the management and distribution of stem cell products at the research institution, it will be stored in a portable programmable incubator provided by the preparation institution. The storage conditions require a temperature of 4℃, and the storage time is limited to 10 hours after the preparation of the product. Once the local injection treatment with the stem cell product is completed, the temperature record of the transportation and storage process for the stem cell product will be printed and archived.

The laboratory tests included in the safety assessment indicators of this study will be carried out by the laboratory department. Some of the test items will require blood samples to be sent to an external testing facility for examination. These items include high-sensitivity C-reactive protein, infectious disease testing (cytomegalovirus CMV-IgM, cytomegalovirus CMV-IgG), immunological testing (IgA, IgG, IgM, total IgE), and pregnancy tests. The testing will be conducted after a trilateral agreement is signed between the research institution, the preparation institution, and the external testing facility. The external testing facility is Beijing Edicon Medical Laboratory Co., Ltd., which has obtained ISO 15189 accreditation, CMA metrology certification, and CLIA certification from the CAP, known as the "gold standard" for clinical laboratories. After the completion of the external blood sample testing for this study, Beijing Edicon Medical Laboratory Co., Ltd. will be responsible for the uniform destruction of the samples and will provide periodic destruction certificates to the research institution.

The imaging tests included in the efficacy assessment indicators of this study will be conducted using CBCT measurement, which will be carried out by the radiology department.

（7） Participant inclusion and exclusion criteria and group assignment method

1. Inclusion criteria: In order to be included in this study, subjects must meet all of the following criteria:

（1）Subjects must be between the ages of 18 and 65 years old, and there are no gender restrictions for inclusion in this study;

（2）Subjects must voluntarily participate in this clinical study and sign an "Informed Consent Form";

（3）Radiographic examination of the periodontal defect site reveals a wedge-shaped bone defect;

（4）PD at the periodontal defect site ranges from 4 to 8mm.

2. Exclusion criteria: Subjects who meet any of the following criteria will be excluded from this study:

（1）Subjects with hypertension who have blood pressure readings of systolic blood pressure ≥180mmHg or diastolic blood pressure ≥110mmHg within one month prior to the surgery will be excluded from this study;

（2）Subjects with systemic diseases such as malignancy (including those with positive tumor screening results), diabetes, heart failure due to heart disease, myocardial infarction within the past six months, angina symptoms within the past six months, or congenital heart disease will be excluded from this study;

（3）Subjects who have used NSAIDs or corticosteroids within three months prior to the surgery, or those who have been on long-term steroid therapy, or have used bisphosphonate medications, will be excluded from this study;

（4）Systemic infectious patients;

（5）Patients with periodontal defects and adjacent periodontal tissues that have received surgical treatment;

（6）Patients with known allergies to any materials that may be used during the surgical procedure (such as patients with allergy prone constitution or history of allergy to blood products);

（7）Patients with severe periodontitis (alveolar bone resorption exceeding two-thirds of the root length);

（8）Patients with severe liver or kidney dysfunction;

（9）Patients with bleeding tendencies or coagulation disorders (patients with WBC < 3.0 × 109/L or PLT < 60 × 109/L);

（10）Patients infected with HIV or hepatitis B virus;

（11）Patients who had unprotected sexual activity within one month prior to screening;

（12）Pregnant or lactating females or females using female hormone contraceptives;

（13）Patients who plan to become pregnant themselves or whose partner plans to become pregnant during the study period or within 6 months after study completion;

（14）Smokers (currently smoking more than 10 cigarettes per day);

（15）Patients with an expected survival period of less than 12 months;

（16）Patients deemed unsuitable for participation by the investigators for any other reasons.

3. Exit/withdrawal criteria：

（1） Adverse events occurring during the study period that, according to the investigators, make it impossible to continue with the study;

（2） Poor compliance of subject with failure to complete follow-ups in a timely manner;

（3） Use of medications affecting tolerability or safety assessments, such as glucocorticoids, bisphosphonates, estrogens;

（4） Subjects who consume other substances that may affect tolerance or safety, such as betel nut, or regularly chew tough foods that exert excessive biting forces, such as beef tendon, will be excluded from this study;

（5） Subject's voluntary withdrawal from the clinical study by expressing their unwillingness to continue to participate to the researcher;

（6） If a female subject becomes pregnant during the course of the study, she will be discontinued from further participation;

（7） Attrition.

4.Translation Assignment Grouping Method: In this study, a block randomization method was used at each stage, and the randomization was generated by a statistician using SAS 9.4 software. The randomization and grouping coding were implemented in this study. In the first stage, subjects were assigned to either the stem cell treatment group or the saline control group in a 1:1 ratio based on the assigned coding. After completion of this stage, the randomization envelopes were collected by the statistical unit. In the second stage, subjects were assigned to the saline control group, stem cell treatment group, or stem cell re-administration group in a 1:1:1 ratio based on the assigned coding. The statisticians were not involved in the screening, inclusion of subjects, or subsequent clinical research. Throughout the study, the researchers responsible for the safety and efficacy data analysis maintained blinding.

(8) Required number of cases

A total of 96 subjects were recruited for this study, divided into three groups: the saline control group, the stem cell treatment group, and the stem cell re-administration group.

The sample size calculation was performed using PASS 16 software. The significance level was set at a=0.05, and the power of the test was set at 1-b=0.80. The research data from the two stages were combined for statistical analysis. Assuming roughly equal sample sizes in the three groups, the sample size calculation was conducted according to the following formula

$$2\left[\frac{(t_{1-\alpha/2}+t_{1-\beta})\ S}{\delta}\right]^2$$

In the formula, S represents the standard deviation, δ represents the effect size (i.e., the difference between the means of the two groups), and n represents the required number of cases per group.

In the first stage of this study, there are two groups, and it is expected to include 46 subjects. They will be randomly assigned in approximately a 1:1 ratio to the stem cell treatment group and the saline control group. In the second stage, there are three groups, and it is expected to include 50 subjects. They will be randomly assigned in a 1:1:3 ratio to the saline control group, the stem cell treatment group, and the stem cell re-administration group.

Based on the information provided, for the two stages of this study, the estimated number of subjects to be included in the saline control group is approximately 33, the estimated number of subjects in the stem cell treatment group is approximately 33, and the estimated number of subjects in the stem cell re-administration group is 30. These numbers fulfill the statistical analysis requirements.

(9) Subject management.

1. **recruitment methods for subjects:** In this study, the recruitment of subjects is conducted during the clinical diagnostic process by Dr. Jingchao Hu, a periodontist, and Dr. Yu Cao, a physician from the Emergency Comprehensive Diagnostic Center.

2. **informed consent process:** The researchers responsible for recruitment will provide a project introduction to patients who express an interest in participating in this study. This will be conducted in a separate treatment room or a relatively private seating area. They will explain the study requirements, administration process, and steps in detail, as well as the potential risks and discomfort that may arise from participation. At the same time, patients will be informed that participating in the study is a voluntary choice, and they have the right to choose whether to participate or explore other treatment options. They can discuss this decision with their family members or friends and also have the option to withdraw from the study at any time. During the pre-enrollment conversation with patients, the researchers should address any questions the patients may have about the study and ensure that patients

have sufficient time to consider their participation.

Once patients agree to participate in this study, they formally become subjects of the study and will co-sign the latest version of the informed consent form approved by the Ethics Committee of Peking University School and Hospital of Stomatology (Beijing Stomatological Hospital). The version number and date of the consent form should be clearly indicated, and both parties should sign their names and include the date. Additionally, the researcher should inform the subjects that if any new important information regarding the stem cell preparation arises during the course of the study, the research team must make written modifications to the informed consent form. These modifications must be approved by the Ethics Committee of Peking University School and Hospital of Stomatology (Beijing Stomatological Hospital), and the informed consent of the subjects must be obtained again.

3. **allocation of screening numbers:** The researchers assign screening numbers to the subjects based on their screening order.

4. **verification of inclusion and exclusion criteria:** The researchers screen subjects who meet the inclusion criteria and do not meet the exclusion criteria. Comprehensive assessments are conducted for the enrolled subjects to verify if they meet all the screening and inclusion criteria.

5. **allocation of treatment/randomization numbers**: Once the researchers confirm that the subjects meet all the inclusion criteria during the screening period, they will allocate randomization numbers to the subjects based on the randomization codes provided by the statisticians. This random allocation determines the group assignment for each subject. During the baseline/treatment period, the researchers will once again confirm that the subjects meet all the inclusion criteria and inform them of their final group assignment for enrollment.

6. **study compliance management:** Once subjects receive their randomization numbers and are assigned to the researchers involved in this study, it becomes the responsibility of the researchers to track and follow up with the subjects. The researchers should diligently adhere to the study protocol and informed consent form, ensuring that the subjects fully understand the study requirements and cooperate with the research. They should also regularly remind the subjects of scheduled follow-up appointments through phone calls.

7. **compensation and reimbursement:** Subjects participating in this study will receive a fee of 300 RMB per visit as compensation for transportation expenses and loss of income. The researchers are responsible for providing this compensation in cash to the subjects at the end of each visit. If the subjects are able to complete all the scheduled visits for the study, they will receive a total of 7 compensation payments, amounting to 2100 RMB per subject.

(10) Subject motivation and compensation

1. **research expenses:** The expenses related to periodontal basic treatment, stem cell infusion treatment, periodontal clinical indicators, laboratory tests, and imaging examinations (CBCT) incurred by the subjects participating in this study will be covered by the matching funds provided by the Beijing Municipal Science and Technology Commission for the special project. Any expenses beyond this will be borne by the formulation preparation institution.

2. **treatment and compensation for research-related injuries:**Research-related injuries refer to damages caused by the execution of research procedures or intervention measures solely for the purpose of the study. Subjects should receive corresponding compensation and/or free medical treatment. Compensation refers to legitimate civil acts where the researcher is not at fault but has caused certain losses to the other party. On the other hand, compensation is provided in cases where the researcher's wrongful actions have caused personal or property damage to the other party. Adverse reactions related to investigational treatments, diagnoses, or expected adverse events that are of the same type, severity, and incidence as those recognized in standard medical practices should be provided

with free medical treatment and may not require compensation for the subjects.

If a subject experiences an adverse event related to the study during the research period, the researcher will provide appropriate medical treatment and report it to the formulation preparation institution. The formulation preparation institution has purchased clinical trial insurance related to stem cell research for this study. In the event of research-related injuries, the formulation preparation institution is responsible for covering the corresponding treatment and compensation expenses for all subjects in both the stem cell treatment group and the saline control group. This responsibility is outlined in a cooperation agreement signed between the formulation preparation institution and the research institution.

(11) Subject privacy and confidentiality

Privacy: This study involves biomedical research involving humans, and during the research process, it is necessary to collect the personal information of the subjects, which includes: information related to personal identity such as name, gender, date of birth, ethnicity, height, weight, telephone number, identification numbers (such as ID cards, social security cards), outpatient numbers, and handwritten signatures; personal health-related information such as personal medical records, including current medical history, allergies, family history, past medical history, etc. The collection of personal information by researchers during the diagnostic and treatment process is in accordance with the duties of a medical professional, and is for the purpose of making correct diagnoses and providing effective treatment for the patients' illnesses. Therefore, the collection of personal information by researchers in this study does not necessarily constitute a violation of the subjects' right to privacy. However, the use of the personal information collected in the study is subject to specific restrictions and requires the consent of the subjects if it goes beyond the scope of application.

1. **Research samples, materials, data:**

During the study, the subject's study sample (e.g., venous blood sample), study materials (all paper and electronic records), and all study data will be kept strictly confidential at all times. Conventional confidentiality measures include: (1) training researchers on the importance of confidentiality; (2) De-identification of samples, research materials and research data to subjects: each subject is given a screening number, and the original medical record and case report form of each subject are recorded with the screening number and initials; (3) Put all the research documents of the study in a special cabinet and lock them, and designate a special researcher to be responsible; (4) After the research files and data are locked, the viewing permissions of the files and data are restricted. This information is limited to the investigator of the project, the ethics committee, and the national drug regulatory department when necessary.

After the end of the study, the research institution shall keep all the information of the clinical research project in accordance with the relevant national laws and regulations, including the clinical research protocol of the research project, the informed consent form, the original medical record, the case report form, the investigator's manual, the approval of the academic committee, the approval of the ethics committee, the research protocol, the detailed records of the distribution of stem cell preparations, the records of adverse events and serious adverse events, etc. The retention period of all records shall be implemented in accordance with the requirements of the national drug administration department.

2. **The results of the study are published/published:**

After the completion of the research, the project team will publish a research paper or disclose other forms of research results. In published research papers or published research results, the subject's personal information will be hidden, and the subject's name or any other content that can identify the subject will not appear and will not be disclosed at any time in the future.

(12) The use method, dose, time and course of stem cell preparations

1. **Preparation of stem cell preparations**: Stem cell preparations are prepared by Beijing SH Biotechnology Co., Ltd., Beijing 10070, China in a cell production workshop with GMP production conditions: healthy human dental pulp is selected for primary cell isolation and culture, primitive cell banking, and working cells. The library and cell stock solution are produced and cryopreserved, and the cell stock solution is the cell stock solution after the verification of various indicators meets the proposed standards. The cells were thawed before use, washed with sodium chloride injection, and then resuspended in 0.6mL of sodium chloride injection, which was a human pulp mesenchymal stem cell preparation, and the preparation specification was approximately 1×107 hDP-MSC cells/0.6 mL/branch.

The batch numbers of the three batches used in this study are: 01012012110205D, 01012012110701D, 01012012111304D, all of which have passed the strict enterprise self-inspection and the third-party verification of the China Institute for Food and Drug Control.

2. **Stem cell preparation therapeutic dose:** This study is conducted in 2 phases.

In the first phase, there are 2 groups, and the therapeutic dose of stem cell preparation is as follows:

- **Stem cell therapy group:** A treatment dose of 0.6 mL/tooth position, the cell volume was about 1×107 hDP-MSC cells/tooth position/person, and the suspension was 0.6 mL of normal saline.
- **Normal saline control group:** a treatment dose of 0.6 mL/tooth position, normal saline 0.6 mL/tooth position/person.

In the second phase, there are 3 groups, and the therapeutic dose of stem cell preparation is as follows:

- **Normal saline control group:** The therapeutic dose was 0.6 mL/tooth position, and normal saline 0.6 mL/tooth position/person.
- **Stem cell therapy group:** The therapeutic dose was 0.6 mL/tooth position, the cell volume was about 1×107 hDP-MSC cells/tooth position/person, and the suspension was Normal saline 0.6 mL.
- **Stem cell secondary administration group:** The first treatment dose was 0.6 mL/tooth position, and the cell volume was about 1×107 hDP-MSC cells/tooth position/person. The suspension was 0.6 mL of normal saline. One month later, the second treatment dose was the same as the first treatment dose of 0.6 mL/tooth position, the cell volume was about 1×107 hDP-MSC cells/tooth position/person, and the suspension was 0.6 mL of normal saline

3. **Stem cell preparation treatment:** local bilateral multi-point injection of a single affected tooth of the subject.

（1） **Treatment operation procedures:** under local anesthesia, use a periodontal probe to determine the position of the alveolar ridge, insert the needle at the bottom of the periodontal pocket (in the tissue around the bottom of the periodontal pocket), and wait for the needle to reach the periodontal ligament on the medial side of the alveolar ridge, and touch the junction between the bone surface and the tooth root, and use 1ml screw.The rotary syringe is injected at multiple intervals on both sides of the buccal (lip) and tongue and palate, and each injection time is 20-30 seconds. The insertion point of the needle is located on the surface of the tooth root to ensure that the injected drug can accumulate at the tooth root. After the end of the treatment with bilateral multi-point local injection of hDP-MSC cells/normal saline, all subjects underwent 2-hour adverse reaction observation and safety evaluation in the periodontology/emergency comprehensive care center.

（2） **Course of treatment:** In the first phase of this study, one local injection administration is a course of treatment. In the second phase of this study, two local injections were administered as a course of treatment, and subjects in the stem cell secondary administration group were administered with one local injection one month after the first dose.

(13) Stem cell preparation management

1. **Stem cell preparations are out of the warehouse**: Investigators applying stem cell preparations need to apply to the preparation institution 1 day in advance, and the quantity should be clarified. After the preparation of the preparation is completed, the preparation institution shall immediately conduct quality inspection. Stem cell preparations are shipped out of the warehouse together with the quality inspection report. The test items include: appearance, cell number, cell viability, sterility test, bacterial endotoxin content, bovine serum albumin residue, dimethyl sulfoxide, etc. Among them, the results of sterility test, bovine serum albumin residue and dimethyl maple can lag behind.

(Note: The preparation institution produces 1 more stem cell preparation per batch for release inspection and sample traceability.)

2. **Stem cell preparations received**: The personnel of the preparation institution are responsible for ensuring that the preparation is transported at 4 °C and recording the temperature. When the agent arrives at the research institution, the personnel of the preparation institution will hand over and record the researcher responsible for the management of the stem cell preparation, and check the following information:

- The packaging of stem cell preparations is complete and undamaged, and the label is clear and legible.
- Appearance of stem cell preparation: After shaking well, it is a light milky white liquid, and does not contain insoluble substances visible to the naked eye.
- Complete label information of stem cell preparations: marked as exclusively for clinical research, indicating the research protocol number, preparation name, specification, preparation batch number, storage conditions, method of use, production date, expiration date, preparation preparation institution, etc.
- Quality inspection report information: the preparation name, specification, preparation batch number and stem cell preparation label are consistent, and all inspection items are qualified.
- Temperature monitoring record: check whether the temperature of the temperature recorder meets the stability requirements of the preparation, record the highest value, the lowest value, the current value, and print the temperature record sheet of the preparation transportation and storage process after the local injection treatment of the stem cell preparation is completed.

Handover completed, fill out the "Registration Form for Receiving and Recycling of Stem Cell Formulations".

3. **Stem cell preparation release**: The preparation institution produces 1 more stem cell preparation per batch for institutional release inspection and sample traceability. The investigator responsible for the management and distribution of stem cell preparations will hand over the preparations to the quality control personnel of stem cell preparations; Subsequently, the quality control personnel of stem cell preparations used an inverted microscope to complete the release test in the laboratory of the Institute of Stomatology

4. **Stem cell preparation recovery:** Recovered preparations include: empty bottles of post-injection preparations, unused remaining preparations and unqualified preparations dose. The personnel of the preparation institution and the researcher responsible for the management and distribution of stem cell preparations jointly check the label information of the recovered stem cell preparations, and put them in the shipping box after confirming that they are correct. At the same time, fill in the "Stem Cell Preparation Receipt and Recycling Registration Form".

(14) Criteria for discontinuation and termination of clinical studies

1. Suspension criteria: Once infection, hematoma or periodontitis progression is found in the course of clinical research, it will be dealt with in strict accordance with the expected adverse event treatment measures specified in advance, and the relationship between the adverse reaction and stem cell preparations will be analyzed, and the comprehensive evaluation will continue. Risks of conducting this clinical study, and if continuing the study would pose a greater or more serious risk to the subject, the study will be discontinued.

2. Termination Criteria: Once any of the following problems are found in the course of clinical research, the study will be terminated:

(1) Serious safety problems occurred in the study (such as those with serious adverse reactions or serious complications or rapid deterioration of the condition);

(2) The study found that the effect was poor, or even ineffective, and had no clinical value, and continued treatment would delay the subject's treatment;

(3) In the study, it was found that there were major mistakes in the formulation of the clinical research plan, or there were major deviations in the implementation of the plan, and it was difficult to evaluate it if it continued.

(15) Efficacy evaluation criteria

The efficacy evaluation was evaluated at D7±1 (telephone follow-up), D30±3, D90±3, D180±5, D360±5 after study treatment.

1. Safety evaluation index

(1) Vital signs: respiration, heart rate, blood pressure (systolic blood pressure, diastolic blood pressure), body temperature (armpits), and clinical evaluation of general condition. During the screening period, before administration, 2h±30min after the end of administration, D30±3 during the follow-up period, and in the second stage of this study, the subjects in the stem cell second dose group received second dose treatment for 2 hours ± 30 minutes after the end of the second dose treatment.

(2) Laboratory tests：

- Routine blood count: white blood cells, red blood cells, hemoglobin, hematocrit, mean corpuscular volume, mean hemoglobin amount, mean hemoglobin concentration, platelet count, mean platelet volume, platelet hematocrit, neutrophil cells, lymphocytes, monocytes, eosinophils, basophils, neutrophils, lymphocytes, monocytes, eosinophils, basophils, erythrocytes SD, erythrocytes CV, macroplatelet ratio, platelet width; It was examined during the screening period, before dosing, and during the follow-up period D30±3, D90±3, D180±5.

- Coagulation function: plasma prothrombin time, international normalized ratio of prothrombin time, activated partial thromboplastin time, thrombin time, plasma fibrinogen; During the screening period, pre-dose examination.

- Liver and kidney function: alanine aminotransferase, aspartate aminotransferase, total protein, albumin, globulin, white globule ratio, total bilirubin, direct bilirubin, indirect bilirubin, alkaline phosphatase, creatinine, γ-glutamyl transfection Enzyme transfers, lactate dehydrogenase, creatine kinase, urea, uric acid, total carbon dioxide, glucose; It was examined during the screening period, before dosing, and during the follow-up period D30±3, D90±3, D180±5.

- Inflammatory indicators: hypersensitive C-reactive protein, which was examined during the screening period, before administration, 2h±30min after the end of administration, and before and after the second dose of stem cells in the second phase of stem cell secondary administration group of this study2h±30min。

- Infectious diseases: HBsAg, HBsAb, HBeAg, HBeAb, HBcAb, Anti-HCV, HIVcombin, Anti-TP, CMV-IgM, CMV-IgG; check D30±3, D90±3, D180±5 during the screening period and follow-up period.
- Immunology: IgA, IgG, IgM, Total IgE; During the screening period, before administration, 2h±30min after the end of administration, D30±5, D90±5, D180±5, D360±5, and the second dose group of stem cells in the second stage of this study 2h ±30min after the end of the second dose treatment.
- Pregnancy test: blood β-HCG, premenopausal female subjects in the screening period, pre-drug, follow-up period D30±5, D90±5, D180±5 inspection.
- Urine routine: PH value, specific gravity, protein, ketone body, bilirubin, urobilinogen, nitrite, white blood cells, red blood cells, urine glucose, urine sediment microscopic examination, during the screening period, before treatment.

(3) For subjects with adverse reactions, timely treatment, follow-up inspection and recording should be carried out in accordance with the diagnosis and treatment routine.

(4) Adverse events and serious adverse events: The occurrence of adverse events and serious adverse events was recorded in a timely manner during the study, and the degree of adverse events was determined according to the NCI CTCAE v4.03 grading standard. Swollen and painful gums within one week after surgery, recorded as an adverse event, judged by the investigator to be an administration-related adverse reaction.

2. Efficacy evaluation index

(1) Primary efficacy measures

- 【Oral clinical testing indicators】During the screening period, before administration, and follow-up period, D90±3, D180±5, and D360±5 were examined with Florida probe, and the measured value of D90±3 was used as the evaluation endpoint.

  Periodontal attachment level AL (PD+GR).

(2) Secondary efficacy measures

- 【Radiological detection index】D90±3, D180±5, D360±5 were examined during the screening period and follow-up period, and the indicators in the screening period were used as the baseline index, and the measured value of D180±5 was used as the evaluation endpoint.
- Change in periodontal defect height at 3 months, 6 months, and 12 months after the end of treatment compared to baseline (CBCT measurements, change in periodontal defect height = difference in distance from enamel bone boundary to alveolar crest).
- Change in mean density of alveolar ridge after 3 months, 6 months, 12 months after the end of treatment compared to baseline (CBCT measurement results).

[Oral clinical testing indicators]: During the screening period, pre-administration, and follow-up periods, D90±3, D180±5, and D360±5 were examined with Florida probes, and the measured values of D90±3 were used as the evaluation endpoints.

- Periodontal probing depth PD compared to baseline
- Periodontal healing compared to baseline (probing bleeding index BOP, gingival recession GR, tooth looseness TM).

(16) Recording requirements for adverse events and reporting methods and treatment measures for serious adverse events

1. Security Data Collection

The safety of stem cell preparations was assessed by recording, reporting, and analyzing baseline conditions, adverse events, vital signs, laboratory tests, and oral examinations. Throughout the study, any adverse events experienced by the subjects will be comprehensively assessed starting with the subject's signing of informed consent.

Subjects who cannot undergo safety evaluation can be replaced by newly enrolled subjects, such as subjects who have not completed the study according to the requirements of the protocol, including those who have withdrawn early, do not meet the inclusion criteria, and have not completed the treatment and visits as specified in the protocol.

Subjects may withdraw from the study at any time without giving any reason. If the subject has poor compliance or violates the protocol, the investigator can also decide whether to withdraw the subject from the study.

2. Adverse events (AE)

Adverse events (AEs): refer to all adverse medical events that occur in subjects during the course of a clinical study, which can be manifested by symptoms and signs, diseases or abnormal laboratory tests, and do not necessarily have a causal relationship with treatment.

Note: Planned surgery or hospitalization that existed prior to the subject's receipt of informed consent to participate in the clinical study but did not deteriorate during the study or was performed prior to the implementation of study entry into the study is not an adverse event.

3. Judgment of relevance of adverse events

The relationship between AEs and the study drug was judged according to five levels of "definitely related, probably related, probably related, probably unrelated, and definitely unrelated", and the incidence of adverse reactions was included in the first three levels.

Affirmatively related: AEs are consistent with the types of adverse effects common to the study drug, and their occurrence has a reasonable temporal relationship with the study drug, AEs disappear after discontinuation and are not related to concomitant medications or non-pharmacological factors (primary disease, complications, food, environment, etc.).

Likely related: AEs consistent with common types of adverse effects of study medication, reasonable temporal relationship with study medication, significant remission of AEs after discontinuation, and AEs unrelated to concomitant medications or non-pharmacological factors.

Possibly related: AE conforms to the common adverse reaction types of study medication and has a reasonable time relationship with study medication. The AE is not significantly relieved after drug withdrawal. The relationship between concomitant medication or non-drug factors and AE cannot be ruled out.

Possibly unrelated: The adverse reaction has a reasonable time relationship with the study drug, but does not meet the common adverse reaction types of the study drug. The AE is not relieved after stopping the drug. The AE can be explained by concomitant medication or non-drug factors.

Irrelevant: The AE has no reasonable time relationship with the study medication and does not meet the common adverse reaction types of the study medication. The AE is not relieved after discontinuation of the medication. The AE is definitely related to concomitant medication or non-drug factors.

4. Anticipated adverse events and treatment plans

Anticipated Adverse Events: Adverse events that subjects may face while participating in the study. Possible

adverse events in this study and The handling measures are as follows.

(1) Local adverse events on teeth: mild inflammatory reactions such as mild redness and swelling of the gums; severe inflammatory reactions such as loose periodontal pockets and pus overflow, periodontal ligament edema, etc. The researchers conducted symptomatic treatment in accordance with the routine clinical diagnosis and treatment of periodontal disease: for mild inflammatory reactions in periodontal tissue, local irrigation of the periodontal pockets and gargling with mouthwash were performed; for severe inflammatory reactions in periodontal tissues, debridement of the periodontal pockets was performed. Antibiotics are built into the bag. If necessary, local abscesses can be incised and drained and gargled with mouthwash.

(2) Injection local hematoma: It manifests as injection local blood vessel damage, intra-tissue bleeding, and purple-red petechial or masses under the mucosa or subcutaneously. Principles of prevention and treatment: If hematoma has appeared locally, immediate compression can be used to stop the bleeding and cold compress should be applied; subjects are instructed to apply hot compress after 48 hours to promote the absorption and dissipation of the hematoma. Antibiotics and hemostatic drugs may be given as appropriate.

(3) Systemic adverse events: chills, fever, mild headache, which can subside naturally.

(4) Progression of periodontitis: During the study period, if the subject's periodontitis progresses, the researcher will judge whether to intervene or not. For example, other treatments or drugs may be used to intervene that may affect the study, or the researcher may evaluate whether to withdraw.

(5) In clinical drug research, a very small number of subjects may suffer from shock due to allergic reactions. Once a subject develops anaphylactic shock, treatment will be carried out in accordance with the "Anaphylactic Shock First Aid Plan Standard Operations".

5. Unexpected adverse events

Unexpected adverse events: events that occur during clinical research and meet the following three criteria: ① The nature, severity and incidence are unexpected; ② Related or potentially related to participation in the study; ③ Subjects or others face greater risks. Big risk. In clinical studies, when the same adverse event occurs in multiple subjects, and the nature, severity, and frequency are not mentioned in the current investigator's manual or protocol as related to the study drug, the investigator should report it to the preparation preparation as soon as possible. The institution reports this adverse event. If the adverse event is confirmed to be an unexpected adverse drug reaction, the researcher should assist the preparation preparation institution to write a safety report and submit it to the drug regulatory department and ethics committee, and notify all researchers, and amend the protocol and informed consent form if necessary, while revising the Investigator's Manual to include new adverse reactions or changes in the frequency and severity of known adverse reactions.

6. Serious adverse events (SAE)

Events that require hospitalization, prolong hospitalization, become disabled, affect work ability, endanger life or death, or cause congenital malformations occurrence during clinical research.

Note: "Life-threatening" in the definition means that the subject is in danger of death if the event occurs; it does not mean that if the event is serious, it will lead to death.

7. Important adverse events

In addition to serious adverse events, any adverse events that result in targeted medical measures (such as discontinuation, dose reduction, and symptomatic treatment) and significant abnormalities in hematology or other laboratory tests.

Note: Medical and scientific judgment should be used to determine whether an event is a medically important event. An important medical event may not necessarily be immediately life-threatening and/or result in death or

hospitalization. However, if it is determined that an event may harm the subject or may require medical intervention to prevent the occurrence of any of the above serious adverse event outcomes, then the important medical event should be reported as a serious adverse event.

8. Requirements for recording adverse events

From the time the subject signs the Informed Consent Form (ICF) until the end of the study, regardless of whether or not the subject receives treatment with stem cell preparations, both Adverse Events (AEs) should be recorded regardless of whether they are observed by the investigator or reported by the subject. The investigator should fill in truthfully during the study. The type, degree, time of appearance, duration, treatment measures and treatment history of AEs should be recorded in detail. The comorbidities should be considered in a comprehensive manner, combined medications and evaluate their relevance to DPSCs preparations.

All physical examination and laboratory test results required by the clinical research protocol should be recorded in the original medical record of the subject. Compare the subject's physical examination results and laboratory tests after the local injection treatment with the results before the local injection treatment. If the change is suggestive of a deterioration in the subject's clinical status, the Investigator must evaluate to determine if it meets the definition of AEs. All changes determined to meet the definition of an AEs will be documented in the AEs section of the original medical record.

Medical documentation of AEs should be documented in the original file, including oral periodontal exams, blood exams, imaging, and CBCT exams. Examination notification forms and results report forms for CBCT examinations. If the subject is unable to continue to treatment by the Investigator, etc., the Investigator shall submit a summary of the subject's medical record (including treatment schedule and indication of the need for continued follow-up of adverse events, etc.) to the person in charge of the study (including a description of the treatment schedule and the need for continued follow-up of adverse events, etc.) to the physician responsible for their continued treatment. This information should also be recorded in the original documentation.

AEs need to be followed up by the investigator to the baseline level or to the final outcome.

9. Reporting of adverse events

9.1 Anticipated adverse events: collected, recorded in a timely manner and reported regularly to the Ethics Committee.

9.2 Unintended adverse events: timely collection, recording and regular reporting to the Ethics Committee, assessment of risk, the investigator will assist the preparation organization to write a safety report to the institutional ethics committee and inform all investigators, and amend the protocol and informed consent form if necessary. The protocol and consent form should be amended and the investigator's manual should be revised.

9.3 Serious adverse events: If any serious adverse event or significant AE occurs, whether or not it is related to the study intervention and whether or not the intervention has been performed, the investigator should report the event to the institution, the ethics committee, the preparation organization, the Beijing Municipal and National Health Organization, and to the investigator within 24 hours of knowledge. The clinical research organization ensures that all reporting procedures required by laws and regulations are met. The investigator should document in the

source material when, how, and to whom the serious adverse event was reported.

The contact list for serious adverse events is as follows:

Name	Function	Fixed telephone	Mobile phone
General duty office	On administration duty	010-57099005	
Wang Songling<br>Liu Yi	Principal investigator	010-57099450	13601324511<br>18600262811
Jing Xinying	Institutional Office	010-57099310	
Li Ying	Ethics Committee	010-57099318	
Jin Jun	Medical Service	010-57099020	
Su Min	Formulation preparation institution	010-83602352-807	13716028122
Bai Bing	Beijing Health Commission	010-89150001	
Yin Xuke	Beijing Health Commission	010-68792955	

The investigator must complete the SAE Report Form. All study-related information must be documented on the SAE form, including a description of the clinical course of the serious adverse event, assessment of severity, causal relationship to the study drug or procedure, action taken, and event outcome. The subject should be closely observed until the condition resolves or the cause of the condition has been determined and all follow-up information has been recorded on the Serious Adverse Event Follow-up Report Form.

Subjects withdrawing from the study with an SAE will be followed until normal or until there is a reasonable medical explanation. According to the severity of the adverse events, the mode of follow-up can be inpatient, outpatient, home visit, telephone or other forms.

10. Management and reporting of pregnancy events during the study period

In the event that a pre-menopausal female subject experiences a pregnancy during the study, the investigator should immediately discontinue the subject's clinical. The Investigator shall communicate scientifically and rigorously with the subject based on the information about the stem cell preparation and inform her of the possible effects and risks of the stem cell preparation on the pregnant woman and the fetus. The investigator should complete a "Pregnancy Report Form" within 24 hours of confirmation of pregnancy in a female subject of childbearing potential. The report should be made to the institution, the ethics committee, the preparation organization, the Beijing Municipality and the National Health Commission.

(XVII) Risks and benefits of research

1. Risks and precautions, treatment plan:

1.1 Physical risks and treatment:

(1) Localized dental adverse events: mild inflammatory reactions such as mild gingival redness and swelling; severe inflammatory reactions such as periodontal pocket relaxation and pus overflow, periodontal edema, etc. The researcher followed the clinical periodontal disease diagnosis and treatment routine for symptomatic treatment: for the mild inflammatory reaction of periodontal tissues, local flushing of periodontal pockets can be given; for severe inflammation of periodontal tissues, internal debridement of periodontal pockets and internal

antibiotics are given, and local abscess incision and drainage with mouthwash can be done if necessary.

(2) Local hematoma of injection: manifested as local blood vessel injury, bleeding in the tissue, purplish-red bruise under the mucous membrane or subcutaneous. Principles of prevention and treatment: if local hematoma has already appeared, it can be immediately compressed to stop bleeding, and cold compresses can be applied; subjects should be instructed to apply hot compresses after 48 hours to promote the absorption and dissipation of hematoma. Antibiotics and hemostatic drugs can be given as appropriate.

(3) Systemic adverse events: chills, fever, mild headache, which can subside spontaneously. In rare cases, a generalized allergic reaction may occur, and shock may occur as a result of the allergic reaction. In the event of anaphylaxis, follow the "Anaphylaxis Emergency Procedures". If anaphylaxis occurs in a subject, treatment should be carried out in accordance with the "Standard Practice for Anaphylaxis".

1.2 Psychological Risks and Management: Some subjects may experience psychological stress due to a lack of knowledge about DPSCs preparations. If so, the researcher will provide psychological intervention and introduce the current status of stem cell clinical research in China and the world, the possible mechanism of action of the DPSCs preparation in this study, and the expected adverse events in the clinic without reason at any time. And inform the subjects that they can withdraw from the study at any time without any reason, so as to eliminate or alleviate the psychological pressure of the subjects.

1.3 Risks to society and treatment: Some subjects may have concerns about the safety of the personal information collected during the study. If so, the researcher will explain to the subjects that their private information will be kept strictly confidential at all times during the study. It is also stated that the subjects' private information will only be provided to the investigators of the project, the Ethics Committee and the State Drug Administration when necessary.

1.4 Financial risks and management: Subjects may be concerned about the cost of the study and may be burdened with the cost of lost labor due to participation in the study. The Investigator will inform the subject that periodontal treatment, DPSCs injection therapy, and other periodontal treatment that may have been performed as a result of participation in the study will not be covered by the Investigator. Basic treatment, DPSCs injection treatment, and periodontal clinical indicators, laboratory tests, and CBCTperformed for participation in this study will be covered by the subject's group. The investigator affirms that subjects will receive \$300 per visit as a transportation allowance and compensation for missed work for participation in this study. The investigator will be responsible for providing cash payment to the subject at the end of each visit. If the subject can complete all visits in this study, the subject will receive transportation allowance and compensation for lost work for 7 visits, totaling \$2100 per subject.

2.Benefit:

2.1 Benefit to Subjects: this study intends to enroll subjects with chronic moderate periodontitis. During the study, the investigator will conduct the oral periodontal examination, laboratory and CBCT examination, and the subjects will obtain their own health-related information. All subjects were screened and enrolled in the study and underwent a full-mouth periodontal foundation treatment (including supragingival scaling, subgingival scraping, and a periodontal treatment), which was beneficial for chronic moderate periodontitis. If subjects were assigned to the DPSCs injection group, after basic periodontal treatment combined with

local DPSCs injection, the subjects' periodontal inflammation may be alleviated or resolved.
2.2 Benefit to science and society: In chronic moderate periodontitis, current conventional treatments are barely able to regenerate the defective periodontal tissues. The purpose of this study is to evaluate the safety and efficacy of DPSCs injection in the treatment of chronic moderate periodontitis, and to provide a basis for the development of a standardized clinical protocol for the treatment of chronic moderate periodontitis with DPSCs injection.
The results of this study are summarized in the following table.

(XVIII) Sample case report form (see Annex 2: Case Report Form (CRF)

(XIX) Statistical analysis of study results

1. Data management

This study used paper version of the original medical record book (see Annex 3: "Original Medical Record Book") for data collection, which was conducted by the researcher in accordance with the GCP and the requirements of the study protocol to fill in the original medical record book accurately, timely, truthful, complete and standardized, signed and dated. The present study used Clinflash EDC (Electronic Data Capture) system, which was used for data collection by the entry personnel according to the original medical records. The data were entered into the EDC in an accurate, timely, complete and standardized manner in accordance with the EDC guidelines. eCRFs were completed by a supervisor assigned by the preparation facility. The completed eCRF will be logged into the EDC by the supervisor assigned by the preparation institution at the research center to check the consistency between the eCRF data and the source data, and any problems found can be challenged online at any time. If problems are found, they can be challenged online at any time. The data administrator will manually check the data, and if there is any problem, he/she will send out a query to the researcher for confirmation and answer the query.
After the data review is completed, the principal investigator, data management personnel, statistical analysts, and the preparation organization will jointly review the data and complete the final definition and judgment of the analyzed population. The database is locked by the data management staff after the electronic signature of the principal investigator is completed.

2. Statistical analysis

The statistical analysts of this study were not involved in the screening, inclusion of subjects and subsequent clinical studies. During the course of the study, only blinded to the statistical analysts.
(1) Analyzed Population:
Statistical analysis datasets for the study results included the full analysis dataset (FAS), the protocol compliance dataset (PPS), and the safety Sexuality dataset (SS).
Full Analysis Set (FAS): for all cases that had used the study drug after enrollment and had completed at least one efficacy assessment. cases with at least one efficacy assessment completed after enrollment. The estimation of missing values for the main indicators was performed by the method of LOCF (last observation carrying forward).
Per Protocol Set (PPS): a subset of the FAS, all cases that comply with the study protocol,

have good compliance, do not use prohibited drugs during the study period, have completed 6 months of clinical observation, and have completed the CRF;

Safety Set (SS): all cases that received the study drug after enrollment and had at least one safety assessment. Cases.

In this study, baseline information was analyzed using FAS and PPS analysis. Primary efficacy indicators, secondary efficacy indicators, etc. were analyzed by both FAS and PPS. However, the conclusions obtained from FAS analysis were the main conclusions. When the conclusions from FAS and PPS are consistent, the credibility of the conclusions can be increased.

In the safety analysis, adverse events and adverse reactions, vital signs, and laboratory indicators were analyzed using SS. subjects who were terminated prematurely due to adverse events and various non-therapeutic reasons were included in the safety analysis.

(2) Statistical analysis plan: the analysis of the study results included the description of the distribution of subjects, the balance analysis of baseline indicators, the analysis of efficacy, and the analysis of safety.

Mean, standard deviation, median, quartile, minimum and maximum values were used for the statistical description of the measurement data. Comparisons between groups were performed by ANOVA or Kruskal-Wallis rank sum test, and comparisons in groups were performed by paired t-test or Wilcoxon signed rank test. test for within-group comparisons.

Statistical descriptions of counts and rank data used rate or composition ratio, comparisons of count data used x^2 test or Fisher's exact probability method, and comparisons of rank data used Kruskal-Wallis rank-sum test.

(XX) Follow up plan and implementation methods

The follow-up period of this study was 12 months, with telephone follow-up at D7±1 after local injection treatment and regular follow-up at D30±3, D90±3, D180±5, and D360±5. The investigator should observe the subjects strictly according to the protocol, and the subjects should try to follow up regularly as requested by the investigator. During the study period, treatment and follow-up should be performed in strict accordance with the protocol, taking into account the fact that a time window of ±3 days is allowed from 1 month after treatment with the stem cell preparation, and a time window of ±5 days is allowed from 6 months after treatment. If a subject exceeds the window at a follow-up visit, the next follow-up visit should be recalculated based on the time of the previous visit.

The examinations required to be performed at each follow-up visit were conducted by the investigator, and the relevant examinations were conducted in the imaging department, laboratory department and other specialized departments of the research center, and timely, accurate and complete records were made of the clinical indicators, laboratory results and imaging results of each observation, and of the combination of medications, adverse events, and so on. For subjects with adverse reactions, all indicators of the screening period were followed up until they were normalized.

1. Post-treatment follow-up 1: Telephone follow-up 1 week (D7±1 day) after the end of treatment.

a. Record of adverse events

b. Record of co-administration of drugs/treatment

2. Post-treatment follow-up 2: 1 month (D30±3 days) after the end of treatment.

a. Vital signs: respiration, heart rate, blood pressure (systolic, diastolic), temperature (axillary), clinical assessment of the general body condition, and another checkup 2h±30min after the end of the second administration of treatment for the subjects in the secondary stem cell administration group in the second phase of this study.

b. Laboratory tests:

- Blood count: leukocytes, erythrocytes, hemoglobin, erythrocyte pressure volume, mean erythrocyte volume, mean hemoglobin concentration, platelet count, mean platelet volume, platelet pressure, neutrophil, lymphocytes, monocytes, eosinophils, basophils, absolute value of neutrophils, absolute value of lymphocytes, absolute value of monocytes, absolute value of eosinophils, absolute value of basophils, erythrocyte distribution width SD, erythrocyte distribution width CV, large platelet ratio, platelet distribution width.
- Liver and kidney function: alanine aminotransferase, aspartate aminotransferase, total protein, albumin, globulin, white globule ratio, total bilirubin, direct bilirubin, indirect bilirubin, alkaline phosphatase, creatinine, gamma-glutamyltransferase, lactate dehydrogenase, creatine kinase, urea enzyme, lactate dehydrogenase, creatine kinase, urea, uric acid, total carbon dioxide, glucose.
- Infectious disease detection: HBsAg, HBsAb, HBeAg, HBeAb, HBcAb, Anti-HCV, HIVcombin, Anti-TP, CMV-IgM, CMV-IgG.
- Immunological tests: IgA, IgG, IgM, Toal IgE.
- Pregnancy test: blood beta-HCG, for premenopausal female subjects only.
- Inflammatory indicators: only for subjects in the secondary stem cell dosing group in phase II of this study before secondary dosing treatment, examined 2h±30min after the end of treatment.

c. Record of adverse events

d. Record of combined medications/treatments

e. Intraoral photography: photographic record of oral cavity and treatment site conditions.

3. Post-treatment follow-up 3: conducted 3 months (D90 ± 3 days) after the end of treatment.

a. Periodontal Clinical Indicators: Periodontal Clinical Indicators: Periodontal examinations were standardized using the Florida Probe of the Beijing Stomatological Hospital of Capital Medical University. Records: periodontal attachment level AL, periodontal probing depth PD, probing bleeding index BOP, gingival recession GR, and tooth looseness TM.

b. Laboratory tests:

- Blood count: leukocytes, erythrocytes, hemoglobin, erythrocyte pressure volume, mean erythrocyte volume, mean hemoglobin concentration, platelet count, mean platelet volume, platelet pressure, neutrophil, Lymphocytes, monocytes, eosinophils, basophils, absolute value of neutrophils, absolute value of lymphocytes, absolute value of monocytes, absolute value of eosinophils, absolute value of basophils, erythrocyte distribution width SD, erythrocyte distribution width CV, large platelet ratio, platelet distribution width.
- Hepatic and renal function: alanine aminotransferase, aspartate aminotransferase, total protein, albumin, globulin, leukoglobulin ratio, total bilirubin, direct bilirubin, indirect bilirubin, alkaline phosphatase, creatinine, gamma-glutamyltransferase enzyme, lactate

dehydrogenase, creatine kinase, urea, uric acid, total carbon dioxide, glucose.

- Infectious disease tests: HBsAg, HBsAb, HBeAg, HBeAb, HBcAb, Anti-HCV, HIVcombin, Anti-TP, CMV-IgM, CMV-IgG.
- Immunologic tests: IgA, IgG, IgM, total IgE.
- Pregnancy test: blood beta-HCG for premenopausal female subjects only.

c. Record of adverse events

d. Record of co-medication/treatment

e. Imaging: CBCT examination.

f. Intraoral photography: photographic record of oral cavity and treatment site conditions.

4. Post-treatment follow-up 4: conducted 6 months (D180 ± 5 days) after the end of treatment.

a. Periodontal clinical indicators: The periodontal examination was standardized using the Florida Probe of the Beijing Stomatological Hospital of Capital Medical University. Examination. Records: periodontal attachment level AL, periodontal probing depth PD, probing bleeding index BOP, gingival recession GR, tooth looseness TM. recession GR, and tooth looseness TM.

b. Laboratory tests:

- Blood count: leukocytes, erythrocytes, hemoglobin, red blood cell pressure, mean red blood cell volume, mean hemoglobin concentration, platelet count, mean platelet volume, platelet pressure volume, neutrophils, lymphocytes, monocytes, eosinophils, basophils, absolute value of neutrophils, absolute value of lymphocytes, absolute value of monocytes, absolute value of eosinophils, absolute value of basophils, erythrocyte distribution width SD, erythrocyte distribution width CV, large platelet ratio, platelet distribution width.
- Liver and kidney function: alanine aminotransferase, aspartate aminotransferase, total protein, albumin, globulin, white globule ratio, total bilirubin, direct bilirubin, indirect bilirubin, alkaline phosphatase, creatinine, gamma-glutamyltransferase enzyme, lactate dehydrogenase, creatine kinase, urea, uric acid, total carbon dioxide, glucose.
- Infectious disease tests: HBsAg, HBsAb, HBeAg, HBeAb, HBcAb, Anti-HCV, HIVcombin, Anti-TP, CMV-IgM, CMV-IgG.
- Immunologic tests: IgA, IgG, IgM, total IgE.
- Pregnancy test: blood beta-HCG for premenopausal female subjects only.

c. Record of adverse events

d. Record of co-medication/treatment

e. Imaging: CBCT examination.

f. Intraoral photography: photographic documentation of the oral cavity and treatment site conditions.

5. Long-term follow-up: 12 months (D360±5 days) after the end of treatment.

a. Periodontal clinical indicators: The periodontal examination was standardized using the Florida Probe of the Beijing Stomatological Hospital of Capital Medical University. Records: periodontal attachment level AL, periodontal probing depth PD, probing bleeding index BOP, gingival recession GR, tooth looseness TM.

b. Laboratory tests:

- Immunologic tests: IgA, IgG, IgM, total IgE.

c. Record of adverse events

d. Record of co-medication/treatment
e. Imaging: CBCT examination.
f. Intraoral photography: photographic record of oral cavity and treatment site conditions.

6. Unplanned follow-up and treatment

If an adverse event occurs during the study period, the investigator may increase the number of follow-up visits or treatments, i.e., unplanned follow-up visits and treatments, according to actual needs. The investigator must accurately record the contents of each unplanned follow-up visit and treatment in the unplanned follow-up section of the original medical record and CRF.

- Chief complaint symptoms;
- Vital signs: respiration, heart rate, blood pressure (systolic, diastolic), temperature (axillary), clinical assessment of general condition;
- Intraoral photography: postoperative condition of the oral cavity and each injection site;
- Laboratory tests: relevant laboratory tests for the screening period as practically required;
- Adverse event records;
- Records of combined medications/treatments;
- Decision on whether to perform imaging tests (CBCT examination) and the selection of appropriate ancillary screening programs, as actually required;
- Appointment for the next follow-up visit.

6. Follow-up of withdrawn/dislodged subjects:

Subjects may withdraw from the study at any time during the study. The researcher will examine all subjects to determine their periodontal health status, inform them of the precautions to be taken, and to contact the researcher promptly after any discomfort occurs. The researcher should also leave the contact information of the subjects to facilitate follow-up visits if necessary. In case of early withdrawal/exfoliation, the subject should contact the investigator or return to the hospital if any discomfort occurs during his/her absence.

For subjects who withdraw/dislodge early, the investigator should try to find out why they withdrew/dislodged and accurately record the time of withdrawal from the study/ shedding time.

(XXI) Quality control and assurance of research

1. Quality control measures

Standard operating procedures should be adopted by both the preparation organization and the investigator to ensure quality control and quality assurance systems for clinical studies. The quality control and quality assurance system should be implemented. All observations and findings in a clinical study should be verified to ensure the reliability of the study data and that the conclusions in the clinical study are derived from the original data. Quality control must be applied at every stage of data processing to ensure that all data are reliable and correctly processed.

2. Investigator training

Prior to the commencement of a clinical study, the study project leader should train the

investigator on the study protocol in order to provide the investigator with an understanding of and familiarity with the nature, effects, efficacy, and safety of the DPSCs preparation (including information from preclinical and preclinical studies of DPSCs preparations), as well as to keep abreast of all new information related to the stem cell preparation that comes to light during the course of the clinical study.

3. Improvement of subject compliance

After subjects obtain a randomization number and are assigned to an investigator for this research project, the investigator will be responsible for the follow-up of the subject. The investigator should carefully implement the Declaration of Helsinki, the current regulations in China, as well as the clinical study protocol and informed consent form, so that the subjects fully understand the requirements of the study. The investigator will make sure that the subjects fully understand the requirements of the study and cooperate with the study, and will remind the subjects of the follow-up time by telephone on a regular basis.

During the study, the periodontal basic treatment, DPSCs injection treatment and periodontal clinical indicators, laboratory tests, CBCT performed by the subjects as a result of participating in the study will be used to assess the effectiveness of the study. The cost of periodontal basic treatment, DPSCs injection treatment, periodontal clinical indicators, laboratory tests, and CBCT for subjects participating in this study will be covered by the Beijing Municipal Commission of Science and Technology Special Subject Matching Fee.

4. Ethical requirements and informed consent

This study follows the guidelines in the Declaration of Helsinki and adheres to the Code for Quality Management of Pharmaceutical Clinical Trials (2003 year), Measures for the Administration of Stem Cell Clinical Research (for trial implementation, 2015), Quality Control of Stem Cell Preparations and Preclinical Research Guiding Principles (Trial Implementation, 2015). The study protocol and its revision, informed consent form and its revision, original medical record and its revision, case report form (CRF) and its revision, and other written information to be provided to the subjects shall be approved by the Ethics Committee of Beijing Stomatological Hospital affiliated with Capital Medical University.

The investigator is responsible for explaining the benefits and risks of participating in this study to each subject, and subjects will be allowed to participate in the study only after signing the ICF. The final text of the ICF should contain the following information: the purpose of the study, obligations of the subject, foreseeable benefits and foreseeable risks and inconveniences of participating in the study, treatment and appropriate compensation for any research-related harm, access to research data, and confidentiality of the subject's information.

The informed consent form should be written in a language that the subject can read. The informed consent form should be signed and dated by the subject and the investigator performing the informed consent process. Two copies of the informed consent form should be kept by the research organization and the subject respectively.

During the the study, if new important information concerning the DPSCs preparation is discovered, the informed consent form must be revised in writing and the subjects' consent was obtained again after approval by the Ethics Committee of Beijing Stomatological Hospital of Capital Medical University.

5.Changes of the study protocol

After the approval of the study protocol by the Ethics Committee of Beijing Stomatological Hospital of Capital Medical University, any modification of the study protocol should be decided by the investigator in consultation with the preparation institution. The protocol should be revised by the investigator in consultation with the preparation institution, and a "Statement of Clinical Research Protocol Revision" and "Comparison Table of Clinical Research Protocol Revision" should be written and signed by the principal investigator implementation. No participant shall violate the study protocol.

6. Duties of the Parties

(1) Investigator

The investigator shall safeguard the quality of the clinical research performed and protect the safety and rights of the subjects. The clinical research institution shall establish an organizational structure suitable for the clinical research, set up a corresponding management system and equip relevant personnel, facilities and equipment to manage the clinical research on DPSCs undertaken by the institution.

The researcher shall explain to the subjects the details of the DPSCs clinical research as agreed by the Ethics Committee of Beijing Stomatological Hospital of Capital Medical University before the commencement of the clinical research.

(2) Preparation organization

The organization is responsible for initiating, applying for, organizing, and supervising DPSCs clinical research, and providing research funding according to the clinical research project cooperation agreement.

If an investigator conducts DPSCs clinical research in violation of the approved research protocol or relevant regulations, the preparation organization shall point out and seek correction. If the situation is serious or persistent, the organization shall terminate the participation of the researcher in the clinical research and report the case to the Ethics Committee of Beijing Stomatological Hospital of Capital Medical University, the research institution and the Food and Drug Administration.

7. Data Retention

In order to ensure the supervision and management by the State Health Commission and the State Drug Administration, the clinical research organization shall keep all the data of the clinical research project in accordance with the provisions of the relevant national laws and regulations, including the confirmation of all the subjects (which can effectively check different records), the original medical records and case report forms of the subjects, all original signed subject informed consent. All the originals of the clinical study protocol, investigator's manual, academic committee approval, ethics committee approval, research protocol, detailed records of stem cell preparation distribution, records of adverse events and serious adverse events, etc.. At the same time, the preparation organization shall keep a copy. The retention period of all records shall be in accordance with the requirements of the State Pharmaceutical Administration.

References

[1] Loesche WJ, Grossman NS. Periodontal disease as a specific, albeit chronic, infection: diagnosis and treatment. Clin Microbiol Rev. 2001;14(4):727-752.
[2] Gronthos S, Mankani M, Brahim J, et al. Postnatal human dental pulp stem cells (DPSCs) in vitro and in vivo. Proc Natl Acad Sci USA, 2000;97(25): 13625-13630.
[3] Marquez-Curtis LA, Janowska-Wieczorek A, McGann LE, et al. Mesenchymal stromal cells derived from various tissues: Biological, clinical and cryopreservation aspects. Cryobiology. 2015;181-197.
[4] Egusa H, Sonoyama W, Nishimura M, et al. Stem cells in dentistry--part I: stem cell sources. J Prosthodont Res. 2012;56(3):151-165.
[5] Feng F, Akiyama K, Liu Y, Yamaza T, et al. Utility of PDL progenitors for in vivo tissue regeneration: a report of 3 cases. Oral Dis. 2010;16(1):20-28.
