# Supplementary material for: Impact of allogeneic dental pulp stem cell injection on tissue regeneration in periodontitis: a multicenter randomized clinical trial
Source: Signal Transduct Target Ther. 2025 Jul 31;10:239. doi: 10.1038/s41392-025-02320-w (PMC12311062; doi:10.1038/s41392-025-02320-w)
Supplement: Supplementary file 3 — Supplementary File 2 [file 41392_2025_2320_MOESM3_ESM.pdf]

A randomized, double-blind, dose-increasing, (blank) controlled clinical safety and tolerability trial of periodontal initial therapy combined with DPSCs injection for chronic periodontitis

Product Name:	DPSCs injection
Solution No.:	SH-hDP-MSC-101
Edition:	V3.3
Edition Date:	February 11, 2022
Research Center:	Department of Stomatology, Peking University Third Hospital
Principal Investigator:	Wang Xiao; Li Haiyan
Applicant:	Beijing SH Biotechnology Co., Ltd.<br>Capital Medical University
Applicant Address:	Floor 2, Building 2, Yard 8, Haiying Road, Fengtai District, Beijing<br>No.10, Youanmenwai, West 1st Alley, Fengtai District, Beijing

Confidentiality Statement

In this clinical trial protocol, there are commercial secrets, and the commercial information is privileged. The ownership of all information contained in this plan belongs to Beijing SH Biotechnology Co., Ltd., and Capital Medical University. No information herein shall be disclosed without legal permission. It can only be provided for review by relevant institutions, including researchers, collaborating researchers, relevant ethics committees and regulatory authorities Without approval from the applicants and research centers under the plan, it is prohibited to disclose any information to any third party unrelated to this research or for any other purposes, except for providing necessary explanation when signing informed consent with the subjects related to this research.

Signature page

A randomized, double-blind, dose-increasing, (blank) controlled clinical safety and tolerability trial of periodontal initial therapy combined with DPSCs injection for chronic periodontitis

Declaration and signature of relevant personnel

Applicant

We have carefully discussed and revised the clinical trial protocol of the aforesaid project with all parties involved in the trial, and we will conduct the clinical trial in accordance with the provisions of this clinical trial protocol. During the clinical trial process, we will fulfill our obligations as an Applicant in accordance with the provisions of the *Good Clinical Practice (GCP), Management Measures for Stem Cell Clinical Research (Trial)* and relevant laws and regulations.

Applicant: Beijing SH Biotechnology Co., Ltd., Beijing 100070, China

Project leader: Wang Hua

Project leader (Signature): Wang Hua (signature) Date: Feb. 16, 2022

Signature page

A randomized, double-blind, dose-increasing, (blank) controlled clinical safety and tolerability trial of periodontal initial therapy combined with DPSCs injection for chronic periodontitis

Declaration and signature of relevant personnel

Applicant

We have carefully discussed and revised the clinical trial protocol of the aforesaid project with all parties involved in the trial, and we will conduct the clinical trial in accordance with the provisions of this clinical trial protocol. During the clinical trial process, we will fulfill our obligations as an Applicant in accordance with the provisions of the *Good Clinical Practice (GCP), Management Measures for Stem Cell Clinical Research (Trial)* and relevant laws and regulations.

Applicant: Capital Medical University

Project leader: Wang Songling

Project leader (Signature): Wang Songling (signature) Date: Feb. 18, 2022

Signature page

A randomized, double-blind, dose-increasing, (blank) controlled clinical safety and tolerability trial of periodontal initial therapy combined with DPSCs injection for chronic periodontitis

Declaration and signature of relevant personnel

Research Center

I, the undersigned, agree with all the content of this plan and will carry out the clinical trial in accordance with this plan, and hereby confirm that:

I understand and will carry out this clinical trial in accordance with this plan and all approved amendment to this plan and will follow all provisions of the *Good Clinical Practice (GCP)* and all laws and regulations applicable for clinical trial.

I will fulfill my obligations according to this plan. Without prior written permission from the Applicant and written approval from the independent ethics committee, I will not violate this plan except necessary measures to be taken for avoiding immediate harm to the subjects.

Research Center: Department of Stomatology, Peking University Third Hospital, Beijing 100191, China

Principal Investigator: Wang Xiao; Li Haiyan

Principal Investigator (Signature): Wang Xiao (signature) Date: Feb. 17, 2022

Principal Investigator (Signature): Li Haiyan (signature) Date: Feb. 17, 2022

Signature page

A randomized, double-blind, dose-increasing, (blank) controlled clinical safety and tolerability trial of periodontal initial therapy combined with DPSCs injection for chronic periodontitis

Declaration and signature of relevant personnel

Data Management and Statistical Analysis Entity

We participated in the discussion and revision of this clinical trial protocol, and we will conduct the clinical trial in accordance with the provisions of this clinical trial protocol. During the clinical trial process, we will fulfill our obligations as an Data Management and Statistical Analysis Entity in accordance with the provisions of the *Good Clinical Practice (GCP), Management Measures for Stem Cell Clinical Research (Trial)* and relevant laws and regulations, follow relevant guiding principles for data management and statistical analysis, and ensure the data and statistical analysis results are authentic, scientific and standard.

Data Management and Statistical Analysis Entity: Clinflash Healthcare Technology (Jiaxing) Co., Ltd.

Project leader: Hu Zhenwei

Project leader (Signature): Hu Zhenwei (signature) Date: Feb. 11, 2022

Contact List

Applicant: Beijing SH Biotechnology Co., Ltd., Beijing 100070, China
Project leader: Wang Hua
Position: Investigator
Phone: 18511712135
Email: 18511712l35@l63.com
Address: Floor 2, Building 2, Yard 8, Haiying Road, Fengtai District, Beijing

Applicant: Capital Medical University
Medical Leader: Wang Songling
Position: Vice President
Phone: 13601324511
Email: slwang@ccmu.cdu.cn
Address: No.10, Youanmenwai, West 1st Alley, Fengtai District, Beijing

Research Center: Department of Stomatology, Peking University Third Hospital, Beijing 100191, China
Principal Investigator: Wang Xiao
Position: Director of Stomatology Department
Phone: 13601067229
Email: bysywangxiao@l63.com
Address: No. 49, Huayuan North Road, Haidian District, Beijing

Research Center: Department of Stomatology, Peking University Third Hospital, Beijing 100191, China
Principal Investigator: Li Haiyan
Position: Director of Drug Clinical Trial Institution
Phone: 13910405602
Email: Iuuyanlil027@hotmail.com
Address: No. 49, Huayuan North Road, Haidian District, Beijing

Contract Research Organization: Tianjin GoalGen Biotechnology Co., Ltd.
Project leader: Zhang Tieqiang
Position: Project Manager
Phone: 18622504391
Fax: 022-25322880-800S
Email: zhanglieqiang@goalgen.com
Address: Floor 5, Building 2, TEDA Group Science & Technology Development Center, Intersection of Dongting 1st Street and Dongting Road, Tianjin Economic Developing Area, Tianjin

Data Management and Statistical Analysis Entity: Clinflash Healthcare Technology (Jiaxing) Co., Ltd.
Project leader: Hu Zhenwei
Position: Head of Data Management Department, Data Coordinator (Level II)
Phone: 13586353387
Email: zhenwei.hu@clinflash.com
Address: Floor 4, TigerMed Building, No. 28, Huixin Road, Nanhu District, Jiaxing City, Zhejiang Province

Table of Contents

Program Summary 1

List of Acronyms 12

1. Background information 13

1.1 Disease and treatment background 13

1.2 Clinical study drug background 13

1.2.1 Basic information of the species 13

1.2.2 Overview of pharmacologic study information 14

1.2.3 Overview of pharmacologic-toxicologic studies 14

1.2.4 Relevant data from previous human studies 21

1.2.5 Clinical indications 21

2. Purpose of clinical studies 21

3. Clinical Study Design 22

3.1 Rationale for program design 22

3.1.1 The results of the previous pharmacological and toxicological studies of hDPSCs injection. 22

3.1.2 The idea of program design based on the results of the previous study dose escalation. 22

3.2 Study Design and Sample Size 23

3.2.1 Dose Escalation 23

3.2.2 Randomized, double-blind (blank) control 25

3.2.3 Sample Size 26

4. The Clinical Study Population 26

4.Clinical study subjects 26

4.2 Inclusion criteria 26

4.3 Exclusion criteria 26

4.4 Withdrawal (Shedding) Criteria 28

4.5 Middle/termination criteria 28

4.6 Treatment of Shedding and Termination of Study Subjects 28

5. Clinical research process 29

5.1 Screening period (D-28～D-9) 29

5.2 Basic periodontal treatment of the contralateral half of the study teeth (D-8±2)30 30

5.3 Issuance of randomization number (D-1) 30

5.4 Pharmacologic treatment (D1) 30

5.4. Examination within 2h prior to drug administration 31

5.4.2 Basic periodontal treatment with drug administration 31

5.5 Treatment observation (D1~D2) 32

5.6 Follow-up 33

5.6.1 Post-treatment follow-up (D7±1D,D14±1D,D30±3D, D90±7D,D180±14D) 33

5.6.2 Long-term follow-up (D360±14D, D720±30D) 34

5.7 Unplanned follow-up and treatment 34

5.8 Adverse event follow-up 35

5.9 Evaluation indicators 35

5.9.1 Safety indicators 35

5.9.2 Efficacy indicators 36
5.10 Endpoint indicators 36
5.11 Combined medications 37
6. Safety evaluation 37
6. Safety evaluation indicators 37
6.2 Adverse events, serious adverse events 38
6.2.1 Definitions 38
6.2.2 Collection and recording of adverse events 38
6.2.3 Criteria for grading the severity of adverse event 39
6.2.4 Assessment of the relevance of adverse events to the medicines used in the trial 39
6.3 Serious adverse events 40
6.3.1 Definitions 40
6.3.2 Reporting of serious adverse events 41
6.4 Assessment of clinical laboratory test abnormalities and other abnormalities considered as adverse events or serious adverse events 42
6.5 Suspected and Unanticipated Serious Adverse Reactions (SUSAR) 43
6.5.1 Definition of SUSAR 43
6.5.2 SUSAR Reporting 43
6.6 Pregnancy 44
7. Trial drug administration 44
7.1 Basic principles of drug management 44
7.2 Basic information on medicines 44

7.3 Preservation of medicines 44

7.4 Transportation of medicines 45

7.5 Medication Use, Recall and Records 45

8. Data management 45

8.1 Electronic case report form (eCRF) design 45

8.2 Database creation and testing 45

8.3 Data verification 45

8.4 Data entry, verification and query answering 46

8.5 Medical coding 46

8.6 Data locking 46

8.7 eCRF archiving and data management report. 46

9.Code of Ethics and Informed Consent 46

9.1 Code of Ethics 46

9.2 Informed Consent 47

10.Statistical analysis 47

10.1 Analyzing the population 47

10.2 Methods of analysis 47

11.Clinical trial risk management 48

11.1 Possibility of Success and Failure Analysis 48

11.2 Risk control 48

11.3 Risk treatment 49

12.Expected timeframe for clinical trial completion 49

Attachment 1: References 50

Program Version Revision Information

Serial number	Program No.	Version Number	Version Revision Date
1	SH-hDP-MSC-101	V1.0	2017.10.11
2	SH-hDP-MSC-101	V2.0	2020.06.17
3	SH-hDP-MSC-101	V3.0	2020.12.22
3	SH-hDP-MSC-101	V3.1	2021.01.18
4	SH-hDP-MSC-101	V3.2	2021.05.20
5	SH-hDP-MSC-101	V3.3	2022.02.11

Program summary

Applicant: Beijing SH Biotechnology Co.,Ltd., Beijing 100070, China<br>Capital Medical Univesity	
Tral drug: Human dental pulp mesenchymal stem cells injection	
Program No.: SH-hDP-MSC-101 V3.3	Study Stage: Phase I
Study Center	1
Study population: subjects with chronic periodontitis	

Study Objective:

Primary Objective

- To explore the safety and tolerability of hDPSCs for the treatment of chronic periodontitis.

Secondary Objective

- Dosage exploration to provide the basis of dosing regimens for subsequent clinical studies.
- To explore the preliminary effectiveness of hDPSCsin the treatment of chronic periodontitis.

Study Design:

This was a dose-escalation, randomized, double-blind, (blank) controlled study with 36 eligible subjects selected for enrollment into the study. There were 5 dose groups, 4 subjects in the first dose group, and 8 cases in each of the remaining 4 dose groups, for a total of 36 cases enrolled; each dose group was randomly assigned to the experimental group or the blank control group according to 3:1. Subjects were discharged from the hospital for 24 h of observation after administration of a single local injection, and the study was concluded at 6 months (D180±14D) after administration, with clinical safety and efficacy indices collected from the subjects. Long- term follow-up was conducted at the 12th month after administration (D360±14D) and 24th month after administration (D720±30D) to collect clinical efficacy observations.

(1)Dose Increment

①Starting dose design

The data of preclinical toxicology study of this product show that the hDPSCs were injected into the periodontal tissues repeatedly at the dose of $0.25x10^6$~$2.5x10^6$cells/kg and intravenously injected into the Bama minipig repeatedly at the dose of $2.5x10^6$ cells/kg, and the test indexes did not show any abnormal changes related to the injection of the drug. It is suggested that the NOAEL for tissue injection or intravenous infusion of hDPSCs in PNG pigs is $2.5x10^6$ cells/kg, which is about 15 times of the proposed clinical dose. Meanwhile, in view of the limited proliferation or differentiation potential and relatively low immunogenicity of MSC products, a safety factor of 10 was set for a single administration of $1.50x10^6$ cells/person /dose at 60kg (body weight of each personwas set at 60kg) in a comprehensive analysis. According to the design of the clinical study of similar drugs, the dosage used in reference to the clinical research literature: ① DPSCs in patients with bone defects caused by periodontal disease in a single administration dose of $5.0x10^6$ cells / person / times ； 《Retrieval of a periodontally compromised tooth by allogeneic grafting of mesenchymal stem cells from dental pulp: a case report》 [1], ②DPSC was administered locally at a dose of $1.0x10^6$ cells/person/

dose in patients with chronic periodontal disease; Study of Local Periodontal Regeneration of Chronic Periodontal Disease Patients Receiving Allogenic Human Dental Stem Cells Injection Therapy>[2].

According to the available clinical data of this product, all 13 subjects received basic periodontal treatment combined with a single local injection of hDPSCs, administered doses ranging from $1x10^7$ to $8x10^7$ cells, and were observed for 18/24/48 months of safe follow-up, with data suggesting that the safety of DPSCs ranging from 10^7 to $8x10^7$ cells was well tolerated.

To summarize, the starting dose of this study is proposed to be $x10^6$ cells/person/times.

②Dose progression program

There are 5 dose groups in the study, 4 subjects in the first dose group, and 8 subjects in each of the remaining 4 dose groups, with a single local injection. The range of dose increment is tentatively set as:

First dose group: $1x10^6$ cells/periodontal defect site.

Second dose group: $5x10^6$ cells/periodontal defect site.

Dose group 3: $1x10^7$ cells/periodontal defect site.

Dose group 4: $1x10^7$ cells/periodontal defect site, totaling 2 periodontal defect sites, with a total cell injection volume of $2x10^7$ cells/2 periodontal defect sites.

Dose group 5: $1x10^7$ cells/periodontal defect site, a total of 3~4 periodontal defect sites, the total cell injection volume was $3x10^7$~$4x10^7$ cells/3~4 periodontal defect sites.

Subjects were sequentially enrolled from the low dose group to the high dose group in accordance with the principle of dose escalation, with each subject receiving only one corresponding dose. Four subjects were enrolled in the first dose group and randomly assigned to the experimental group (3 cases) or the blank control group (1 case); 8 subjects were enrolled in each of the remaining 4 dose groups and randomly assigned to the experimental group (6 cases) or the blank control group (2 cases) to carry out the safety and tolerability study of a single administration. During the course of the study, at the investigator's discretion, subjects will be entered into the next dose group if they do not experience an adverse event related to meeting the criteria for termination of the dose escalation within 2 weeks of receiving an injection of the test drug. The investigator will determine the subsequent trial schedule based on the safety profile of each subject

3.Termination of Dose Escalation Criteria

The degree of adverse events observed in the study is determined by the NCI CTCAE v5.0 grading criteria. Dose escalation will be terminated and the previous dose will be defined as the MTD if the following occurs within 2 weeks of receiving an injection of the test drug

① Half or more of the subjects in either dose group experience a Grade 3 or higher drug-related adverse event;

② If the maximum dose ($3x10^7$ to $4x10^7$ cells/3 to 4 periodontal defect sites) is reached without half or more of the subjects experiencing grade 3 or higher adverse events potentially related to the test drug, the investigator, in consultation with the sponsor, decides whether it is necessary to continue the dose escalation; if the dose escalation is not continued this dose group will be defined as the MTD.

④ Treatment of Shedding Subjects

If a subject is dislodged within 4 weeks of receiving the test drug injection, the subject will be replenished in the appropriate dose group; if dislodged after 4 weeks, the subject will not be replenished.

(2) Randomized, double-blind

①Randomization
In this study, a complete randomization method was used to generate the random table and the groups corresponding to the random table using SAS (version 9.4 or above) software, and the random number was assigned using the Electronic Central Randomization System for Clinical Trials (IWRS). Each dose group was randomized separately. Each eligible subject was given a randomization number on D-1 according to screening number from smallest to largest and entered into the corresponding group.

② Double-blind
To minimize or control for bias, this study used a double-blind design. There exists slightly different injection appearance of the test drug and the blank control drug due to the dosage form. A non-blinded panel will be set up in this study to ensure that other relevant researchers remain blinded for the duration of the study. The unblinded team members include: unblinded physicians, unblinded nurses, unblinded CRC, unblinded drug administrators, and unblinded supervisors, who will be responsible for receiving, distributing, administering, retrieving, documenting, and data monitoring of the trial drug. The unblinded team members will not perform any assessments during the study. All study physicians were required to undergo concordance evaluation before participating in the study, and members with a concordance score of ≥ 85% were allowed to proceed to the next step of the study.
To better maintain blinding, non-blinded physicians should use simulated blinding of subjects (e.g., use of eye masks).

(3) (Blank) control
Test drug: a single local injection of hDPSCs given on top of basic periodontal treatment (supragingival scaling, subgingival scraping and root planing);
Blank control: a single local injection of saline given on the basis of basic periodontal treatment (supragingival scaling, subgingival scraping and root planing).

Sample size: 36 eligible subjects with chronic periodontitis.

Inclusion Criteria:
Subjects must meet all of the following criteria to be enrolled in the study:
(1) Age 18-65 years old (including the threshold) and gender unlimited;
(2) Periodontal defect sites radiologically detected as vertical-type bone defects, and the probing depth (PD) of the periodontal defect sites is 4-8 mm;
(3) Voluntarily participated in this clinical study, understood and signed the informed consent form, and voluntarily complied with the relevant regulations of this study during the study period and 18 months after the end of the study.

Exclusion Criteria:
Subjects who fulfill any of the following will be excluded from this study:
History of local/systemic treatment:
(1) Subjects with severe periodontal disease (alveolar bone resorption generally exceeding two-thirds of the root length) that interferes with the judgment of the tooth;
(2) Study teeth with ≥ 2 degrees of looseness (dental forceps clamping method: 1 degree for buccolingual movement only; 2 degrees for both buccolingual and proximal-distal-medial movement; 3 degrees if vertical looseness is present);
(3) Those with previous surgical treatment of periodontal bone defect sites and adjacent periodontal tissues;
(4) Those who have been treated with non-steroidal anti-inflammatory drugs, steroidal (steroidal) hormones within 3 months prior to screening, and/or those who have used hormones (except topical topical hormones) within 3 months prior to screening, and those who have used bisphosphonates within 3 months prior to screening;
congenital heart disease, etc.);

(5) Subjects with severe systemic infections within 3 months prior to screening; or antibiotic use within 72 h prior to screening;

History of co-morbidities/conditions:

(6) Those with uncontrolled hypertension (definition: systolic blood pressure ≥ 160 mmHg or diastolic blood pressure ≥ 95 mmHg in the seated position of the subject after receiving an optimal regimen of antihypertensive therapy) within 1 month prior to screening;

(7) Persons with systemic systemic diseases (including, but not limited to: patients with malignant tumors or those with positive tumor tests during the screening process diabetic patients, patients with cardiac disease resulting in heart failure, those who have had a myocardial infarction within six months prior to screening, those who have developed symptoms of angina pectoris within six months prior to screening, and patients with congenital heart disease, etc.);

History of allergies:

(8) Persons known to be potentially allergic to any of the materials used in the treatment process;

(9) Persons with allergies, previous history of allergy to blood products;

Laboratory Tests:

(10) Laboratory tests (any one of which meets): abnormal liver function: ALT>80U/L or AST>70U/L, abnormal renal function: blood creatinine (picric acid method) >97umol/L

(11) Individuals with bleeding tendency or coagulation disorders (International Normalized Ratio (INR) ≥ 1.5 times ULN or Activated Partial Thromboplastin Time (APTT) ≥ 1.5 times ULN (except for patients on anticoaguant therapy)) or severe hematologic disorders (e.g., grade 3 or greater anemia (Hb < 80 g/L)); grade 2 or greater platelet decreased (<75.0×10^9/L);

(12) Positive serum virology (HBsAg, HCV antibody, HIV antibody, syphilis spirochete antibody) test positive individuals;

History of childbearing:

(13) Those who have had unprotected sexual intercourse within 1 month prior to screening;

(14) Pregnant or breastfeeding women, or those who tested positive for human chorionic gonadotropin beta (β-HCG) prior to screening, or those who are unable and unwilling to use investigator-approved contraception as directed by the investigator for the duration of the vestigator for the duration of the study and for 6 months after the study;

(15) Women who apply oral contraceptives for contraception;

Others:

(16) Subjects with a history of tobacco addiction (≥ 10 cigarettes per day) in the 12 months prior to screening;

(17) Subjects with mental or consciousness disorders;

(18) Subjects who have participated in other clinical studies within 3 months prior to screening;

(19) Other conditions deemed inappropriate for participation by the investigator.

Withdrawal (Shedding) Criteria:

(1) Subjects experienced an adverse event during the course of the study that, in the opinion of the investigator, precludes continuation of the study;

(2)Subjects have poor compliance and are unable to complete follow-up visits on time;

(3) Use of other medications (e.g., glucocorticoids, bisphosphonates, estrogens, etc.) that, in the judgment of the investigator, interfere with tolerability or safety judgments;
etc.) that, in the judgment of the investigator, affect tolerability or safety judgment;

(4) Use of other foods (e.g., betel nut, beefsteak, etc.) that, in the judgment of the investigator, affect tolerability or safety judgment;

.

(5) Subjects are unwilling to continue with the clinical study and withdraw from the investigator;
(6) Female subjects become pregnant during the course of the study;
(7) Loss to visit.

Mid/Termination Criteria:

The study may be suspended or terminated early for good cause, which may include, but is not limited to:
(1) A toxic reaction during the course of the study that meets the criteria for termination dose escalation;
(2) The sponsor's own reasons such as insufficient funding, patent disputes, or changes in national pharmaceutical development policies;
(3) Suspension or termination of the approved study at the request of the drug regulatory authority or ethics committee.

In addition, the sponsor reserves the right to suspend or terminate the study at any time.

Once the above mentioned issues of drug safety, protocol compliance, etc. that caused the suspension or termination of the study have been resolved and agreed upon by the sponsor, the Ethics Committee and the regulatory body, the study may continue.

Any party that decides to suspend or terminate the study shall immediately give written notice to the other parties (including, but not limited to, the sponsor, the investigator, the ethics committee, and the regulatory body) and provide the relevant reasons.

Combined medications

Throughout the trial, the investigator will be provided with information regarding any therapeutic interventions, surgical procedures, etc., that accompany the diseases and medications, and where possible, the diagnosis of all diseases and the date of onset of all diseases and date of remission of symptoms, as well as the name of the medications included, the date of administration, and a adescription of the surgery.

Any medication taken prior to the test drug administration will be considered concomitant, and any other medication taken after the administration of the test drug will be considered comorbid, and the name of the medication, the reason for the medication, and the date of the comorbid/concomitant medication will also be recorded. Subjects should be asked about their co-medication/concomitant medication at each follow-up visit.

In the event of an AE or SAE, it will be treated aggressively and the medication used will be recorded on the eCRF.Subjects may receive other treatment as determined by the investigator after 3 months of treatment with the test medication based on the condition of the contralateral tooth, and after 6 months of treatment with the test medication as determined by the investigator based on the condition of tooth.

Study steps:

(1) Screening period (D-28 to D-9)

After subjects signed the informed consent form, they underwent various screening examinations at the clinic, and subjects who met all the inclusion criteria and those who did not meet any of the exclusion criteria, as judged by the investigator, were allowed to enter the next phase of the study. The subjects underwent basic periodontal treatment (saline rinsing and gargling, full-mouth ultrasonic supragingival cleaning and polishing, and saline rinsing) by a blinded physician.

(2) Periodontal basic treatment in the half of the mouth contralateral to the study tooth (D-8±2)

Subjects returned to the hospital on D-8±2, and a blinded physician performed periodontal basic treatment (4% articaine, half-mouth ultrasonic subgingival scaling, root planing, polishing, and saline rinsing) on the half-mouth opposite the study tooth of the subject, and completed a full-mouth periodontal examination of the following periodontal clinical indicators within 2 h prior to receiving the periodontal basic treatment on the half-mouth opposite the study tooth: periodontal attachment level AL, periodontal probing depth PD, probing bleeding index BI, gingival recession GR, and tooth mobility TM. (The PD and TM examinations at this visit point were only used as a baseline record

and were not used as an inclusion criterion)

(3) Issuance of Randomization Number (D-1)

The investigators check the inclusion/exclusion criteria on subjects who completed the relevant examinations on D-1, and subjects who met all inclusion criteria and those who did not meet either exclusion criterion were enrolled in the study, and a randomization number was issued to all enrolled subjects.

(4) Medication (D1)

Subjects receive periodontal basal therapy (4% articaine, ultrasonic subgingival scraping of the study tooth side half of the mouth, root planing and polishing, and saline rinsing) in the study tooth side half of the mouth on D1, and completed within 2 h prior to receiving the basal therapy: vital signs (respiration, heart rate, blood pressure (systolic blood pressure, diastolic blood pressure), and temperature); and laboratory examination (blood routine, coagulation, liver and kidney functions, inflammatory index tests, immunological tests, urine routine).

After completion of the examinations, the blinded physician performed periodontal basic treatment (4% articaine, ultrasonic subgingival scraping of the study tooth side half of the mouth, root planing and polishing, and saline rinsing). Local injections administered to subjects by a non-blinded practitioner immediately after completion of basic periodontal treatment with the medication assigned by the randomization methodology.

This was done as follows:

Experimental drug treatment: the 1st to 3rd dose groups were injected locally with 0.6 ml of hDPSCs injection into a single periodontal defect site in the subject's mouth/periodontal defect site, the 4th dose group was injected locally with 0.6 ml of hDPSCs injection into two periodontal defect sites on

the same side of the subject's mouth/periodontal defect site, and the 5th dose group was injected locally with 0.6 ml of hDPSCs injection into three or four periodontal defect sites on the same side of the subject's mouth/periodontal defect site, respectively. 0.6 ml hDPSCs injection /periodontal defect site was injected, and the concentrations administered in the dose groups were:

First dose group: $1x10^6$cells/periodontal defect site.

Second dose group: $5x10^6$ cells/periodontal defect site.

hird dose group: $1x10^7$ cells/periodontal defect site.

Fourth dose group: $1x10^7$ cells/periodontal defect site, totaling 2 periodontal defect sites, with a combined cell injection volume of $2x10^7$ cells/2 periodontal defect sites.

Fifth dose group: $1x10^7$ cells/periodontal defect site, totaling 3-4 periodontal defect sites, with a combined cell injection volume of $3x10^7$~$4x10^7$/3-4 periodontal defect sites.

Blank control treatment: local injection of saline 0.6ml/periodontal defect site.

(4) Treatment observation (D2)

After the treatment, the subjects will be observed in the therapeutic room for 30 min and then transferred to the study ward for 24-hour clinical observation and safety assessment.

(5) Follow-up (D7±1D, D14±1D, D30±3D, D90±7D, D180±14D, D360±14D, D720±30D)

Post-treatment follow-up: Subjects were followed up once each on D7±ID, D14±1D, D30±3D, D90 ±7D, D180±14D after leaving the hospital.Clinical safety and efficacy indicators were collected from the subjects and the study was concluded

Long-term follow-up: one follow-up visit at month 12 (D360±14D) and one follow-up visit at month 24 (D720±30D) after the administration of the drug to collect clinical efficacy observations.

Evaluation Indicators:

1) Safety indicators

①Vital signs

Respiration, heart rate, blood pressure (systolicblood pressure, diastolic blood pressure),temperature. Checked during the screening period, within 2h before administration, 30min±5min, 2h±5min, 24h±30min after dministration, and D7±1D, D14±1D, D30±3D, D90±7D, and D180±14D during the follow-up period.

②Laboratory examination

-Blood routine: hemoglobin, red blood cells, white blood cells, neutrophil count, lymphocyte count, and platelet count. Checked during the screening period, within 2h before administration, 24h±30min after administration, and D7±1D, D14±1D, D30±3D, D90±7D, and D180±14D during the follow-up period.

-Coagulation function: including prothrombin time (PT), activated partial thromboplastin time (APTT), and international normalized ratio (INR). Checked during the screening period, within 2h before drug administration.

-Hepatic and renal function: total bilirubin, direct bilirubin, alanine aminotransferase, aspartate aminotransferase, total protein, albumin, total bile acids, urea, creatinine, uric acid, glucose, potassium, sodium, chloride.Examined during the screening period, within 2h prior to administration , 24h±30min after administration and D7±ID, D14±1D, D30±3D, D90±7D, and D180±14D during the follow-up period.

-Inflammatory index test: ultrasensitive C-reactive protein. Examined during the screening period, within 2h before administration, and 2h±5min after administration.

-Infectious disease test: HBsAg, HBsAb, HBcAg, HBcAb, HBcAb, Anti-HCV, HIVcombin, Anti-TP, CMV-IgM, CMV-IgG, examined only during the screening period, and D30±3D, D90±7D, D180±14D during the follow-up period .

-Immunological tests: IgA, G, IgM, total IgE; examined during the screening period, within 2h prior to administration, 24h±30min after administration, and D7±1D, D14±1D, D30±3D, D90±7D during the follow-up period .

-Pregnancy test: female subjects; examined during the screening period, issuance of randomization number D-1, follow-up period D30±3D, D90±7D, D180±14D. (Blood pregnancy only during the screening period, urine pregnancy test for the rest.)

-Urine routine: examined during the screening period, within 2h before drug administration, within 2h after drug administration, and 24h±30min.

-Stool routine: examined during the screening period, within 24h after drug administration.

-Electrocardiogram: examined during the screening period, issuance of randomization number D-1, 2h±30min, 24h±30min after drug administration, and D7±1D, D14±1D, D30±3D, D90±7D, and D180±14D during the follow-up period.

③Adverse events and serious adverse events

The occurrence of adverse events and serious adverse events was recorded in a timely manner during the study period, and the degree of adverse events was determined according to the NCI CTCAE v5.0 grading criteria.

(2) Main efficacy indicators

Oral clinical test indicators: periodontal attachment level AL (periodontal probing depth PD + gingival recession GR), using the full mouth clinical test index collected at the time point of the periodontal basic treatment (D-8±2) in the opposite half of the mouth of the study tooth as the baseline index.

(3) Secondary efficacy indicators

Radiologic testing indicators: CBCT examination with screening period indicators as baseline indicators.

Height of periodontal bone defect

Mean density of alveolar ridge

Oral clinical test indices: periodontal probing depth (PD), periodontal healing (probing bleeding index BI, gingival recession GR, tooth mobility TM), and full mouth clinical test indices collected at the time point of periodontal basal treatment (D-8±2) on the contralateral half of the study teeth were used as baseline indicators.

Study Endpoints

Primary Endpoint Indicator:

Safety Endpoint Indicators

-Incidence and severity of adverse events (AEs) associated with pulpal MSC therapy within 180 days of administration;

Secondary Endpoint Indicator:

Safety Endpoint Indicator.

-Adverse events (AEs) and serious adverse events (SAEs) related to pulpal MSC therapy within 360 days and 720 days after dosing incidence(SAE) and severity:

Efficacy Endpoint Indicators

-Change in periodontal attachment level (AL) from baseline on day 90 after administration;

-Change from baseline in periodontal probing depth (PD) on day 90 after dosing;

-Change from baseline in periodontal healing (probing bleeding index (BI), gingival recession (GR), tooth looseness (TM)) on day 90 after dosing.

-Change from baseline in periodontal bone defect height on day 180 after dosing;

-Change from baseline in mean alveolar ridge density on day 180 after drug administration.

Statistical considerations:

The results of this study were mainly described statistically. Measures were listed as mean, standard deviation, median, maximum, and minimum values, and counts and ranks were listed as frequency (constitutive ratio), rate, and 95% confidence interval.

Descriptive statistics on demographic and baseline characteristics are provided by describing the number of cases of subjects enrolled in each dose group, dislodged and rejected.

Evaluation of safety and tolerability: Descriptive statistical analysis was used to tabulate the adverse events and adverse reactions that occurred in each dose group of the study (where adverse reactions were defined as "definitely related, probably related, probably related, probably not related" to the test drug).

Laboratory test results describing pre-study normal but post-dose abnormalities. Vital signs (blood pressure, heart rate, body temperature) and laboratory parameters for each dose group before and after administration were calculated separately as mean, standard deviation, median, minimum and maximum values.

Interim analysis:

This study proposes to conduct an interim analysis after 36 subjects have completed dosing and D180 follow-up to analyze the enrolled subjects' efficacy and safety data and complete an interim analysis report.

All statistical analyses will be calculated using SAS 9.4 statistical analysis software programming.

A randomized, double-blind, dose-escalation, (blank)-controlled, safety, and tolerability clinical trial of periodontal basic therapy combined with human pulpal mesenchymal stem cell injections for the treatment of chronic periodontitis

X	Visiting Program	Enrollment and treatment period					Follow-up							
							Post-treatment follow-up						Long-term follow-up	
		Screening period	Periodontal Basis Treatment for the Contralateral Half of the Study Teeth	Issuance of randomization numbers	Medication	Treatment observation [11]	Follow-up visit 1	Follow-up visit 2	Follow-up visit 3	Follow-up visit 4	Follow-up visit 5	Early withdrawal	1st long term follow up	2ed long term follow up
		D-28~D-9	D-8±2	D-1	D1[17]	D2[17]	D7±1D	D14±1D	D30±3D	D90±7D	D180±14D		D360±14D	D720±30D
1	Signing the ICF	X												
2	Inclusion/Exclusion Criteria	X	X	X										
3	Demographic information[1]	X												
4	Medical history[2]	X												
5	Vital signs: respiration, heart rate, blood pressure (systolic, diastolic), temperature	X			X	X	X	X	X	X	X	X		
6	Periodontal clinical index examination: Periodontal attachment level AL, periodontal probing depth PD, probing bleeding index BI, gingival recession GR, tooth missing TM[3]		X							X	X	X	X	X
7	Routine blood tests[4]	X			X	X	X	X	X	X	X	X		
8	Coagulation function[5]	X			X									
9	Liver and kidney function[6]	X			X	X	X	X	X	X	X	X		
10	Inflammatory indicators: ultrasensitive C-reactive protein[7]	X			X									
11	Infectious disease tests: HBsAg, HBsAb, HBeAg, HbcAb, 1lbcAb, Anti-HCV , HIVcombi, Anti-TP, CMV-IgM, CMV-IgG	X							X	X	X	X		
12	Immunologic tests: lgA, IgG, IgM, total IgE	X			X	X	X	X	X	X				
13	Pregnancy test[8]	X		X					X	X	X	X		
14	Urine routine[9]	X			X	X								
15	Stool routine[10]	X				X								
16	Randomization			X										
17	Basic periodontal treatment	X												

	supragingival scaling[12]													
18	Basic periodontal treatment (subgingival scaling, root planing)		X		X									
19	Trial administration of treatment				X									
20	Record of adverse events[13]		X		X	X	X	X	X	X	X	X	X	X
21	Record of combined medication/treatment[14]	X			X	X	X	X	X	X	X	X	X	X
22	Imaging (CBCT)[15]	X								X	X	X	X	X
23	Electrocardiogram[16]	X		X	X	X	X	X	X	X	X	X		

Notes:

(1) Demographic information: including sex, date of birth, age in weeks, ethnicity, height, weight.

(2) Medical history information: current medical history: diagnosis, symptoms; history of allergies, family history, past history (e.g., history of smoking, diabetes mellitus, other surgical procedures, etc.).

(3) Periodontal clinical index examination: periodontal attachment level AL, periodontal probing depth PD, probing bleeding index BI, gingival recession GR, and tooth looseness TM. The examinations were performed by a blinded practitioner within 2 h prior to the periodontal foundation treatment of the contralateral half of the oral cavity of the study tooth, D90±7D, D180±14D, D360±14D, and D720±30D. The whole mouth clinical test indexes collected at the time point of periodontal basic treatment (D-8±2) in the opposite half of the mouth of the study teeth were used as the baseline indexes (the PD and TM examinations at this visit point were only used as the baseline values and were not used as the criteria for the enrollment determination), and the efficacy indexes were observed up to D180±14D.

(4) Blood routine: including hemoglobin, red blood cells, white blood cells, neutrophil count, lymphocyte count and platelet count. It was checked during the screening period, within 2h before administration, 24h±30min after administration, and D7±1D, D14±1D, D30±3D, D90±7D, and D180±14D during the follow-up period.

(5) Coagulation function: prothrombin time (PT), activated partial thromboplastin time (APTT), international normalized ratio (INR). Examined during the screening period, within 2h before drug administration.

(6) Liver and kidney function: total bilirubin, direct bilirubin, alanine aminotransferase, aspartate aminotransferase, total protein, albumin, total bile acids, urea, creatinine, uric acid, glucose, potassium, sodium, chloride. D7±1D, D14±1D, D30±3D, D90±7D, and D180±14D were examined during the screening period, within 2h prior to administration, and 24h±30min after administration, and during the follow-up period D90±7D.

(7) Inflammation index test: ultrasensitive C-reactive protein, reference value of ultrasensitive C-reactive protein in whole blood of healthy adults <5mg/L. Checked during the screening period, within 2h prior to the administration of the drug, and 2h±5min after the administration of the drug.

(8) Pregnancy test: female subjects; examined during the Screening Period, issuance of randomization number D-1, and during the follow-up period D30±3D, D90±7D, D180±14D. (Blood pregnancy for screening period only, urine pregnancy test for others.)

(9) Urine routine: including urine specific gravity, pH, urine glucose, urine protein, urine leukocytes (qualitative), urine ketone bodies, bilirubin, urine occult blood (qualitative). It was checked during the screening period, within 2h before drug administration, within 2h after drug administration, and 24h±30min.

(10) Stool routine: including fecal character, fecal leukocytes, fecal erythrocytes, fecal color, fecal occult blood test (OBT). Examined during the screening period, 24h after drug administration.

(11) Therapeutic observation period, for 24h after the subject receives the test drug injection.

(12) Before performing periodontal basic treatment (supragingival scaling) during the screening period, the subjects were required to be tested for periodontal probing depth PD and tooth looseness TM. the PD and TM examinations were used as the enrollment criteria for determination.

(13) Adverse event recording: only ADRs are recorded for adverse events of D360±14D and D720±30D.

(14) Recording of co-medication/treatment: D360±14D, D720-30D only record co-medication for ADR.

(15) Imaging (CBCT): during the screening period, D90±7D, D180±14D, D360±14D, D720±30D were examined during the follow-up period. Screening period indicators were used as baseline indicators, and efficacy indicators were observed up to D180±14D. Data collection was performed by the Third Hospital of Peking University and analyzed by Beijing Stomatological Hospital of Capital Medical University.

(16) Electrocardiogram: examined during the screening period, issuance of random number D-1, 2h±30min, 24h±30min after drug administration, and D7±1D, D14±1D, D30±3D, D90±7D, and D180±14D in the follow-up period.

(17) The specific time for vital signs check here is: within 2h before administration, 30min±5min, 2h±5min, 24h±30min after administration; the specific time for immunological check is: within 2h before administration, 24h±30min after administration.

List of Abbreviations

Abbreviations	Full Noun	Abbreviations	Full Noun
ADR	Adverse Reaction	HBsAb	Hepatitis B Surface Antibody
AE	Adverse Event	HBsAg	Hepatitis B Surface Antigen
AL	Attachment Level	HIV	Human Immunodeficiency Virus
Anti-HCV	Anti-Hepatitis C Virus Antibody	HIVcombin	HIVcombin
Anti-TP	Syphilis Antibody	IB	Investigators' Handbook
APTT	Activated Partial Thromboplastin Time	ICR	Institute of Cancer Research
BI	Bleeding Index	INR	International Normalized Ratio
β-HCG	Human Chorionic Gonadotropin β	IWRS	International Central Randomized System
CBCT	Cone Beam Computed Tomography	MedDRA	International Dictionary of Medical Terms
CDE	Drug Evaluation Center	MSC	Mesenchymal Stem Cells
CMV-IgG	Cytomegalovirus antibody immunoglobulin G	MTD	Maximum Tolerance
CMV-IgM	Cytomegalovirus antibody immunoglobulin M	CTCAE	Common Terminology Criteria for Adverse Events
CRA	Clinical Auditor	NMPA	National Pharmaceutical Administration
CRC	Clinical Coordinator	NOD SCID	Non-Obese Severe Diabetes Mellitus Combined with Immunodeficiency
CRF	Clinical Record Reporting Form	OBT	Occult blood test in feces
CRO	Contract Research Organization	PD	Probing Depth
CS	Abnormalities of clinical significance	PLT	Platelets
DM	Data Manager	PPS	Compliance with protocol set
DPSC	Dental Pulp Stem Cells	PT	Prothrombin time
DMSO	Dimethyl sulfoxide	PV	Pharmacovigilance
eCRF	Electronic Clinical Record Report Form	SAE	Serious Adverse Events
FAS	Full Analysis Set	SAP	Statistical Analysis Program
GCP	Code of Practice for the Quality Management of Pharmaceutical Clinical Trials	SS	Safety Data Set
GMP	Good Manufacturing Practice	SUSAR	Unintended Serious Adverse Reactions
GR	Gingival Recession	TM	Tooth Mobility
HBcAb	Hepatitis B Surface Core Antibody	WBC	White Blood Cells
HBeAb	Hepatitis B E Antibody		

1. Background information

1.1 Background of disease and treatment

Periodontal disease is one of the two major diseases that jeopardize the oral health of human beings, and it is called the "incurable disease" of dentistry, and it is the first cause of tooth loss among adults in China [3]. Periodontal disease also increases the risk of systemic diseases such as cardiovascular disease, diabetes mellitus and rheumatoid arthritis. Periodontal disease includes gingivitis and periodontitis, and the ultimate goal of its treatment is the regeneration of periodontal tissues (alveolar bone, periodontal membrane, osteoid, and gingiva), however, at present, conventional clinical treatments such as basic periodontal therapy, periodontal surgery (periodontal flap surgery, guided bone regeneration, guided tissue regeneration) are difficult to obtain satisfactory tissue regeneration results. In recent years, the rapid development of stem cells and tissue engineering has provided new ideas for periodontal regeneration [4], in which mesenchymal stem cells (MSC) are from a wide range of sources, easy to culture and expand in vitro, and self-secrete a variety of biologically active molecules with immunomodulatory ability, anti-apoptosis, anti-inflammatory, anti-fibrotic, and pro-angiogenesis, and can be converged to the site of tissue trauma and inflammation, which make MSC widely used in the study of regenerative medicine. research in regenerative medicine is widely used, as of 2017, the clinical treatment studies of MSC conducted worldwide have amounted to more than 600 kinds [5-7] , and there are clinical trials registered bone marrow mesenchymal stem cells and oral mucosal stem cells for periodontal tissue regeneration research and periodontal disease treatment research (https: //www.clinicaltrials. gov/). MSC therapy has also been carried out in several hospitals in China for immune disorders and degenerative and injurious diseases. These studies have shown the very good safety of applying MSC to treat localized tissue lesions or systemic systemic diseases. In this project, preclinical and clinical studies have revealed that dental pulp MSCs have a very good effect in repairing periodontal defects based on their multidirectional differentiation and immunomodulatory ability. Therefore, we aim to develop a novel cellular drug for periodontitis treatment that can regenerate tissues and form new periodontal attachments.

1.2 Background of the clinical study drug

1.2.1 Basic information of the species

Drug name: human dental pulp mesenchymal stem cell injection.

Main ingredients: the active ingredient is human dental pulp mesenchymal stem cells, and the excipient is sodium chloride injection..1.5
Dosage form and specification:
-Dosage form: injection
-Specification: $1x10^7$ cells/0.6ml/branch.

This product is mainly used for the treatment of chronic periodontitis, such as periodontal bone tissue defects caused by chronic periodontitis.When human pulp mesenchymal stem cells are injected locally, the cells are planted in the bone pocket, differentiate into osteoblasts and secrete biologically active substances to promote regeneration of periodontal tissues; at the same time, it can reduce the infiltration of inflammatory cells and the release of inflammatory factors and play an anti-inflammatory role; thus, it achieves the therapeutic purpose of periodontitis.

1.2.2 Overview of pharmacological study data

Healthy permanent teeth were obtained from the hospital oral surgery clinic, the pulp was obtained by amputating the crown under GMP conditions, and the primary cells, called PO generation cells, were obtained by tissue block culture after digestion with collagenase I and dispase, and the PO generation cells were passaged, cultured, split, and quality-assessed, and the original and working cell banks were established, respectively. The production process of dental pulp mesenchymal stem cells was established, and three batches of cellular drugs were produced according to this process, and the finished products were subjected to quality checking, and the results of the checking were in accordance with the proposed quality standards, indicating that the production process of this product is stable. The results of preliminary stability experiments show that the cell stock solution can be stored under liquid nitrogen (-196) for a long time, and the cell stock solution can be stored stably for at least 36 months in the lyophilized solution containing 90% serum, and the validity period is tentatively set at 36 months when the lyophilized solution is 10% DMSO + 90% fetal bovine serum lyophilized solution; and the validity period is tentatively set at 3 months when the lyophilized solution is serum-free mesenchymal stem cell lyophilized solution. The cell preparation was stable within 10 hours of storage at 4.

The above studies show that the production process of human dental pulp mesenchymal stem cells is stable and the quality of the product is controllable, which is in accordance with the proposed quality standards and relevant regulations.

1.2.3 Overview of pharmacological and toxicological studies

1.2.3.1 Toxicity experiment of dental pulp MSCs given to ICR mice by single intramuscular and intravenous injections

The purpose of this experiment is to evaluate the acute toxic reaction produced by a single intramuscular and intravenous injection of a larger dose of dental pulp MSCs given to ICR mice, and to provide animal experimental information for clinical studies. Sixty ICR mice were used for the experiment, randomly divided into three groups according to sex segments, i.e., intravenous negative control group, intramuscular injection of the test article group, and intravenous injection of the test article group, with 20 mice and 20 females and 50 males in each group. The dose of the test group was $5x10^7$ cells/kg and the volume of drug administered was 25 mL/kg. the test group was observed continuously for 4 hours after the administration of the drug, and then once a day in the morning and once a day in the afternoon for 14 consecutive days. Animals were weighed at D1 (before administration), D8 and D15, and food intake was determined at D8 and D15. At the end of the observation period, the animals were euthanized for gross autopsy observation.

The viability of the prepared donor cell suspension was 92.6% and the viability was above 70%; the concentration of the donor was $2.05x10^6$ cells/mL with an accuracy of 103%, which was within 80% to 120% of the theoretical concentration ($2x10^6$ cells/mL). The viability and concentration of the formulated test material were within the acceptable range specified in the experimental protocol.

No significant abnormal reactions were observed in all groups of animals during 4 hours of continuous observation after administration; during the experimental period, no significant abnormal reactions related to the administration were observed clinically in all animals.

A statistically significant slow increase in D8 body weight was seen in males in the intravenous donor group compared to the negative control group during the same period ($p \leqslant 0.05$). Otherwise, no significant difference was seen in body weight and food consumption at $P \leqslant 0.05$ between animals in the intramuscular and intravenous donor groups.

Pathologic results showed no significant gross and microscopic toxic pathologic changes associated with the test supply.

Under the conditions of this experiment, dental pulp MSCs given to ICR mice by single intramuscular and intravenous injections had a maximum tolerated dose (MTD) greater than or equal to $5x10^7$ cells/kg.

1.2.3.2 Toxicity experiments of repeated periodontal tissue injections and intravenous drip administration of dental pulp MSCs to Bama minipigs for 4 weeks and 8 weeks of recovery period

The purpose of this experiment is to observe the possible toxic reactions and target organs after repeated periodontal tissue injections and intravenous drip injections of dental pulp MSCs given to Bama miniature pigs for 4 weeks, the colonization and distribution of dental pulp MSCs in the animal body, as well as the recovery of the toxic reactions or possible delayed toxic reactions at 8 weeks after stopping the drug, so as to provide animal experimental reference information for the clinical study of the test article.

For the experiment, 40 Bama miniature pigs, half male and half female, were randomly divided into 4 groups of 10 animals each according to sex section, respectively, the negative control group, the periodontal tissue injection of the test article low and high dose group and the test article intravenous drip group, the negative control group was given sodium chloride injection, the test article group was given dental pulp mesenchymal stem cells, the dose of the periodontal tissue injection group was $0.25x10^6$ and $2.5x10^6$ cells, respectively. The negative control group and the periodontal tissue injection group were given the control/supplier by periodontal tissue injection, and the intravenous drip group was given the supplier by subcutaneous vein in the limbs or by vein at the ear margin, and the drug was administered once every 2 weeks for 4 consecutive weeks, with a total of 3 administrations. During the experimental period, the animals were subjected to clinical observation and ophthalmologic examination, body weight, body temperature, electrocardiogram, hemocytometer IFN-y count, coagulation function, blood biochemistry, urine, anticellular antibodies, cytokines (TNF-α, IFN-γ, IL-10, and IL-6), and T-lymphocyte subpopulations ($CD3^+$, $CD4^+$, $CD8^+$, $CD4^+/CD8^+$), immunoglobulin (IgG, IgA and IgM), T lymphocyte proliferation and other indicators; toxicokinetic analysis of venous blood collected from animals in groups 1-4 after the first and third doses, and the study of colonization and distribution of dental pulp MSCs in animals 2 days after the last dose and at the end of the recovery period. The first 3 animals of each group, male and female, were euthanized 2 days after the last dose, and the remaining animals were euthanized 8 weeks after drug withdrawal. After euthanasia, animals were subjected to gross autopsy and weighing of major organs, and more than 40 major tissues and organs were examined histopathologically. One animal each from the negative control group and the periodontal tissue injection $2.5x10^6$ cells/kg dose group died on D24 and D67 during the experimental period, and the cause of death was respiratory dysfunction and respiratory failure due to spontaneous lung injury (including hemorrhage, edema, and diffuse infiltration of inflammatory cells, abscess, etc.).

Except for the dead animals, during the experimental period, no abnormal changes related to drug administration were observed in the clinical observation, body weight, body temperature, electrocardiogram, ophthalmologic examination, blood cell count, coagulation function indexes, blood biochemistry, urine, cytokines, immunoglobulins, T-lymphocyte subpopulations, T-lymphocyte proliferation, organ weights, and organ coefficients of the animals in each dosing group of the negative control group and the supplier test product.

Gross and microscopic examinations 2 days after the final dose and at the end of the recovery

period did not show significant drug-related systemic toxicologic physiological changes (except localized by injection).

No anti-cellular antibody production was detected after multiple administrations of the test material to the animals, suggesting that dental pulp MSCs are weakly immunogenic in animals.

The results of <10^6~$2.5x10^6$ toxicokinetics and tissue distribution showed no exposure in thep eripheral blood after repeated periodontal tissue injections of dental pulp MSCs given to Parmesan miniature pigs at a dose of $0.25x10^6$~$2.5x10^6$ cells/kg. After repeated intravenous injections of dent al pulp MSCs at a dose of $2.5x10^6$ cells/kg given to Bama minipigs, dental pulp MSC genomic DN A was detected in the peripheral blood of only a few animals immediately after the end of the first a dministration, at a concentration ranging from 484 to 3886 copies/μg minipig genomic DNA.

After repeated periodontal tissue injections and intravenous drip administration of dental pulp MSCs to Bama minipigs at doses ranging from $0.25x10^6$ to $2.5x106^6$cells/kg, the amount of dental pulp MSC genomic DNA in all tissues of the animals in all dose groups was below the lower limit of quantification (the lower limit was 100copies/500ng of minipig genomic DNA), and no distribution of tissues was detected.

Repeated periodontal tissue injections of dental pulp MSCs given to Bama minipigs at a concentration of $2x10^7$ cells/mL ($0.25x10^6$, $2.5x10^6$ cells/kg/kg dose groups) resulted in foci of periodontal membrane abscesses or inflammatory cell infiltration localized in the injection area in association with the administration of the drug, with a trend toward significant recovery seen after discontinuation of the drug.

In summary, under the conditions of this experiment, dental pulp MSCs were given to Bama minipigs by repeated periodontal tissue injections at doses of $0.25x10^6$ and $2.5x10^6$ cells/kg, and repeated intravenous drip injections at a dose of $2.5x10^6$ cells/kg, once every 2 weeks for 4 consecutive weeks for a total of 3 times, and there were no abnormal changes in the various detection indexes related to the administration of the drug, and the safe dose (NOAEL) was 2. $5x10^6$ cells/kg, which is about 15 times the proposed clinical dose. Repeated intravenous and periodontal tissue injections of dental pulp MSCs given to Bama mini pigs did not detect anti-cell antibodies, which were weakly immunogenic. Repeated periodontal tissue injections of dental pulp MSCs at a concentration of $2x10^7$ cells/mL were given to PNG minipigs, and foci of periodontal membrane abscess or inflammatory cell infiltration were seen locally in association with the administration of the drug, with a tendency for significant recovery seen after discontinuation of the drug. After repeated periodontal tissue injections or intravenous drip administration of MSCs at doses of $0.25x10^6$ and $2.5x10^6$ cells/kg, genomic DNA of MSCs could be detected in only a few animals in the intravenous drip group immediately after the first administration of the drug, and no tissue distribution was detected in the tissues of animals in all groups of the test article after repeated periodontal tissue injections and intravenous drip administration. Tissue distribution was not detected in any of the groups of animals.

1.2.3.3 Systemic active allergic reaction experiments in guinea pigs given dental pulp MSCs

To provide animal experimental information for clinical studies by observing the occurrence of rapid systemic allergic reactions in guinea pigs after repeated acceptance of the test material.

Twenty-four guinea pigs, males, were randomly divided into four groups, six in each group. They were negative control group (saline), positive control group (human albumin, sensitizing dose 50mg/kg, stimulating dose 100mg/kg), low dose group of the test article (sensitizing dose $2x10^5$/kg, stimulating dose $4x10^5$/kg) and high dose group of the test article (sensitizing dose $1x10^6$/kg,

stimulating dose 2x10^6/kg), respectively. Sensitization was performed by intraperitoneal injection once every other day for a total of three times; the first three animals in each group were stimulated by intravenous foot injection 14 days after the last sensitization dose, and the remaining animals were stimulated 21 days after the last sensitization dose. After sensitization, the animals in each group were observed for signs of allergic reactions.

On the day of each sensitization and excitation, the concentration analysis of the low and high dose groups of the test material was carried out, and the cell viability was above 70%, and the formulated concentration was in the range of 90% to 115% of the theoretical concentration, which was in accordance with the requirement of the accuracy of the formulation (80% to 120%).

During the sensitization period, no abnormal reactions were observed clinically in all groups of animals.

After 14 days of intravenous stimulation after the final sensitization, 3 animals (3/3) in the negative control group did not show any symptoms of allergic reaction, and their allergic reactions were negative; 3 animals (3/3) in the positive control group showed different degrees of symptoms of allergic reaction such as uneasiness, erect hair, nose scratching, sneezing, coughing, urination, respiratory difficulty, unstable gait, spasm, tidal respiration and death, etc., and the allergic reactions were extremely strong positive; the test article Three animals (3/3) in the low-dose group and three animals (3/3) in the high-dose group of the test article did not show signs of allergic reaction, and the allergic reaction was negative.

Twenty-one days after the final sensitization, three animals (3/3) in the negative control group showed no signs of anaphylaxis and were negative for anaphylaxis. Three animals (3/3) in the positive control group showed signs of anaphylaxis of varying degrees, such as restlessness, erect hair, scratching of the nose, coughing, urination, respiratory distress, rales, unstable gait, spasms, tidal respiration, and death, and the anaphylactic reactions were strongly positive to very strong positive; Three animals (3/3) in the low-dose group of the test article and three animals (3/3) in the high-dose group of the test article did not show signs of anaphylactic reaction, and the anaphylactic reaction was negative.

Under the conditions of this experiment, dental pulp MSCs were sensitized by intraperitoneal injection at a dose of 2x10^5 cells/kg1x10^6 cells/kg, and stimulated by intravenous injection at a dose of 4x10^5 cells/kg and 2x10^6 cells/kg, and the systemic active anaphylactic reaction given to guinea pigs was negative.

1.2.3.4 In vitro hemolysis of New Zealand rabbit erythrocytes by dental pulp MSCs

The purpose of this experiment is to observe whether dental pulp MSCs cause hemolysis or coagulation of rabbit erythrocytes in vitro, so as to provide in vitro experimental information for clinical research.

In this experiment, the effect of dental pulp MSCs on hemolysis and coagulation of rabbit erythrocytes was observed using the in vitro test tube method. Dental pulp MSCs at a concentration of 1.7x10^7 cells/mL were added in different volumes (0.5-0.1mL) to glass test tubes that already contained different volumes (2.0-2.5mL) of sodium chloride injection and 2.5mL of 2% erythrocyte suspension, and at the same time, sodium chloride injection and sterilized water for injection were used as the negative and positive controls, respectively. The total volume of each test tube was 5.0 mL, and it was placed in a 37 thermostat for 3 hours to observe the hemolysis and coagulation of erythrocytes.

Concentration analysis was performed on the prepared test material, the cell viability was

94.86%, and the prepared concentration was 1.8x10^7 cells/mL, which was 105.88% of the theoretical concentration, and met the requirement of preparation accuracy (80%-120%).

In the test tube containing dental pulp MSCs (concentration of 1.7x10^7 cells/mL) and the negative control tube of sodium chloride injection, when incubated for 3h, the upper layer of the liquid in the test tube could be seen as colorless and clear at each observation point, and the erythrocytes at the bottom of the tube could be seen to be sinking; the positive control tube of sterilized water for injection could be seen to be clear and red, with no delamination and no residual red cells at the bottom of the tube, when the test tube was incubated for 15min to the end of the 3h observation. No erythrocytes remained at the bottom of the tubes.

Under the conditions of this experiment, the concentration of 1.7x10^7 cells/mL of dental pulp MSCs had no hemolytic effect on rabbit erythrocytes in vitro, and did not cause coagulation of erythrocytes.

1.2.3.5 Validation of the method for quantitative PCR to detect the distribution of dental pulp mesenchymal stem cell genome in minipigs

In this experiment, plasmid pMD19-homo4 containing human species-specific sequence fragments was used as a standard, and real-time fluorescence quantitative PCR was utilized to quantitatively detect the content of human dental pulp MSCs in the genomic DNA of minipigs.

This experiment was mainly accomplished to validate the quantitative PCR method for determining the genomic distribution of dental pulp MSCs in minipigs. The specificity, linearity, precision, accuracy and lower limit of quantification of the method were examined, as well as the stability of the samples under different placement conditions.

The method was validated to be able to specifically detect the genomic distribution of dental pulp MSCs in minipigs. The linear coefficients (R2) of the standard curves ranged from 0.991 to 0. 996 in the range of 100 to 10 copies/reaction, the amplification efficiencies ranged from 0.84 to 0.90, and the lowest limit of quantification was 100copies/reaction. The accuracy of the quality control samples at each concentration (100, 104, 107copies/reaction) ranged from 59.30 to 170.79%, and the precision ranged from 13.29 to 19.44%.

The recoveries of the low, medium and high concentration stability samples under each storage condition (4 h at 2-8 , 7 and 14 days at -70 , and three cycles of freeze-thaw at -70 and room temperature) ranged from 99.00 to 162.19%, which meets the stability requirements.

In conclusion, this method is sensitive and has good precision and accuracy, and can be used to quantitatively determine the genomic distribution of dental pulp MSCs in small pigs. The genomic DNA samples can remain stable under three cycles of 2-8 for 4h, below -70 for 7 days and 14 days, and below -70 - room temperature freeze-thaw.

1.2.3.6 Experiment on the effect of dental pulp MSCs on the tumor growth of human cervical cancer Hela cell NOD SCID loaded mice

PURPOSE: To establish a subcutaneous transplantation tumor model of human cervical cancer Hela cell NOD SCID mice, and to investigate the effect of dental pulp mesenchymal stem cells on the tumor growth of NOD SCID mice.

METHODS: Thirty-two model animals were randomly divided into four groups: group 1 (cell dilution, i.v.), group 2 (pulp MSCs, 4x10^7/kg, i.v.), group 3 (pulp MSCs, 8x10^7/kg, i.v.), and group 4 (pulp MSCs, 8x10^7/kg, subcutaneous), with eight female NOD SCID mice. The dosage was 0.2 ml/ 10g, the administration rate was 3-5 min/ml, and the first, third, and fifth doses were given once each week. And the animals were observed continuously for 8 weeks after the first dose.

During the period of drug administration, the general clinical performance of the animals was observed twice a day, and the body weight and tumor diameter were measured twice a week. After euthanasia, the tumors were stripped and weighed. Tumor volume, relative tumor volume RTV, relative tumor proliferation rate T/C%, and tumor weight inhibition rate IRTW% were calculated. Tumor growth inhibition was defined as relative tumor proliferation rate T/C% ⩽ 40% and relative tumor volume RTV P < 0.05 compared with negative control group. If T/C% ⩾ 140%, there was tumor growth promotion; if 40% < T/C% < 140%, there was neither promotion nor inhibition of tumor growth. The tumor weight inhibition rate IRTW% was also calculated as a reference.

RESULTS: Throughout the experiment, symptoms such as tumor rupture or softening, and roughness of coat were observed in animals in groups 1 to 4 from day D5. The average body weight of animals in each group continued to grow steadily, except for a slight decrease on day D5. The average body weights of groups 1 to 4 at the time of euthanasia at D57 were 24.0±1.0g, 23.6±1.0g, 23.1±1.6g, and 23.6±0.7g, respectively, and there was no significant difference between the groups (P>0.0.05).

CONCLUSION: Under the conditions of this experiment, the following conclusions can be drawn: donor dental pulp MSCs given to human cervical pain Hela cell NOD SCID mouse subcutaneous graft tumors at doses of $4x10^7$/kg and $8x10^7$kg intravenously and $8x10^7$/kg subcutaneously did not show any promotional or inhibitory effects on the growth of the subcutaneous transplant tumors in the mice.

1.2.3.6 Experiment on the effect of dental pulp MSCs on the tumor growth of human lymphoma Raji cell NOD SCID loaded mice

PURPOSE: To establish a subcutaneous transplantation tumor model of NOD SCID mice with human lymphoma Raji cells, and to investigate the effect of donor dental pulp MSCs on the tumor growth of NOD SCID mice.

METHODS: 32 model-forming animals were randomly divided into 4 groups: group 1 (cell dilution, intravenous injection), group 2 (dental pulp MSCs, 4x107/kg, intravenous injection), group 3 (dental pulp MSCs, 8x107/kg, intravenous injection), and group 4 (dental pulp MSCs, 8x107/kg, subcutaneous injection), with 8 female NOD SCID mice. The dosage administered was 0.2 ml/10g, and the rate of administration was 3-5 min/ml. 1 dose was given in each of the 1st and 3rd weeks, and the animals were observed continuously for 24 days after the first dose. During the period of drug administration, the animals were observed twice a day for general clinical manifestations, weight and tumor diameter were measured twice a week, and blood was taken before euthanasia for hematological indexes. After euthanasia, the tumors were stripped and weighed. Tumor volume, relative tumor volume RTV, relative tumor proliferation rate T/C%, and tumor weight inhibition rate IRTW% were calculated. Tumor growth inhibition was defined as relative tumor proliferation rate T/C% ⩽ 40% and relative tumor volume RTV with P < 0.05 compared with negative control group T/C % ⩽ 40%. If T/C% ⩾ 140%, there was tumor growth promotion; if 40% < T/C% < 140%, there was neither promotion nor inhibition of tumor growth. The tumor weight inhibition rate IRTW% was also calculated as a reference.

RESULTS: Throughout the experiment, animals in groups 1-4 were observed to have tumor rupture, hind limb weakness, mental instability, emaciation, and even death individually from day D15, which might be related to the higher degree of tumor malignancy, invasion and metastasis

during tumor growth. According to the principle of humane endpoint to end the experiment early, at the time of euthanasia on D25, the average body weight of groups 1-4 was 23.1±2.5g, 20.3±2.4g, 22.0±1.3g and 21.7±21.7±2.5g, respectively, and there was no significant difference between the groups (P>0.05).

CONCLUSION: Under the conditions of this experiment, it can be concluded that the subcutaneous graft tumors of NOD SCID mice given human lymphoma Raji cells by intravenous injection of donor dental pulp MSCs at the doses of 4x107/kg and 8x18x107 and subcutaneous injection of 8x107/kg did not show any promotional or inhibitory effect on the growth of subcutaneous transplant tumors of NOD SCID mice.

1.2.3.7 Tissue distribution and stereotyped differentiation of dental pulp MSCs in NOD/SCID mice

This experiment mainly investigated the tissue distribution characteristics and colonization and differentiation of dental pulp MSCs given to NOD/SCID mice by a single periodontal tissue injection to provide data support for subsequent experiments.

A total of 48 female NOD/SCID mice were used in the experiment, and the dosage was $2x10^6$ cells/animal, the method of administration was periodontal tissue injection, the frequency of administration was a single injection, and the volume of administration was 0.04 mL/animal.

The animals were euthanized at 4h, 24h, 48h, 3d, 5d, 7d, 14d and 21d after the administration of the drug and then tissue samples were collected. Six animals were euthanized at each time point, and the brain, muscle, heart, liver, spleen, lungs, kidneys, spinal cord, and administered localities of the first three animals (in order of animal number size) were taken for the colonization and differentiation study.0 The brain, muscle, heart, liver, spleen, lungs, kidneys, spinal cord, administered localities, and whole blood of the last three animals were taken for the tissue distribution study. If there was a positive result in any of the 3 animals used for tissue distribution assay at each time point, the same tissues from the other 3 animals were taken for colonization and differentiation assay.

A validated Q-PCR method with a lower limit of quantification of 100 copies/500 ng of mouse genomic DNA was used to determine the content of human dental pulp MSCs in tissue samples from NOD/SCID mice, and tissues with positive PCR results were tested for colonization and differentiation of dental pulp MSCs in NOD/SCID mice by immunohistochemistry using murine anti-human cell nuclear antibody. SCID mice.

The experimental results showed that:

(1) After the dental pulp MSCs were injected into the periodontal tissues of NOD/SCID mice at a dose of 2x10°cells/animal, the presence of the dental pulp MSC genome was detected in the injected local tissues from 4h after the administration of the drug to 7d after the administration of the drug at a content of 514-50732 copies/μg of mouse genomic DNA, while no distribution was observed in other tissues, and the pulp MSC genome was not detected in other tissues, and the dental pulp MSC genome was not found in other tissues, and the dental pulp MSC genome was not found in other tissues. The content of MSC genomes in the injected localities showed a decreasing trend with the extension of the time after the administration of the drug, and the results were below the lower limit of quantification of the method at 14d and 21d after the drug was administered.

(2) In this experiment, no colonization and differentiation of dental pulp MSCs were seen in the injected local tissues of NOD/SCID mice.

1.2.4 Relevant data from previous human studies

The safety analysis of the clinical study data of all 18 subjects with chronic periodontitis from two non-registered clinical studies conducted in the previous period, and the preliminary validity analysis of the clinical evaluation index data of 199 teeth totaling 1,194 test sites of 14 subjects (7 male and 7 female, aged 27-60 years old) who had complete follow-up data, showed the following results:

(1) No serious adverse events or serious adverse reactions occurred in 18 subjects during the study period, and no subjects withdrew from the study because of adverse events or adverse reactions. 13 subjects received periodontal basic therapy combined with a single local injection of DPSCs injection, and cell administration was safe and well tolerated within the dose range of 1×10^7~8×10^7 cells/case, with no dose-limiting toxicity or adverse reactions.

(2) None of the 18 subjects were followed up for tumor development, among which: 8 subjects in the open study (Project 1) did not have tumor development at 48 months after administration of the drug; 10 subjects in the randomized controlled study (Project 2) did not have tumor development at 18/24 months after administration of the drug, and there were no clinically significant positive results of the blood tumor marker test, and no human pulp mesenchymal stem cell injections were observed in the follow-up of both studies. No tumorigenic risk was observed for DPSCs injection in both studies.

(3) The clinical evaluation data of the 14 subjects with complete follow-up data showed that the clinical efficacy of periodontal basic treatment + cellular drug delivery treatment was better than that of periodontal basic treatment + untreated/saline control treatment, ① the periodontal attachment level (AL) of the subjects improved, and the periodontal attachment level (AL) of the subjects improved significantly 3 months after cellular drug delivery treatment compared with that of the control teeth; ② the periodontal probing depth (PD) of the subjects improved; and ③ the clinical efficacy of the periodontal basic treatment + cellular drug delivery treatment was better than that of the control teeth. Probing depth (PD) improved in all subjects, and compared with control teeth, periodontal probing depth (PD) significantly improved in the cell-delivered treatment group at 3 and 6 months after treatment.

(4) As shown in the randomized controlled study of 10 cases (Project 2), the periodontal soft tissue recovery in the periodontal basic treatment combined with cellular drug delivery treatment group was better than that in the saline control group. 2/5 subjects in the saline control group had mild gingival redness and swelling, and bleeding on probing in multiple places throughout the mouth on the 90th day after the treatment, while 5/5 subjects in the cellular treatment group did not have any recurrence of gingival redness, swelling, and bleeding on probing in the 6-month post-treatment period as well as during the increase in the follow up period (18/24 months). gingival erythema and bleeding on probing.

Data from previous human studies are described in the “Investigator's Manual”.

1.2.5 Clinical indications

It is intended for the treatment of chronic periodontitis, such as periodontal bone tissue defects due to chronic periodontitis.

2. Purpose of clinical study

Main purpose

- To explore the safety and tolerability of DP-MSC for the treatment of chronic periodontitis.

Secondary objectives

- Dose exploration to inform the design of dosing regimens for subsequent clinical studies.
- To explore the preliminary effectiveness of DP-MSC in the treatment of chronic periodontitis.

3. Clinical study design

3.1 Basis of program design

3.1.1 Results of pharmacological and toxicological studies of DPSCs injection in the previous study

The pre-study used 1×10^7 cells/0.6ml DPSCs injection for the unit point of Bama minipig localized periodontal defects within the injection. After three months, the clinical examination indexes and radiological analysis results consistently showed that DPSCs injection could promote the regeneration of periodontal soft and hard tissues; at the same time, it was found that the inflammatory cell infiltration in the injected local gingival tissues was reduced, and the inflammation was alleviated. The small pig model experiment proved that DPSCs injection has good periodontal tissue regeneration and anti-inflammatory effect, and can be used for regeneration treatment of periodontal soft and hard tissue defects. The clinical proposed dose was extrapolated from the efficacy to 1×10^7 cells/periodontal defect site.

The results of acute toxicity experiments of DP-MSC administered by single intramuscular and intravenous injections in ICR mice suggested that the maximum tolerated dose of DP-MSC in mice was greater than or equal to 5×10^7 cells/kg, which was 250 times of the clinically proposed dose.

The results of long-term toxicity experiments of DP-MSC administered by repeated periodontal tissue injections and intravenous drip in Bama minipigs showed that the safe dose of DP-MSC in Bama minipigs was 2.5×10^6 cells/kg, which was more than 10 times of the effective dose in minipigs, and about 15 times of the clinically proposed therapeutic dose. And DP-MSC have no obvious target organ for toxic response.

Therefore: the DPSCs injection is safe for local injection treatment within the dose concentration range of 2.5×10^6 cells/kg.

3.1.2 Program design ideas based on the results of previous studies

Based on the relevant provisions of the 2020 Code for Quality Management of Drug Clinical Trials and the 2017 Guiding Principles for General Considerations in Drug Clinical Trials issued by the State Food and Drug Administration, with reference to the relevant contents of the 2020 Technical Guidelines for Clinical Trials of Human-derived Stem Cells and Derived Cell Therapy Products (Draft for Comment) and the domestic and international experience of research and development of stem cell drugs, and in view of the project's disease diagnosis and treatment characteristics of the indications and the biological characteristics of DP-MSC, it is not possible to carry out human metabolism studies of the cell drug in healthy populations and subjects, and it is proposed to carry out a Phase I clinical trial with the primary purpose of exploring the safety and tolerability of the cell drug, and the secondary purpose of the Phase I clinical trial is to carry out the follow-up observation of the effectiveness. After obtaining safety and tolerability data, further clinical trial programs will be scientifically and rationally designed.

This study was conducted by the Phase I Clinical Trial Research Laboratory, a nationally approved drug clinical trial organization, and investigators in the dental profession.

Consideration of subject selection, based on the relevant provisions of the 2020 Code for Quality Management of Drug Clinical Trials issued by the State Food and Drug Administration, with reference to the 2020 Technical Guidelines for Clinical Trials of Human-Derived Stem Cells and Derived Cell Therapy Products (Exposure Draft), and with full consideration of the possible effects of this cellular medication on regeneration of periodontal soft and hard tissues in healthy humans, it was determined that the study was selected to be carried out in subjects with chronic periodontitis subjects.

This study was conducted in subjects with chronic periodontitis. The design of the dosage concentration was based on the clinical proposed therapeutic dose of 1×10^7 cells/periodontal defect site as projected by the laboratory efficacy, and the area tolerance factor of the local injection operation for periodontitis treatment and the actual therapeutic demand were taken into account, so that five dosage groups were designed in the range above and below the clinical proposed therapeutic dose, including the starting concentration of low dose, the concentration of medium-low dose, the concentration of medium dose, the concentration of higher dose, and the concentration of the highest dose, and the dosage groups were evaluated at each dose concentration of this cellular drug. The human safety and tolerability of this cellular drug at each dose concentration were examined and evaluated.

To minimize or control bias, this study was designed in a double-blind fashion. Due to the slightly different appearance of the injections of the test drug and the blank control drug due to the dosage form, a non-blinded panel will be formed to ensure that other relevant researchers remain blinded during the study. The non-blinded team members include: non-blinded physicians, non-blinded nurses, non-blinded CRCs, non-blinded drug administrators, and non-blinded supervisors, who will be responsible for tasks related to receiving, distributing, administering, recalling, recording, and data monitoring of the trial drug. The unblinded team members will not perform any assessments during the study. All study physicians were required to undergo a concordance evaluation before participating in the study, and members with a concordance score of ≥85% were allowed to proceed to the next step of the study.

To better maintain blinding, non-blinded physicians should use simulated blinding (e.g., use of a blindfold) for subjects.

The safety and tolerability assessment indexes were designed in accordance with the needs of clinical assessment, and based on the time of acute and delayed immune response due to cellular drugs, the inpatient observation period after administration was set at 24 h. To ensure the protection of the rights and interests of the subjects and maximize the benefits, and taking into account the secondary objectives of the study, the follow-up observation time for safety and efficacy was set at 6 months after the administration of the drug, and at the same time, at the 12th month after the administration of the drug (D360±14D) and 24 months (D720±30D).

3.2 Study design and sample size

The study was dose-escalating, randomized, double-blind, (blank) controlled, and 36 eligible subjects were selected for enrollment in the study. There were 5 dose groups, 4 subjects in the first dose group, and 8 cases in each of the remaining 4 dose groups, totaling 36 cases; each dose group was randomly assigned to either the experimental group or the blank control group according to 3:1. Subjects were discharged from the hospital for 24 h of observation after administration of a single local injection, and the study was concluded at 6 months (D180±14D) after administration, with clinical safety and efficacy indices collected from the subjects. Long-term follow-up was conducted at the 12th month after administration (D360±14D) and 24th month after administration (D720±30D) to collect clinical efficacy observations.

3.2.1 Dosage escalation

(1) Starting dose design

Data from the preclinical toxicology study of this product showed that DP-MSC were given at a dose of 0.25×10^6~2.5×10^6 cells/kg by repeated periodontal tissue injection and 2.5×10^6 cells/kg by repeated intravenous drip, and no abnormal changes were observed in all the test indexes related to the administration of the drug. It is suggested that the safe dose (NOAEL) of DP-MSC for tissue injection or intravenous drip in PNG pigs is 2.5×10^6 cells, which is about 15 times of the proposed clinical dose. Meanwhile, in view of the limited proliferation or differentiation potential and relatively low immunogenicity of MSC products, a comprehensive analysis was conducted to set a safety factor of 10, and a single administration of 1.50×10^6 cells/person/dose was calculated at 60 kg (body weight of each person was set at 60 kg).

According to the design of clinical studies of similar drugs, the dosage used in the reference clinical research literature: ① DP-MSC in patients with bone defects caused by periodontal disease was administered at a single dose of 5.0×10^6 cells/person/dose; "Retrieval of a periodontally compromised tooth by allogeneic grafting of mesenchymal stem cells from dental pulp: A case report" [1]; ② DP-MSC was administered locally at a dose of 1.0×10^6 cells/person/dose in patients with chronic periodontal disease; "Study of Local Periodontal Regeneration of Chronic Periodontal Disease Patients Receiving Allogeneic Human Dental Pulp Stem Cells Injection Therapy" [2].

According to the available clinical data of this product, all 13 subjects received periodontal basic treatment combined with a single local injection of DP-MSCs, administered at a dose ranging from 1×10^7 to 8×10^7 cells, and were observed for 18/24/48 months of safe follow-up, and the data suggested that DP-MSC were safely and well tolerated.

In summary, the starting dose of 1×10^6 cells/person/dose was formulated for this study.

(2) Dose-escalation program

There were 5 dose groups in the study, 4 subjects in the first dose group, and 8 subjects in each of the remaining 4 dose groups, administered by single local injection. The range of dose increment was tentatively set as:

First dose group: 1×10^6 cells/periodontal defect site.

Second dose group: 5×10^6 cells/periodontal defect site.

Third dose group: 1×10^7 cells/periodontal defect site.

Fourth dose group: 1×10^7 cells/periodontal defect site, totaling 2 periodontal defect sites, with a combined cell injection volume of 2×10^7 cells/2 periodontal defect sites.

Fifth dose group: 1×10^7 cells/periodontal defect site, a total of 3 to 4 periodontal defect sites, with a combined cell injection volume of 3×10^7 to 4×10^7 cells/3 to 4 periodontal defect sites.

Subjects were sequentially enrolled from the low-dose group to the high-dose group according to the principle of dose escalation, and each subject received only one corresponding dose. Four subjects were enrolled in the first dose group and randomly assigned to the test group (3 cases) or the blank control group (1 case); 8 subjects were enrolled in each of the remaining 4 dose groups and randomly assigned to the test group (6 cases) or the blank control group (2 cases) to carry out the safety and tolerability study of single administration.

During the course of the study, at the investigator's discretion, subjects will proceed to the next dose group if they do not experience an adverse event related to the test drug that meets the criteria for termination of the dose-escalation within 2 weeks of receiving the test drug injection. The investigator will determine the subsequent trial schedule based on the safety profile of each subject within each dose group.

(3) Termination Criteria for Dose Escalation

The degree of adverse events observed in the study will be determined by the NCI CTCAE v5.0 grading criteria. Dose escalation will be terminated and the previous dose will be defined as the MTD if any of the following occurs within 2 weeks of receiving the test drug injection:

① Half or more of the subjects in either dose group experience a Grade 3 or higher drug-related adverse event;

② If the maximum dose ($3 \times 10^7 \sim 4 \times 10^7$ cells/3 to 4 periodontal defect sites) is reached without the occurrence of Grade 3 or higher adverse events potentially related to the test drug in half or more of the subjects, the investigator, in consultation with the CMO, decides whether it is necessary to continue the dose escalation; if the dose escalation is not continued then this dose group will be defined as MTD.

(4) Treatment of Shedding Subjects

If a subject is dislodged within 4 weeks of receiving the test drug injection, the subject will be supplemented in the corresponding dose group; if dislodged after 4 weeks then no supplementation will be made.

3.2.2 Randomized, double-blind (blank) control

[Randomized, double-blind design]

Randomization

In this study, complete randomization was used to generate the random table and the groups corresponding to the random table with SAS (version 9.4 or above) software, and random numbers were assigned using the electronic central randomization system (IWRS) for clinical trials. Each dose group was randomized separately. Each eligible subject was assigned a randomization number on D-1 according to screening number from smallest to largest and entered into the corresponding group.

Double-blind

To minimize or control for bias, the study was designed to be double-blind. Due to the slightly different injection appearance of the test drug and the blank control drug due to the dosage form, a non-blinded panel will be set up to ensure that other relevant researchers remain blinded for the duration of the study. The unblinded team members include: unblinded physicians, unblinded nurses, unblinded CRCs, unblinded drug administrators, and unblinded supervisors, who will be responsible for receiving, distributing, administering, retrieving, documenting, and data monitoring of the trial drug. The unblinded team members will not perform any assessments during the study. All study physicians were required to undergo concordance evaluation before participating in the study, and members with a concordance score of ≥85% were allowed to proceed to the next step of the study.

To better maintain blinding, non-blinded physicians should use simulated blinding of subjects (e.g., use of a blindfold).

Contingency letters: electronic contingency letters were used in this study, with one contingency letter (electronic) corresponding to each randomization number, recording the treatment group to which the randomization number corresponds. Emergency letters were used for emergency blinding and were authorized to the investigator. The IWRS retained the trajectory of the emergency blinding operation.

Emergency blinding: Emergency blinding through the IWRS may be performed in emergency situations when the investigator believes that knowledge of the medication used by the subject would be beneficial in the management of an adverse event.

Blinding rules: one blinding method was used in this study. Blinding was performed after completion of the statistical analysis and the drug corresponding to the group code was revealed. The blinding document was signed by the principal investigator, sponsor, and statistician.

[(blank) Control]

Test drug: a single local injection of DPSCs injection given on top of basic periodontal treatment (supragingival scaling, subgingival scraping and root and leveling);

Blank control: a single local injection of saline given on the basis of basic periodontal treatment (supragingival scaling, subgingival scraping and root leveling).

3.2.3 Sample size

Number of subjects: 36 qualified subjects with chronic periodontitis.

4. Clinical Study Population

4.1 Clinical study subjects:

Subjects with chronic periodontitis.

4.2 Inclusion Criteria.

Subjects must fulfill all of the following criteria to be enrolled in the study:

(1) Age 18-65 years old (including the critical value), gender is not limited;

(2) Radiologic detection of a vertical-type bone defect at the periodontal defect site and a probing depth (PD) of 4-8 mm at the periodontal defect site;

(3) Voluntarily participate in this clinical study, understand and sign the informed consent form, and voluntarily comply with the relevant regulations of this study during the study period and for 18 months after the end of the study.

4.3 Exclusion Criteria

Subjects who fulfill any of the following will be excluded from this study:

History of local/systemic treatment:

(1) Subjects with severe periodontal disease (alveolar bone resorption generally exceeding two-thirds of the root length) that interferes with the judgment of the study teeth;

(2) Study teeth with looseness ≥2 degrees (dental forceps clamping method: 1 degree for buccolingual movement only; 2 degrees for both buccolingual and proximal-distal-medial movement; 3 degrees if vertical looseness is present);
(3) Those with previous surgical treatment of periodontal bone defect sites and adjacent periodontal tissues;
(4) Those who have been treated with non-steroidal anti-inflammatory drugs, steroidal (steroidal) hormones within 3 months prior to screening, and/or those who have used hormones (except topical topical hormones) within 3 months prior to screening, and those who have used bisphosphonates within 3 months prior to screening;
(5) Those with severe systemic infections within 3 months prior to screening; or those using antibiotics within 72h prior to screening; history of co-morbidities/conditions:
(6) Those with uncontrolled hypertension (definition: systolic blood pressure ≥160 mmHg or diastolic blood pressure ≥95 mmHg in the seated position of the subject after receiving an optimal regimen of antihypertensive therapy) within 1 month prior to screening;
(7) Persons with systemic systemic diseases (including, but not limited to: patients with malignant tumors or those who had a positive tumor test during the screening process, diabetic patients, patients with cardiac disease resulting in heart failure, those who had a myocardial infarction within six months prior to screening, those who had symptoms of angina pectoris within six months prior to screening, and patients with congenital heart disease, etc.);

History of allergies:

(8) Persons known to be potentially allergic to any of the materials used in the treatment process;
(9) Persons with allergies, previous history of allergy to blood products;

Laboratory Tests:

(10) Laboratory tests (any one of which meets): liver function: ALT > 80 U/L or AST > 70 U/L, renal function: blood creatinine (picric acid method) > ULN; and
(11) Individuals with bleeding tendency or coagulation disorders (International Normalized Ratio (INR) ≥1.5 times ULN, or Activated Partial Thromboplastin Time (AAPTT) ≥1.5 times ULN (except for patients on anticoagulant therapy)) or severe hematologic disorders (e.g., grade 3 or higher anemia ((Hb <80 g/L); grade 2 or higher thrombocytopenia (<75.0 × 10^9/L))
(12) Positive serum virology (HBsAg, HCV antibody, HIV antibody, syphilis spirochete antibody) test;

History of childbearing:

(13) Those who have had unprotected sex within 1 month prior to screening;
(14) Pregnant or breastfeeding women, or those who tested positive for human chorionic gonadotropin β (β-HCG) prior to screening, or those who are unable and unwilling to use investigator-approved contraception as directed by the investigator for the duration of the study and for 6 months after the study;

(15) Women who apply oral contraceptives for contraception;

Others:

(16) Subjects with a history of tobacco addiction (≥10 cigarettes per day) in the 12 months prior to screening;
(17) Subjects with mental or consciousness disorders;
(18) Subjects who have participated in other clinical studies within 3 months prior to screening;
(19) Other circumstances deemed by the investigator to be inappropriate for participation.

4.4 Withdrawal (Shedding) Criteria

(1) The subject experiences an adverse event during the course of the study that, in the opinion of the investigator, precludes continuation of the study;
(2) The subject has poor compliance and is unable to complete follow-up visits on time;
(3) Use of other medications (e.g., glucocorticoids, bisphosphonates, estrogens, etc.) that, in the judgment of the investigator, interfere with tolerability or safety judgments;
(4) Use of other foods (e.g., betel nut, beef slats, etc.) that, in the judgment of the investigator, affect tolerability or safety judgment;
(5) Subjects who do not wish to continue with the clinical study and withdraw from the investigator;
(6) Female subjects becoming pregnant during the course of the study;
(7) Loss of visit.

4.5 Discontinuation/Termination Criteria

The study may be suspended or terminated early if justified for reasons including, but not limited to:
(1) Toxic reactions during the course of the study that meet the criteria for termination dose escalation;
(2) The sponsor's own reasons such as insufficient funding, patent disputes, or reasons for changes in national pharmaceutical development policies;
(3) Suspension or termination of the approved study at the request of the drug regulatory authority or ethics committee; in addition, the sponsor reserves the right to suspend or terminate the study at any time.

Once the above mentioned issues of drug safety, protocol compliance, etc. that caused the suspension or termination of the study have been resolved and agreed upon by the sponsor, the Ethics Committee and the regulatory body, the study may continue.

Upon deciding to suspend or terminate the study, any party shall immediately send a written notice to the other parties (including but not limited to the sponsor, investigator, ethics committee, and regulatory body) and provide the relevant reasons.

4.6 Disposal of dislodged and terminated study subjects:

(1) Completion of the last follow-up examination and recording of comorbid medications and adverse events, etc.
(2) Record the time and reason for study termination in detail on the case report form.
(3) Patients terminated from the study due to an adverse event must be followed until the adverse event is resolved or the investigator deems that no further follow-up is necessary.

(4) If a subject is unable to continue in the study for special reasons after randomization and before administration of the study drug, or if a subject is dislodged before and within 4 weeks after non-dosing, a medically cleared subject of the same sex will be used as a replacement. The replacement subject will be assigned a subject randomization number obtained by adding 100 to the digits of the dislodged subject's randomization number (e.g., subject A106 will replace subject A006). The replacement subject uses the shed subject's medication. SAS software was used to generate the randomization number and the corresponding group. The randomization form (blind backed) is sealed in duplicate at the sponsor and at the research center.

5. Clinical study process

The entire clinical study process is divided into the following six phases:

(1) Screening period (D-28 to D-9)
(2) Basic periodontal treatment in the contralateral half of the study teeth (D-8±2)
(3) Issuance of randomization number (D-1)
(4) Medication (D1)
(5) Treatment observation (D2)
(6) Follow-up (D7±1D, D14±1D, D30±3D, D90±7D, D180±14D, D360±14D, D720±30D)

5.1 Screening period (D-28 to D-9)

After signing the informed consent form, subjects underwent various screening tests in the outpatient clinic, and those who met all inclusion criteria and those who did not meet any of the exclusion criteria, as determined by the investigator, were allowed to proceed to the next phase of the study. The subjects underwent basic periodontal treatment (saline rinsing and gargling, full-mouth ultrasonic supragingival cleaning and polishing, and saline rinsing) by a blinded physician.

(1) Subjects signed the ICF;
(2) Review of subject inclusion and exclusion criteria (prior to treatment) and registration of subject screening numbers for enrolled subjects;
(3) Collection of demographic information (gender, date of birth, ethnicity, height, weight);
(4) Recording of medical history:
    i. Current medical history: diagnosis, symptoms;
    ii. History of allergies, family history, past history (e.g., history of smoking, diabetes, other surgeries, etc.);
(5) Vital signs: respiration, heart rate, blood pressure (systolic, diastolic), temperature;
(6) Blood count: hemoglobin, red blood cells, white blood cells, neutrophil count, lymphocyte count, and platelet count;
(7) Liver and renal function: total bilirubin, direct bilirubin, alanine aminotransferase, aspartate aminotransferase, total protein, albumin, total bile acids, urea, creatinine, uric acid, glucose, potassium, sodium and chloride;

(8) Coagulation function: including prothrombin time (PT), activated partial thromboplastin time (APTT), international normalized ratio (INR);
(9) Inflammation index test: ultrasensitive C-reactive protein;
(10) Immunologic tests: IgA, IgG, IgM, total IgE;
(11) Infectious disease testing: HBsAg, HBsAb, HBeAg, HBeAb, HBcAb, Anti-HCV, HIVcombin, Anti-TP, CMV-IgM, CMV-IgG; and
(12) Pregnancy test: blood beta-HCG, female subjects;
(13) Urine and stool routine;
(14) Dental CBCT examination;
(15) Electrocardiogram;
(16) Record of combined medications and combined treatments.

5.2 Periodontal basic treatment in the contralateral half of the study teeth (D-8±2)

Subjects returned to the hospital on D-8±2, and a blinded physician performed periodontal basic treatment (4% ativan, half-mouth ultrasonic subgingival scraping, root planing, polishing, and saline rinsing) on the half-mouth opposite the study tooth of the subject, and completed a full-mouth periodontal examination of the following periodontal clinical indicators in the 2 h prior to receiving the periodontal basic treatment on the half-mouth opposite the study tooth: periodontal attachment level AL, periodontal probing depth PD, probing bleeding index BI, gingival recession GR, and tooth looseness TM. (This visit point PD and TM examination is only used as a baseline record, and is not used as an enrollment criteria determination)

5.3 Issuance of randomization number (D-1)

The investigator checked the inclusion/exclusion criteria on subjects who completed the relevant examinations on D-1, and those who met all the inclusion criteria and those who did not meet any of the exclusion criteria were enrolled in the study, and a randomization number was issued to all enrolled subjects.

5.4 Medication (D1)

Subjects received periodontal basal therapy (4% ativan, ultrasonic subgingival scraping of the study tooth side half of the mouth, root planing and polishing, and saline rinsing) in the study tooth side half of the mouth on D1, and completed within 2 h prior to receiving the basal therapy: vital signs (respiration, heart rate, blood pressure (systolic blood pressure, diastolic blood pressure), and temperature); and laboratory investigations (routine blood, coagulation, liver and kidney functions, inflammatory index tests, immunological tests, urine routine).

After completion of the examinations, the blinded physician performed periodontal basic treatment (4% ativan, ultrasonic subgingival scraping, root planing, polishing, and saline rinsing) on the study tooth side of the subject's half of the mouth. Immediately after completion of the periodontal basic treatment, the subjects were treated with local injections by a non-blinded practitioner of the medication assigned by the randomization method.

The specific operation was as follows:

Test drug treatment: dose groups 1 to 3 were injected locally with DPSCs injection 0.6 ml at a single periodontal defect site in the subject's oral cavity, dose group 4 was injected locally with DPSCs injection 0.6 ml/periodontal defect site at two periodontal defect sites ipsilaterally in the subject's oral cavity, and dose group 5 was injected locally with DP-MSC at three to four periodontal defect sites ipsilaterally in the subject's oral cavity, and the concentrations administered in the dose groups were as follows. injection 0.6 ml/periodontal defect site, and the concentrations administered in the dose groups were:

First dose group: 1×10^6 cells/periodontal defect site.

Second dose group: 5×10^6 group cells/periodontal defect site.

Third dose group: 1×10^7 cells/periodontal defect site.

Fourth dose group: 1×10^7 cells/periodontal defect site, totaling 2 periodontal defect sites, with a combined cell injection volume of 2×10^7 cells/2 periodontal defect sites.

Fifth dose group: 1×10^7 cells/periodontal defect site, totaling 3 to 4 periodontal defect sites, with a combined cell injection volume of 3×10^7 to 4×10^7 cells/3 to 4 periodontal defect sites.

Blank control treatment: saline 0.6 ml/periodontal defect site was injected locally in the subjects' periodontal defect sites.

5.4.1 Checks within 2h before drug administration

a) Vital signs: respiration, heart rate, blood pressure (systolic, diastolic), body temperature;
b) Blood routine: hemoglobin, red blood cells, white blood cells, neutrophil count, lymphocyte count and platelet count;
c) Liver and kidney functions: total bilirubin, direct bilirubin, alanine aminotransferase, aspartate aminotransferase, total protein, albumin, total bile acids, urea, creatinine, uric acid, glucose, potassium, sodium, and chloride;
d) Coagulation function: including prothrombin time (PT), activated partial thromboplastin time (APTT), international normalized ratio (INR);
e) Inflammatory index test: ultrasensitive C-reactive protein;
f) immunological tests: IgA, lgG, IgM, total IIgE IgG,
g) Urine routine: urine specific gravity, pH, urine glucose, urine protein, urine leukocytes (qualitative), urine ketone bodies, bilirubin, urine occult blood (qualitative);
h) Documentation of adverse events. The degree of adverse events was determined by NCI CTCAE v5.0 grading criteria;
i) Record of coadministration of medications and combined treatments.

5.4.2 Periodontal basic treatment and administration of medications

(1) Periodontal initial therapy:

a) Periodontal initial treatment: A blinded practitioner will perform periodontal initial treatment (4% ativan, ultrasonic subgingival scraping, root planing, polishing, and saline rinsing on the study side of the mouth) on the study side of the tooth and half of the mouth. Immediately after the completion of the basic treatment, a non-blinded practitioner will administer the local injection to the subject according to the medication assigned by the randomization method. The non-blinded practitioner will make sure that the package of the test/control product is intact and undamaged or contaminated before the injection; and that the treatment procedure will comply with the operating procedures and methods of use as described in the instructions for use of the test/control product.
b) The investigator records the name of the initial therapy, procedure, duration, and complications of the procedure; the non-blinded practitioner records the procedure, duration, and complications of the local injection after the injection procedure;
c) The investigator recorded the number and location of periodontal defects treated.
d) Drug administration treatment: the non-blinded physician independently recorded the name, specification, model, and production lot number of the products used in the test group and blank control group.
e) Recording of combined medications by the investigator (routine medications such as anesthetics, hemostatic drugs, antibiotics, etc., used in initial periodontal treatment procedures need not be recorded).
f) Record of adverse events: the degree of adverse events was determined according to NCI CTCAE v5.0 grading standards.

(2) Administration of treatment:

By a blinded physician in accordance with different dose groups and randomly assigned numbers, after the completion of semi-oral periodontal initial treatment (4% ativan, ultrasonic subgingival scraping on the side of the study teeth, root planing and polishing, and saline rinsing) on the side of the study teeth, the non-blinded physician immediately gave the subjects the test drug or blank control agent for local injection treatment.

5.5 Treatment observation (D1 to D2)

(1) Observation in the clinic 30 minutes after drug administration

Subjects were observed in the treatment room for 30 minutes after drug administration and then transferred to the study ward for inpatient observation.The following assessment checks were completed during the treatment room observation:

a) Vital signs: respiration, heart rate, blood pressure (systolic, diastolic), and temperature, were checked 30 min ± 5 min after drug administration;

b) Record of adverse events; the degree of adverse events was determined according to NCICTCAE v5.0 grading criteria;

c) Record of combined medication and combined treatment.

(2) 24h hospitalization observation after drug administration

Subjects were hospitalized for 24h observation after drug administration, and those without abnormalities were discharged for follow-up. Hospitalization observation to complete the following assessment checks:

a) Vital signs: respiration, heart rate, blood pressure (systolic blood pressure, diastolic blood pressure), body temperature; check 2h±5min and 24h±30min after drug administration;

b) Blood routine: hemoglobin, erythrocyte, leukocyte, neutrophil count, lymphocyte count and platelet count; checked 24h±30min after drug administration;
c) Hepatic and renal function: total bilirubin, direct bilirubin, alanine aminotransferase, aspartate aminotransferase, total protein, albumin, total bile acids, urea, creatinine, uric acid, glucose, potassium, sodium and chloride; checked 24h±30min after drug administration;
d) Immunologic examination: IgA, IgG, IgM, IgM, total IgE; examined 24h±30min after administration;
e) Urine and stool routine: urine routine was performed within 2h and 24h±30min after drug administration; stool routine was performed within 24h after drug administration;
f) Electrocardiogram: examined 2h±30min and 24h±30min after drug administration;
g) Inflammation index test: ultrasensitive C-reactive protein, checked 2h±5min after drug administration;
h) Record of adverse events; the degree of adverse events was determined according to NCICTCAE v5.0 grading standards;
i) Record of coadministration and combined treatment.

5.6 Follow-up

5.6.1 Post-treatment follow-up (D7±1D, D14±1D, D30±3D, D90±7D, D180±14D)

Subjects were followed up once each at D7±1D, D14±1D, D30±3D, D90±7D, and D180±14D to collect clinical safety and efficacy indices and to conclude the study. The main follow-up assessment items were as follows:

a) Vital signs: respiration, heart rate, blood pressure (systolic, diastolic), and temperature, which were examined at D7±1D, D14±1D, D30±3D, D90±7D, and D180±14D during the follow-up period;
b) Periodontal clinical index examination: examined by a blinded physician at D90±7D, D180±14D in the follow-up period;
   ① periodontal attachment level AL, periodontal probing depth PD, probing bleeding index BI, gingival recession GR, tooth looseness TM;
   ② Record of periodontal healing (probing bleeding index BI, gingival recession GR, tooth looseness TM):
   - Grade A healing: is the initial healing with excellent healing and no adverse reactions;
   - Grade B healing: is defined as poor healing with inflammatory reaction at the healing site, such as redness, swelling, hardness, hematoma, effusion, etc., but without suppuration;
   - Grade C healing: the injection site is purulent and needs incision and drainage treatment;
c) Blood routine: hemoglobin, erythrocytes, leukocytes, neutrophil count, lymphocyte count, and platelet count, which were examined at D7±ID, D14±1D, D30±3D, D90±7D, and D180±14D during the follow-up period;
d) Liver and kidney functions: total bilirubin, direct bilirubin, alanine aminotransferase, aspartate aminotransferase, total protein, albumin, total bile acids, urea, creatinine, uric acid, glucose, potassium, sodium, and chloride at the follow-up period D7±1D, D14±1D, D30±3D, D180±14D.Periods D7±1D, D14±1D, D30±3D, D90±7D, D180±14D were examined;

e) Immunological examination: IgA, IgG, IgM and total IgE were examined at D7±lD, D14±lD, D30±3D and D90±7D during the follow-up period;

f) Pregnancy test: female subjects were examined for Urine β-HCG at D30±3D, D90±7D and D180±14D during the follow-up period;

g) Infectious disease detection: HBsAg, HBsAb, HBeAg, HBeAb, HBcAb, Anti-HCV, HIVcombin, Anti-TP, CMV-lgM and CMV-IgG were examined at D30±3D, D90±7D and D180±14D during the follow-up period;

h) Records of adverse events: recorded at D7±1D, D14±1D, D30±3D, D90±7D and D180±14D during the follow-up period, and the severity of adverse events was determined according to the NCI CTCAE v5.0 grading standard;

i) Combined medication records: recorded at D7±1D, D14±1D, D30±3D, D90±7D and D180±14D during the follow-up period;

J) Dental CBCT examination: performed at D90±7D and D180±14D during the follow-up period;

k) Electrocardiogram: performed at D7±1D, D14±1D, D30±3D, D90±7D, and D18O±14D during the follow-up period.

5.6.2 Long-term follow-up (D360±14D, D720±30D)

Two follow-ups were conducted at 12 months (D360±14D) and 24 months (D720±30D) after administration, and clinical efficacy observation indexes were collected. The details are as follows:

Clinical efficacy observation index

a) Periodontal clinical indicators: periodontal adhesion level (AL), periodontal probing depth (PD), probing bleeding index (BI), gingival retraction (GR), and tooth mobility (TM) were examined at D360±14D and D720±30D during the follow-up period by the blind clinical doctor;

b) Dental CBCT examination: performed at D360±14D and D720±30D during the follow-up period;

c) Record of adverse events: the severity of adverse events was determined according to the NCI CTCAE v5.0 grading standard, at D360±14D and D720±30D during the follow-up period;

d) Combined medication records: recorded at D360±14D and D720±30D during the follow-up period.

5.7 Unplanned follow-up and treatment

During the study, for the safety of subjects, if adverse events occur to subjects, researchers can increase the number of follow-up visits or treatment of subjects according to actual needs, that is, unplanned follow-up and treatment. The investigator must accurately record the contents of each unplanned follow-up and treatment in the unplanned follow-up section of the original medical record and CRF data.

a) Chief complaint of symptoms;

b) Vital signs: respiration, heart rate, blood pressure (systolic and diastolic), body temperature;

c) Laboratory examination: blood routine, blood biochemistry, coagulation function,

inflammatory indicators, immunological examination (as required), electrocardiogram;

d) Record adverse events;

c) Record the combination of drugs and combined treatment;

f) Decide whether to conduct dental CBCT examination and select corresponding auxiliary examination items according to actual needs;

g) Make an appointment for the next follow-up.

5.8 Follow-up of adverse events

AE that do not recover at the end of the study should be followed up for final evaluation, and AE should be followed up until any of the following conditions occur:

1) Complete recovery or return to baseline level;

2) NCI-CTCAE5.0 grade ≤1;

3) The investigator confirmed that the AE has stabilized and is not expected to improve further;

4) Death or loss of follow-up;

5) Subjects receive other cell therapy.

After the end of the study, if the subject develops SAE, and the investigator determines after learning that the SAE is related or likely to be related to the use of the study drug, the investigator shall promptly inform the agent.

5.9 Evaluation Indicators

5.9.1 Security Indicators

① Vital Signs

Respiration, heart rate, blood pressure (systolic and diastolic) and body temperature were examined during the screening period, within 2h before administration, 30min±5min, 2h±5min, 24h±30min after administration, the follow-up period was D7±1D, D14±1D, D30±3D, D90±7D, D180±14D.

② Laboratory examination

● Blood routine: hemoglobin, red blood cells, white blood cells, neutrophil count, lymphocyte count and platelet count were examined during the screening period, within 2h before administration, 24h±30min after administration, and D7±1D, D14±1D, D30±3D, D90±7D, D180±14D during the follow-up period.

● Coagulation function: including prothrombin time (PT), activated partial thromboplastin time (APTT), and International standardized ratio (INR) were examined during the screening period and within 2h before administration.

● Liver and kidney function: total bilirubin, direct bilirubin, alanine aminotransferase, aspartate aminotransferase, total protein, albumin, total bile acid, urea, creatinine, uric acid, glucose, potassium, sodium, and chlorine were detected in the screening period, within 2h before administration, 24h±30min after administration, and D7±1D, D14±1D, D30±3D, D90±7D, D180± 14D during the follow-up period.

● Detection of inflammatory indicators: hypersensitive C-reactive protein was detected during the screening period, within 2h before administration and 2h±5min after

administration.

● Infectious disease detection: HBsAg, HBsAb, HBcAg, HBcAb, HBcAb, Anti-HCV, HIVcombin, Anti-TP, CMV-IgM and CMV-IgG were examined during the screening period and follow-up period D30±3D, D90±7D, D180±14D.

● Immunological examination: IgA, IgG, IgM, and total IgE were examined during the screening period, within 2h before administration, 24h±30min after administration, and D7±1D, D14±1D, D30±3D, and D90±7D during the follow-up period.

● Pregnancy test: female subjects were examined in the screening period, distribute random number period (D-1), and the follow-up period was D30±3D, D90±7D and D180±14D. (Only the screening period was blood test, and the other periods were urine test.)

● Urine routine: examined during screening period, within 2h before administration, within 2h and 24h±30min after administration.

● Stool routine: examined during the screening period and within 24h after administration.

● ECG: examined during the screening period, distribute random number period (D-1), 2h±30min after administration, 24h±30min after administration and D7±1D, D14±1D, D30±3D, D90±7D, D180±14D during the follow-up period.

③ Adverse events and serious adverse events

Adverse events and serious adverse events were recorded in time during the study, and the severity of adverse events was determined according to the NCI-CTCAE v5.0 grading standard.

5.9.2 Therapeutic indicators

5.9.2.1 Main therapeutic indicators

Oral clinical detection indicators: periodontal attachment level AL (probe depth PD+ gingival regression GR) were collected as baseline data from the contralateral dentition at the time of initial periodontal treatment (D-8±2).

5.9.2.2 Minor efficacy indicators

Radiological detection indicators: CBCT examination indicators during screening period were used as baseline data.

● Height of periodontal bone defect

● Average alveolar ridge density

Oral clinical detection indicators: depth of periodontal probe (PD) and periodontal healing status (probe bleeding index BI, gingival recession GR, tooth mobility TM) were collected as baseline data from the contralateral dentition at the time of initial periodontal treatment (D-8±2).

5.10 End point Indicators

Primary end points:

<u>Safety end point</u>

● Incidence and severity of adverse events (AE) associated with dental pulp mesenchymal

stem cell therapy within 180 days after administration;

Secondary end points:

Safety end point

- Incidence and severity of adverse events (AE) and serious adverse events (SAE) associated with dental pulp mesenchymal stem cell therapy within 360 days and 720 days after administration;

Efficacy end point

- Changes in periodontal attachment level (AL) from baseline to day 90 after administration;
- Changes in periodontal probing depth (PD) from baseline to day 90 after administration;
- Changes in periodontal healing (probing bleeding index BI, gingival receding GR, tooth mobility TM) from baseline to day 90 after administration;
- Changes in periodontal bone defect height from baseline to day 180 after administration;
- Change in mean alveolar ridge density from baseline to day 180 after administration.

5.11 Combined drug use

Throughout the trial period, investigators will be provided with information on any therapeutic interventions, surgical procedures, including accompanying diseases and medications, including, where possible, the diagnosis and date of onset of all diseases and the date of symptom resolution, as well as the name of the drug, the date of administration, and a description of the procedure.

Any drug taken before administration of the trial drug will be considered as concomitant drug, any other drug taken after administration of the trial drug will be considered as combination drug, and the name, reason and date of the drug used in the combination/concomitant drug will also be recorded. At each follow-up visit, subjects should be asked about the concomitant/concomitant medication.

When an AE or SAE occurs, it should be actively treated and the medication used should be recorded on the eCRF.

After receiving the experimental drug treatment for 3 months, the investigator can decide whether to receive other treatment according to the condition of the contralateral teeth; after 6 months of treatment with the experimental drug, the investigator can decide whether to receive other treatment based on the condition of the teeth studied.

6. Safety evaluation

6.1 Security evaluation indicators

All subjects were observed for any adverse events that occurred during the clinical study, including abnormalities in clinical symptoms and vital signs, abnormalities in laboratory tests, and recorded their clinical manifestations, severity, occurrence time, end time, treatment measures and outcomes, and judged the correlation with the study drugs.

Its safety was evaluated by vital signs, physical examination, laboratory examination, and immunogenicity test results during the screening period and after administration.

6.2 Adverse Events, serious adverse events

6.2.1 Definitions

Adverse Events (AE): all adverse medical events that occur after the clinical trial subjects receive the investigational drug, but a clear causal relationship with the investigational drug may not be inferred. Adverse events can manifest as signs and symptoms, disease, or abnormalities in laboratory tests, including the following:

(1) An aggravation of the pre-existing (prior to entry into the clinical trial) medical condition/disease (including an aggravation of symptoms, signs, laboratory abnormalities);

(2) Any newly occurring adverse event: any newly occurring adverse medical condition (including symptoms, signs, newly diagnosed diseases);

(3) Abnormal clinically significant laboratory test values or results that are not caused by concomitant disease.

Adverse reaction (ADR): any harmful or unintended reaction that may be associated with the investigational drug during a clinical trial. There is at least one reasonable possibility of a causal relationship between the investigational drug and the adverse reaction, that is, an association cannot be ruled out.

Significant adverse events: are defined as any AE and significant abnormalities in hematologic or other laboratory tests, other than SAEs, that led to the use of targeted medical measures (e.g., drug discontinuation, dose reduction, and symptomatic treatment).

6.2.2 Collection and recording of adverse events

Adverse events were monitored throughout the study and it was the responsibility of the investigator to record all AE observed during the study period. In this study, AE and SAE should be recorded from the beginning of the initial periodontal treatment (D-8±2) of the contralateral dentition to the end of follow-up. Any adverse medical events occurring during the period, regardless of severity and causal relationship with the experimental drug, should be recorded in the corresponding AE page of the medical record.

All AE must record the following in detail:

Description of AE: Use medical terms to describe adverse events, rather than the language reported by subjects. This should include symptoms, signs, laboratory tests of abnormalities, and diagnosis. If the same AE occurs more than once in the same subject and the subject has recovered between the two events, two AE should be recorded separately.

Date of AE occurrence: The date the subject first experienced an AE or symptoms related to an AE. If the AE is the result of a clinically significant abnormal laboratory test or examination, the date of occurrence is the sampling date.

AE outcome date: The date on which the outcome of an AE or symptoms related to an AE was recorded. If the AE still exists, do not record the return date.

The severity of AE.

Causal relationship between AE and investigational drug products.

Measures taken: ① Measures taken for experimental drugs: continue to use, stop using, use has ended, not applicable; ② Measures for AE: none, drug therapy, non-drug therapy.

Outcome of adverse events: disappearance, recovery with sequelae, remission, stability, aggravation, death, subject refused review or lost follow-up, unknown.

6.2.3 Severity rating criteria for adverse events

In this study, AE severity was graded according to the common adverse reaction time evaluation criteria (CTCAE v5.0), and AE intensity was graded as follows:

Grade 1: mild; asymptomatic or mild; found only on clinical examination; no treatment required.

Grade 2: moderate; require minor, local or non-invasive treatment; age-appropriate limitations in instrumental activities of daily living (such as cooking, buying clothes, using the telephone, managing finances, etc.).

Grade 3: serious or medically important, but not immediately life-threatening; resulting in hospitalization or prolonged hospitalization; to become disabled; limited activities of self-rational daily living (self-rational daily living means bathing, undressing, eating, washing, taking medication, and not being bedridden).

Grade 4: life-threatening and requires urgent treatment.

Grade 5: death related to AE.

6.2.4 Evaluation of association between adverse events and investigational drugs

(1) The analysis of the association between adverse events and investigational drugs shall comprehensively consider the following factors:

① Whether there is a reasonable chronological order between the adverse events and the duration of the investigational drug action;

② Whether the adverse events disappeared or were alleviated after the experimental drug was discontinued or reduced;

③ Whether the adverse events reappear after repeated administration;

④ Whether the clinical or pathological manifestations of the adverse events are consistent with known knowledge of the investigational product or the taxonomic pharmacology and toxicology of the drug;

⑤ Whether the adverse events can be explained by the original disease or the subject's own or environmental factors.

(2) Adverse events are associated with investigational drugs in the following circumstances:

① Certainly relevant: there is evidence of the use of the experimental drug. There is a reasonable time sequence between the occurrence of adverse events and the use of investigational drugs; There are instructions, similar drugs or documentary evidence; The reaction is alleviated or disappeared after withdrawal or reduction; Re-administration (if feasible) reoccurrence of adverse events; Other confounding factors such as the original disease have been excluded, and the experimental drug explanation is more reasonable than other explanations.

② Likely to be relevant: evidence of use of the experimental drug. No history of repeated drug use, the same as the previous, or although there is combined drug use, the possibility of adverse events caused by combined drug use can be basically ruled out.

③ May be relevant: evidence of use of the experimental drug. There is a reasonable time sequence between the occurrence of adverse events and the use of investigational drugs. There are instructions, similar drugs or documentary evidence; It is not clear whether the adverse events of re-administration occur again. Adverse events may be caused by more than one drug or by factors that cannot be excluded from the progression of the original disease.

④ May not be relevant: there is evidence of the use of the investigational drug. There is a reasonable time sequence between the occurrence of adverse events and the use of investigational drugs. It is not consistent with the known adverse reactions in the instructions, similar drugs or literature; It is not clear whether the adverse events of re-administration occur again. The occurrence of adverse events may be better explained by other causes.

⑤ Certainly irrelevant: no experimental drugs used; There is evidence of the use of the investigational drug, but there is no correlation between the use of the investigational drug and the timing of the adverse event, or there is a clear cause of the adverse event.

Table 1 Determination of the relationship between adverse events and investigational drugs

	Certainly relevant	Likely to be relevant	May be relevant	May not be relevant	Certainly irrelevant
A reasonable chronological order for the use of experimental drugs	＋	＋	＋	＋	—
Known types of drug reactions	＋	＋	＋	—	—
Relieved or disappeared after withdrawal or reduction of drugs	＋	＋	±/?	±/?	—
Reappear after a second dose	＋	?	?	?	—
Be explained by the combination of drugs, the progression of the subject's disease, and the effects of other treatments	—	—	±/?	±/?	＋

＋: positive; —: negative; ±: possible but difficult to judge; ?: unknown

6.3 Serious adverse events

6.3.1 Definition

A serious adverse event occurs when a subject receives an investigational drug product and occurs one or more of the following criteria:

● Resulting in death;

● Life-threatening (" life-threatening "in the definition is an AE that causes immediate death to the subject when it occurs, and does not include AE that cause death after further development);

● Cause permanent or severe disability/loss of function;

● Subject requires hospitalization or extended hospitalization;

● Causes congenital abnormalities/birth defects.

● Other important medical events: Medical and scientific judgment must be used to determine whether to expedite the reporting of other situations, such as important medical events that may not immediately threaten life, death or hospitalization, but are generally considered serious if medical measures are required to prevent the occurrence of one of the above. Examples of these events include, but are not limited to: allergic bronchospasm requiring intensive treatment in the emergency room or at home, hematological exoxia or convulsions that do not require hospitalization, potential drug-induced liver injury, suspicious transmission of pathogens (pathogenic or non-pathogenic) via experimental drugs, pregnancy, drug overdose, secondary tumors, etc.

The following situations are not considered as SAE reports:

(1) Hospitalizations or prolonged stays not associated with worsening of adverse events are not SAE. For example:

● No new AE or aggravation of the original disease (e.r., to check for laboratory abnormalities that persist from before the test);

● Hospitalization for management reasons (e.g., annual routine medical check-ups);

● Hospitalization specified in the trial protocol during the clinical trial period (e.g., operation according to the requirements of the trial protocol);

● Elective hospitalization unrelated to AE deterioration (e.g., elective cosmetic surgery)

● Scheduled treatment or surgical procedures should be recorded in the overall protocol and/or subject's individual baseline data;

● Admission to hospital solely for use of blood products.

(2) The subject's admission to hospital for administrative and social purposes is not reported as AE, such as the subject's hospitalization for recuperation, or for reasons of medical insurance reimbursement.

(3) Diagnostic or therapeutic invasive (such as surgery), non-invasive procedures should not be reported as AE. However, when the medical condition leading to this procedure meets the definition of an AE, it should be reported, such as acute appendicitis that developed during the reporting period of an AE should be reported as an AE, and the appendectomy performed as a result should be recorded as the treatment for that AE.

(4) Adverse events in clinical studies that result in hospitalization or prolonged hospitalization should be considered SAE. Hospitalization does not include: rehabilitation institutions; nursing home; routine emergency room treatment; same-day surgery (e.g. outpatient/same-day/ambulatory surgery).

(5) If hospitalizations due to signs and symptoms of disease progression during the trial period should not be reported as SAE, but deaths due to disease progression (including signs and symptoms of progression) should be reported as SAE.

6.3.2 Serious adverse event report

In this study, AE and SAE were recorded from the beginning of the initial periodontal treatment (D-8±2) in the contralateral mouth of the study teeth to the end of follow-up.

If a subject develops SAE during the study, regardless of whether it is related to the investigational drug, the investigator shall immediately take appropriate treatment measures for the subject, ensure the safety of the subject, and promptly report to the clinical trial manager of the research center. Upon learning of the SAE, the investigator must immediately inform the CRA and complete the SAE report form. CRA shall immediately report to PM and send the SAE report form to CRO and sponsor medical personnel. Medical personnel shall conduct medical review of the content of SAE report form, and then feedback the problems to researchers by email. After multi-party review and confirmation, researchers shall revise and finalize the SAE report form, sign and date it.

For SAE whose information is temporarily incomplete and uncertain, it should also report to the sponsor in a timely manner according to the principles of GCP, and supplement the report in the form of follow-up report after more information is obtained. The SAE narrative section should record and describe in detail the symptoms, severity, occurrence time, treatment time, measures taken, follow-up time and manner, and outcome of SAE. All SAEs should also be filled out in the CRF form at the same time, and the information provided in the SAE report form must be consistent with the data recorded in the CRF about the event.

The investigator must provide the severity criteria for SAE in the SAE report form, and must provide an assessment of the expected occurrence of SAE and the association (causality) with the investigational drugs based on the investigator's manual and his or her own medical judgment. If the researcher's assessment of causality is missing or not available, the sponsor will make the judgment and give the final evaluation, which will be confirmed by the researcher.

If the investigator cannot determine whether the adverse event is SAE, it is considered SAE until its nature has been proven. Such events shall be notified to the sponsor immediately in writing in accordance with the principles of the GCP.

Any death that occurs during the trial, including one that is not due to the progression of the disease being studied, whether or not related to the investigational drug, shall be promptly treated by the investigator and other required information, such as autopsy reports and final medical reports, shall be provided to the sponsor and the Ethics Committee.

For all SAE (including those that were still in development after the end of the study and up to the end of the last subject follow-up), investigators were required to follow up until the SAE resolved, improved, stabilized, reasonably explained, subject died, and lost follow-up to ensure that all issues were resolved. Provide detailed follow-up information (for example, after the study, whether special treatment was required, whether hospitalization was required, etc.).

The investigator shall submit a follow-up report to the sponsor until the SAE are cured, improved, stabilized, reasonably explained, the subject dies, and the follow-up is lost. If the damage is permanent, follow up until the adverse events are considered to have stabilized.

6.4 Evaluation of clinical laboratory test abnormalities and other abnormalities

considered as adverse or serious adverse events

Laboratory anomalies that are not clinically significant are not recorded as AE or SAE. Clinically significant laboratory test abnormalities (labeled as "clinically significant" or CS, such as clinical blood routine, blood biochemistry, etc.) and other abnormal assessment (such as electrocardiogram, vital signs, etc.) must be recorded as AE or SAE if determined by the investigator to meet the AE or SAE definition. Such as:

- All laboratory test results that are clinically significant or meet the SAE definition.
- Abnormal results of all laboratory tests requiring the subject to use specialized conventional treatment.

If the laboratory test abnormality is part of the syndrome, the syndrome or diagnosis (such as anemia) is recorded instead of the laboratory test result (i.e. decreased hemoglobin).

6.5 Suspected and Unexpected Serious Adverse Reactions (SUSAR)

6.5.1 SUSAR Definition

An unexpected adverse reaction is an adverse event whose nature, severity, consequence, or frequency is different from the expected risk described in current information about the investigational drug, such as the investigator's manual. All unexpected and serious adverse reactions that occurred during clinical trials that were definitely related or suspected to the investigational drug were SUSAR.

The prospective evaluation of SUSAR reports is based on the following four aspects: (1) whether adverse reactions are listed in the Investigator Manual (IB); (2) whether adverse events, although listed in IB, are consistent in their severity (e.g. listed adverse reactions are average, but lead to hospitalization); (3) Adverse reactions are listed in IB but occur more frequently than described in IB; (4) Other circumstances.

The relevant evaluation of the SUSAR report is based on the five principles followed by the China Adverse Drug Reaction Monitoring Center: (1) whether there is a reasonable time relationship between drug use and the occurrence of adverse reactions; (2) whether the reaction is consistent with the known type of adverse reaction of the drug; (3) Whether the reaction disappears or is alleviated after withdrawal or reduction of the drug; (5) whether the reaction event can be explained by the effect of the drug combination, the progression of the subject's disease, or the effects of other treatments.

6.5.2 SUSAR Report

SUSAR is required to report quickly in the form of individual safety reports in accordance with the procedures of the "Standards and Procedures for Rapid Reporting of Safety Data during Drug Clinical Trials" issued by the National Center for Drug Evaluation (CDE) on April 27, 2018.

Sponsor will be informed of the SAE medical evaluation, determine whether it is SUSAR, and the judgment of SUSAR SAE report form sent to PV personnel, PV personnel input the source SAE report form data into the case report page, and verify, question, and code the data into MedDRA.

For fatal or life-threatening SUSAR, PV personnel should report to the CDE, the National Health Commission, all clinical trial investigators and clinical trial institutions, and the Ethics Committee within 7 days after first being informed, and complete follow-up information within the following 8 days (day 0 on the day the sponsor was first informed).

For non-fatal or life-threatening SUSAR, PV personnel should report it to the CDE, the National Health Commission, all clinical trial investigators and clinical trial institutions, and the Ethics Committee within 15 days after first being notified. After the initial report, SUSAR should continue to follow up and submit new information or changes to the previous report in a timely manner in the form of a follow-up report, and the reporting time limit is 15 days after the new information is obtained.

SAE that occurs between the end of clinical trials or follow-up and the conclusion of review approval are reported by the investigator to the sponsor, which is SUSAR, should also be reported expeditiously.

6.6 Pregnancy

If a female subject or the partner of male subject using the investigational drug becomes pregnant during the trial, the pregnancy will be recorded and reported to the Ethics Committee and Sponsor. Any female subject who becomes pregnant (intrauterine) while participating in the study must withdraw from the trial and immediately discontinue use of the investigational drug, and any pregnancy events that occur will be reported using the clinical Trial pregnancy chart.

A female subject or the partner of male subject who has had a pregnancy event must be followed up until the birth of the baby to determine the outcome of the pregnancy (including early termination of pregnancy) and the maternal and infant status. Pregnancy complications and elective termination of pregnancy for medical reasons must be reported as AE or SAE, and spontaneous abortion must be reported as SAE.

7. Management of experimental drugs

7.1 Basic Principles of drug management

The drugs provided in the clinical trial can only be used for qualified subjects enrolled in the clinical trial, and the drugs provided in the clinical trial shall be kept and used in strict accordance with the drug instructions; All experimental drugs will be issued and recalled in strict accordance with the inclusion serial number in the Experimental Drug Distribution and Recovery Record Form; The release and recall of all drugs are strictly controlled during the inspection process.

7.2 Basic drug information

Drug name: DPSCs injection.

Main ingredients: The active ingredient is human DPSCs, and the excipient is sodium chloride injection.

Dosage forms and specifications:

- Dosage form: injection

● Size: 1×10^7 cells /0.6ml/ branch

7.3 Drug Storage

This clinical trial drug is received by a dedicated non-blind drug administrator, distributed to the trial non-blind doctors for use and retrieve. PD-MSCs should be sealed, transported and stored at 2-8°C, and used up within 10h. PD-MSCs are active biological agents and are considered ineffective if no injection is given after 10h of resuscitation.

7.4 Drug Transportation

Sealed, 2 ~ 8°C cold chain transport to the clinical trial facility.

7.5 Drug use, recovery and recording

The research center shall maintain accurate records of the use and retrieve of PD-MSCs until the number of used and discarded PD-MSCs is available at the end of the trial.

8. Data management

In this experiment, Clinflash electronic data acquisition system (EDC) was used for data acquisition. Data Management Plan: Written by the data Manager (DM) as a guidance document for the entire data management process, all data management processes should be operated according to the time, content, and method defined therein.

8.1 Electronic Case Report Form (eCRF) design

The design of eCRF must ensure the collection of all data specified in the test plan and meet the requirements of statistical analysis. The DM designs the data collection form, data form name and data items collected according to the requirements of the plan. After the eCRF finalization, the DM wrote the electronic case report Form entry Guide (eCCl) according to the eCRF, which was reviewed by the sponsor for the research center to fill in and use.

8.2 Database establishment and testing

The eCRF is designed to comply with FDA 21 CFR Part 11 and comply with the requirements of the Clinical Trial Quality Management Practice for Drugs (ICH GCP) and the Clinical Trial Quality Management Practice for Drugs (NMPA,2003) for data collection. The database programmer will build the database interface according to CRF, and perform logical verification programming according to DVS. After the database interface construction and logic check programming are completed, user acceptance test is needed, that is, data interface test and logic check test are completed respectively in the test environment. After the user acceptance test is completed, the database programmer will export the blank eCRF and submit it to the sponsor for approval, and the eCRF will be pushed to the formal environment after receiving the sponsor's confirmation.

8.3 Data Verification

Data verification includes computerized system verification and manual verification.

When the data entered conforms to the logical verification procedure, the system will automatically generate a data challenge. When the data is corrected to meet the logical verification conditions, the system will automatically shut down the doubts generated. However, if it is confirmed that the data does not need to be corrected, it is necessary to provide a reply

to the DM for confirmation. If the response is justified, the data challenge will be closed. If the data issue is not resolved, the data challenge will continue to be sent until the final resolution. For data that cannot be verified electronically, manual verification will be performed according to manual verification instructions defined in the DVS. The DM will conduct manual checks after the FSFV on a quarterly basis. As part of the data manager's dynamic data review, manual verification will ensure data quality and logical consistency. According to the data problems found by manual verification, the data question will be sent to the research center for confirmation. These queries are closed manually by the DM after the data has been corrected or confirmed.

8.4 Data entry, verification and question answering

Researchers need to collect subject data in accordance with the GCP and study protocol requirements, and fill in the eCRF accurately, timely, complete and standardized according to the filling guidelines, and the eCRF is not used as the original record.

The inspectors log in EDC at the research site of each center, check the consistency of eCRF data and source data, and can issue questions online at any time if they find problems.

Researchers can answer questions online in real time, or the CRC can update the questions or input the answers into the EDC. The data manager and the monitor give the researcher permission to answer questions, and can issue questions again, if necessary, until the data is "cleaned."

8.5 Medical Coding

This trial history and adverse events were coded using the MedDRA dictionary (version: V22.1 or later) and WHODrug (version of March 2020 or later) for combination drugs.

8.6 Locking Data

After all subjects have completed the experiment, all medical records have been entered into the system and all data questions have been answered, and the principal investigator, sponsor, statistical analyst and data manager have conducted data review and confirmed that the established database is correct, the data manager will lock the data. After all the data is locked, the data manager submits the final database export dataset to the statisticians for statistical analysis. After the data is locked, if there is conclusive evidence that it is necessary to unlock, the data can be updated after the researcher and the sponsor sign the database unlock confirmation form, and all updates must be documented. After the update is complete, the locking process need to be performed again.

8.7 eCRF Archiving and data management report

At the end of the study, cCRF electronic documents of each subject were generated and stored on CD. After the study, the data manager writes a data management report based on the actual implementation of the project.

9. Ethics and informed consent

9.1 Code of Ethics

This clinical trial strictly follows the Declaration of Helsinki (2013 edition), the NMPA's Good

Practice for the Management of Clinical Trials (GCP), and medical regulations. The approval of the lead single Ethics Committee must be obtained before the trial can commence. During the clinical trial period, any modification of this trial protocol shall be reported to the Ethics Committee and approved for filing.

9.2 Informed consent

The subject shall give informed consent to participate in the trial. It is the responsibility of the investigator to inform the subject or his designated representative about the purpose of the trial, the role of drugs, possible toxicity and possible risks and benefits. Subjects will only be enrolled after signing the informed consent form.

10. Statistical Analysis

10.1 Analyzed population

FullAnalysis Set (FAS): All cases who signed informed consent, enrollment and surgical use of this product.

Per-Protocol Population Set (PPS): All cases in FAS that met the test protocol, had good compliance without major protocol violation, took no banned drugs during the trial, and completed the contents specified in CRF, constitute the compliance protocol data set.

Safety Set (SS): All cases enrolled and all cases using the trial product, with post-treatment safety evaluation data, constitute the safety analysis data set for this trial.

10.2 Analytical method

a) The statistical analysis software will be the use of SAS Statistical analysis Software version 9.4 or above. The description of the quantitative indicators will be means, standard deviation, median, minimum, maximum, and interquartile spacing were calculated. The description of the qualitative indicators will calculate the number of cases and the percentage of each classification.

b) Validity analysis using the FullAnalysis Set(FAS) and the Per-Protocol Population Set (PPS).

c)The Safety evaluation was analyzed for the Safety Set (SS).

Adverse events were coded using the internationally accepted MedDRA Terminology Set classification, summarising the type of adverse event, frequency of occurrence, severity, and relationship to the test product and procedure by group, with special notations for subjects who were terminated from the trial due to an adverse event and those who experienced a serious adverse event. A detailed list of adverse events is provided and the frequency of each adverse event is summarised.

Describe changes in normal/abnormal laboratory and other test results before and after treatment. List the post-treatment measurements of laboratory and other tests that are abnormal and clinically significant.

Specific statistical analysis methods and related details will be detailed in the Statistical Analysis Plan (SAP), and the SAP will be finalized before the clinical trial database is locked.

10.3 An interim analysis

This study proposes to conduct an interim analysis of the efficacy and safety data of the enrolled subjects after 36 subjects have completed dosing and D180 follow-up, and to complete an interim analysis report.

11. Clinical trial risk management

11.1 Analysis of likelihood of success and failure

① This trial adheres to the Helsinki Statement (18th Joint Assembly of the World M dical Association, Helsinki, Finland, June 1964) and its latest revision (64th Joint Ass embly of the World Medical Association, Fortaleza, Brazil, October 2013), the Measures for the Administration of Drug Registration (2020), and the Code for Quality Manageme nt of Pharmaceutical Clinical Trials (2020), and other relevant regulations are implement ed, and the Measures for the Administration of Stem Cell Clinical Research (Trial, 2015) , and the trial protocols are subject to approval by the Ethics Committee. Subjects can par ticipate in the trial only after signing the ICF, which ensures the compliance of the subjec ts. According to the experience of previous clinical trials of similar products, the sheddin g rate can be controlled within 20%.

② The indications of the test products are clear, and the composition structure and technical indicators are in line with the national industry standards.

③ In the course of the trial, objective indicators were used to avoid selection bias and information bias.

④ Before the start of the trial, the clinical supervisor designated by the sponsor shall conduct unified training on the trial protocol and relevant data for the relevant personnel involved in the clinical trial.

⑤ The participating units are all drug clinical trial institutions approved by the state. The leading units have completed the filing of the national stem cell clinical research institutions, and have good clinical trial experience and quality control system.

⑥ The researchers involved in the clinical trial have rich clinical experience, are skilled in periodontal treatment and related surgery, and have obtained GCP training certificates.

11.2 Risk control

The results of the preliminary pharmacological efficacy and toxicology tests show the high safety profile of the test drug, and on this basis, the investigator will:

- Conduct pre-clinical trial training, and focus on training on informed content, injection operation specifications and possible adverse reactions of the product and its treatment.
- Inclusion of eligible subjects in strict adherence to entry/exclusion criteria;
- Closely monitor the subject's vital signs and possible adverse events during treatment;
- Before and after treatment, detailed laboratory tests should be conducted according to the trial process, including blood routine, liver and renal function, coagulation function, inflammatory indicators, immune indicators, etc.

■ Focus on observation and monitoring of local, immune function, hepatic, cardiac, renal and other organ responses and functional changes of periodontal drug administration

■ In the event of adverse events (including but not limited to clinically significant laboratory test abnormalities, injection local haematoma, injection local infection and redness, fever and allergic reaction), the investigator will promptly provide timely and appropriate treatment in accordance with clinical diagnostic and treatment routines and first-aid treatment protocols, and add the necessary laboratory tests according to the clinical needs, and consider whether to continue the trial in the interests of the subjects at all. All adverse events will be followed up until remission or stabilisation. Laboratory abnormalities should be followed until baseline levels are restored and/or a clear cause is identified.

11.3 Risk treatment

Address possible adverse reactions and serious adverse reactions:

■ Treatment of haematoma: local haematoma has appeared, can be immediately compressed to stop bleeding, and give cold compresses; after 48h hot compresses, to promote the absorption of haematoma dissipation. Antibiotics and haemostatic drugs can be given as appropriate.

■ Treatment of infection: Strict sterilisation of injection instruments and injection areas should be adhered to, and injections should be avoided through or directly in the inflamed area. Those who have been infected should be treated according to the principles of treatment of inflammation.

■ Anaphylactic reaction treatment: during the screening period, pay attention to the collection of drug allergy history of the subjects. When acute allergic reaction occurs, the treatment shall be conducted according to the drug clinical trial institution according to the Standard Operating Procedures for Allergy Emergency Collection (SOP) of the drug clinical trial institution.

12.Expected time limit for completion of clinical trials

This clinical trial is planned to occur in one center, and the overall expected time limit of completion is one year.

Annex 1: References:

[1]Hernández-Monjaraz B, Santiago-Osorio E, Ledesma-Martínez E, Alcauter-Zavala A, Mendoza-Núñez VM. Retrieval of a periodontally compromised tooth by allogeneic grafting of mesenchymal stem cells from dental pulp: A case report. J Int Med Res. 2018 Jul;46(7): 2983-2993. doi: 10.1177/0300060518773244. Epub 2018 Jun 18. PMID: 29911458; PMCID: PMC6124270.
[2]Study of Local Periodontal Regeneration of Chronic Periodontal Disease Patients Receiving Allogeneic Human Dental Pulp Stem Cells Injection Therapy; NCT02523651
[3]Loesche WJ, Grossman NS. Periodontal disease as a specific, albeit chronic, infection diagnosis and treatment. Clin Microbiol Rev. 2001;14(4): 727-752.
[4]Gronthos S, Mankani M, Brahim J, et al. Postnatal human dental pulp stem cells (DPSCs) in vitro and in vivo. Proc Natl Acad Sci USA, 2000;97(25):13625-13630.
[5]Marquez-Curtis LA, Janowska-Wieczorek A, McGann LE, et al. Mesenchymal stromal cells derived from various tissues: Biological, clinical and cryopreservation aspects. Cryobiology. 2015 Oct;71(2):181-97.
[6]Egusa H, Sonoyama W, Nishimura M, et al. Stem cells in dentistry--part I: stem cell sources. J Prosthodont Res. 2012 Jul;56(3):151-65.
[7]Feng F, Akiyama K, Liu Y, Yamaza T, et al. Utility of PDL progenitors for in vivo tissue regeneration: a report of 3 cases. Oral Dis. 2010 Jan;16(1):20-28.
