# Supplementary material for: Impact of allogeneic dental pulp stem cell injection on tissue regeneration in periodontitis: a multicenter randomized clinical trial
Source: Signal Transduct Target Ther. 2025 Jul 31;10:239. doi: 10.1038/s41392-025-02320-w (PMC12311062; doi:10.1038/s41392-025-02320-w)
Supplement: Supplementary file 4 — Supplementary File 3 [file 41392_2025_2320_MOESM4_ESM.pdf]

Beijing SH Biotechnology Co.,Ltd

Clinical Study of Allogenic Human Dental Pulp Stem Cells for Treatment of Chronic Periodontitis

(Randomized, Open-label, Controlled Clinical Study of Dental Pulp Mesenchymal Stem Cell injection/preparation with Basic Periodontal Treatment for Moderate Chronic Periodontitis)

hDPMSC-CP-03-2019

Statistical Analysis Plan

Version: 2.0

Date: March 22, 2023

Sponsor Approval Page

Clinical Study of Allogenic Human Dental Pulp Stem Cells for Treatment of Chronic Periodontitis

(Randomized, Open-label, Controlled Clinical Study of Dental Pulp Mesenchymal Stem Cell injection/preparation with Basic Periodontal Treatment for Moderate Chronic Periodontitis)

hDPMSC-CP-03-2019

Statistical Analysis Plan

Version: 2.0

Project Statistician: Yongchao Liu

Company: Jiaxing Clinflash Healthcare Technology

Signature: ______________________ Date: ______________________

Reviewing statistician: Weiyu Wang

Company: Jiaxing Clinflash Healthcare Technology

Signature: ______________________ Date: ______________________

Sponsor APPROVER: Jiawen Gao

Company: Beijing SH Biotechnology Co.,Ltd

Signature: ______________________ Date: ______________________

Revision History

Version	Date	Author	Description
1.0	January 10, 2022	Sha Tao	Finalized
2.0	March 22, 2023	Yongchao Liu	1. Updated MedDRA and drug dictionary versions;<br>2. Added PPS, baseline data and efficacy analyses based on both FAS and PPS; modified changes to planned analysis: "PPS definition was updated"

Catalogue

1. Introduction 6
2. Trial Overview....6
3. Estimation Objectives....10
4. Sample Size....10
5. Analysis Sets....10
6. Statistical Analysis Methods....11
6.1. Overall Statistical Considerations....11
6.2. Data Processing Methods....11
6.2.1. Early Termination and Missing Data....11
6.2.2. Derived and Transformed Data....11
6.3. Subject Disposition Analysis....11
6.4. Demographics and Baseline Characteristics Analysis....11
6.5. Compliance and Concomitant Medications....12
6.6. Primary Analyses....12
6.6.1. Primary Estimation Methods....12
6.6.2. Sensitivity Analysis Methods....12
6.7. Secondary Analyses....12
6.8. Exploratory Analyses 12
6.9. Safety Analyses....12
6.9.1. Drug Exposure....12
6.9.2. Adverse Events....12
6.9.3. Lab Test Results....13
6.9.4. Electrocardiograms....13
6.9.5. Other Safety Assessments....13
6.10. Subgroup Analyses.... 13
6.11. Supplementary Analyses 13
7.... Multiplicity Considerations....14
8. Interim Analysis....14
9. Changes to Planned Analyses....14
10. References....14

Abbreviations

Abbreviation	Definition
Anti-HCV	Hepatitis C Virus Antibody
Anti-TP	Treponema Pallidum Antibody
CMV-IgM	Cytomegalovirus IgM
CMV-IgG	Cytomegalovirus IgG
CRF	Case Report Form
FAS	Full Analysis Set
HBcAb	Hepatitis B Core Antibody
HBeAb	Hepatitis B e Antibody
HBeAg	Hepatitis B e Antigen
HBsAb	Hepatitis B Surface Antibody
HBsAg	Hepatitis B Surface Antigen
HIVcombin	Human Immunodeficiency Virus Antigen/Antibody
IgA	Immunoglobulin A
IgE	Immunoglobulin E
IgG	Immunoglobulin G
IgM	Immunoglobulin M
LOCF	Last Observation Carried Forward
MedDRA	Medical Dictionary for Regulatory Activities
NMPA	National Medical Products Administration
PK	Pharmacokinetics
PPS	Per Protocol Set
PT	Preferred Term
SAE	Severe Adverse Event
SAS	Statistical Analysis Software
SOC	System Organ Class
SS	Safety Set
TEAE	Treatment Emergent Adverse Event
WHO	World Health Organization

1. Introduction

This statistical analysis plan is written for the clinical study "Clinical Study of Allogenic Human Dental Pulp Stem Cells for Treatment of Chronic Periodontitis (Randomized, Open-label, Controlled Clinical Study of Dental Pulp Mesenchymal Stem Cell Injection with Basic Periodontal Treatment for Moderate Chronic Periodontitis)" (Protocol No.: hDPMSC-CP-03-2019) conducted by Peking University School and Hospital of Stomatology. It provides a detailed description of the statistical analysis content and methods.

This statistical analysis plan is developed based on Protocol Version 7.0 (July 16, 2021), Case Report Form (CRF) Version 2.1 (March 16, 2022), China National Medical Products Administration (NMPA) Guidelines on Data Management and Statistical Analysis Plan and Reporting for Clinical Trials, and NMPA Technical Guidelines on Biostatistics for Clinical Trials.

2. Trial Overview

Objectives

The primary objective of this trial is to evaluate the safety and efficacy of human dental pulp mesenchymal stem cell injection for the treatment of moderate chronic periodontitis, and provide evidence for establishing the clinical protocol of dental pulp mesenchymal stem cells for moderate chronic periodontitis.

Efficacy Endpoint Assessment

Main Indicators:

【Oral Clinical Examination Indicators】During the screening period, pre-medication, follow-up at D90±3, D180±5, D360±5, examination was conducted using Florida probes, with the measurement values at D90±3 as the evaluation endpoint.

◆ Periodontal attachment level AL (probing depth PD + gingival recession GR).

Secondary efficacy evaluation indicators:

【Imaging Examination Indicators】During the screening period and follow-up at D90±3, D180±5, D360±5, examination was conducted, using the screening period indicators as baseline and the measurement values at D180±5 as the evaluation endpoint.

◆ Changes in periodontal defect height at 3 months, 6 months, and 12 months after treatment compared to baseline (CBCT measurement results, periodontal defect height change = the difference between cemento-enamel junction to alveolar ridge crest distance).

◆ Changes in alveolar ridge average density at 3 months, 6 months, and 12 months after treatment compared to baseline (CBCT measurement results).

【Oral Clinical Examination Indicators】During the screening period, pre-medication, follow-up at D90±3, D180±5, D360±5, examination was conducted using Florida probes, with the measurement values at D90±3 as the evaluation endpoint.

◆ Changes in periodontal probing depth PD compared to baseline.

◆ Changes in periodontal healing status (bleeding on probing index BOP, gingival recession GR, tooth mobility TM) compared to baseline.

Safety indicators:

Adverse events and serious adverse events during the study period were recorded in a timely manner, and the severity of adverse events was determined according to NCI CTCAE v4.03 grading standards. Gum swelling and pain within one week after surgery were recorded as adverse events, and the researcher judged whether they were drug-related adverse reactions.

CRF collected information such as date of birth, gender, ethnicity, height, weight, current medical history, allergic history, family history, and past medical history.

CRF collected the results of serum pregnancy tests for subjects (limited to premenopausal female subjects).

CRF collected the names, start dates, whether continued use, end dates, administration routes, administration frequency, daily total dose, and indications or reasons for past/combined medication.

Study Design:

This study is a single-center, randomized, open-label, controlled study. A total of 96 subjects are planned to be enrolled in two phases at Beijing Stomatological Hospital, Capital Medical University. After obtaining informed consent from subjects diagnosed with chronic moderate periodontitis who meet the inclusion criteria and do not meet the exclusion criteria, the researcher selected the intended treatment site (single tooth site/subject). All subjects received basic periodontal therapy and immediately received bilateral multi-point local injection treatment with hDP-MSC cells/physiological saline for a single tooth site upon completion of basic periodontal therapy. All subjects were observed for 2 hours after receiving study treatment, and continuous follow-up was conducted to collect clinical safety and efficacy observations until the end of the 12-month study.

In the first stage, 46 subjects were randomly divided into 2 groups (n = 23 each) : DPSCs injection group (1×10^7 hDP-MSC cells (0.6mL normal Saline)/tooth site/person) and Saline injection group (0.6mL normal saline/tooth site/person). In phase 2, 50 subjects were enrolled according to a 1:1 ratio: The rats were randomly divided into three groups: Saline injection group (0.6mL normal saline/tooth), Single DPSCs injection group (1×10^7 hDP-MSC (0.6mL normal saline)/tooth) and Double DPSCs injection group injection group (1×10^7 hDP-MSC cells (0.6mL normal saline suspension)/tooth site/person, and the same dose of drug was injected again one month later).

Test Flow Chart

serial number	Visiting Program		Screening enrollment and treatment period		Study follow-up period				Long-term follow-up
			screening period	Baseline/treatment period	1st follow-up visit	2nd follow-up visit	3rd follow-up visit	4th follow-up visit	Long-term follow-up
			(D-14~0)	(D1)	(D7±1，telephone follow-up)	(D30±3)	(D90±3)	(D180±5)	(D360±5)
1	Signature ICF		X						
2	Inclusion/ exclusion criteria		X	X					
3	Demographic information		X						
4	Medical history		X						
5	Vital signs:respiration, heart rate, blood pressure(systolic/diastolic pressure), temperature(axillary), and clinical assessment of generalized conditions		X	X (before injection treatment, 2h±30min after treatment)		X、double DPSCs injection group 2h±30min after injection treatment			
6	Periodontal clinical indicators: periodontal attachment level(AL), periodontal probing depth(PD),probing bleeding index(BOP),gingical recession(GR),dental tooth looseness(TM)		X	X			X	X	X
7	Laboratory test	routine blood test	X	X		X	X	X	
		blood clotting function	X	X					
		liver and kindney function	X	X		X	X	X	
		Hypersensitive C -reactive protein	X	X (before injection treatment, 2h±30min after treatment)		before injection treatment, 2h ±30min after treatment in double DPSCs injection group			
		Infectious disease tests: HBsAg、HBsAb、HBeAg、HBeAb、HBcAb、Anti-HCV、HIVcombin、Anti-TP、CMV-IgM、CMV-IgG	X			X	X	X	
		Immunological：IgA、IgG、IgM、total IgE	X	X (before injection treatment, 2h±30min after treatment)		X、double DPSCs injection group 2h±30min after injection treatment	X	X	X
		Pregnancy test(blood β-HCG)	X	X		X	X	X	
		urine routine	X	X					

**8**	**Periodontal initial therapy: supragingival scaling + subgingival scraping + root planing**	**X（D-14 full supragingival scaling, D-7±1 half subgingival scraping + root planing）**	**X(half subgingival scraping + root planing)**					
**9**	**Study of drug delivery therapy**		**X**		**X（only IIT study double DPSCs injection group）**			
**10**	**Adverse event records**	**X**	**X**	**X**	**X、double DPSCs injection group 2h±30min after injection treatment**	**X**	**X**	**X**
**11**	**Record of combined medication/treatment**	**X**	**X**	**X**	**X**	**X**	**X**	**X**
**12**	**Imaging(CBCT)**	**X**				**X**	**X**	**X**
**13**	**intraoral photography**	**X**	**X**		**X**	**X**	**X**	**X**

Randomization methods and their implementation

Randomization was performed in each phase of the study using block randomization, with random group codes generated by the statistician using SAS 9.4 software. Phase I trail were assigned to the DPSCs injection group and the saline injection group in a 1:1 ration according to the phase code, and the randomization envelopes were retrieved by the statistical management unit at the completion of the phase; IIT study were assigned to the saline injection group, the single DPSCs injection group, and the double DPSCs injection group in a 1:1:3 ratio according to the phase code.

Blindness and blinding measures

This was an open trial and did not involve blingding.

3. Estimated target

None.

4. Sample size

A total of 96 subjects were recruited in this stude, with three groups:the saline injection group, the single DPSCs injection group, and the double DPSCs injection group.

Sample content was calculated using PASS 16 software. The test level a=0.05 and test efficacy 1-b=0.800 were set. The data from the two phases of the study cases in the three groups was approximately equal, and the sample content was calculated according to the formula:

$$n = 2\left[\frac{(t_{1-\alpha/2} + t_{1-\beta})S}{\delta}\right]^2$$

(Where S represents the standard deviation, 8 represents the overall effect variance that is, the difference between the means of the two groups), and n represents the number of cases needed in each group.)

In the phase I trial, there are 2 groups, 46 subjects are expected to be enrolled, roughly according to the ratio of 1:1 randomized into the DPSCs injection group and the saline injection group; in the IIT study, there are 3 groups, 50 subjects are expected to be enrolled, according to the ratio of 1:1:3 randomized into the saline injectiongroup, the single DPSCs injection group, and the double DPSCs injection grouup.

In summary, the two phases of this study, the saline group is expected to include approximately 33 subjects, the single DPSCs injection group approximately 33 subjects are expected to be enrolled, and 30 subjects are expected to be enrolled in the double DPSCs injection group, meeting the requirements for statistical analysis.

5. Analysis set

The analysis sets for this trial included the full analysis set (FAS), the protocol compliant analysis set (PPS), and the safety analysis set(SS).

Full Analysis Set (FAS, Full Analysis Set): for all cases that have been enrolled with the study drug and have completed at least one efficacy assessment.

Per Protocol Set (PPS): a subset of FAS, cases with good adherence, 6 months of clinical observation, and completion of the CRF.

Safety Analysis Set (SS, Safety Set): all cases that received the study drug after enrollment and had at least one safety assessment.

In this study, baseline information was analyzed using FAS and PPS analysis. Primary efficacy indicators and secondary efficacy indicators were analyzed using FAS and PPS.

For the safety analysis, drug exposure, adverse events, vital signs, and laboratory indices were analyzed using SS. Subjects who were terminated early due to adverse events and various non-therapeutic reasons were counted in the safety analysis.

6. Methods of statistical analysis

6.1. Statistical general considerations

The analysis of the study results will include a description of the distribution of subjects, an analysis of the efficacy of the baseline indicators, and an analysis of safety.

All statistical analyses will be completed using SAS version 9.4.

Statistical descriptions of the measurements were performed using the number of cases, mean, standard deviation, median, quartiles, minimum and maximum values, Wilcoxon signed rank test for between-group comparisons, and paired t-test for before-and-after comparisons within groups.

Statistical descriptions of count and rank information were made using rates or component ratios, and comparisons of count information were made using the χ^2 test or the Fisher Exact probability method and the Kruskal-Wallis rank sum test was used for comparison of rank information.

The last non-missing test prior to the first dose of study drug as a baseline.(May be screening period data, e.g., imaging test indicators using screening period indicators as baseline indicators.)

6.2. Data processing methods

6.2.1. Early withdrawal and missing data

Estimation of missing values for the main indicators was done using the last observation carried forward (LOCF, last observation carrying forward) method.Other missing data were not processed.

6.2.2. Derived and converted data

- Age (years) = (date of signing informed consent - date of birth + 1) /365.25, rounded to the nearest whole number.
- Periodontal attachment level AL = probing depth PD + gingival recession GR
- Alveolar bone defect height at each level C=A-B(**where the distance from the enamel bone boundary to the lowest point of the alveolar bone defect is A and the distance from the cemento-enamel junction to the apex of the alveolar ridge is B)**
- Calibrated mean density value of alveolar bone defect area C=(B+1000)*(A2+1000)/(A1 +1000)-1000 (**where the pre-treatment baseline value is A1, the post-treatment baseline value is A2, and the post-treatment mean density value is B)**

The average of the measured data from the three analysts was used as the final evaluation for the alveolar bone defect height and the average density of the alveolar bone defect area, and an analysis of variance was performed to analyze the consistency of the evaluation.

6.3. Distributional analysis of subjects

Descriptive statistical analyses were performed for screening, screening failure, randomization, randomization with medication, failure to administer medication after randomization, completion of treatment, and early termination of subjects in subgroups, and the number of cases and percentages were calculated. The proportions of reasons for screening failure, reasons for failure to administer medication after randomization, and reasons for early termination of treatment were described.

Tabular descriptions of randomization, early termination, and study closure.

Based on all subjects who underwent randomization, calculate the number of cases and percentage of subjects included in FAS, PPS, and SS. List the reasons and percentages for not including FAS, PPS, SS.

6.4. Demographic information and baseline characterization

Information and characterization were analyzed based on FAS and PPS.

Demographic information such as age, gender, ethnicity, height and weight were statistically described.

Statistical descriptions of the medical history information such as current medical history (yes, no), allergy history (yes, no), family history (yes, no), and past history yes,
no) were performed. All the medical histories were categorized statistically. The MedDRA dictionary (version 24.1 or higher) was used to code the current medical history and past history.

Statistical description of the study disease status for infectious disease screening, immunologic screening not screened, normal, abnormal without clinical significance, abnormal with clinical significance).

For the analysis of the baseline periodontal clinical indicators, imaging (CBCT examination) is described in Sections 6.6 and 6.7.

For analysis of the results of baseline laboratory tests and vital signs, see Sections 6.9.3 and 6.9.5.

Tabulation of demographic information, current medical history, allergy history, family history, and past history.

6.5. Adherence and co-medication

SS-based analysis was performed.

The World Health Organization (WHO) Drug Dictionaly (WHODrugGLOBAL(B3) C [V2021SEP] or higher) was used to code the combined medications, and ATC was used to categorize the statistics of the combined medications.

Combined treatments were coded using the MedDRA dictionary (version 24.1 or higher), and combined nonpharmacological treatments were categorized and counted by PT name.

6.6. Major analysis

The validity analysis was based on FAS and PPS.

The computational treatment of the indicators is detailed in Section 6.2.

The main efficacy index was periodontal attachment level AL = probing depth PD + gingival recession GR. The Florida probe was used to obtain the measurements of D90±3, D180±5, and D360 ±5 for each group at the screening, pre-dosing, and follow-up periods. The missing values were first filled using the LOCF method and then evaluated for efficacy based on the filled values at D90±3.

Periodontal attachment levels and their exact 95% confidence intervals were calculated separately for each dose group at each time point.

Comparison of periodontal attachment levels between the two groups at the same time point was performed using the Wilcoxon rank sum test.

The paired t-test was used for within-group comparisons of changes from baseline in periodontal attachment levels before and after treatment.

6.6.1. Main estimation method

None.

6.6.2. Sensitivity analysis methods

None.

6.7. Secondary analysis

Secondary efficacy indicators were changes in periodontal defect height, changes in mean alveolar ridge density, periodontal probing depth PD, and periodontal healing.

Examinations were performed using a Florida probe to obtain measurements of D90±3, D180±5, and D360±5 for each group at the screening, pre-dose, and follow-up periods. Changes in the height of periodontal defects and changes in the average density of the alveolar ridge were evaluated for efficacy at D18015, and the depth of periodontal probing PD and periodontal healing were evaluated for efficacy at D90±3.

The secondary efficacy indices and their exact 95% confidence intervals were calculated separately for each dose group at each time point.

The paired t-test was used for within-group comparisons of changes in secondary efficacy indices before and after treatment.

Comparison of secondary efficacy indicators between the two groups at the same time point was performed using the Wilcoxon rank-sum test.

6.8. Explorator analysis

None.

6.9. Security analysis

6.9.1. Drug exposure

SS-based analysis was performed.

Descriptive statistics on the number of medications administered.

6.9.2. Adverse event

SS-based analysis was performed.Adverse events were coded using the MedDRA lexicon (version 24.1 or higher).

Adverse event(AE): is defined as all adverse medical events that occur in a subject during the course of a clinical study, which may be manifested as signs and symptoms, abnormalities of disease or laboratory tests,and which are not necessarily causally related to treatment. (Surgeries or hospitalizations that existed prior to the subject obtaining informed consent to participate in the clinical study but did not worsen in severity during the course of the study or were planned prior to entry into the study are not classified as AEs.)

The relevance of adverse events to the study drug was determined by a five-level scale of "definitely related, probably related, probably related, probably unrelated, and definitely unrelated," with the first three levels included in the calculation of the incidence of drug-related adverse events.

An event during the course of a clinical study that requires hospitalization, prolongs hospitalization, is disabling, affects the ability to work, is life-threatening or fatal, or results in a congenital malformation. (The term "life-threatening" in the definition means that the subject was at risk of death at the time of the event; it does not mean that the event would have resulted in death if it had been severe.) Summarized by SOC/PT, TEAEs, SAEs, and drug-related TEAEs were summarized separately by group and AE classification, and the number of cases, number of cases, and incidence rate were calculated. If a subject had multiple occurrences of an adverse event for the same standardized term, only one case was counted and counted as the most severe under that standardized term.

The system organ categories (SOC) and/or standardized terms (PT) are presented in order of incidence in the single DPSCs injection group, the double DPSCs injection group, and the saline injection group in descending order of incidence.

6.9.3. Laboratory findings

Analyzed based on SS.

Laboratory tests included changes in quantitative results of pre-, post-, and ive and ode fuminsation of ranius in lictor such a oncurr cases, mean, standard deviation, median, quartiles, minimum, and maximum values were calculated. A cross-classification table was used to describe the change in clinical assessment from pre-drug to post-drug.

Clinical assessment after medication was performed using the 1 most severe of all postdose exams entered the analysis. The definition of most severe was based on clinical assessment, with abnormalities clinically significant > abnormalities not clinically significant > normal.

List subjects with clinically significant post-dose abnormalities.

6.9.4. Electrocardiography

Not available.

6.9.5. Other safety evaluations

SS-based analysis was performed.

Vital Signs

Statistical descriptions of respiratory rate, heart rate, systolic blood pressure, diastolic blood pressure, body temperature, and their changes from baseline were performed for each visit from baseline D1).

Others

Other tests included infectious disease tests, immunologic tests. All abnormal clinically significant results of the above tests are tabulated.

For positive pregnancy test results are tabulated.

6.10. Subgroup analysis

None.

6.11. Supplementary analysis

None.

7. Multiplicity considerations

None.

8. Interim analysis

None.

9. Changes to program plan analysis

Added paired t-tests before and after medication for statistical analysis of key efficacy indicators.

The definition of PPS was modified.

10. Reference

- Guiding Principles for Planning and Reporting of Drug Clinical Trail Data Management and Statistical Analysis, China Food and Drug Administration, July 2016.
- Biostatistical Technical Guidelines for Drug Clinical Trials, China Food and Drug Administration,March 2016.
