# Supplementary material for: Impact of allogeneic dental pulp stem cell injection on tissue regeneration in periodontitis: a multicenter randomized clinical trial
Source: Signal Transduct Target Ther. 2025 Jul 31;10:239. doi: 10.1038/s41392-025-02320-w (PMC12311062; doi:10.1038/s41392-025-02320-w)
Supplement: Supplementary file 5 — Supplementary File 4 [file 41392_2025_2320_MOESM5_ESM.pdf]

Beijing SH Biotechnology Co. Ltd

A randomized, double-blind, dose-escalation, (blank)-controlled, safety, and tolerability clinical trial of periodontal basic therapy combined with human pulpal mesenchymal stem cell injections for the treatment of chronic periodontitis. with human pulpal mesenchymal stem cell injections for the treatment of chronic periodontitis

SH-hDP-MSC-101

Statistical analysis plan

Version: 2.0

Date: January 13, 2023

Sponsor approval page

A randomized, double-blind, dose-escalation, (blank)-controlled, safety, and tolerability clinical trial of periodontal basic therapy combined with human pulpal mesenchymal stem cell injections for the treatment of chronic periodontitis. with human pulpal mesenchymal stem cell injections for the treatment of chronic periodontitis

SH-hDP-MSC-101

Statistical analysis plan version: 2.0

Project Statistician: Liu Yongchao

Company Name. Jiaxing Edixi Computer Technology Co.

Date.

Reviewing Statistician: Wang Weiyu

Company Name. Jiaxing Edixi Computer Technology Co.

Date.

Approved by the Sponsor. He jia

Company Name. Beijing SH Biotechnology Co. Ltd

Date.

Amendment Record

File version	Date	Author	Description
1.0	2022-09-13	Yongchao Liu	Final draft
2.0	2023-01-13	Yongchao Liu	1. Modify the definition of TEAE associated with the investigational drug<br>2.Curative effect analysis: The number of cases was modified to the number of teeth, and the paired t-test was added in the group<br>3.Labial/buccal and lingual/palatine trend plots were drawn

Catalogue

1. Introduction 6
2. Test summary 6
3. Scheduled target 11
4. Sample capacity 11
5. Analytic set 11
6. Statistical analysis method 12
6.1. Statistical population consideration 12
6.2. Data processing method 12
6.2.1. Early exit and missing data 12
6.2.2. Derived and transformed data 12
6.3. Subject distribution analysis 13
6.4. Demographic data and baseline characteristics analysis 13
6.5. Compliance and drug combination 13
6.6. Primary analysis 14
6.6.1. Main estimation method 14
6.6.2. Sensitivity analysis method 14
6.7. Secondary analysis 14
6.8. Exploratory analysis 15
6.9. Security analysis 15
6.9.1. Drug exposure 15
6.9.2. Adverse event 15
6.9.3. Laboratory test results 17
6.9.4. Electrocardiogram 17
6.9.5. Other safety evaluation 17
6.10. Subgroup analysis 17
6.11. Supplementary analysis 17
7. Multiplicity consideration 18
8. Interim analysis 18
9. Changes to the program plan analysis 18
10. Reference 18

Abbreviation

Abbreviation	Paraphrase
AE	adverse event
AL	attachment level
Anti-HCV	antibodies to hepatitis C virus
Anti-TP	antibodies to treponema pallidum
APTT	activated partial thromboplastin time
BI	bleeding index
CBCT	cone beam computed tomography
CMV-IgG	cytomegalovirus immunoglobulinG
CMV-IgM	cytomegalovirus immunoglobulinM
CRC	clinical research coordinator
CRF	case report form
FAS	full analysis set
GR	gingival recession
HBcAb	hepatitis B core antibody
HBeAb	hepatitis B e-antibody
HBsAb	hepatitis B surface antibody
HBsAg	hepatitis B surface antigen
HIVcombin	HIV combination test
INR	international normalized ratio
IWRS	interactive web response system
MedDRA	medical dictionary for regulatory activities
MSC	mesenchymal stem cell
MTD	maximum tolerated dose
NCI-CTCAE	NCI Common Toxicity Criteria Adverse Event
OBT	occult blood test
PD	probing depth
PPS	per protocol set
PT	prothrombin time
SAE	serious adverse event
SS	safety set
TM	tooth mobility

1. Introduction

This statistical analysis plan is written for the "Randomized, double-blind, dose-increasing, (blank) controlled safety and tolerability clinical trial of periodontal basic treatment combined with human pulp mesenchymal stem cell injection for the treatment of chronic periodontitis" (Plan No.: SH-hDP-MSC-101) by Beijing SH Biotechnology Co.,Ltd., Beijing 100070, China. The content and methods of its statistical analysis will be described in detail.

This statistical analysis plan is based on the research protocol version 3.3 dated February 11, 2022 and the case report form (CRF) version 2.0 dated March 17, 2022.

2. Test summary

Research Objective

Main Purpose

- Exploring the safety and tolerability of human pulp mesenchymal stem cells in the treatment of chronic periodontitis

Secondary Purpose

- Dose exploration and providing a design basis for the subsequent clinical study of drug administration plans.
- Exploring the preliminary effectiveness of human pulp mesenchymal stem cells in the treatment of chronic periodontitis

Study endpoint

Main endpoint indicators:

Safety endpoint indicators:

The incidence and severity of adverse events (AE) related to pulp mesenchymal stem cell therapy within 180 days after administration

Secondary endpoint indicators：

Safety endpoint indicators:

- The incidence and severity of adverse events (AE) and severe adverse events (SAE) related to pulp mesenchymal stem cell therapy within 360 and 720 days of administration;

Efficacy endpoint indicators:

- Changes in periodontal attachment level (AL) from baseline on the 90th day after administration
- Changes in periodontal probing depth (PD) from baseline on the 90th day after administration;
- On the 90th day after administration, changes in periodontal healing (probing bleeding index BI, gingival recession GR, tooth looseness TM) compared to baseline;
- Changes in the height of periodontal bone defects from baseline on the 180th day after administration;
- Changes in average alveolar ridge density from baseline on the 180th day after administration.

DesignType

This study is a dose-increasing, randomized, double-blind, (blank) controlled phase I clinical trial. There are a total of 5 dose groups, namely the first dose group (1) × 10^6 cells/periodontal defect site), second dose group (5 × 10^6 cells/periodontal defect site), third dose group (1 × 10^7 cells/periodontal defect site, fourth dose group (2 × 10^7 cells/2 periodontal defect sites and the fifth dose group (3 × 10^7~4 × 10^7 cells/3-4 periodontal defect sites), including 4 subjects in the first dose group and 8 cases in each of the other 4 dose groups, with a total of 36 cases included in the group; Each dose group was randomly assigned to the experimental group or blank control group at a ratio of 3:1.

After a single local injection, the subject was observed for 24 hours and discharged from the hospital. End the study at 6 months (D180 ± 14D) and collect clinical safety and efficacy indicators from the subjects. Long term follow-up was conducted at 12 months (D360 ± 14D) and 24 months (D720 ± 30D) after administration to collect clinical efficacy observation indicators

(1)dose-increasing

Based on preclinical toxicological research data, as well as clinical study designs of similar drugs, and referring to the dosage used in clinical research literature, it is proposed to determine the starting dose of this study as 1 × 10^6 cells/person/time.

The study designed 5 dose groups, with 4 subjects in the first dose group and 8 subjects in each of the other 4 dose groups, administered by a single local injection. The tentative range of dose increase is:

First dose group: 1 × 10^6 cells/periodontal defect sites.

Second dose group: 5 × 10^6 cells/periodontal defect sites

Third dose group: 1 × 10^7 cells/periodontal defect site

Fourth dose group: 1 × 10^7 cells/periodontal defect sites, a total of 2 periodontal defect sites, with a total cell injection volume of 2 × 10^7 cells/2 periodontal defect sites

Fifth dose group: 1 × 10^7 cells/periodontal defect sites, a total of 3-4 periodontal defect sites, with a total cell injection volume of 3 × 10^7~4 × 10^7 cells/3-4 periodontal defect sites.

The subjects were sequentially administered from the low-dose group to the high-dose group according to the principle of increasing dose, with each subject receiving only one corresponding dose. Four subjects were enrolled in the first dose group and randomly assigned to either the experimental group (3 cases) or the blank control group (1 case); The remaining 4 dose groups, each containing 8 subjects, were randomly assigned to either the experimental group (6 cases) or the blank control group (2 cases) for safety and tolerability studies of a single dose

During the research process, it is determined by the researcher that if the subject does not experience any adverse events related to the termination dose increase standard within 2 weeks after receiving the investigational drug injection, they will be assigned to the next dose group. The researchers will determine the subsequent trial arrangement based on the safety status of each participant in each dose group.

(2) Termination dose escalation standard

The severity of adverse events observed in the study was determined according to the NCI CTCAE v5.0 grading criteria. Dose escalation was terminated within 2 weeks after receiving the investigational drug injection, and the previous dose was defined as MTD:

① Half or more of the subjects in any dose group experience drug related adverse events of level 3 or higher;
② If the maximum dose is reached (3 × 107~4 × When 107 cells/3-4 periodontal defect sites were used, no adverse events of grade 3 or more that may be related to the investigational drug occurred in half or more of the subjects. The researchers and the sponsor negotiated to decide whether to continue dose escalation; If the dose does not continue to increase, define this dose group as MTD

(3) Handling of dropout subjects

If the subject falls off within 4 weeks after receiving the investigational drug injection, the subject will be supplemented in the corresponding dose group. If the subject falls off after 4 weeks, it may not be supplemented.

Study flow chart

A randomized, double-blind, dose-increasing, (blank) controlled clinical trial of basic periodontal therapy combined with human pulp mesenchymal stem cell injection for the treatment of chronic periodontitis: safety and tolerability

X	Visiting projects	Screening for inclusion and treatment period					Follow up							
							Follow up after treatment						Long-term follow up	
		screening period	Basic treatment of periodontal disease in the contralateral half of the oral cavity	Distributer andom numbers	medica tion	Treatment observation 11	1st follow-up	2nd follow-up	3rd follow-up	4th follow-up	5th follow-up	Early exit	The first long-term follow-up	The second long-term follow-up
		D-28~D-9	D-8±2	D-1	D1[17]	D2[17]	D7±1D	D14±1D	D30±3D	D90±7D	D180±14D		D360±14D	D720±30D
1	Sign ICF	X												
2	Inclusion/Exclusion Criteria	X	X	X										
3	demographic data (1)	X												
4	Medical history data (2)	X												
5	Vital signs: respiration, heart rate, blood pressure (systolic and diastolic), body temperature	X			X	X	X	X	X	X	X	X		
6	Periodontal clinical indicators examination: periodontal attachment level AL, periodontal probing depth PD, probing bleeding index BI, gingival recession GR, tooth looseness TM (3)		X							X	X	X	X	X
7	Blood routine (4)	X			X	X	X	X	X	X	X	X		
8	Coagulation function (5)	X			X									
9	Liver and kidney function (6)	X			X	X	X	X	X	X	X	X		
10	Inflammatory markers: Hypersensitivity C-reactive protein (7)	X			X									

11	Infectious disease detection: HBsAg、HbsAb、HBeAg、HbeAb、HbcAb、Anti-HCV、HIVcombi、Anti-TP、CMV-IgM、CMV-IgG	X							X	X	X	X		
12	Immunological examination IgA、IgG、IgM、总 IgE	X			X	X	X	X	X	X				
13	pregnancy test（8）	X		X					X	X	X	X		
14	Urinalysis（9）	X			X	X								
15	Urinary routine（10）	X				X								
16	Randomly enrolled			X										
17	Basic periodontal treatment (Supragingival Scaling)	X												
18	Basic periodontal treatment (subgingival scaling, root planing)		X		X									
19	Medication treatment				X									
20	Adverse Event Record 13		X		X	X	X	X	X	X	X	X	X	X
21	Record of combined medication/treatment 14	X			X	X	X	X	X	X	X	X	X	X
22	Imaging Examination (CBCT) 15	X								X	X	X	X	X
23	Electrocardiogram 16	X		X	X	X	X	X	X	X	X	X		

注：

（1） Demographic data: including gender, date of birth, age (one year old), ethnicity, height, weight

（2） Medical history information: current medical history: diagnosis and symptoms; Allergy history, family history, past history (such as smoking history, diabetes history, other surgical history, etc.)

（3） Periodontal clinical indicators examination: periodontal attachment level AL, periodontal probing depth PD, probing bleeding index BI, gingival recession GR, and tooth looseness TM. Within 2 hours before basic treatment of the contralateral half oral periodontium, examinations were conducted by a blinded physician, including D90 ± 7D, D180 ± 14D, D360 ± 14D, and D720 ± 30D. The clinical examination indicators collected at the time point of basic treatment (D-8 ± 2) for the study of the contralateral half oral periodontium were used as baseline indicators (PD and TM examinations at this visit point were only used as baseline values and not as inclusion criteria), and the efficacy indicator was observed to be D180 ± 14D

（4） Blood routine: including hemoglobin, red blood cell, white blood cell, neutrophil count, lymphocyte count, and platelet count. During the screening period, within 2 hours before administration, and 24 hours ± 30 minutes after administration, follow-up examinations were conducted at D7 ± 1D, D14 ± 1D, D30 ± 3D, D90 ± 7D, and D180 ± 14D.

（5） Coagulation function: prothrombin time (PT), activated partial thromboplastin time (APTT), international standardized ratio (INR). Check within the screening period and 2 hours before administration

（6） Liver and kidney function: total bilirubin, direct bilirubin, alanine aminotransferase, aspartate aminotransferase, total protein, albumin, total bile acid, urea, creatinine, uric acid, glucose, potassium, sodium, chlorine. During the screening period, within 2 hours before administration, and 24 hours ± 30 minutes after administration, follow-up examinations were conducted at D7 ± 1D, D14 ± 1D, D30 ± 3D, D90 ± 7D, and D180 ± 14D.

（7） Inflammatory index detection: Hypersensitivity C-reactive protein, reference value of whole blood hypersensitivity C-reactive protein in healthy adults is less than 5mg/L. Check during screening period, within 2 hours before administration. and within 2 hours ± 5 minutes after administration

（8） Pregnancy test: female subjects; During the screening period, distribution of random number D-1, and follow-up period D30 ± 3D, D90 ± 7D, and D180 ± 14D examinations. (Only the screening period is blood pregnancy, the rest are urine pregnancy tests.)

（9） Urinary routine: including urine specific gravity, pH, urine glucose, urine protein, urine white blood cells (qualitative), urine ketone bodies, bilirubin, and urine occult blood (qualitative). Examination during screening period, within 2 hours before administration, within 2 hours after administration, and within 24 hours ± 30 minutes

（10） Routine fecal test: including fecal characteristics, fecal white blood cells, fecal red blood cells, fecal color, and fecal occult blood test (OBT). Check within the screening period and 24 hours after administration.

（11） The observation period for treatment is 24 hours after the subject receives injection of the investigational drug.

（12） Before undergoing basic periodontal treatment (gingival scaling) during the screening period, the subjects need to undergo periodontal probing depth PD and tooth looseness TM testing. PD and TM examinations are used as inclusion criteria for evaluation.

（13） Adverse event recording: Adverse events of D360 ± 14D and D720 ± 30D are only recorded as ADRs.

（14） Record of concomitant medication/treatment: D360 ± 14D and D720 ± 30D only record concomitant medication for ADR.

（15） Imaging examination (CBCT): During the screening period, follow-up examinations include D90 ± 7D, D180 ± 14D, D360 ± 14D, and D720 ± 30D. Using the screening period indicator as the baseline indicator, the efficacy indicator was observed to be D180 ± 14D. Data collection was conducted by Peking University Third Hospital, and analysis was conducted by the Radiology Department of Beijing Stomatological Hospital, Capital Medical University

（16） Electrocardiogram: Examination during screening period, distribution of random number D-1, 2h ± 30min, 24h ± 30min after administration, follow-up period D7 ± 1D, D14 ± 1D, D30 ± 3D, D90 ± 7D, D180 ± 14D

（17） The specific time for vital sign examination here is within 2 hours before administration, 30 minutes ± 5 minutes after administration, 2 hours ± 5 minutes, and 24 hours ± 30 minutes; The specific time for immunological examination is within 2 hours before administration and within 24 hours ± 30 minutes after administration

Randomization Methods and Double Blindness

Randomization:

This study used a fully randomized method, using SAS statistical analysis software version 9.4 or above to generate a random table and the corresponding groups of the random table. The clinical trial electronic central randomization system (IWRS) was used to assign random numbers. Each dose group was randomly assigned separately. Each qualified subject will receive a random number from small to large according to the screening number on D-1 and be assigned to the corresponding group.

Double blind:

To reduce or control bias, this study adopted a double-blind design. Due to dosage form reasons, the injection appearance of the experimental drug and the blank control drug is slightly different. Therefore, this study will establish a non blind group to ensure that other relevant researchers remain blind during the study period. The non blind team members include: non blind physicians, non blind nurses, non blind CRCs, non blind drug administrators, and non blind monitors, responsible for the reception, distribution, administration, recovery, recording, data monitoring, and other related work of experimental drugs. Non blind group members will not undergo any evaluation during the study period. All research physicians are required to conduct a consistency evaluation before participating in the study. Members with a consistency score of ≥ 85% can proceed to the next step of the study. To better maintain blindness, non blinding physicians should use simulated blindness (such as using an eye mask) for the subjects.

Emergency unblinding:

In emergency situations, when researchers believe that knowing the drugs used by the subjects is beneficial for the management of adverse events, IWRS can be used for emergency unblinding.

Unblinding regulations:

This study used a one-time unblinding method. Blinding is carried out after statistical analysis is completed, and the drugs corresponding to the group code are revealed. The unblinding documents are jointly signed by the main researchers, applicants, and statisticians.

3. Expected target

Not have

4. Sample size

This experiment is expected to enroll 36 qualified subjects with chronic periodontitis.

5.Analysis Set

The analysis set includes Full Analysis Set (FAS), Per Protocol Population Set (PPS), and Safety Analysis Set (SS).

Full Analysis Set (FAS)

All cases who signed informed consent, were enrolled and treated with this trial product constitute the complete analysis set

Per Protocol Population Set (PPS)

All cases in FAS that comply with the trial protocol, have good compliance without major protocol violations, have not taken prohibited drugs during the trial period, and have completed the CRF prescribed filling form the analysis set that complies with the protocol.

Safety Set（SS）

All cases enrolled and using the trial product, with post-treatment safety evaluation data, constitute the safety analysis set of this trial.

Security analysis is conducted using the Security Analysis Set (SS).

The validity analysis was conducted using both the Full Analysis Set (FAS) and the Protocol Compliance Analysis Set (PPS).

6. Statistical analysis methods

6.1 Overall statistical considerations

This experiment is a single dose study, with dose groups consisting of the first dose group, the second dose group, the third dose group, the fourth dose group, and the fifth dose group. Each dose group subject is randomly assigned to the experimental group or blank control group at a ratio of 3:1. The blank control groups of each dose group will be combined for analysis. Each dose group name starts with 1 × 10^6 cells, 5 × 10^6 cells, 1 × 10^7 cells, 1 × 10^7 cells (2 tooth positions), 1 × 10^7 cells (3-4 tooth positions) and a blank control group were described.

Unless otherwise specified, the statistical analysis software will use SAS statistical analysis software version 9.4 or higher. The description of quantitative indicators will calculate mean, standard deviation, median, minimum, maximum, and interquartile range. The description of qualitative indicators will calculate the number and percentage of cases for each classification. Use appropriate statistical tables and graphs to summarize safety. Describe the number of subjects enrolled in each dose group, the situation of dropout cases, and conduct descriptive statistical analysis on the baseline characteristics of enrolled cases.

6.2 Data processing methods

6.2.1 Early exit and missing data

The missing dates for AE are supplemented as follows. The filling of missing dates for AE is only used for classifying AE, and the unfilled dates are still displayed when generating an AE related data list.

- AE start date is missing

1) If the year and month are known and the year and month are less than the year and month of the first administration of the investigational drug, the last day of the known month shall be used for filling.

2) If the year and month are known and the year and month are equal to the year and month of the first administration of the investigational drug, then the start date of the AE is equal to the date of the first administration of the investigational drug (date refers to "xx month xx day").

3) If the year and month are known and the year and month are greater than the year and month of the first administration of the investigational drug, the first day of the known month is used to fill in.

4) If only the year is known and the year is less than the year of the first administration of the investigational drug, use "December 31st" to fill in.

5) If only the year is known and the year is equal to the year of the first administration of the investigational drug, then the start date of AE is equal to the date of the first administration of the investigational drug (date refers to "xx month xx day").

6) If only the year is known and the year is greater than the year of the first administration of the investigational drug, use "January 1st" to fill in.

7) If the year, month, and day are missing, the date of the first administration of the investigational drug shall be taken as the corresponding start date.

8) Other situations are considered missing.

- AE end date is missing

1) If the year and month are known, use the last day of the known month to fill in.

2) If only the year is known, use "December 31st" to fill in.

3) If the completed start date is after the end date, the end date will be used as the corresponding start date.

4) Other situations are considered missing.
Missing data will not be processed.

6.2.2 Derived and transformed data

Periodontal attachment level AL=periodontal probing depth PD+gingival recession GR

Note: When the gum is at the root of CEJ, AL=PD+| GR |

When the gums are in the CEJ crown, AL=PD - | GR |.

6.3 Analysis of Subject Distribution

Descriptive statistical analysis was conducted on subjects who underwent screening, failed screening, enrollment, administration after enrollment, no administration after enrollment, early withdrawal, and end of study by grouping, and the number and percentage of cases were calculated.
Describe the main reasons for screening failure and the proportion of reasons for early withdrawal from the experiment. Provide a list description of enrollment and study completion status.
Based on all randomized subjects, list the cases where the protocol deviates from the subjects.
Based on all randomized subjects, calculate the number and percentage of subjects included in the Full Analysis Set (FAS), Protocol Compliant Analysis Set (PPS), and Safety Data Set (SS). List the reasons and proportions for not being included in FAS, PPS, and SS.

6.4 Demographic data and baseline feature analysis

Analysis based on FAS

Demographic information

Statistical description of age, gender, ethnicity, weight, and height

Previous or current medical history

Encode using MedDRA dictionary 22.1 or higher and classify all medical histories according to organ classification terms (SOC) and preferred term (PT) names.
List subjects with a history of past or current illness.

Blood pressure history

List a list of situations with the history

Allergy history

List a list of situations with the history

Family history

List a list of situations with the history

Smoking history

List a list of situations with the history

History of diabetes

List a list of situations with the history

Surgical history

Encode all surgical histories using MedDRA dictionary 22.1 or higher, and classify them according to organ classification terms (SOC) and preferred term (PT) names.

List a list of situations with a history of surgery.

6.5 Compliance and concomitant medications

Analysis based on SS

concomitant medication

The concomitant medication is encoded using WHODrugV2020MAR or higher version. Drugs that are still being used or newly added after starting treatment, except for the investigational drugs. Calculate the number of cases and percentage of subjects using various ATC drugs, using the lowest level of ATC classification encoded. List merge medication.

combined therapy

List.

6.6 Main Analysis

Validity analysis is based on both FAS and PPS simultaneously.

The main therapeutic outcome measure is periodontal attachment level AL (probing depth PD+gingival recession GR), with the full mouth clinical test indicators collected at the time point of basic periodontal treatment (D-8 ± 2) for the contralateral half of the tooth as the baseline indicator.

List the baseline, 90 days post administration, 180 days post administration, 360 days post administration, and 720 days post administration periodontal attachment level AL (probing depth PD+gingival recession GR), as well as the number of teeth, mean, standard deviation, median, quartile, maximum, and minimum values that change from baseline. Use variance analysis for inter group comparison, paired t-test for intra group comparison, and provide P-values.

Draw a trend chart of the mean and standard deviation of periodontal attachment level AL (probing depth PD+gingival recession GR) on the lip/cheek and tongue/palate sides.

List the main efficacy indicators

6.6.1 Main estimation method

Not have

6.6.2 Sensitivity analysis methods

Not have

6.7 Secondary analysis

The secondary efficacy indicators are CBCT examination (periodontal bone defect height and average alveolar ridge density), with screening period indicators as baseline indicators; Periodontal probing depth (PD), periodontal healing status (probing bleeding index BI, gingival recession GR, tooth looseness TM), were collected as baseline indicators at the time point of basic periodontal treatment (D-8 ± 2) in the contralateral half of the oral cavity.

Imaging examination_ CBCT

(1) Height of periodontal bone defect

The formula for calculating the height of alveolar bone defect is C=A-B.
Note: The distance from the enamel cementum boundary to the lowest point of the alveolar bone defect is A, and the distance from the enamel cementum boundary to the vertex of the alveolar ridge is B.

List the baseline, 90 days after administration, 180 days after administration, 360 days after administration, and 720 days after administration of the periodontal bone defect height, as well as the number, mean, and standard deviation of teeth that change from baseline Median, quartile, maximum, and minimum values are compared between groups using analysis of variance, paired t-tests are used for intra group comparison, and P-values are provided.

Draw a trend chart of the mean height and standard deviation of periodontal bone defects

(2) Average density of alveolar ridge

The calculation formula for the average density of alveolar ridges is: C=(B+1000) * (A2+1000)/(A1+1000) -1000.
Note: The baseline value before treatment is A1, the baseline value after treatment is A2, and the average density value after treatment is B.
List the average density of alveolar ridges at baseline, 90 days after administration, 180 days after administration, 360 days after administration, and 720 days after administration, as well as the number of teeth, mean, standard deviation, median, quartile, maximum, and minimum changes from baseline, Use analysis of variance for inter group comparison, paired t-test for intra group comparison, and provide P-values.

The average value of the measurement data from three analysts was used as the final evaluation result for the height of alveolar bone defect and the average density of alveolar bone defect area, and an analysis of variance was conducted to verify the consistency of the evaluation by the three analysts.

Draw a trend chart of the average density and standard deviation of the alveolar ridge.

Periodontal probing depth (PD):

List the baseline, 90 days after administration, 180 days after administration, 360 days after administration, and 720 days after administration, as well as the number of teeth, mean, standard deviation, median, quartile, maximum, and minimum changes from baseline. Use analysis of variance for inter group comparison, paired t-test for intra group comparison, and provide P-values. Draw a trend chart of the mean and standard deviation of periodontal probing depth PD on the lip/cheek and tongue/palate sides.

Periodontal healing status

Descriptive statistics were conducted on the periodontal healing status (probing bleeding index BI, gingival recession GR, tooth looseness TM) relative to baseline changes at 90 days after administration, 180 days after administration, 360 days after administration, and 720 days after administration. The gingival recession GR and the number of teeth, mean, standard deviation, median, quartile, maximum, and minimum changes from baseline were listed. ANOVA was used to compare the gingival recession GR between groups Paired t-test for intra group comparison, Wilcoxon rank sum test for intra group and inter group comparison of probing bleeding index BI and tooth looseness TM, and providing P-values.

Draw a trend chart of the mean and standard deviation of gingival recession on the lip/cheek and tongue/palate sides. List secondary efficacy indicators.

6.8 Exploratory Analysis

Not have

6.9 Safety Analysis

6.9.1 Drug exposure

Analyze based on the security analysis set.

Descriptive statistics are conducted on the number of times medication is administered in groups for a single administration.

List the situation of basic periodontal treatment

6.9.2 Adverse event

Definition

Adverse events (AE) refer to all adverse medical events that occur in clinical trial subjects after receiving the investigational drug, but it may not necessarily be inferred that there is a clear causal relationship with the investigational drug. Adverse events can manifest as symptoms, signs, diseases, or laboratory abnormalities, including the following:
(1) worsening of the original (before entering clinical trials) medical condition/disease (including worsening of symptoms, signs, and laboratory abnormalities);
(2) Any newly occurring adverse events: any newly occurring adverse medical conditions (including symptoms, signs, newly diagnosed diseases);
(3) Abnormal laboratory test values or results with clinical significance, and not caused by accompanying diseases.

Adverse events during treatment (TEAE): Adverse events that begin or worsen after receiving the study medication for the first time.
Serious Adverse Event (SAE) refers to the occurrence of any one or more of the following criteria by a subject after receiving the investigational drug, which is considered a serious adverse event:

- Causing death;

- Endangering life (defined as an AE that causes immediate death of the subject when it occurs, but does not include an AE that only causes death after further development);

- Resulting in permanent or severe disability/loss of function;
- The subject necessitates hospitalization or extended hospitalization;
- Causes congenital abnormalities/birth defects.
- Other significant medical events: Medical and scientific judgment must be employed to determine whether to expedite the reporting of other situations, such as important medical events that may not immediately pose a threat to life, death, or require hospitalization but are generally considered serious if medical measures are necessary to prevent one of the aforementioned outcomes. Examples of these events include, but are not limited to, allergic bronchospasm requiring intensive treatment in the emergency room or at home, hematological exoxia or convulsions not warranting hospitalization, potential drug-induced liver injury, suspected transmission of pathogens (pathogenic or non-pathogenic) via experimental drug products, pregnancy complications, drug overdose incidents, secondary tumors etc.

Adverse event severity grading criteria

The severity of AEs in this study was graded according to the Common Time Criteria for the Evaluation of Adverse Events (CTCAE v5.0), and the AE intensity was graded as follows:
Grade 1: Mild; asymptomatic or mild; seen only clinically or diagnostically; no treatment required.
Grade 2: Moderate; requires minor, local or non-invasive treatment; age-appropriate instrumental activities of daily living (IADLs) are limited. Limitations in instrumental activities of daily living (instrumental activities of daily living are cooking, shopping for clothing, using the telephone, managing money, etc.).
Grade 3: Severe or medically significant but not immediately life-threatening; resulting in hospitalisation or prolonged hospitalisation; Disabling; limiting activities of daily living (activities of daily living are bathing, dressing and undressing, eating, washing, taking medication, and not being bedridden). medication, and is notbedridden).
Grade 4: Life-threatening, requiring urgent treatment.
Grade 5: AE-related death.

Correlation of adverse events with trial drug applications

Based on the criteria for determining the causal relationship between the drug and the adverse event, the correlation between the adverse event and the application of the test drug was classified into the the following five levels, definitely related, probably related, probably related, probably not related, and definitely not related. Definitely related, probably related, possibly related, probably unrelated, and definitely unrelated are classified asdrug-related TEAEs.

① Definitely related: there is evidence of the use of the test drug. The occurrence of adverse events and the use of test drugs have a reasonable time sequence; instructions, similar drugs or literature to support; discontinuation or reduction of the reaction to reduce or disappear; re-administration (if feasible) of adverse events reoccur; has been ruled out the influence of other confounding factors such as the original disease, the explanation of the experimental drug is more reasonable than the other reasons.

② Likely to be related: evidence of use of the test drug. There is no history of re-use of the drug, the rest of the same as "certainly", or although there is a combination of drugs, but basically exclude the possibility of adverse events caused by the combination of drugs.

③Possibly related: Evidence of use of the test drug. The occurrence of adverse events and the use of experimental drugs have a reasonable time sequence; instructions, similar drugs or literature to support; not clear whether the adverse events of re-administration of drugs again The adverse event occurred again; more than one drug was involved in the adverse event, or the progression of the original disease cannot be excluded.

④Possibly unrelated: evidence of use of the test drug. There is a reasonable time sequence between the occurrence of the adverse event and the use of the investigational drug; the adverse event does not coincide with known adverse reactions in the specification, similar drugs, or the literature; it is not clear whether the adverse event will recur with reuse of the investigational drug; and the occurrence of the adverse event may be better explained by other reasons.

⑤ Definitely unrelated: No test drug was used; or there is evidence of test drug use, but the use of the test drug and the timing of the adverse event are not consistent with the use of the test drug.

Analyses are performed based on SS. Adverse events will be graded according to CTCAE v5.0 and coded using the MedDRA dictionary (version: V22.1 or later).

TEAEs, TEAEs related to the trial drug, SAEs, TEAEs leading to discontinuation of the drug, and TEAEs leading to withdrawal from the study will be summarised by group, and the number of cases, number of cases and incidence will be calculated.

The number of TEAEs, TEAEs related to the test drug, SAEs, TEAEs leading to discontinuation of the drug, TEAEs leading to withdrawal from the study, and the incidence rate were summarised according to the System Organ Class (SOC) and Preferred Term (PT) of the human organ system. If multiple occurrences of the same adverse event occurred in a subject, they were statistically analysed according to the most severe severity.

Tabular descriptions of TEAEs occurring in each dose group, TEAEs related to the test drug, SAEs, TEAEs leading to discontinuation of the drug, and TEAEs leading to withdrawal from the study.

6.9.3. Laboratory test results

Analyses were performed based on the safety analysis set.

Laboratory tests include blood routine, liver and kidney function,coagulation function and other indicators before, after and relative to the pre-treatment changes. Post-treatment changes relative to pre-treatment, and the number of cases, mean, standard deviation, median, quartile, minimum and maximum values were calculated.

A cross-tabulation table was used to describe the changes in clinical assessment from pre-treatment to post-treatment. Post-treatment clinical assessments were performed using 1 of the most severe of all posttreatment examinations into the analysis. The definition of the most severe was based on the clinical assessment, with abnormalities clinically significant > abnormal not clinically significant > normal.

Pre-treatment normal, post-treatment abnormal, and both pre- and post-treatment abnormal are listed.

6.9.4. Electrocardiogram

Safety analyses were performed based on the Safety Analysis Set.

A cross-classification table was used to describe the change in ECG findings from pre-treatment to post-treatment. Post-treatment results were entered into the analysis using the 1 most severe of all post-treatment examinations. The definition of the most severe was classified according to the test result, abnormal with clinical significance > abnormal without clinical significance > normal.

Pre-treatment normal post-treatment abnormalities and pre- and post-treatment abnormalities are listed.

6.9.5 Other safety evaluations

Perform analyses based on the safety analysis set.

<u>Vital signs</u>

Describe the change in each test pre-treatment, post-treatment, and post-treatment relative to pre-treatment by calculating the number of cases, mean, standard deviation, median, quartiles, minimum, and maximum values.

Make a list of the subject's vital signs.

<u>Blood pregnancy tests</u>

List subjects who did not have a test or who had a positive test result.

<u>Urine Pregnancy Tests</u>

List of subjects not checked or with positive test results.

6.10 Subgroup analyses

None.

6.11 Additional analyses

None.

7. Multiplicity considerations

None.

8. Interim analyses

This study proposes to conduct an interim analysis of the efficacy and safety data of the enrolled subjects after 36 subjects have completed dosing and D180 follow-up, and to complete an interim

analysis report.**9 Changes to the programme plan analysis**

None.

10. References

●State Food and Drug Administration "Guidelines for Planning and Reporting of Data Management and Statistical Analysis in Drug Clinical Trials", July 2016

●State Food and Drug Administration, Guiding Principles on Biostatistical Techniques for Drug Clinical Trials, March 2016
