# Supplementary material for: Impact of allogeneic dental pulp stem cell injection on tissue regeneration in periodontitis: a multicenter randomized clinical trial
Source: Signal Transduct Target Ther. 2025 Jul 31;10:239. doi: 10.1038/s41392-025-02320-w (PMC12311062; doi:10.1038/s41392-025-02320-w)
Supplement: Supplementary file 6 — Supplementary File 5 [file 41392_2025_2320_MOESM6_ESM.pdf]

According to the latest diagnostic criteria for chronic periodontitis—AL index analysis

Results of phase I clinical trial + IIT study project data combined with stratified statistical analysis

**Sponsor**	Beijing SH Biotechnology Co.,Ltd.
**Author**	Tianjin Clin - nov Medical Technology Co.,Ltd
**Name**	TFLs
**Version number**	1.0
**Version date**	04/26/2023

Catalogs

1. Analysis of the data of the Beijing Stomatology Hospital,Cpital Medical University + Department of Stomatology,Peking university Third Hosipital..3

Table1.1 Summary distribution of severity of periodontitis...4

Table1.2 Analysis of changes in relevant indicators in the DPSCs injection group compared to the saline injection group-grouped by severity of periodontitis........................... 5

The baseline AL was used to determine the severity of periodontitis:AL of 1-2mm was considered mild, 3-4mm was considered moderate, and ≥5mm was considered severe. Criteria for determining the severity of periodontitis: The maximum value of the two labeled data (AL-buccal/AL-lingual) was selected to determine the severity of periodontitis.

(1) Combined analysis of data from Beijing Stomatology Hospital,Cpital Medical University + Department of Stomatology, Peking university Third Hosipital

The data from the DPSCs injection group of Beijing Stomatology Hospital(33 cases) and the 1 × 10^7cells/case group of Peking university Third Hosipital(N=18 cases in the latter three groups) were combined, totaling N=51 cases of subjects; the data from the saline injection group of Beijing Stomatology Hospital(33 cases) and the saline injection group of Peking university Third Hosipital(N=9 cases) were combined, totaling N=42 cases of subjects. The statistical analysis was performed separately, and the statistical analysis indexes included AL (mean of lingual and buccal); PD (mean of lingual and buccal); GR (mean of lingual and buccal; BOP, TM, and the height of alveolar bone defects.

Between-group comparisons of the three groups were made for the DPSCs injection group of 51 subjects grouped according to mild to moderate severity of periodontitis.

1. Data merging analysis of Beijing stomatological hospital and Peking University Third Hospital

Table 1.1 Summary of periodontitis severity distribution

	DPSCs injection (N=45)	Saline injection (N=37)
Severity of periodontitis		
Light	0	0
Moderate	9(14.3)54	3(7.0)
Severe	(85.7)	40(93.0)

Program name: t_ 2_ 1.sas output result: t_ 2_ 1. RTF generation date: 2023-04-26 10:47

Table 1.2 Analysis of changes of relevant indicators in DPSCs injection group compared with saline injection group - grouped according to the severity of periodontitis

		Moderate		Severe	
		DPSCs injection (N=8)	Saline injection (N=3)	DPSCs injection (N=42)	Saline injection (N=35)
AL-The mean values of the labial and lingual sides					
Baseline	Number of teeth (missing)	9(0)	3 (0)	54 (0)	40 (0)
	Mean (SD)	3.61(0.601)	3.83 (0.289)	6.23 (1.393)	5.91 (1.377)
	Median	4.00	4.00	6.00	5.25
	Quartile	3.00,4.00	3.50, 4.00	5.50, 7.50	5.00, 7.00
	Minimum, maximum	2.5,4.0	3.5, 4.0	3.5, 9.0	4.0, 10.5
Day 90 change from baseline	Number of teeth (missing)	9(0)	3 (0)	54 (0)	40 (0)
	Mean (SD)	-0.72(0.795)	-0.67 (0.289)	-1.49 (1.276)	-1.00 (1.068)
	Median	-1.00	-0.50	-1.50	-1.00
	Quartile	-1.00,-0.50	-1.00, -0.50	-2.50, -0.50	-1.75, 0.00
	Minimum, maximum	-1.5,1.0	-1.0, -0.5	-4.5, 2.0	-4.0, 1.0
	*DPSCs injection vs Saline injection	0.9105		0.0515	
Day 180 change from baseline	Number of teeth (missing)	9(0)	3 (0)	54 (0)	40 (0)
	Mean (SD)	-0.56(0.950)	-0.67 (0.289)	-1.67 (1.508)	-1.03 (1.310)
	Median	-1.00	-0.50	-1.50	-1.00
	Quartile	-1.00,-0.50	-1.00, -0.50	-2.50, -1.00	-1.50, 0.00
	Minimum, maximum	-1.5,1.0	-1.0, -0.5	-4.5, 4.0	-5.0, 2.0
	*DPSCs injection vs Saline injection	0.8501		0.0338	

Note: Light group data are not shown because there were no subjects in the Light group. 'NE' means it cannot be calculated.

*The Tukey Kramer test in the analysis of variance was used to calculate the p value of the difference between the least squares means of the two groups,'NC'indicates not calculated.

§ Using paired chi-square test (McNemar's test),# using chi-square test /fisher exact probability method to calculate whether there is a difference in the constituent ratio of BOP between the DPSCs group and the saline control group.

| use of paired data Wilcoxon signed rank test calculation of every visit whether changes from baseline to 0 p values, | | said Wilcoxon rank and inspection is used to calculate DPSCs group with saline control group p value of the relative baseline differences.

Program name: t_ 2_ 2.sas output result: t_ 2_ 2. RTF generation date: 2023-04-26 10:47

Table 1.2 Analysis of changes of relevant indicators in DPSCs injection group compared with saline injection group - grouped according to the severity of periodontitis

		Moderate		Severe	
		DPSCs injection (N=8)	Saline injection (N=3)	DPSCs injection (N=42)	Saline injection (N=35)
PD-The mean values of the labial and lingual sides					
Baseline	Number of teeth (missing)	9 (0)	3 (0)	54 (0)	40 (0)
	Mean (SD)	3.61 (0.601)	3.83 (0.289)	5.79 (1.294)	5.61 (1.146)
	Median	2.00	2.00	3.00	2.50
	Quartile	2.00, 2.00	1.50, 2.00	2.50, 3.50	2.50, 3.50
	Minimum, maximum	1.0, 2.0	1.5, 2.0	0.5, 4.5	2.0, 4.0
Day 90 change from baseline	Number of teeth (missing)	9 (0)	3 (0)	54 (0)	40 (0)
	Mean (SD)	-0.56 (1. 130)	-0.83 (0.289)	-1.57 (1.326)	-1.06 (1.069)
	Median	-1.00	-1.00	-1.50	-1.00
	Quartile	-1.00, -0.50	-1.00, -0.50	-2.00, -0.50	-2.00, 0.00
	Minimum, maximum	-1.5, 2.0	-1.0, -0.5	-6.0, 1.5	-4.0, 0.5
	*DPSCs injection vs Saline injection	0.6913		0.0480	
Day 180 change from baseline	Number of teeth (missing)	9 (0)	3 (0)	54 (0)	40 (0)
	Mean (SD)	-0.67 (1. 118)	-0.83 (0.289)	-1.81 (1.490)	-1.08 (1.289)
	Median	-1.00	-1.00	-2.00	-1.00
	Quartile	-1.50, -0.50	-1.00, -0.50	-3.00, -1.00	-1.50, -0.25
	Minimum, maximum	-1.5, 2.0	-1.0, -0.5	-5.0, 3.0	-5.0, 2.0
	*DPSCs injection vs Saline injection	0.8092		0.0147	

Note: Light group data are not shown because there were no subjects in the Light group. 'NE' means it cannot be calculated.
*The Tukey Kramer test in the analysis of variance was used to calculate the p value of the difference between the least squares means of the two groups,'NC'indicates not calculated.
§ Using paired chi-square test (McNemar's test),# using chi-square test /fisher exact probability method to calculate whether there is a difference in the constituent ratio of BOP between the DPSCs group and the saline control group.
| use of paired data Wilcoxon signed rank test calculation of every visit whether changes from baseline to 0 p values, | | said Wilcoxon rank and inspection is used to calculate DPSCs group with saline control group p value of the relative baseline differences.
Program name: t_ 2_ 2.sas output result: t_ 2_ 2. RTF generation date: 2023-04-26 10:47

Table 1.2 Analysis of changes of relevant indicators in DPSCs injection group compared with saline injection group - grouped according to the severity of periodontitis

		Moderate		Severe	
		DPSCs injection (N=8)	Saline injection (N=3)	DPSCs injection (N=42)	Saline injection(N=35)
GR-The mean values of the labial and lingual sides					
Baseline	Number of teeth (missing)	9 (0)	3 (0)	54 (0)	40 (0)
	Mean (SD)	0.00 (0.000)	0.00 (0.000)	0.44 (0.805)	0.31 (0.676)
	Median	0.00	0.00	0.00	0.00
	Quartile	0.00, 0.00	0.00, 0.00	0.00, 1.00	0.00, 0.50
	Minimum, maximum	0.0, 0.0	0.0, 0.0	-1.5, 3.0	-1.5, 3.0
Day 90 change from baseline	Number of teeth (missing)	9 (0)	3 (0)	54 (0)	40 (0)
	Mean (SD)	0.06 (0. 167)	0. 17 (0.289)	0.08 (0.657)	0.06 (0.483)
	Median	0.00	0.00	0.00	0.00
	Quartile	0.00, 0.00	0.00, 0.50	0.00, 0.00	0.00, 0.00
	Minimum, maximum	0.0, 0.5	0.0, 0.5	-3.0, 2.0	-1.0, 2.0
	*DPSCs injection vs Saline injection	0.4178		0.8658	
Day 180 change from baseline	Number of teeth (missing)	9 (0)	3 (0)	54 (0)	40 (0)
	Mean (SD)	0.22 (0.441)	0. 17 (0.289)	0. 12 (0.726)	0.05 (0.336)
	Median	0.00	0.00	0.00	0.00
	Quartile	0.00, 0.00	0.00, 0.50	0.00, 0.50	0.00, 0.00
	Minimum, maximum	0.0, 1.0	0.0, 0.5	-2.0, 2.0	-0.5, 1.0
	*DPSCs injection vs Saline injection	0.8449		0.5709	

Note: Light group data are not shown because there were no subjects in the Light group. 'NE' means it cannot be calculated.
*The Tukey Kramer test in the analysis of variance was used to calculate the p value of the difference between the least squares means of the two groups,'NC'indicates not calculated.
§ Using paired chi-square test (McNemar's test),# using chi-square test /fisher exact probability method to calculate whether there is a difference in the constituent ratio of BOP between the DPSCs group and the saline control group.
| use of paired data Wilcoxon signed rank test calculation of every visit whether changes from baseline to 0 p values, | | said Wilcoxon rank and inspection is used to calculate DPSCs group with saline control group p value of the relative baseline differences.
Program name: t_ 2_ 2.sas output result: t_ 2_ 2. RTF generation date: 2023-04-26 10:47

Table 1.2 Analysis of changes of relevant indicators in DPSCs injection group compared with saline injection group - grouped according to the severity of periodontitis

		Moderate		Severe	
		DPSCs injection (N=8)	Saline injection (N=3)	DPSCs injection (N=42)	Saline injection(N=35)
Height of alveolar bone defect (mm)					
Baseline	Number of teeth (missing)	9 (0)	3 (0)	54 (0)	40 (0)
	Mean (SD)	1.42 (0.974)	1.20 (0.693)	2.51 (1.283)	2.03 (1.460)
	Median	0.80	1.60	2.25	1.65
	Quartile	0.70, 2.20	0.40, 1.60	1.60, 3.20	1. 10, 2.45
	Minimum, maximum	0.6, 3.3	0.4, 1.6	0.6, 7.7	0.5, 7.7
Day 90 change from baseline	Number of teeth (missing)	9 (0)	3 (0)	54 (0)	40 (0)
	Mean (SD)	-0. 13 (0.374)	-0.30 (0. 173)	-0. 16 (0.383)	-0.08 (0.314)
	Median	-0. 10	-0.20	-0. 10	-0. 10
	Quartile	-0. 10, 0. 10	-0.50, -0.20	-0.30, 0. 10	-0.30, 0. 10
	Minimum, maximum	-0.9, 0.4	-0.5, -0.2	-1.4, 0.5	-0.8, 0.7
	*DPSCs injection vs Saline injection	0.4834		0.2519	
Day 180 change from baseline	Number of teeth (missing)	9 (0)	3 (0)	54 (0)	40 (0)
	Mean (SD)	-0. 18 (0.335)	-0.30 (0.265)	-0.24 (0.471)	-0.02 (0.348)
	Median	-0. 10	-0.20	-0.20	0.00
	Quartile	-0.20, -0. 10	-0.60, -0. 10	-0.50, 0. 10	-0. 15, 0. 15
	Minimum, maximum	-0.9, 0.3	-0.6, -0. 1	-1.7, 0.6	-0.9, 0.6
	*DPSCs injection vs Saline injection	0.5814		0.0147	

Note: Light group data are not shown because there were no subjects in the Light group. 'NE' means it cannot be calculated.
*The Tukey Kramer test in the analysis of variance was used to calculate the p value of the difference between the least squares means of the two groups,'NC'indicates not calculated.
§ Using paired chi-square test (McNemar's test),# using chi-square test /fisher exact probability method to calculate whether there is a difference in the constituent ratio of BOP between the DPSCs group and the saline control group.
| use of paired data Wilcoxon signed rank test calculation of every visit whether changes from baseline to 0 p values, | | said Wilcoxon rank and inspection is used to calculate DPSCs group with saline control group p value of the relative baseline differences.
Program name: t_ 2_ 2.sas output result: t_ 2_ 2. RTF generation date: 2023-04-26 10:47

Table 1.2 Analysis of changes of relevant indicators in DPSCs injection group compared with saline injection group - grouped according to the severity of periodontitis

		Moderate		Severe	
		DPSCs injection (N=8)	Saline injection(N=3)	DPSCs injection (N=42)	Saline injection(N=35)
BOP,n(%)					
Baseline	Bleeding	8 (88.89%)	3 (100.00%)	54 (100.00%)	38 (95.00%)
	No bleeding	1 (11. 11%)			2 (5.00%)
Day 90	Bleeding	7 (77.78%)	3 (100.00%)	51 (94.44%)	38 (95.00%)
	No bleeding	2 (22.22%)		3 (5.56%)	2 (5.00%)
	§P value for comparison within groups	0.5637	NC	0.0833	1.0000
	#DPSCs injection vs Saline injection	1.0000		1.0000	
Day 180	Bleeding	6 (66.67%)	2 (66.67%)	49 (90.74%)	39 (97.50%)
	No bleeding	3 (33.33%)	1 (33.33%)	5 (9.26%)	1 (2.50%)
	§P value for comparison within groups	0.3173	0.3173	0.0253	0.5637
	#DPSCs injection vs Saline injection	1.0000		0.2156	
TM,n(%)					
Baseline	No loosening	7 (77.78%)	3 (100.00%)	36 (66.67%)	34 (85.00%)
	I°	2 (22.22%)		15 (27.78%)	4 (10.00%)
	II°			3 (5.56%)	2 (5.00%)
Day 90	No loosening	8 (88.89%)	3 (100.00%)	42 (77.78%)	34 (85.00%)
	I°	1 (11. 11%)		11 (20.37%)	4 (10.00%)
	II°			1 (1.85%)	2 (5.00%)

Note: Light group data are not shown because there were no subjects in the Light group. 'NE' means it cannot be calculated.
*The Tukey Kramer test in the analysis of variance was used to calculate the p value of the difference between the least squares means of the two groups,'NC'indicates not calculated.
§ Using paired chi-square test (McNemar's test),# using chi-square test /fisher exact probability method to calculate whether there is a difference in the constituent ratio of BOP between the DPSCs group and the saline control group.
| use of paired data Wilcoxon signed rank test calculation of every visit whether changes from baseline to 0 p values, | | said Wilcoxon rank and inspection is used to calculate DPSCs group with saline control group p value of the relative baseline differences.
Program name: t_ 2_ 2.sas output result: t_ 2_ 2. RTF generation date: 2023-04-26 10:47

Table 1.2 Analysis of changes of relevant indicators in DPSCs injection group compared with saline injection group - grouped according to the severity of periodontitis

		Moderate		Severe	
		DPSCs injection (N=8)	Saline injection (N=3)	DPSCs injection(N=42)	Saline injection (N=35)
	| P value for comparison within groups	1.0000	.	0.0557	1.0000
	|| DPSCs injection vs Saline injection	0.5637		0.2001	
Day 180	No loosening	9(100.00%)	3(100.00%)	49(90.74%)	34(85.00%)
	I°			4(7.41%)	4(10.00%)
	II°			1(1.85%)	2(5.00%)
	| P value for comparison within groups	0.5000	.	0.0015	1.0000
	|| DPSCs injection vs Saline injection	0.3918		0.0182	

Note: Light group data are not shown because there were no subjects in the Light group. 'NE' means it cannot be calculated.*The Tukey Kramer test in the analysis of variance was used to calculate the p value of the difference between the least squares means of the two groups,'NC'indicates not calculated.§ Using paired chi-square test (McNemar's test),# using chi-square test /fisher exact probability method to calculate whether there is a difference in the constituent ratio of BOP between the DPSCs group and the saline control group.| use of paired data Wilcoxon signed rank test calculation of every visit whether changes from baseline to 0 p values, | | said Wilcoxon rank and inspection is used to calculate DPSCs group with saline control group p value of the relative baseline differences.Program name: t_ 2_ 2.sas output result: t_ 2_ 2. RTF generation date: 2023-04-26 10:47
